# Influence of scanbody design and intraoral scanner on the trueness of complete arch implant digital impressions: An *in vitro* study

**DOI:** 10.1371/journal.pone.0295790

**Published:** 2023-12-19

**Authors:** Priscila Ceolin Meneghetti, Junying Li, Paulo Sérgio Borella, Gustavo Mendonça, Luiz Henrique Burnett

**Affiliations:** 1 School of Health and Life Sciences, Pontifical Catholic University of Rio Grande do Sul, Porto Alegre, Rio Grande do Sul, Brazil; 2 Department of Biological and Materials Sciences & Prosthodontics, University of Michigan School of Dentistry, Ann Arbor, Michigan, United States of America; 3 Department of General Practice, Virginia Commonwealth University School of Dentistry, Richmond, Virginia, United States of America; 4 Department of Occlusion, Fixed Prosthodontics, and Dental Materials, Federal University of Uberlândia, Uberlândia, Minas Gerais, Brazil; Yerevan State Medical University Named after Mkhitar Heratsi, ARMENIA

## Abstract

This study aimed to compare the accuracy of full-arch digital implant impressions using seven different scanbodies and four intraoral scanners. A 3D-printed maxillary model with six implants and their respective multi-unit abutments was used for this study. Seven scanbodies (SB1, SB2, SB3, SB4, SB5, SB6, and SB7) and four intraoral scanners (Primescan®, Omnican®, Trios 3®, and Trios 4®) were assessed. Each combination group was scanned ten times and a dental lab scanner (D2000, 3Shape) was used as a reference. All scans were exported as STL files, imported into Convince software (3Shape) for alignment, and later into Blender software, where their 3D positions were analyzed using a Python script. The 3D deviation, angular deviation, and linear distance between implants #3 and #14 were also measured. Accuracy was measured in terms of “trueness” (scanbody 3D deviation between intraoral scan and desktop scan). Kruskal-Wallis followed by the Bonferroni correction was used to analyze the data (⍺ = .05). The study found statistically significant differences in digital impression accuracy among the scanners and scanbodies (p<0.001). When comparing different intraoral scanners, the Primescan system showed the smallest 3D deviation (median 110.59 μm) and differed statistically from the others, while Trios 4 (median 122.35 μm) and Trios 3 (median 130.62 μm) did not differ from each other (p = .284). No differences were found in the linear distance between implants #3 and #14 between Trios 4, Primescan, and Trios 3 systems. When comparing different scanbodies, the lowest median values for 3D deviation were obtained by SB2 (72.27μm) and SB7 (93.31μm), and they did not differ from each other (p = .116). The implant scanbody and intraoral scanner influenced the accuracy of digital impressions on completely edentulous arches.

## Introduction

The digital workflow in implant dentistry offers numerous advantages over conventional methods, such as reducing errors from impression material distortion, simplifying production processes, lowering costs, and improving patient acceptance [1, 2]. Furthermore, digital impressions have revolutionized the field of dentistry by providing a more efficient, accurate, and patient-friendly alternative to conventional impressions, promoting a more predictable workflow for implant-supported restorations [3–5].

An accurate implant impression is a foundation for fabricating a good implant restoration [6]. Thus, assessing and ensuring the accuracy of digital impressions is essential for achieving optimal clinical outcomes in implant-supported restorations. The role of Intraoral Scanner (IOS) systems in achieving appropriately fitting implant-supported prostheses is significant, with trueness and precision being key determinants [7–11].

According to the International Organization for Standardization (ISO) standards, accuracy is a composite of both trueness and precision. Trueness is defined as the proximity of a measurement to the actual value of the object under measurement. It also declares that a superior level of trueness implies a close match between the measurement and the actual dimensions of the object. Conversely, precision pertains to the consistency or repeatability of measurements. An elevated level of precision signifies that repeated measurements closely align, irrespective of their concurrence with the object’s actual value.

While the accuracy of implant digital impressions is well established in the literature for single and partial cases [9], complete arch implant scanning remains a challenge [7, 10, 12–14]. The accuracy of implant digital impressions is a multifactorial process influenced by several variables, including the type of IOS used, scanning strategy [14, 15], the position, number, and distance of implants, the level of operator experience, and the design of the scanbodies (SBs) used to transfer the 3D position of implants or abutments in the arch [7, 8, 14, 16–28]. The correct acquisition of implant positions by SBs depends on factors such as shape, material, color, and alignment between the captured mesh and the.STL file used in the software [16, 17, 20, 22, 25, 26, 29, 30]. However, it’s important to note that factors such as implant angulation, implant connections, and implant depth have no significant effect [31].

Therefore, the accurate assessment and assurance of digital impressions are critical for achieving optimal clinical outcomes in implant-supported restorations. Implant-supported restorations that do not fit properly can lead to additional time needed for adjustments and can also create tension at the connection points between the bone and implant, as well as between the implant and the prosthetic structure. This tension has the potential to result in harmful biological and technical issues [32, 33].

Thus, this in vitro study aimed to investigate the influence of SB design and IOS system on the trueness of digital impressions for complete-arch implant-supported restorations. Seven different SB designs and four different IOS systems were tested, and the distance and angular deviations were measured from a reference scanning obtained by an accurate bench scanner. The study also investigated whether changes in the linear distance between implants #3 and #14 occurred with different SB designs and IOS systems.

The null hypothesis for the study is that there would be no significant difference in the overall trueness of digital impressions for complete-arch implant-supported restorations between different SB designs and IOS systems when measuring the linear distance and angular deviation.

## Materials and methods

### Master model

**Fig 1:**
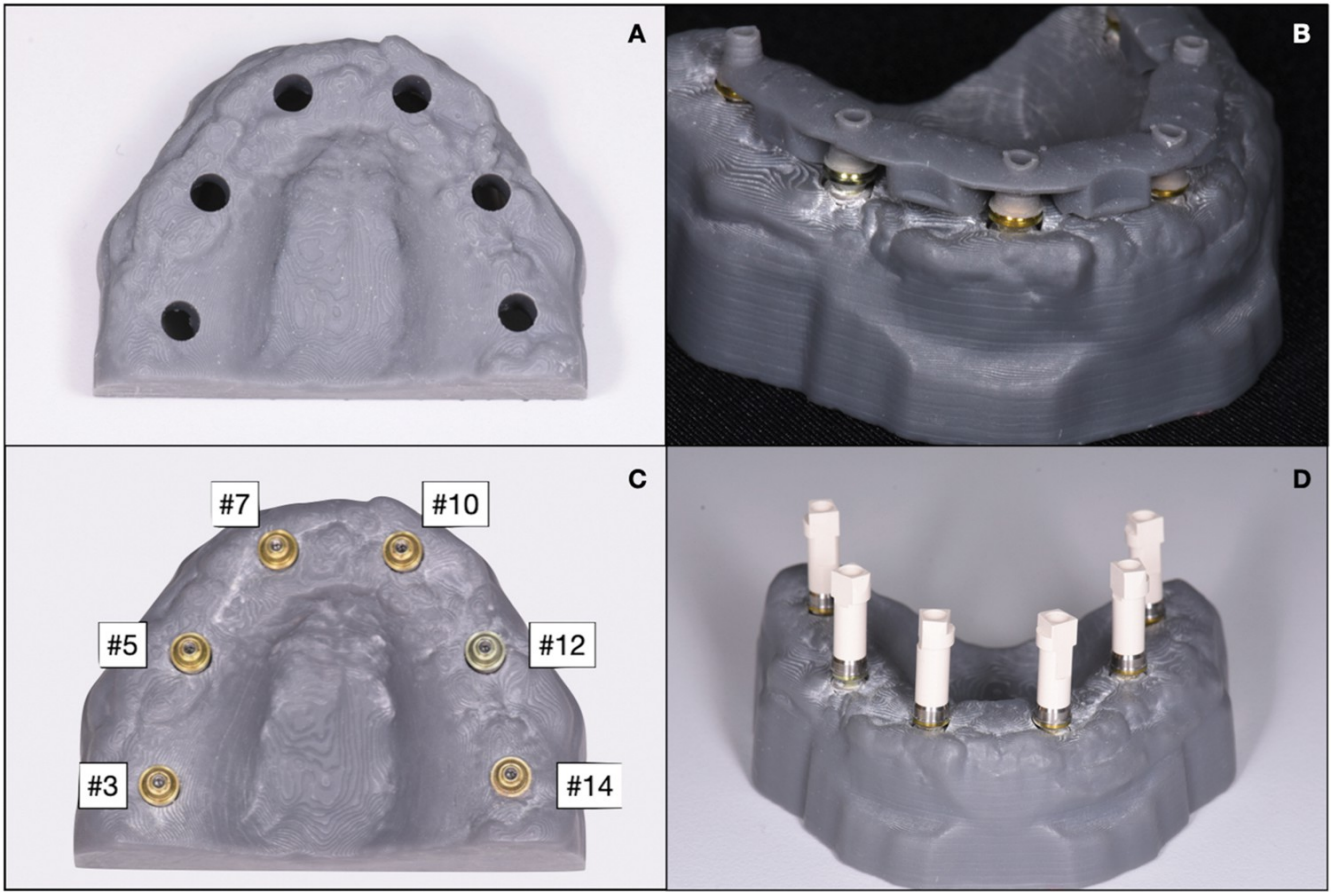
Master model. (A) 3D printed maxillary model with 6 roles. (B) Implants and respective multi-unit abutments installed with the position guide. (C) A master model with multi-unit abutments. (D) A master model with the SB1 group attached.

A master model was created using a reference Standard Tessellation Language (STL) file of an edentulous maxilla, which was modified with Meshmixer software (Autodesk, Inc). The software was used to create six parallel holes corresponding to the implant positions of the first molars (#3 and #14), pre-molars (#5 and #7), and lateral incisors (#10 and #12) (Fig 1A).

The model and an index position guide were 3D printed on the Form2 3D Printer (FormLabs, Somerville, MA, USA) using grey resin material (Formlabs) with a thickness of 100μm. After printing, they were cleaned in 92% isopropyl alcohol for 20 minutes using Form Wash (FormLabs), then cured in an ultraviolet light chamber for 15 minutes with Form Cure (FormLabs).

Multi-unit abutments were hand-tightened into the implants (4.1mm x 10mm, S.I.N. Implant System, Brazil), and with the position guide, the set was placed and secured in the printed model using acrylic resin (Pattern resin LS, GC, Tokyo, Japan) (Fig 1B and 1C). The placement was below the gingival margin and the abutments were parallel to each other.

### Reference scanning

**Fig 2:**
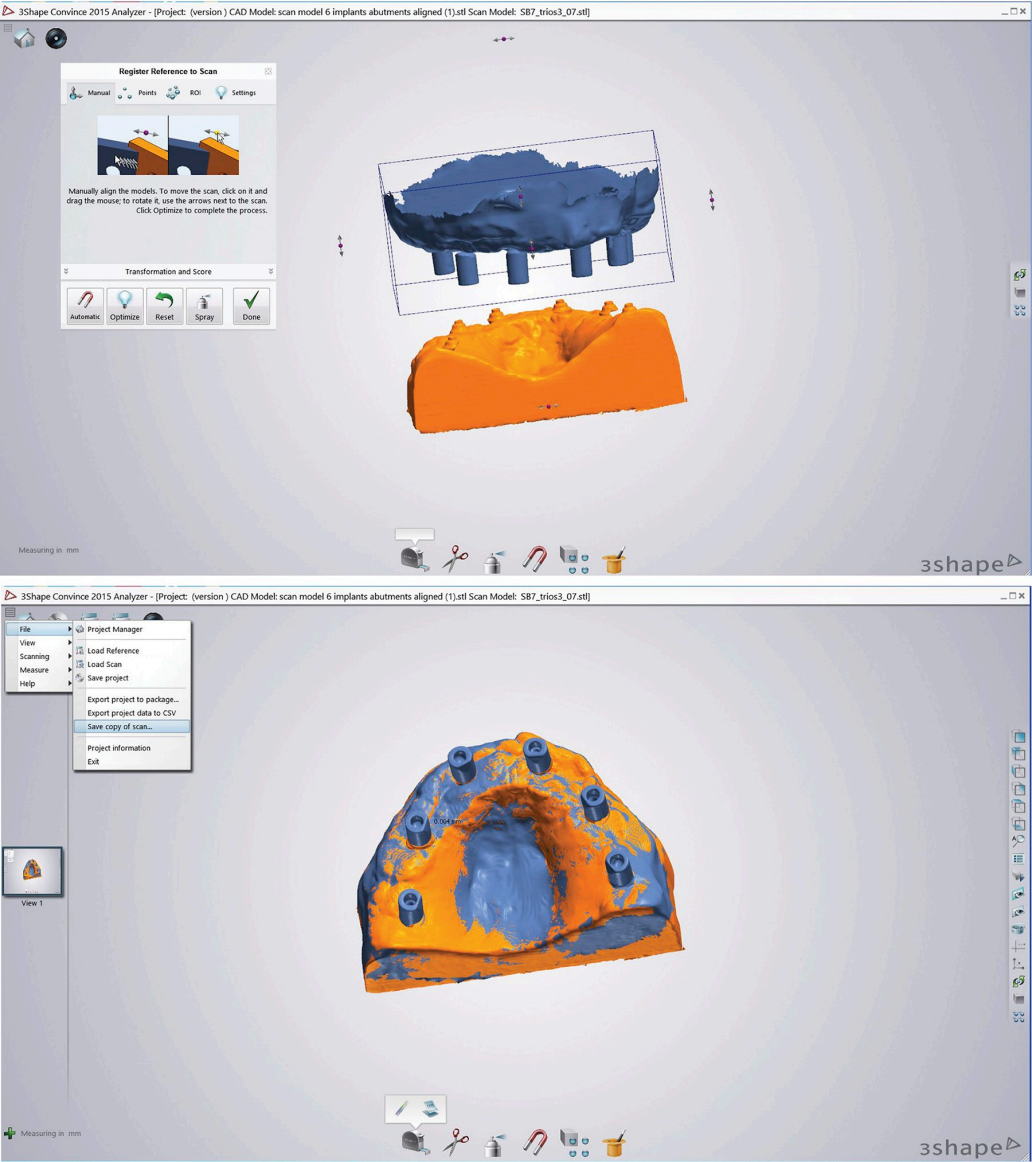
Test model alignment. (A). The baseline model used as a reference to align all scans on Convince System 2015 Analyzer. (B) Scan aligned and ready to be saved and exported.

The master model (model with multi-unit abutments) was scanned using a desktop scanner (D2000 Dental Desktop, 3Shape, Copenhagen, Denmark) to establish a common reference point for aligning all the STL files. This master model served as a consistent frame of reference, ensuring that all digital impressions were in the same 3D position for subsequent measurements. This step was essential for maintaining a consistent and standardized reference for our analysis. Then, the model with each set of scanbody tested were scanned with the bench scanner as a reference to align each scanbody. All files were exported in Standard Tessellation Language (.STL) format for further processing. The alignment with the master model was done using reverse engineering software (Convince System 2015 Analyzer, 3Shape, Inc., Copenhagen, Denmark). Automatic alignment was performed and optimized (Fig 2A and 2B).

### Scanbodies, intraoral scanners and scanning process

To assess the impact of SB design on trueness, the study examined four different commercially available sets of SBs and three new designs developed by the authors based on earlier observations during the pilot project. The new SBs designs were obtained in 3D software (Meshmixer). Then, the new SBs were 3D printed on the Form2 3D Printer (FormLabs, Somerville, MA, USA) using grey resin material (Formlabs) with a thickness of 100μm. After printing, they were cleaned in 92% isopropyl alcohol for 20 minutes using Form Wash (FormLabs), then cured in an ultraviolet light chamber for 15 minutes with Form Cure (FormLabs).

**Fig 3:**
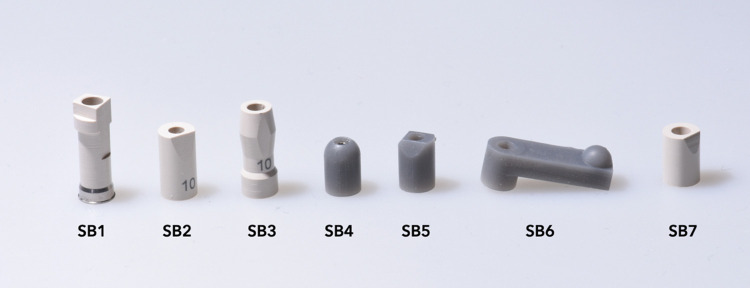
Implant SBs used in the study. PEEK materials represent commercial brands, and grey materials represent SBs design and 3D printed by the authors.

The SBs created by the authors consisted of three different types. The first type (SB4) had a cylindrical shape with a height of 7mm and a rounded top. The second type (SB5) also had a cylindrical shape with a height of 7mm, with three flat surfaces on the top arranged at an angle to one another. The third type (SB6) was a bar-shaped SB with a length of 16 mm and a convex half-circle shape at its ends. Table 1 and Fig 3 list the specific SBs analyzed in the study.

**Table 1:** Scanbodies characteristics.

Group	Design	Material	Manufacturer
SB1	Cylinder, with a trapezoidal in the top, with different surfaces. 14 mm high	PEEK* with a metal connection	S.I.N., São Paulo, Brazil
SB2	Cylinder with an angled flat surface, 9 mm high	PEEK*	Neodent, Curitiba, Brazil
SB3	Cylinder with diamond surfaces, 12 mm high	PEEK*	Neodent, Curitiba, Brazil
SB4	Prototype, rounded, 7 mm high	3D printed, grey resin	Custom
SB5	Prototype, tri-angled flat surfaces, 7 mm high	3D printed, grey resin	Custom
SB6	Prototype, bar 16 mm with a convex ball in the extremity	3D printed, grey resin	Custom
SB7	Cylinder with an angled flat surface, 7 mm high	PEEK*	S.I.N., São Paulo, Brazil

*PEEK, polyetheretherketone.

Each set of SBs was manually screwed onto the multi-unit abutments and hand-tightened (Fig 1D). The sample size for this study was determined by drawing upon previous projects in our field [5, 22, 23, 25, 29], as well as conducting a pilot study using Sigma Plot software. The pilot study revealed that a minimum of 8 specimens per group was necessary to meet our study’s predefined statistical criteria. Consequently, in this research, we conducted ten consecutive digital scans (n = 10) for each group of study subjects, employing all four Intraoral Scanners (IOS) under evaluation: PRIMESCAN®, OMNICAM®, TRIOS 3®, and TRIOS 4®. All scanners were calibrated before use, and scans were performed by the same experienced operator. The technical specifications of each scanner used in the study are summarized in Table 2.

**Table 2:** Digital scanners characteristics.

Name	Manufacturer	Acquisition	Output files
OMNICAM^®^	Dentsply Sirona, York, PA USA	Optical triangulation and Confocal Microscopy	cs3, sdt, cdt, idt (proprietary format) with the possibility to export.stl files (open format) with Cerec Connect
PRIMESCAN^®^	Dentsply Sirona, York, PA USA	High-resolution Sensors and Shortwave Light with Optical High-Frequency Contrast Analysis for Dynamic Deep Scan (20mm)	Dxd (property format) with the possibility to export.stl files (open format) with Cerec Connect.
TRIOS 3^®^	3Shape, Copenhagen, Denmark	Confocal Microscopy and Ultrafast Optical Scanning	Dcm (propriety format), with the possibility to export.stl files (open formats with Trios on Dental Desktop).
TRIOS 4^®^	3Shape, Copenhagen, Denmark	Confocal Microscopy and Ultrafast Optical Scanning	Dcm (propriety format), with the possibility to export.stl files (open formats with Trios on Dental Desktop.
D2000	3Shape, Copenhagen, Denmark	4 high-resolution 5.0 MP cameras; Blue LED Multi-line; Accuracy 5 μm (ISO 12836) / 8 μm (Implant bar)	Color scanning of textures; Integrated multi-die scan technology.

**Fig 4:**
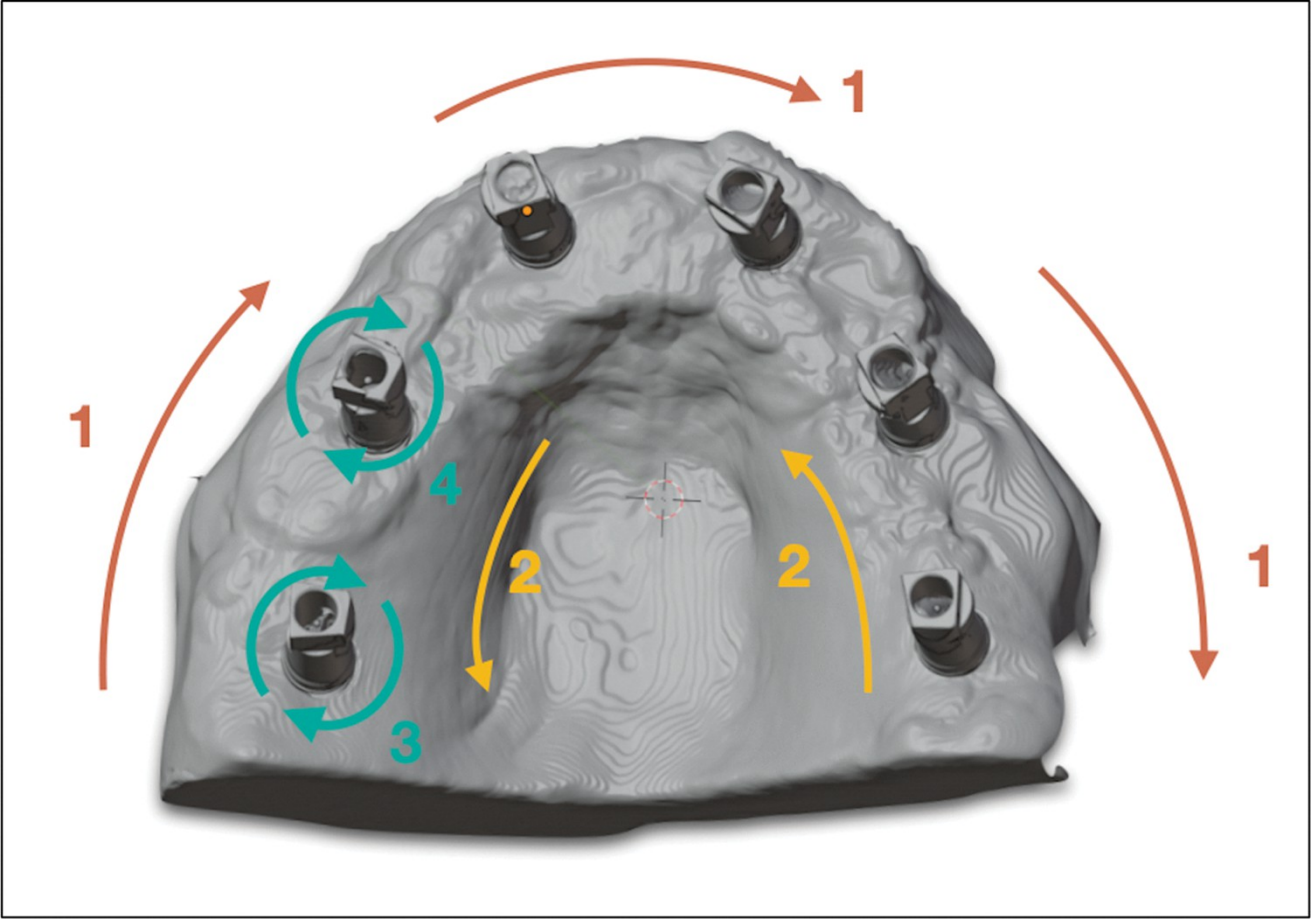
Scanning strategy. Schematic steps of the scanning strategy adopted.

The following scanning strategy was used: starting with the occlusal/buccal surface of the SB from #3 to #14. The scanner tip was then rotated to the buccal surface at a 45-degree angle and returned to SB #3, following the lingual surface to the other side. A complimentary scan was also performed around each ISB in circles (Fig 4).

All virtual model meshes were inspected to ensure the absence of voids and missing areas. Afterward, the digitized data were exported as.STL files using each scanner’s conversion software. For Omnicam and Primescan, the STL files were exported with the maximum resolution to ensure the accuracy of the files. The 3Shape files (D2000, Trios 3, and Trios 4) did not offer the option to select the quality of.STL files.

### Model alignment

**Fig 5:**
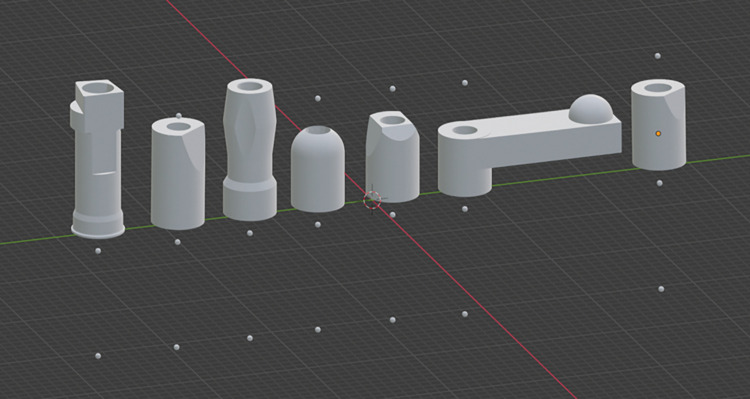
Scanbodies tested. Scanbodies mesh files with dots.

The.STL files generated by the scans were aligned with the baseline model using reverse engineering software (Convince System 2015 Analyzer, 3Shape, Inc., Copenhagen, Denmark), ensuring all models were in the same 3D position for future measurements, as mentioned before. Additionally, dots were create and added to the files to identify the long axis of the implant/scanbody using Blender Software, as shown in Fig 5. The dot that is marked in orange in SB8 (Fig 5) and it is hidden in the others, was indeed the one utilized to measure the distance and angle between the dots of the tested scan body and the reference scan using a Python scrip. This dot was strategically placed and associated with a specific geometrical point on the implant, multi-unit position and scanbody. Using these dots, each scan body was aligned one by one with the digital impression. This approach ensured that accurately assess the alignment and fit of each scan body in comparison to the reference model.

**Fig 6:**
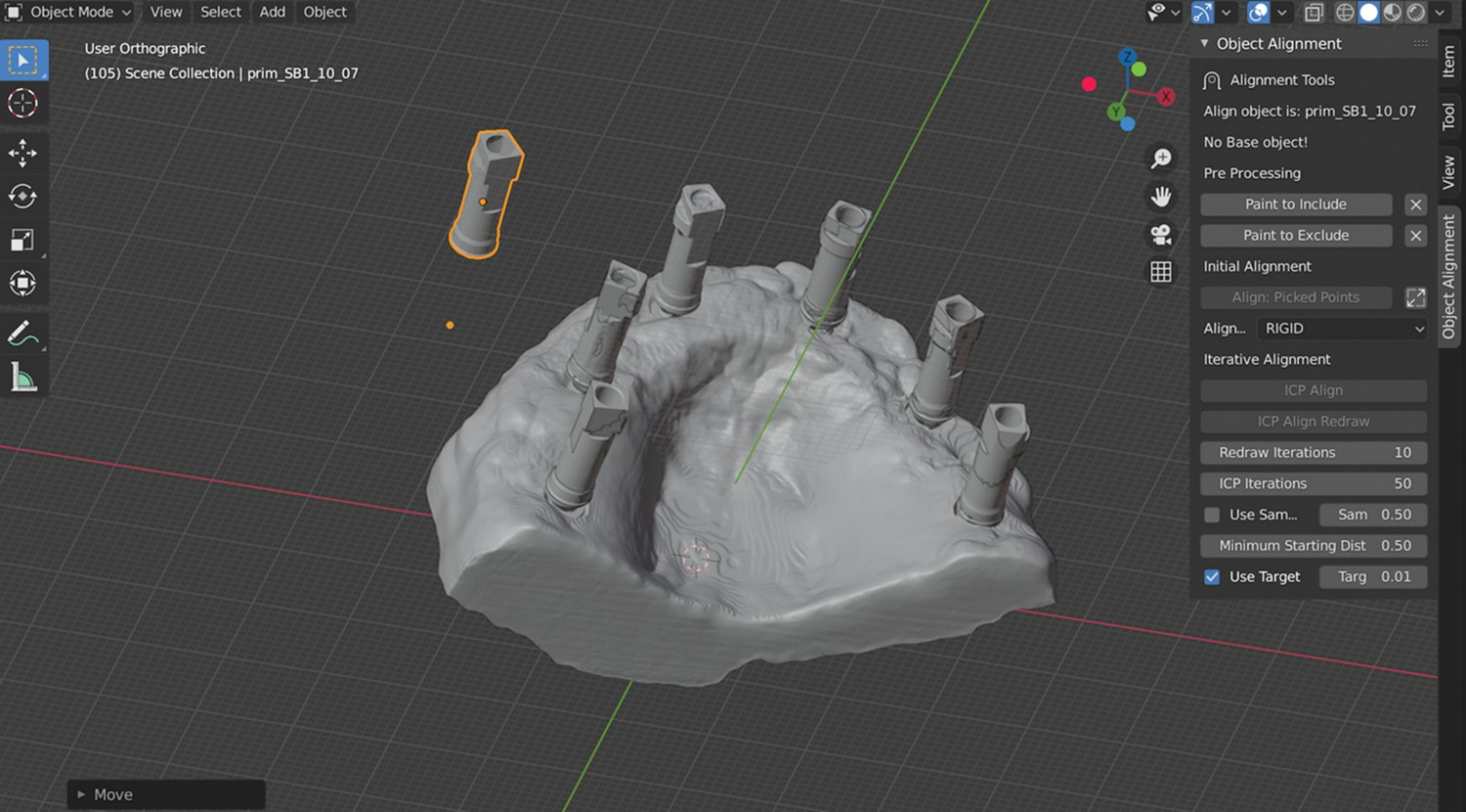
Scanbody alignment. The mesh with dots alignment to the full arch scan on Blender Software using the ICP align tool.

**Fig 7:**
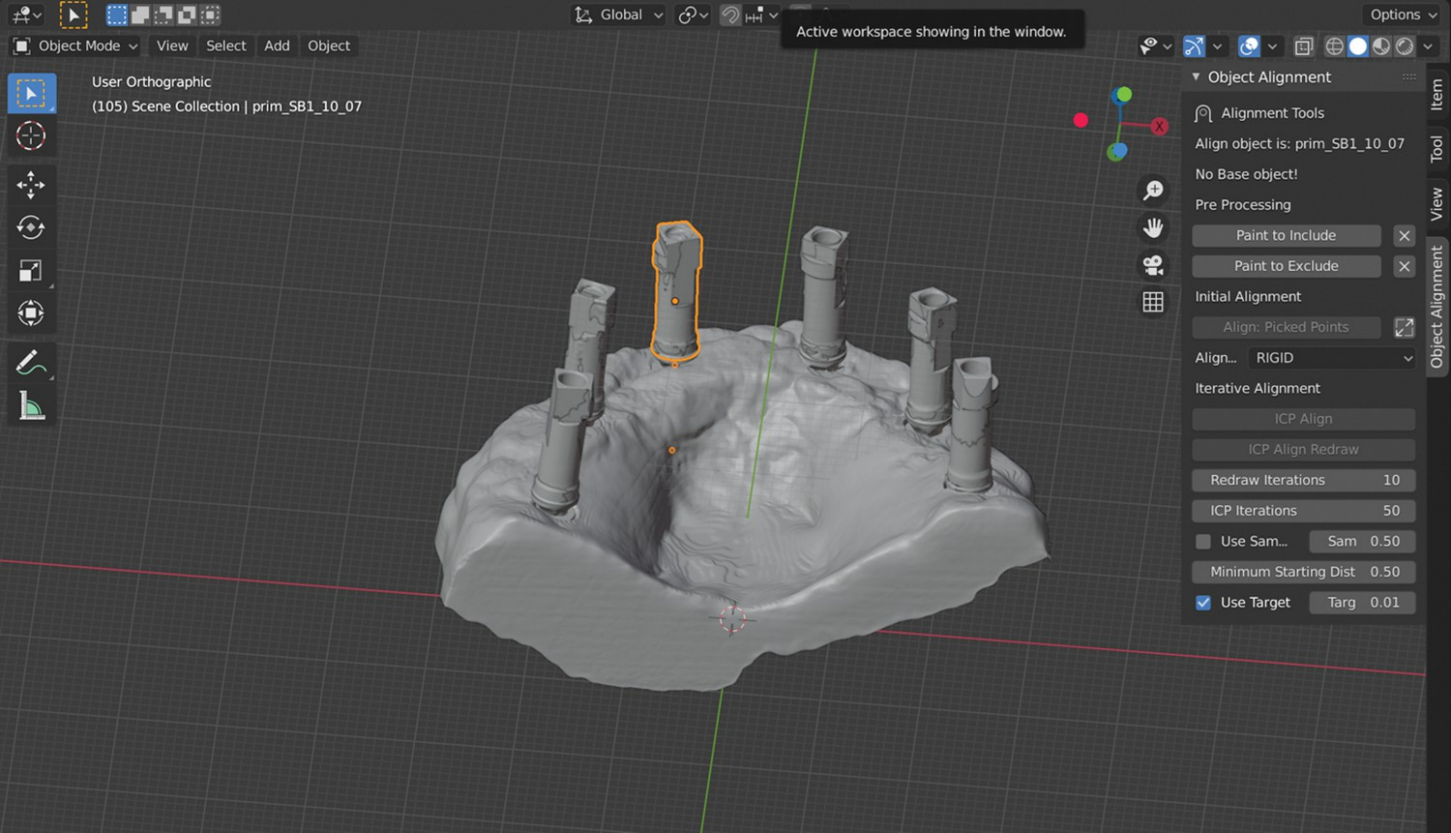
Scanbody alignment. Scanbody mesh after alignment on Blender Software using the ICP Align tool.

Those SBs with dots were aligned both in the reference and experimental scans using an ADD-ON for Blender called Object align/ICP align (Figs 6 and 7). This ADD-ON allows more precisely aligning the SBs with dots on the models.

An automated Python script (S1 and S2 Files) for a measurement tool (MeasureIt) in Blender software was developed to allow the calculation of the distance between the dot in the test scans to the corresponding dot in the reference scan. The distance from dot of the SB #3 in the test scan was measured to the dot in the SB #3 in the reference scan, and so on. This allowed it to measure the 3D deviation. 3D deviation refers to the overall spatial variation or difference between two 3D models, often measured in terms of the magnitude and direction of changes in position, orientation, or shape. In our study, it is used to assess the global misalignment or variation between the scan bodies and the reference model in three-dimensional space.

The angular deviation was evaluated considering the angle between the central axis of each SB and the corresponding axis in the reference data. The linear distance deviation specifically focuses on the variation in the straight-line distance between two points in a 3D space. It quantifies the difference in the separation between two specific locations, in this study, specifically was measured the linear distance between implants #3 and #14. In the end, the script generated a worksheet file (Microsoft Excel) containing all data. The scripts are available in supplementary data.

### Statistical analysis

Trueness was obtained by comparing the data from the test and the reference for each group. Statistical analyses were performed with a statistical software program (IBM SPSS Statistics, v20.0; IBM Corp). Since the Shapiro-Wilk test showed that the data were not normally distributed, the Kruskal-Wallis test followed by Bonferroni correction were used for the analysis (α = .05). The distribution shape, Chi-Square values, degrees of freedom, and p-values are also available in the S3 File for reference.

## Results

**Fig 8:**
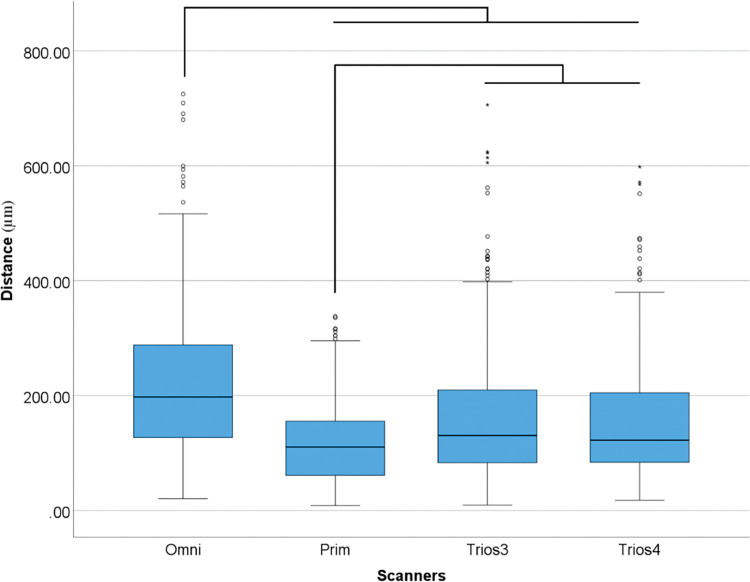
Overall scanners data (μm) in 3D deviation. Boxplot graphic showing differences between scanners for 3D deviation. Lines connecting groups indicate significant differences between groups after Bonferroni correction (P < .005).

The performed tests found significant differences in the scanners (p<0.001) and SBs (p<0.001). The tested intra oral scanners and SB designs also affect the 3D deviation (p<0.001), angular deviation (p<0.001), and linear distance deviation between implants #3–14 (p<0.001). Overall differences (p<0.001) within scanners are following described (Table 3 and Fig 8).

**Table 3:** Descriptive analyses of overall differences among scanners for 3D deviation in medians.

	*Distance (μm)*
*Omni*	197.7
*Prime*	110.59
*Trios 3*	130.62
*Trios 4*	122.35

**Fig 9:**
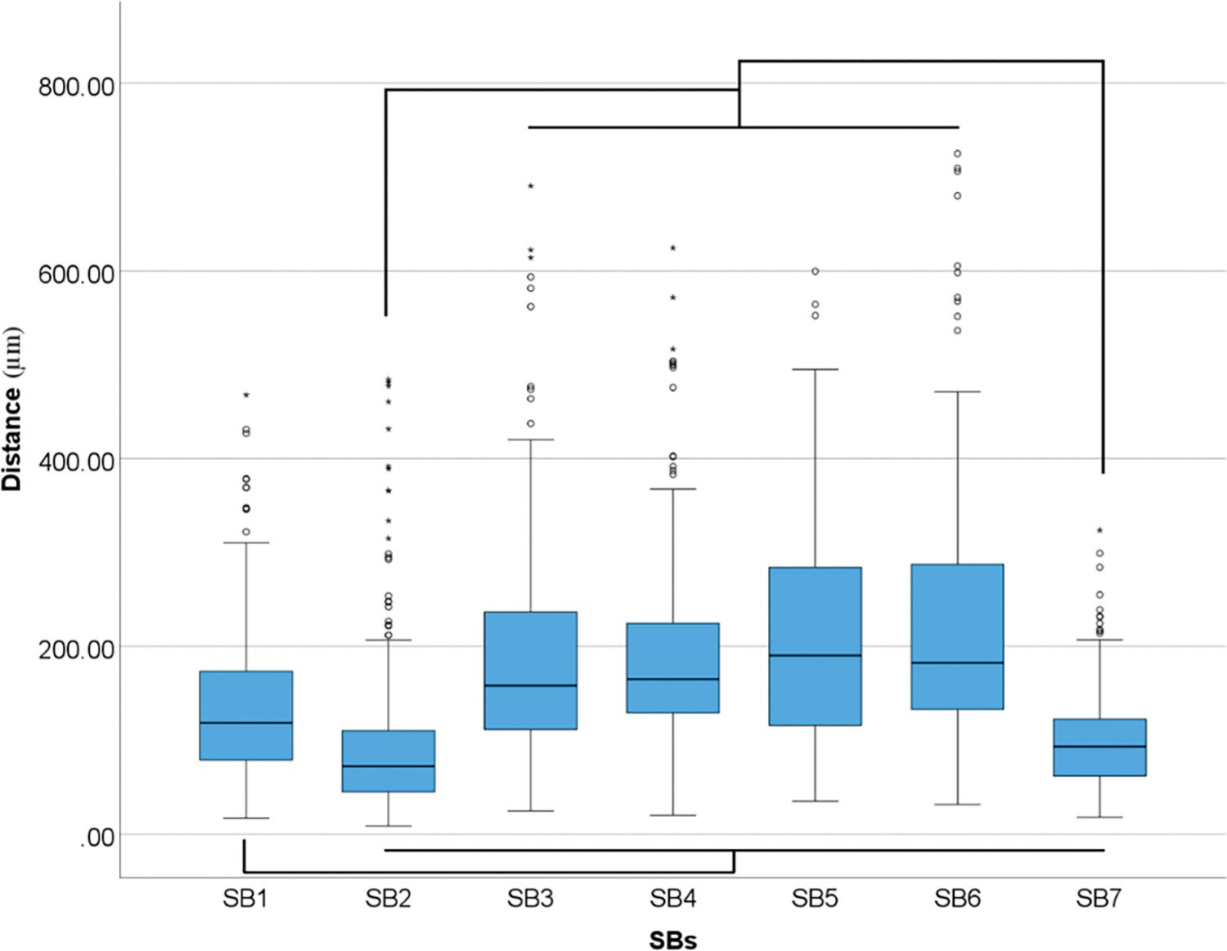
Overall scanbodies data (μm) in 3D deviation. Boxplot graphic showing differences between scanbodies for 3D deviation. Lines connecting groups indicate significant differences between groups after Bonferroni correction (P < .005).

Overall differences were found within SBs in linear deviation (p<0.001) (Table 4 and Fig 9).

**Table 4:** Descriptive analyses of overall differences among SBs for 3D deviation in median.

	*Distance (μm)*
*SB1*	118.47
*SB2*	72.27
*SB3*	158.23
*SB4*	165.08
*SB5*	190.29
*SB6*	182.51
*SB7*	93.31

**Fig 10:**
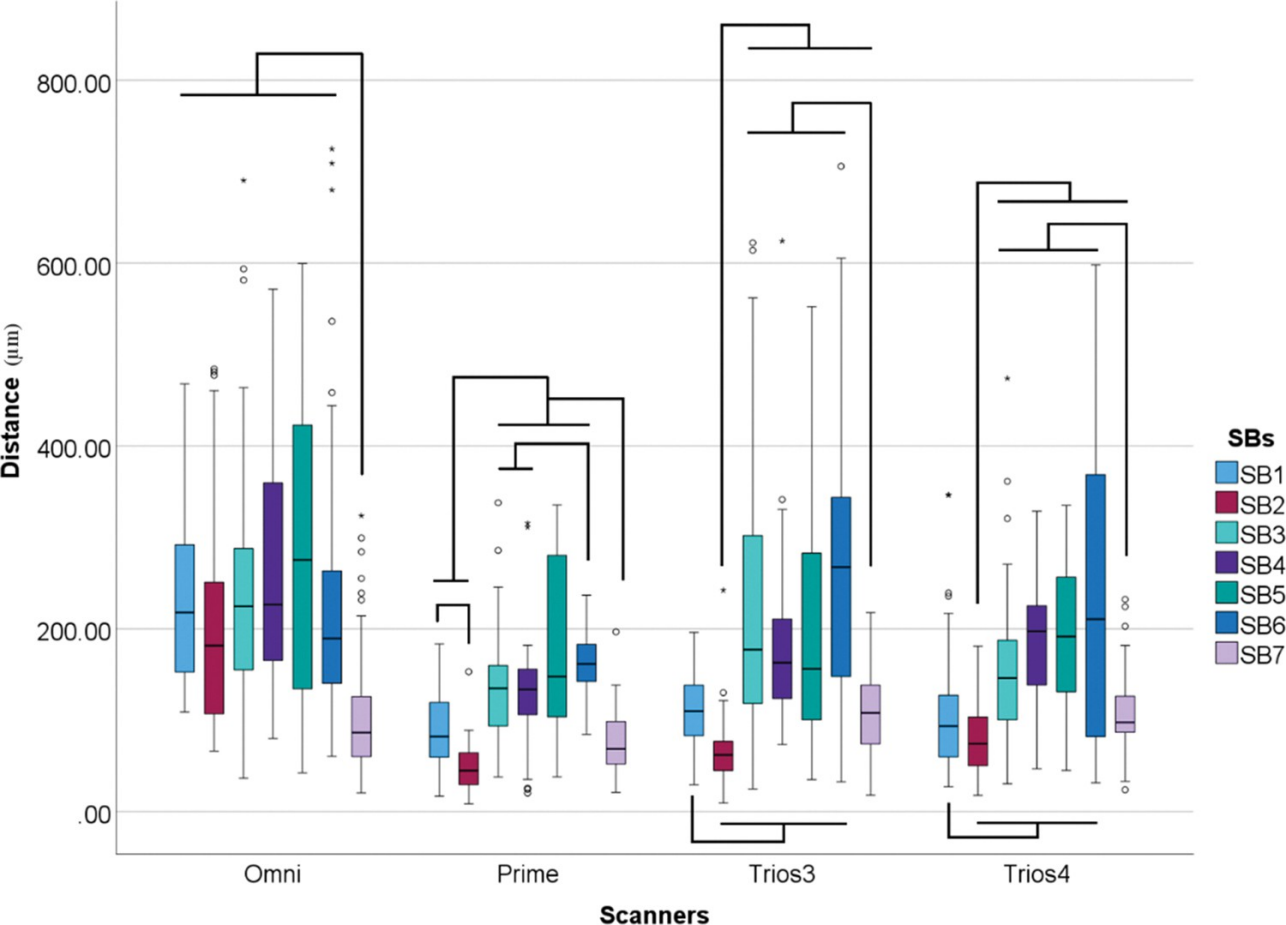
Scanbodies data (μm) within each scanner. Boxplot graphic showing differences between scanbodies inside each scanner for 3D deviation. Lines connecting groups indicate significant differences between groups after Bonferroni correction (P < .005).

Considering all SBs within each Scanner (Table 5 and Fig 10) it was found that the type of SB influenced the 3D deviation for all scanners (p<0.001).

**Table 5:** Descriptive analyses of distance (μm) variation among tested groups in median values.

	*SB1*	*SB2*	*SB3*	*SB4*	*SB5*	*SB6*	*SB7*
*Omni*	217.88	181.59	224.56	226.56	275.21	189.44	86.50
*Prime*	82.14	44.89	134.98	133.69	147.80	161.65	68.68
*Trios 3*	109.82	62.18	177.22	162.99	156.12	267.55	108.09
*Trios 4*	98.66	74.35	146.05	197.20	191.60	210.46	97.67

**Fig 11:**
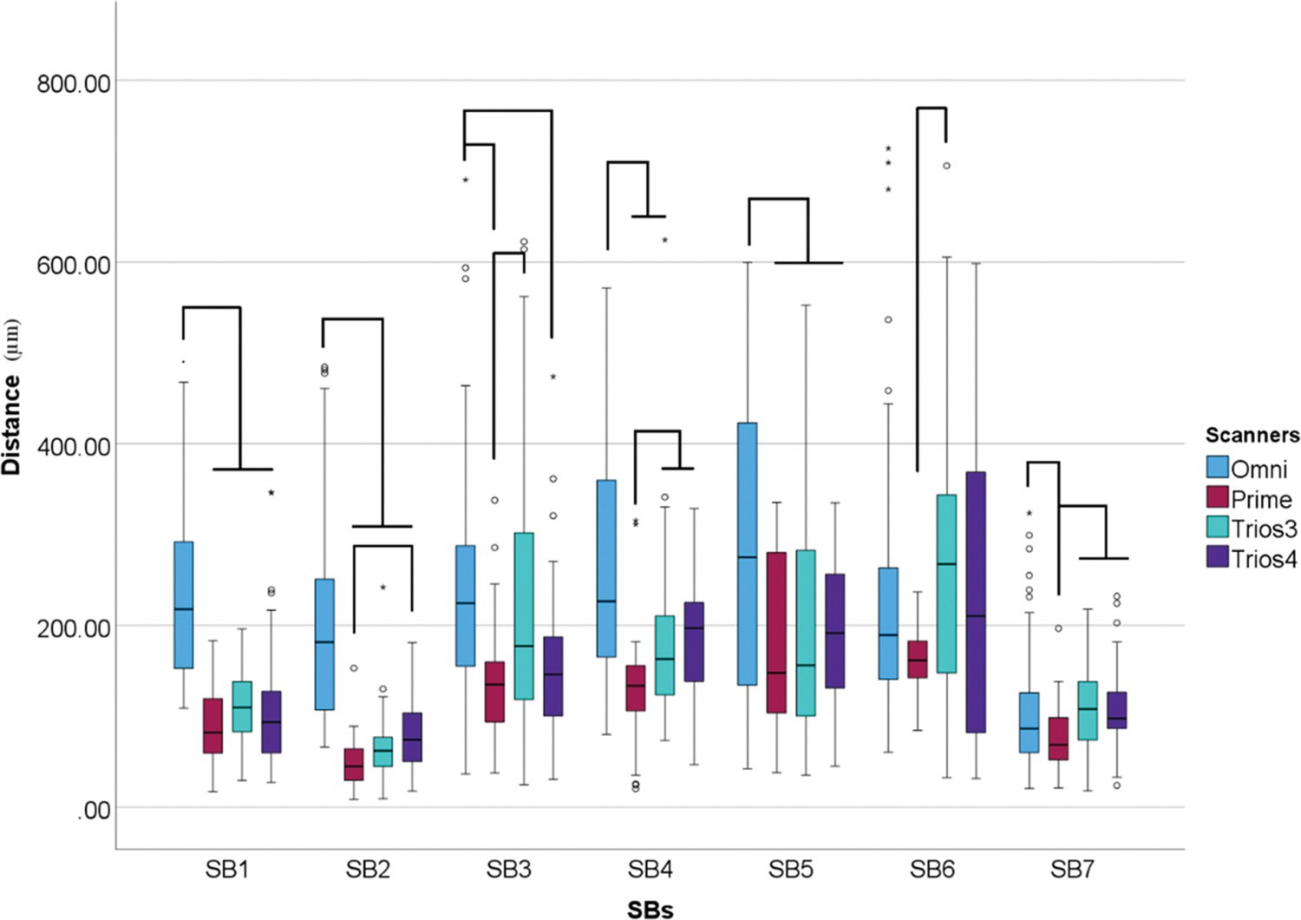
Scanner data (μm) within each scanbodies. Boxplot graphic showing differences between scanners inside each scanbody for 3D deviation. Lines connecting groups indicate significant differences between groups after Bonferroni correction (P < .005).

Considering all scanners within each SBs it was found that the scanners used influenced the 3D deviation for all SBs (p<0.001) (Fig 11).

**Fig 12:**
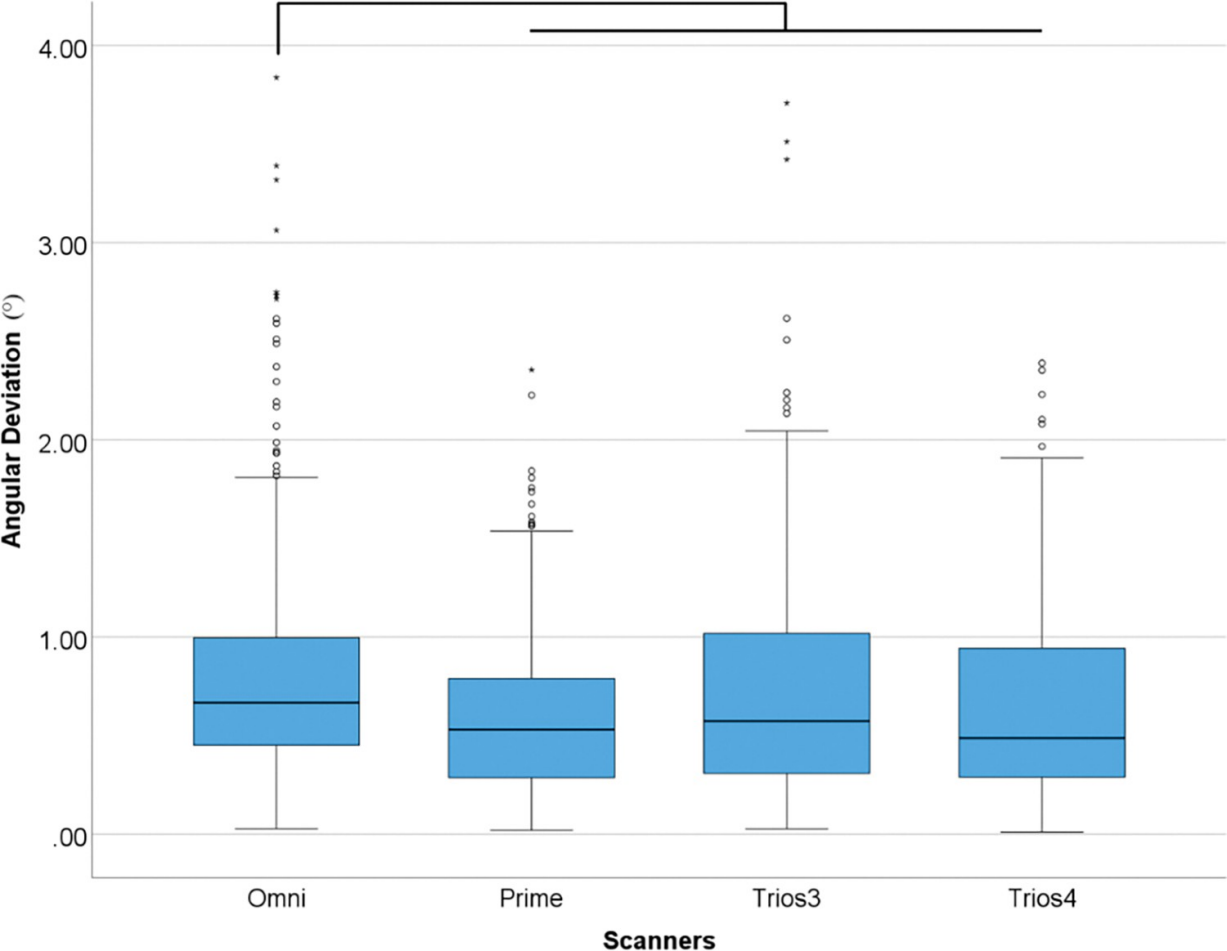
Overall scanners data in angular (°) deviation. Boxplot graphic showing differences between scanners for angular deviation. Lines connecting groups indicate significant differences between groups after Bonferroni correction (P < .005).

The tested scanners showed overall differences in angular deviation (p<0.001) (Table 6 and Fig 12).

**Table 6:** Descriptive analyses of overall differences among scanners for angular (°) deviation in medians.

	*Angular (°)*
*Omni*	0.67
*Prime*	0.53
*Trios 3*	0.57
*Trios 4*	0.49

**Fig 13:**
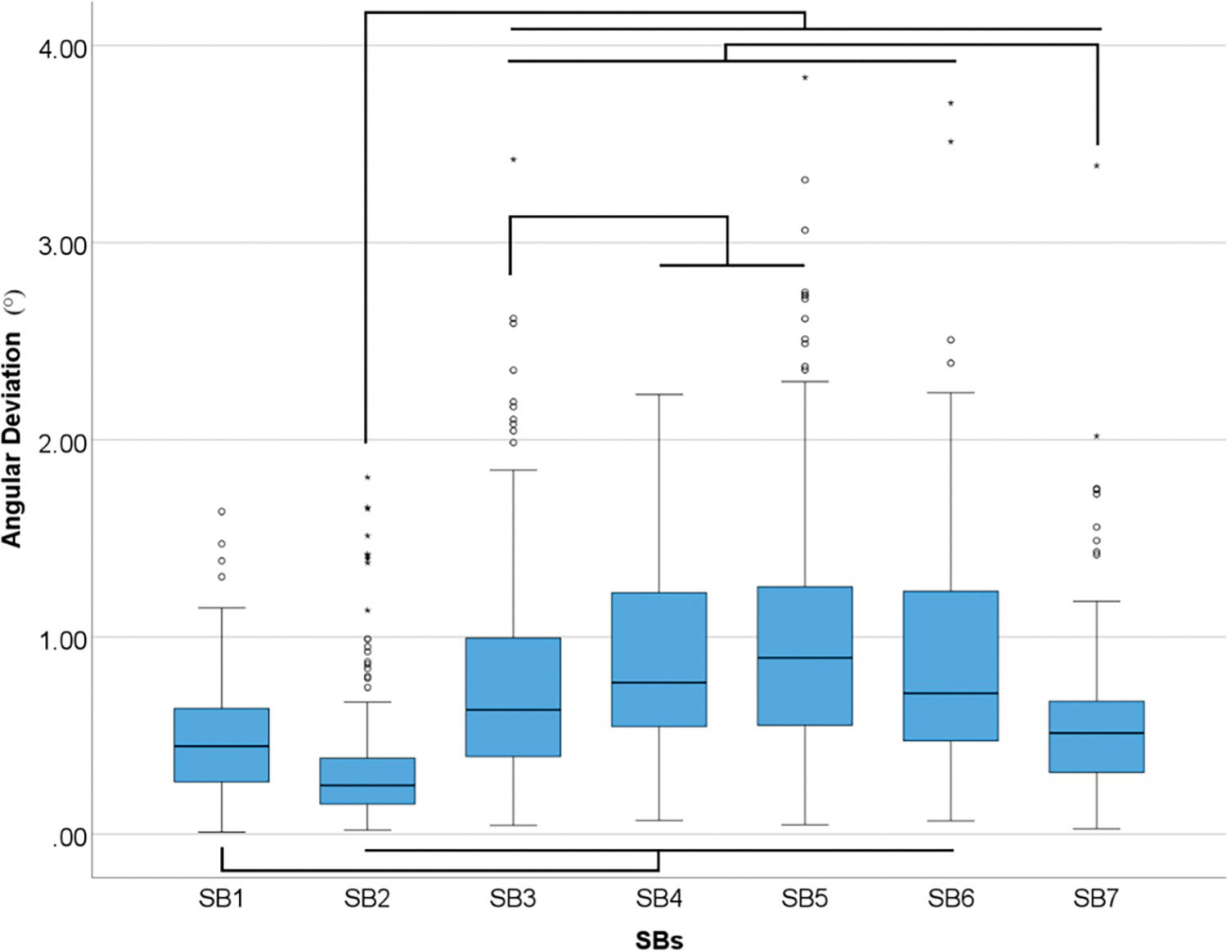
Overall scanbodies data in angular (°) deviation. Boxplot graphic showing differences between scanbodies for angular deviation. Lines connecting groups indicate significant differences between groups after Bonferroni correction (P < .005).

Overall differences were found in angular deviation between SBs (p<0.001) (Table 7 and Fig 13).

**Table 7:** Descriptive analyses of overall differences among SBs for angular (°) deviation in median.

	*Angular (°)*
*SB1*	0.45
*SB2*	0.25
*SB3*	0.63
*SB4*	0.77
*SB5*	0.89
*SB6*	0.71
*SB7*	0.51

**Fig 14:**
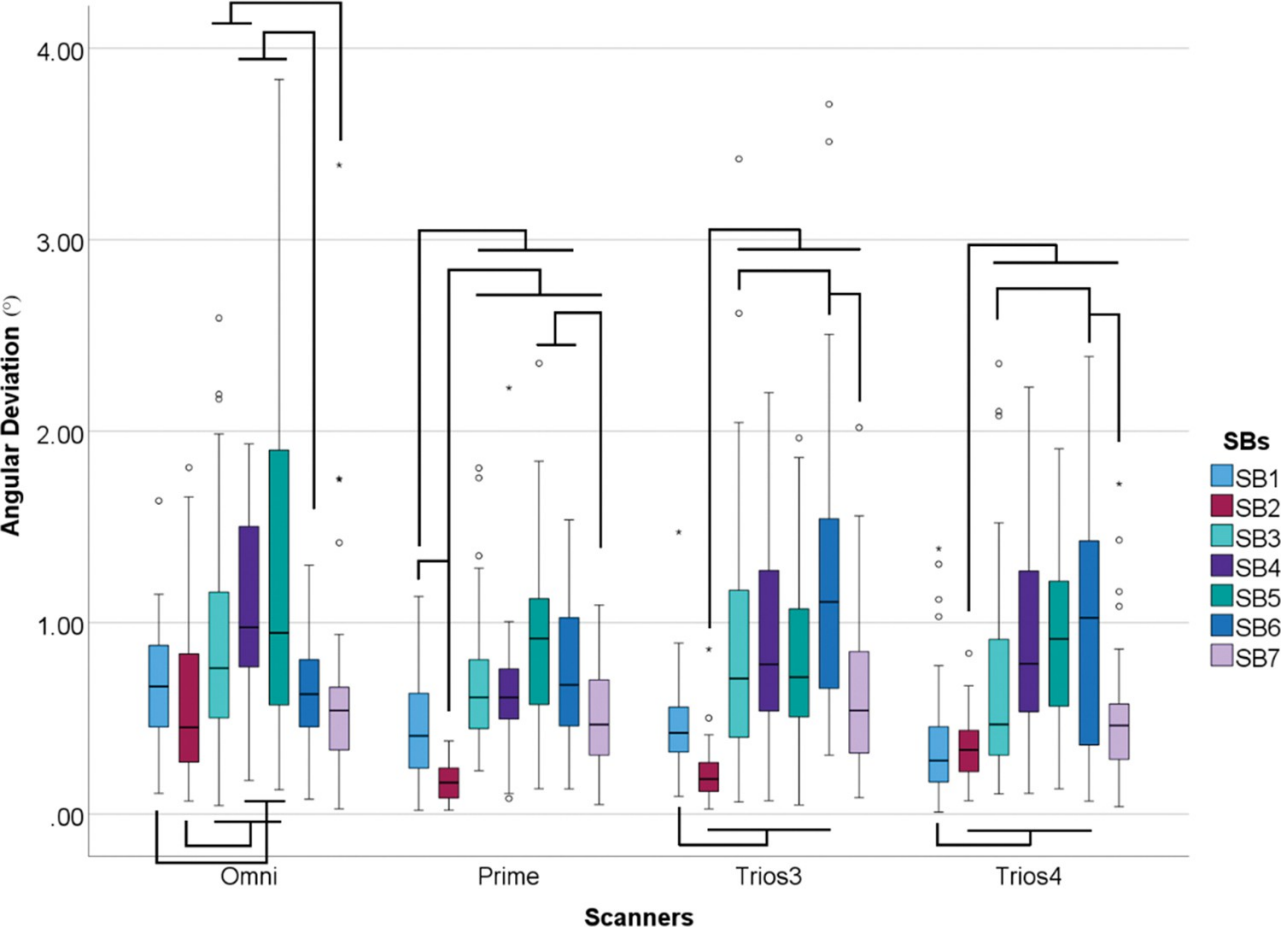
Scanbodies data (°) within each scanner. Boxplot graphic showing differences between scanbodies inside each scanner for angular deviation. Lines connecting groups indicate significant differences between groups after Bonferroni correction (P < .005).

Differences were found in angular deviation for SBs within scanners (p<0.001) (Table 8 and Fig 14).

**Table 8:** Descriptive analyses of angular (°) deviation in median values.

	*SB1*	*SB2*	*SB3*	*SB4*	*SB5*	*SB6*	*SB7*
*Omni*	0.67	0.45	0.76	0.98	0.95	0.63	0.54
*Prime*	0.41	0.16	0.61	0.61	0.92	0.67	0.47
*Trios 3*	0.42	0.18	0.71	0.78	0.72	1.11	0.54
*Trios 4*	0.28	0.34	0.47	0.79	0.92	1.03	0.46

**Fig 15:**
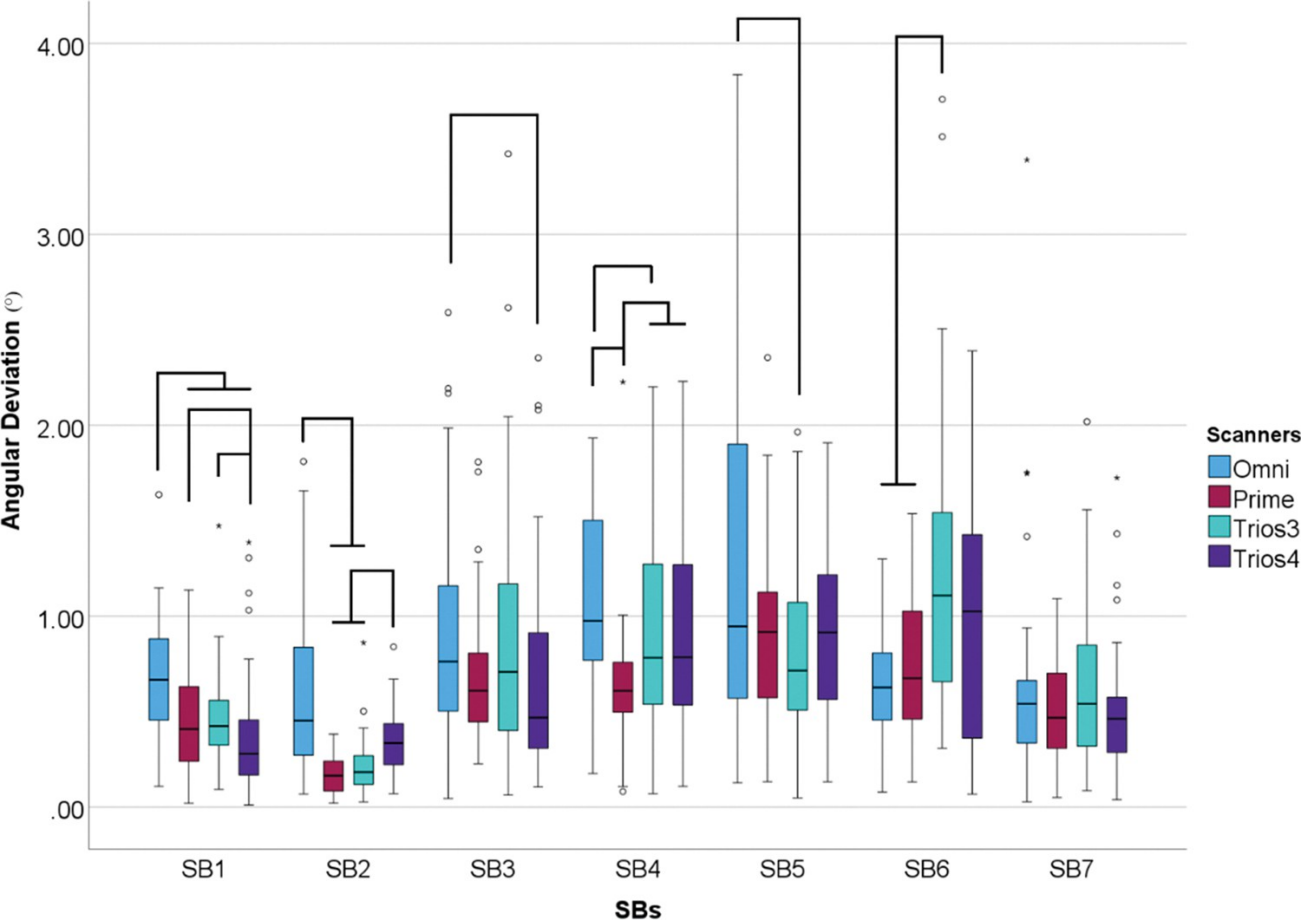
Scanner data (°) within each scanbodies. Boxplot graphic showing differences between scanners inside each scanbodies for angular deviation. Lines connecting groups indicate significant differences between groups after Bonferroni correction (P < .005).

Differences were found between all SBs (p<0.001) but SB7 had no differences between scanners (p = 0.222) (Fig 15)

**Fig 16:**
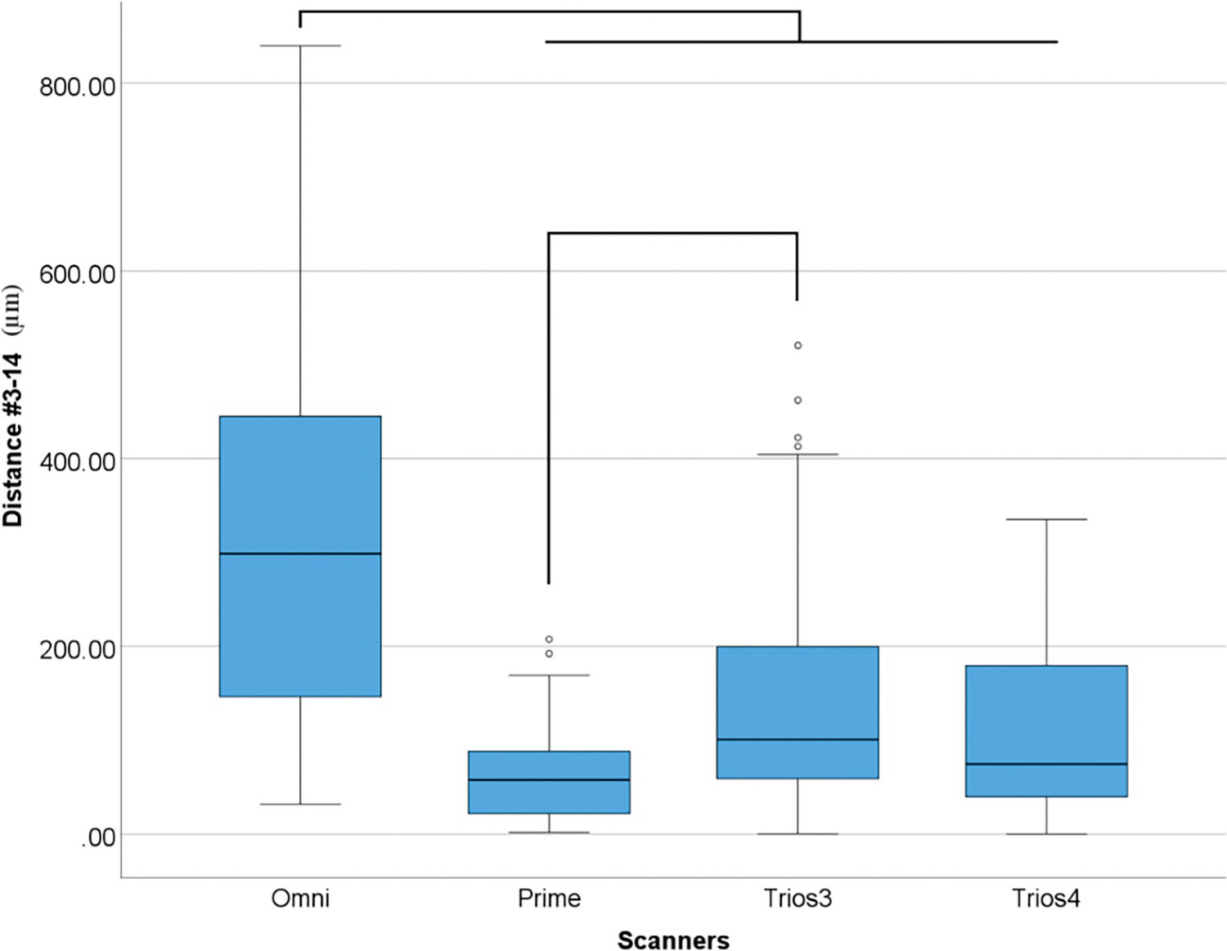
Overall scanners data (μm) in linear distance deviation between #3 and #14. Boxplot graphic showing differences between scanners for linear distance deviation between implants #3 and #14. Lines connecting groups indicate significant differences between groups after Bonferroni correction (P < .005).

Regarding the distance deviation between implants #3 to #14, differences were found in overall data (p<0.001) (Table 9 and Fig 16).

**Table 9:** Descriptive analyses of overall differences among scanners for linear distance (μm) deviation between #3–14 in medians.

	*Distance (μm)*
*Omni*	298.83
*Prime*	57.85
*Trios 3*	100.82
*Trios 4*	74.71

**Fig 17:**
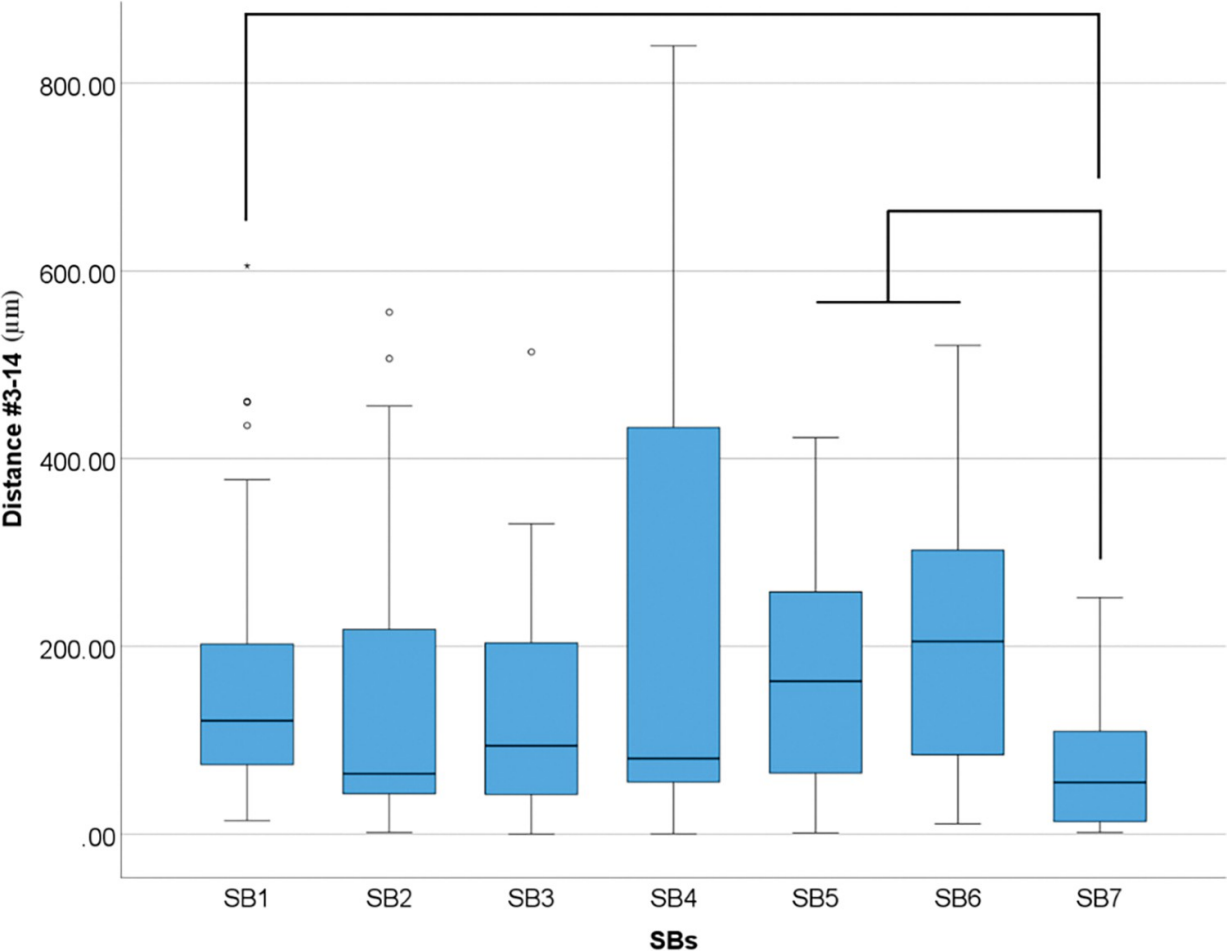
Overall scanbodies data (μm) in linear distance deviation between implants #3 and #14. Boxplot graphic showing differences between scanbodies for linear distance deviation. Lines connecting groups indicate significant differences between groups after Bonferroni correction (P < .005).

Differences were also found in SBs for linear distance deviation between implants #3 and #14 (p<0.001) (Table 10 and Fig 17).

**Table 10:** Descriptive analyses of overall differences among SBs for linear distance deviation (μm) between #3–14 in medians.

	Distance (μm)
SB1	120.95
SB2	64.44
SB3	94.17
SB4	80.65
SB5	162.86
SB6	205.27
SB7	55.17

**Fig 18:**
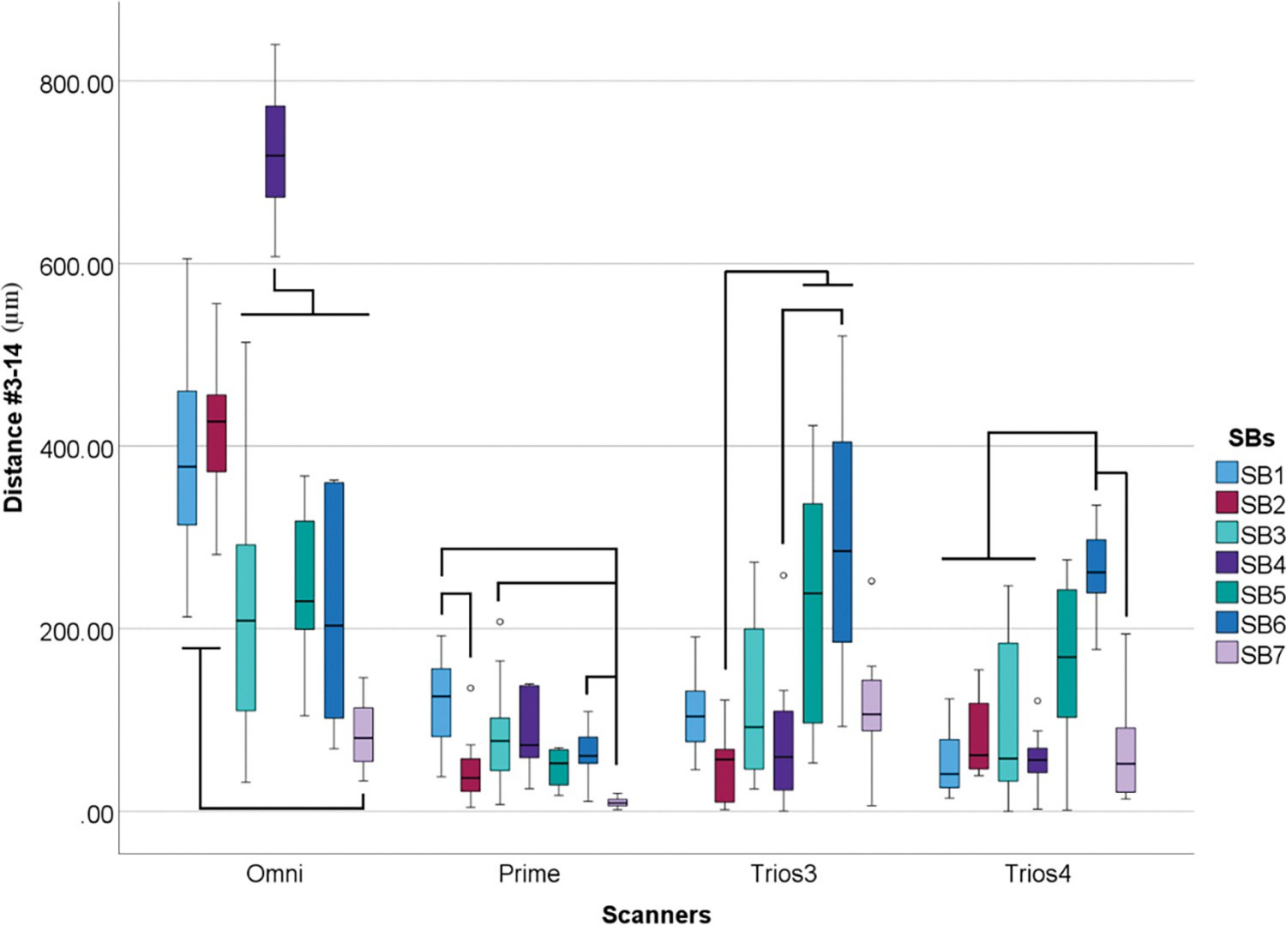
Scanbodies data (μm) within each scanner. Boxplot graphic showing differences between scanbodies inside each scanner for linear distance deviation between implants #3 and #14. Lines connecting groups indicate significant differences between groups after Bonferroni correction (P < .005).

Considering the linear distance deviation between implants #3 and #14 inside scanners and SBs, differences were also found (p<0.001) (Table 11 and Fig 18).

**Table 11:** Descriptive analyses of linear distance deviation (μm) between #3-#14 in median values.

	SB1	SB2	SB3	SB4	SB5	SB6	SB7
Omni	377.42	426.87	208.77	718.16	230.17	203.49	49.8
Prime	125.97	36.59	77.11	72.61	55.77	60.95	23.79
Trios 3	104.02	56.83	92.37	59.55	238.82	285.05	106.32
Trios 4	40.84	61.480	57.92	56.22	168.91	261.81	52.27

**Fig 19:**
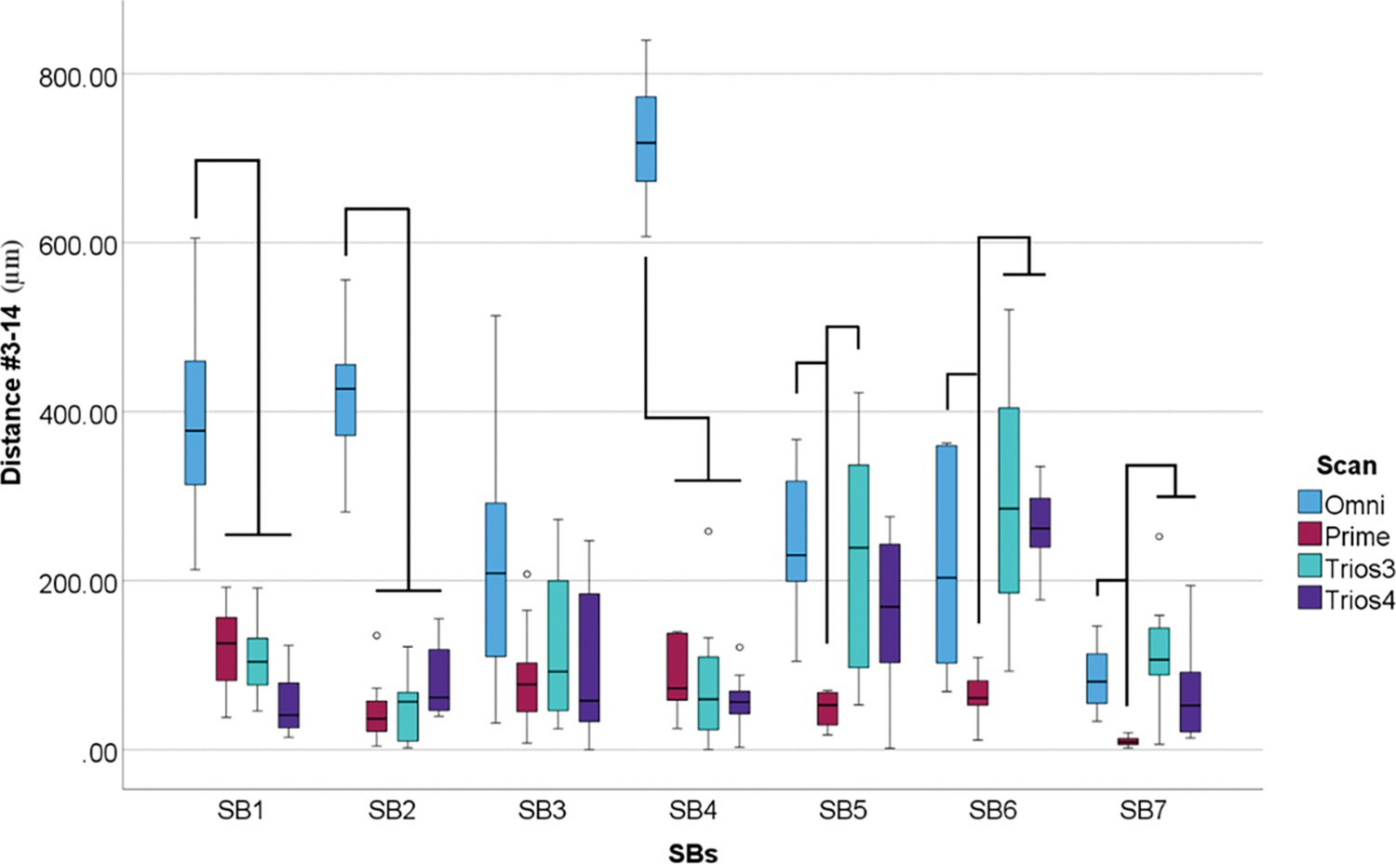
Scanner data (μm) within each scanbodies. Boxplot graphic showing differences between scanners inside each scanbodies for linear distance deviation between implants #3 and #14. Lines connecting groups indicate significant differences between groups after Bonferroni correction (P < .005).

Differences were also found in scanners within SBs for linear distance deviation between implants #3 and #14 (p<0.001) (Fig 19).

## Discussion

The present study investigated the influence of seven SB designs and four intraoral scanners (IOS) on the trueness of complete arch implant digital impressions. The null hypothesis was rejected, indicating a significant difference in the overall trueness of digital impressions for complete-arch implant-supported restorations when different SB designs and IOS systems were used.

Accurate digital impressions for implant-supported restorations are critical for achieving optimal clinical outcomes. Currently, several commercially available intraoral scanning devices operate on different principles and exhibit varying levels of accuracy. In this study, four IOS and one bench scanner were used: Primescan, Omnicam, Trios 3, Trios 4, and D2000 which have been used in several other studies [8, 14, 16, 17, 19–23, 26, 29, 30, 34–41]. The present study found that digital complete-arch implant impressions obtained with Primescan were significantly more accurate considering the 3D deviation and linear distance deviation between implants #3 and #14, compared to the other IOS groups. Meanwhile, the Omnicam group showed the worst overall values in the same analysis. Trios 4 group tended to be placed between or sometimes similar to Primescan and Trios 3.

The Primescan intraoral scanner utilizes confocal microscopy to capture high-resolution data, which is then processed into a 3D digital model of the patient’s mouth. Its design prioritizes speed and accuracy, enabling the capture of full arches and deep preparation margins up to a depth of 20mm. Previous studies [19, 29, 30, 34, 38, 39, 41] have assessed the influence of different scanners on complete-arch digital implant scans showing that Primescan exhibits the lowest discrepancies in terms of trueness and precision. However, in other ones, it does not show a statistically significant difference when compared to the Trios 3 and Carestream CS-3700 [19].

The 3Shape Trios 3 is a popular device used in dental research studies because it provides high-quality digital impressions and has been extensively validated for accuracy and reliability. The Trios 3 scanner uses a powerful multi-line blue LED technology to capture high-resolution images, has a high scanning speed, and can capture up to 3,000 2D images per second, making it an efficient scanner for clinical use. The Trios 4 scanner is an upgraded version of the Trios 3, offering enhanced scanning capabilities. It uses a four-camera setup and, in combination with artificial intelligence, can capture images at an even higher resolution than Trios 3. However, in this study, both Trios 3 and Trios 4 did not exhibit any statistically significant differences in the measurements evaluated.

The CEREC Omnicam is an intraoral scanner that uses "confocal microscopy" technology to capture digital impressions of teeth and oral structures. The scanner also uses "continuous scanning" technology, which means it captures images in real-time as the operator moves the scanner around the mouth, allowing for a more efficient and comfortable scanning experience for the patient. However, in this study, Omnican showed the highest median values for all measurements, which may be acceptable, as its primary indication is for small regions or half-arches.

The 3Shape software (D2000, Trios 3, and Trios 4) do not offer the option to select the quality of.STL files, exporting a standard quality file, while Primescan allows to select the desired quality. In this study, the highest quality was chosen to export the files, and this could have interfered with the lower deviation values the Primescan group has shown.

The precise location of the scan bodies may distort their actual positions, which could impact the accuracy of the scans. While there is insufficient scientific evidence to support the effectiveness of complete-arch implant digital scans, additional techniques utilized during intraoral scanning may improve accuracy [11]. However, such techniques may be more complex and require additional expertise. This study did not find relevant data regarding the different sites of the implants. The accuracy of digital impressions can be influenced by various factors such as the type of impression material, implant angulation, and the operator’s experience [3, 7, 10, 17, 31]. In this study, an expert operator was selected to conduct the scanning from the beginning to the end. Despite the recommended scanning strategies for dentate arches provided by intraoral scanner systems, these may not be optimal for complete-arch implant impressions [21, 40]. The design of the IOS tip and the SBs, depending on their height, might not allow the scan to be initiated from the occlusal surface. Therefore, adapted scanning strategies were implemented to enable correct 3D image acquisition. In this study, the scanning path was adapted according to the type of scanner tip and SB height. The scan started at the occlusal surface of implant #3 and subsequently rotated the scanner tip to the buccal surface at a 45-degree angle, ending at the position of SB #14. Following the lingual side and additional scan around each SB.

The trueness of digital impressions varied significantly based on the linear distance deviation between implants #3 (at the beginning of the scanning path) and #14 (at the end of the scanning path). Presumably, this is due to the accumulation of errors resulting from the superimposition of scan images, particularly in longer scanning paths. A study has shown that IOS systems tend to be less accurate in posterior areas due to an expansion in the mesh on posterior areas [42].

The SB plays a critical role in the digital workflow for transferring the position of dental implants to computer-aided design (CAD) software for the design of the prosthesis. They are typically made of materials such as titanium, polyetheretherketone (PEEK), or a mix of them, and they come in different sizes and designs to match different implant systems. In this study, multi-unit abutments were used to allow different implant systems to be used in the same model. By comparing the trueness of different IOS on different SB designs, the study aimed to provide insights into how the design of these components can affect the trueness of digital impressions and subsequent prosthesis design. It could not be concluded which combination between scanner and scanbody showed the best results since the data were not parametric and comparison crossing groups would not be allowed. Considering that in private practice, the operator mainly chooses one scanner for their dental office, this should be considered a considerable limitation. Instead of that, the shape of the SB and the behavior of the IOS system they choose should be considered. Knowing how to determine which SB design works better with the acquired IOS system can lead to more successful outcomes. In this study, SB2 and SB7 showed the lowest values in all tested parameters. Furthermore, SB7 did not show any differences between scanners in angular deviation, and SB2 showed the lowest angular deviation values in Primescan, Trios 3, and Trios 4 (Fig 15). The feature they have in common, are the cylindric shape, the reduced high, and just one beveled site compared to the other groups. This led to believe that the size and the shape might have an influence on trueness, which corroborate with other previous studies [7, 16–18, 20, 25–29].

Another crucial step in the digital workflow is the alignment between the SB scan and the SB reference file mesh. In this study, the alignment between the digital scans and the SBs files was done on Blender software (Blender Foundation), which is an open-source, using a tool called Object align/ICP align. In this study, the tool used to align was standardized to avoid one more variable. Studies on ISBs are typically focused on factors such as the best fitting between mesh and mesh file, implant connection, and angulation. The congruence between the SB mesh files and the implant library can also impact the determination of implant position errors in CAD software [22, 25, 30]. More studies are needed to determine the behavior of the dental software in the alignment of SBs.

The 3D-printed SBs created specifically for this study had the advantage of being shorter, making the scanning procedure easier. However, their inferior performance in the study may be attributed to the limitations of the fabrication process [36]. Nowadays, commercially available SB is similar to the proposed design of group SB6, where each sale kit comes with a QR code with the respective STL file of that specific SB. It is believed that these can be a potential limitation of using 3D-printed SBs. Added to that, their accuracy may not be good enough, which could lead to distortions in the alignment of the software when compared with the STL mesh. Using custom-printed SBs in this study introduces potential errors due to variations between different SBs, making it difficult to solely test the influence of SB shape. Therefore, it is important to verify the accuracy of custom-printed SBs before using them in research studies.

Regarding the limitations of this study, the in vitro design did not fully replicate clinical conditions, such as mucosal mobility and the presence of saliva, which may affect the accuracy of the system, particularly for intraoral scanners. Another inherent limitation when comparing IOSs is the software version utilized. Companies are always releasing new versions of the software, which can lead to better accuracy. The 3Shape files (D2000, Trios 3, and Trios 4) did not offer the option to select the quality of.STL files. One of the main limitations is also the use of custom 3D-printed SBs, which may not have the same level of accuracy as commercially available SBs. The 3D-printed SBs did not have a smooth surface finish, which could also interfere with the alignment with the STL mesh in the software. This study focused on trueness, but because repeated measurements were taken for each group (n = 10), precision can also be inferred by the interquartile data shown in boxplot figures. Finally, the study did not evaluate the fit of the final prostheses, which is an important factor in determining the clinical significance of the accuracy of digital impressions. Thus, more in vitro studies and clinical trials are needed to access the adaptation, complications, and outcomes of final restorations.

## Conclusions

Within the limitations of this in vitro study, the following conclusions were drawn:

1. The 3D deviation, angular deviation, and linear distance between implants #3 and #14 were influenced by both SBs and IOSs tested.
2. The Primescan system demonstrated the highest trueness in 3D deviations among all scanners.
3. SB2 and SB7 had the highest trueness result in general.
4. The new SBs designs had the highest deviation values compared to the others.

## Supporting information

S1 File Script used to access the distance between #3 and #14 implants. (PY)

S2 File Script used to access the deviation between implants. (PY)

S3 File Report generated by SPSS software. (PDF)
