# Supplementary material for: Influence of scanbody design and intraoral scanner on the trueness of complete arch implant digital impressions: An in vitro study
Source: PLoS One. 2023 Dec 19;18(12):e0295790. doi: 10.1371/journal.pone.0295790 (PMC10729975; doi:10.1371/journal.pone.0295790)
Supplement: S3 File — (PDF) [file pone.0295790.s003.pdf]

Nonparametric Tests

Notes

Output Created		30-MAY-2023 09:09:44
Comments		
Input	Active Dataset	DataSet2
	Filter	<none>
	Weight	<none>
	Split File	<none>
	N of Rows in Working Data File	280
Syntax		NPTESTS /INDEPENDENT TEST (Distance) GROUP (SBs) KRUSKAL_WALLIS (COMPARE=PAIRWISE) /MISSING SCOPE=ANALYSIS USERMISSING=EXCLUDE /CRITERIA ALPHA=0.05 CILEVEL=95.
Resources	Processor Time	00:00:00.83
	Elapsed Time	00:00:00.74

[DataSet2]

Hypothesis Test Summary

	Null Hypothesis	Test	Sig.[a,b]
1	The distribution of Distance is the same across categories of SBs.	Independent-Samples Kruskal-Wallis Test	<.001

Hypothesis Test Summary

	Decision
1	Reject the null hypothesis.

a. The significance level is .050.

b. Asymptotic significance is displayed.

Independent-Samples Kruskal-Wallis Test

Distance across SBs

Independent-Samples Kruskal-Wallis Test Summary

Total N	280
Test Statistic	31.904[a]
Degree Of Freedom	6
Asymptotic Sig.(2-sided test)	<.001

a. The test statistic is adjusted for ties.

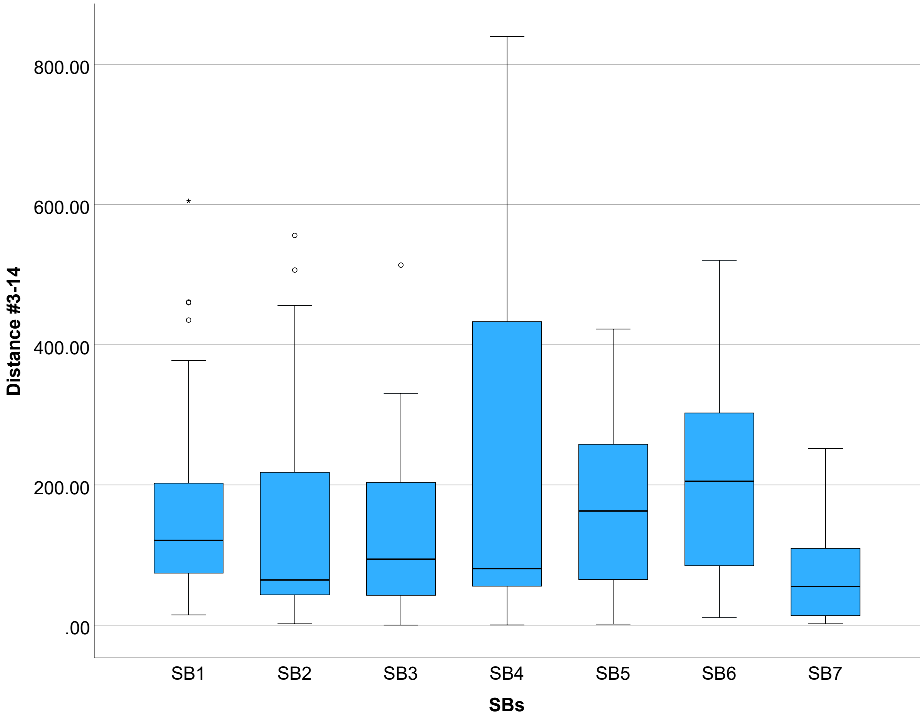

Pairwise Comparisons of SBs

Sample 1-Sample 2	Test Statistic	Std. Error	Std. Test Statistic	Sig.	Adj. Sig.[a]
SB7-SB2	38.700	18.106	2.137	.033	.684
SB7-SB3	42.800	18.106	2.364	.018	.380
SB7-SB4	54.675	18.106	3.020	.003	.053
SB7-SB1	65.950	18.106	3.642	<.001	.006
SB7-SB5	71.125	18.106	3.928	<.001	.002
SB7-SB6	93.200	18.106	5.147	<.001	.000
SB2-SB3	-4.100	18.106	-.226	.821	1.000
SB2-SB4	-15.975	18.106	-.882	.378	1.000
SB2-SB1	27.250	18.106	1.505	.132	1.000
SB2-SB5	-32.425	18.106	-1.791	.073	1.000
SB2-SB6	-54.500	18.106	-3.010	.003	.055
SB3-SB4	-11.875	18.106	-.656	.512	1.000
SB3-SB1	23.150	18.106	1.279	.201	1.000
SB3-SB5	-28.325	18.106	-1.564	.118	1.000
SB3-SB6	-50.400	18.106	-2.784	.005	.113
SB4-SB1	11.275	18.106	.623	.533	1.000
SB4-SB5	-16.450	18.106	-.909	.364	1.000
SB4-SB6	-38.525	18.106	-2.128	.033	.701
SB1-SB5	-5.175	18.106	-.286	.775	1.000
SB1-SB6	-27.250	18.106	-1.505	.132	1.000
SB5-SB6	-22.075	18.106	-1.219	.223	1.000

Each row tests the null hypothesis that the Sample 1 and Sample 2 distributions are the same.
Asymptotic significances (2-sided tests) are displayed. The significance level is .050.

a. Significance values have been adjusted by the Bonferroni correction for multiple tests.

Pairwise Comparisons of SBs

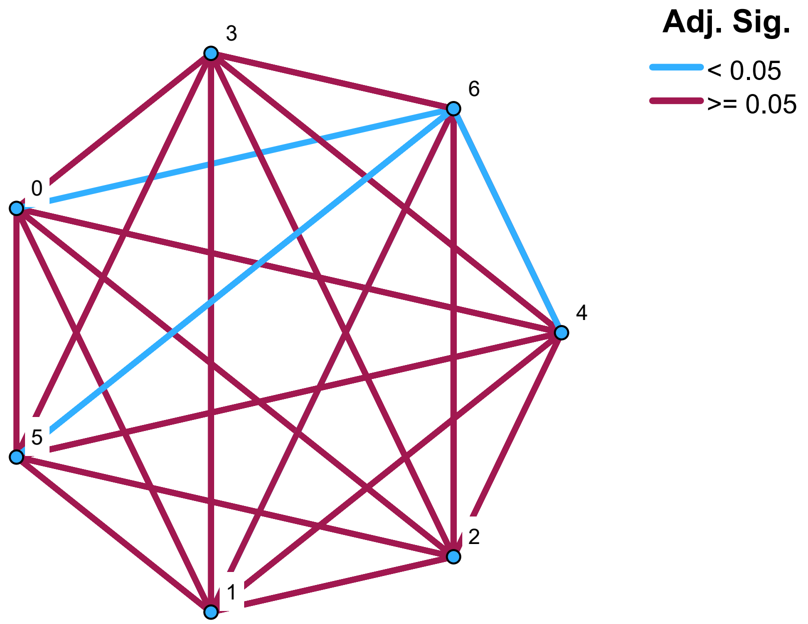

Each node shows the sample average rank of SBs.

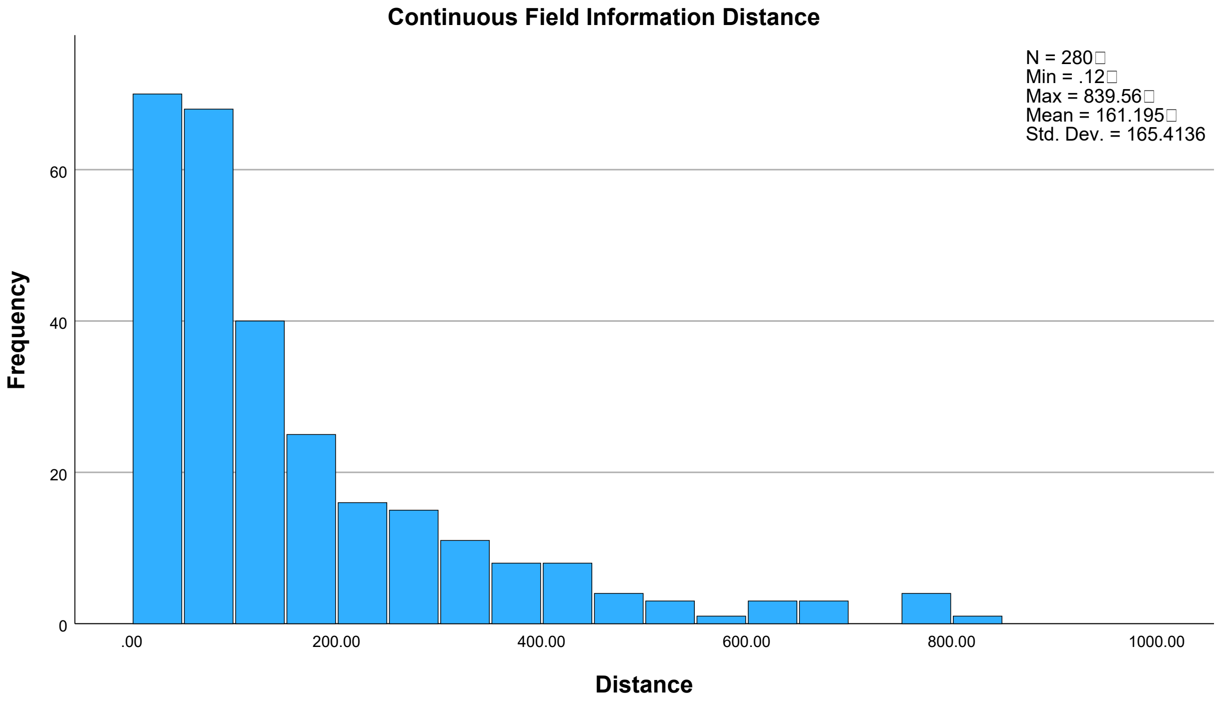

Continuous Field Information Distance
N = 280
Min = .12
Max = 839.56
Mean = 161.195
Std. Dev. = 165.4136
Frequency
0
20
40
60
.00
200.00
400.00
600.00
800.00
1000.00
Distance

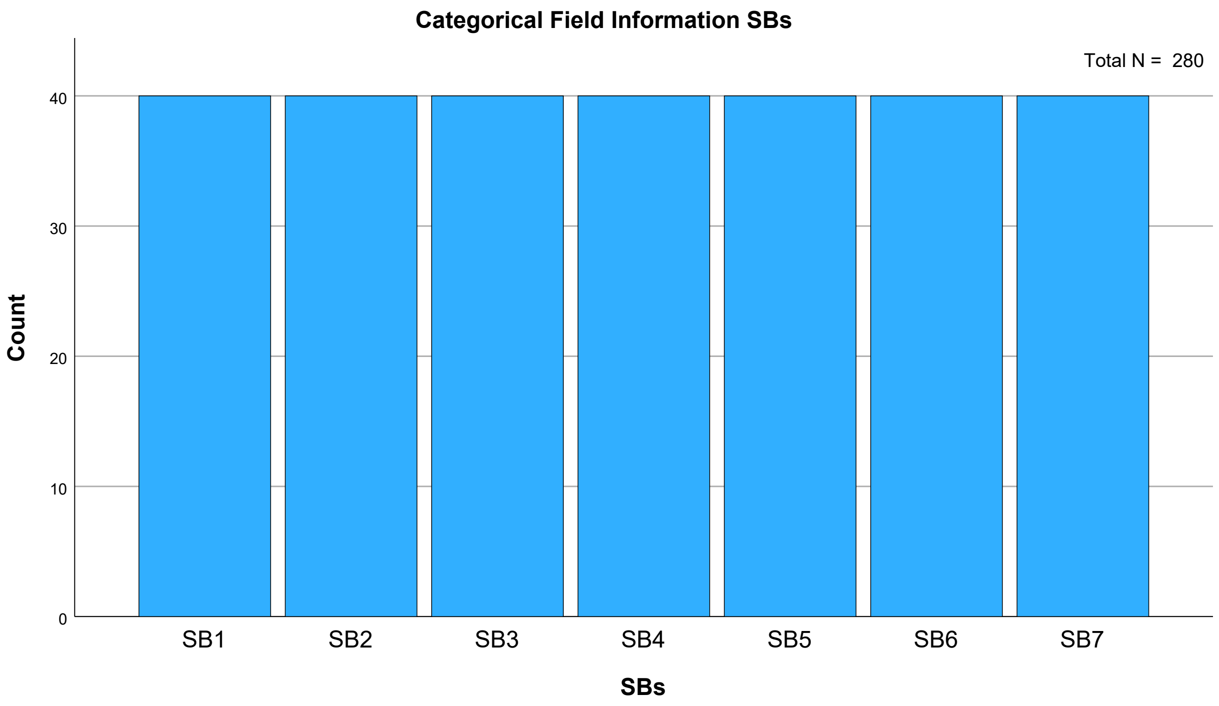

Categorical Field Information SBs
Total N = 280
Count
0
10
20
30
40
SB1
SB2
SB3
SB4
SB5
SB6
SB7
SBs

Nonparametric Tests

Notes

Output Created		30-MAY-2023 09:17:14
Comments		
Input	Active Dataset	DataSet2
	Filter	<none>
	Weight	<none>
	Split File	<none>
	N of Rows in Working Data File	280
Syntax		NPTESTS /INDEPENDENT TEST (Distance) GROUP (Scan) KRUSKAL_WALLIS (COMPARE=PAIRWISE) /MISSING SCOPE=ANALYSIS USERMISSING=EXCLUDE /CRITERIA ALPHA=0.05 CILEVEL=95.
Resources	Processor Time	00:00:00.81
	Elapsed Time	00:00:00.75

Hypothesis Test Summary

	Null Hypothesis	Test	Sig.[a,b]
1	The distribution of Distance is the same across categories of Scan.	Independent-Samples Kruskal-Wallis Test	<.001

Hypothesis Test Summary

	Decision
1	Reject the null hypothesis.

a. The significance level is .050.

b. Asymptotic significance is displayed.

Independent-Samples Kruskal-Wallis Test

Distance across Scan

Independent-Samples Kruskal-Wallis Test Summary

Total N	280
Test Statistic	85.618[a]
Degree Of Freedom	3
Asymptotic Sig.(2-sided test)	<.001

a. The test statistic is adjusted for ties.

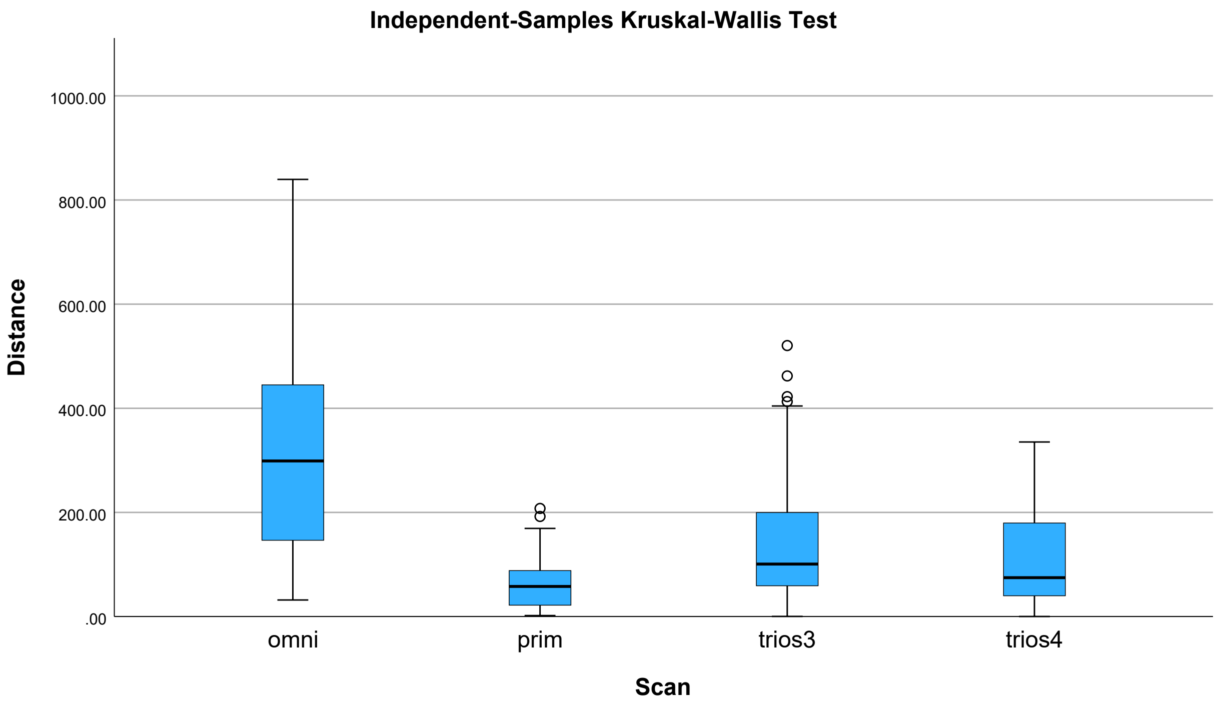

Pairwise Comparisons of Scan

Sample 1-Sample 2	Test Statistic	Std. Error	Std. Test Statistic	Sig.	Adj. Sig.[a]
prim-trios4	-32.800	13.687	-2.396	.017	.099
prim-trios3	-52.871	13.687	-3.863	<.001	.001
prim-omni	122.329	13.687	8.938	<.001	.000
trios4-trios3	20.071	13.687	1.466	.143	.855
trios4-omni	89.529	13.687	6.541	<.001	.000
trios3-omni	69.457	13.687	5.075	<.001	.000

Each row tests the null hypothesis that the Sample 1 and Sample 2 distributions are the same.
Asymptotic significances (2-sided tests) are displayed. The significance level is .050.

a. Significance values have been adjusted by the Bonferroni correction for multiple tests.

Pairwise Comparisons of Scan

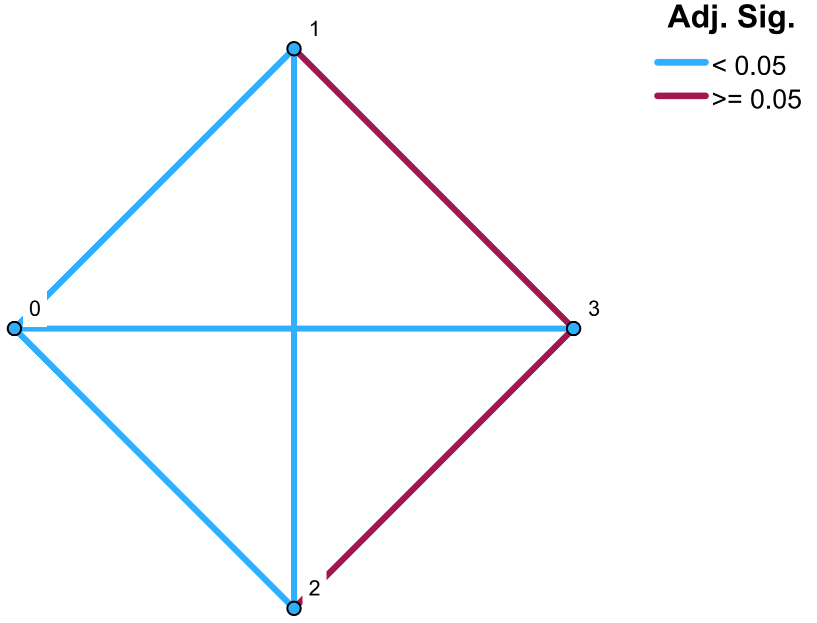

Each node shows the sample average rank of Scan.

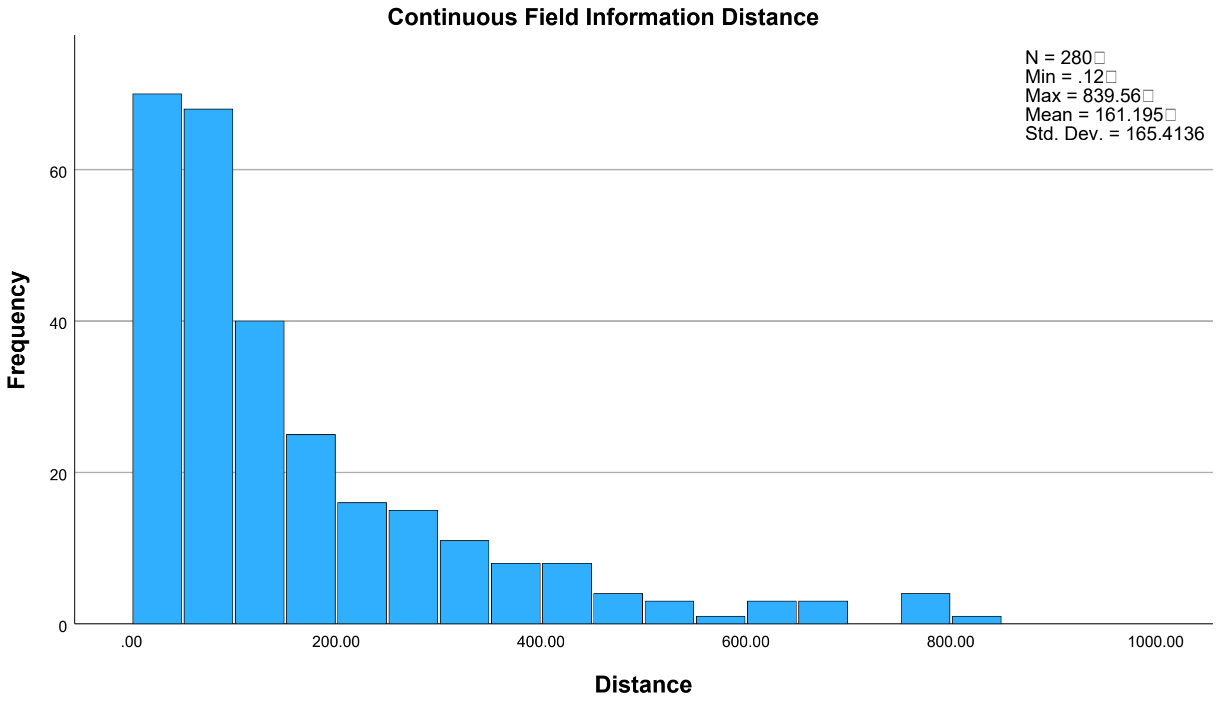
Continuous Field Information Distance
N = 280
Min = .12
Max = 839.56
Mean = 161.195
Std. Dev. = 165.4136
Frequency
0
20
40
60
.00
200.00
400.00
600.00
800.00
1000.00
Distance

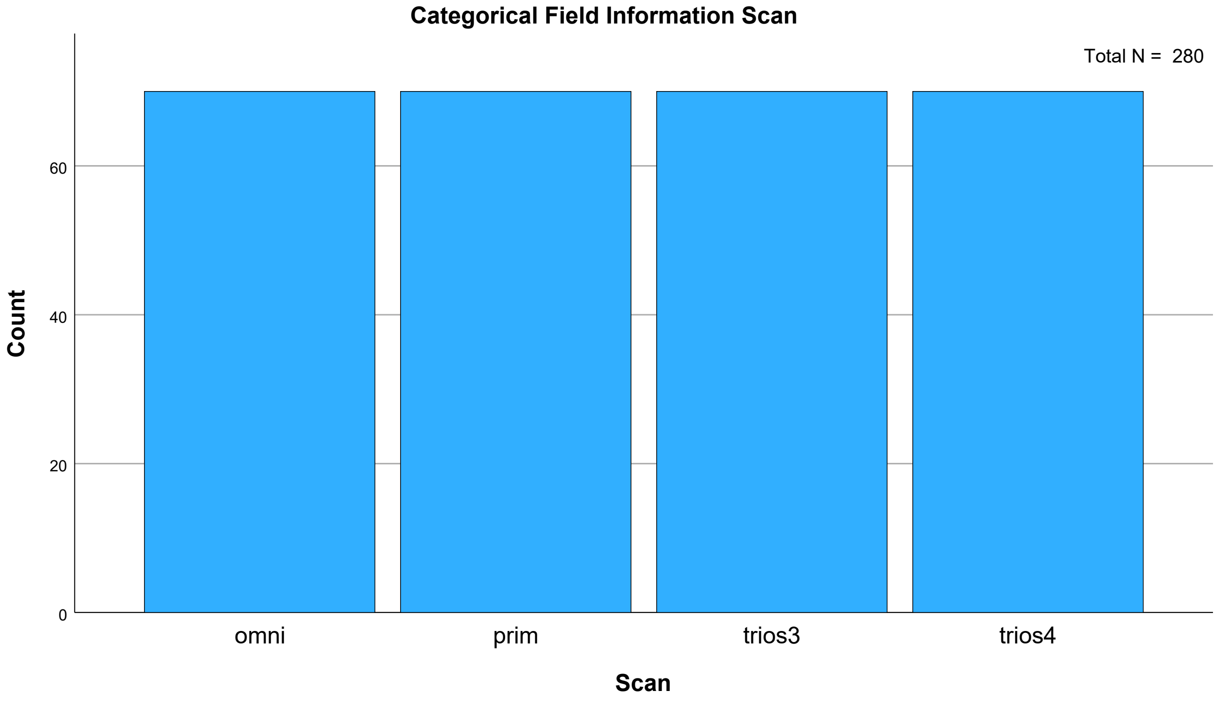
Categorical Field Information Scan
Total N = 280
Count
0
20
40
60
omni
prim
trios3
trios4
Scan

Nonparametric Tests

Notes

Output Created		30-MAY-2023 09:20:43
Comments		
Input	Active Dataset	DataSet2
	Filter	<none>
	Weight	<none>
	Split File	<none>
	N of Rows in Working Data File	280
Syntax		NPTESTS /INDEPENDENT TEST (OmniDis) GROUP (Omni) KRUSKAL_WALLIS (COMPARE=PAIRWISE) /MISSING SCOPE=ANALYSIS USERMISSING=EXCLUDE /CRITERIA ALPHA=0.05 CILEVEL=95.
Resources	Processor Time	00:00:00.70
	Elapsed Time	00:00:00.70

Hypothesis Test Summary

	Null Hypothesis	Test	Sig.[a,b]
1	The distribution of OmniDis is the same across categories of Omni.	Independent-Samples Kruskal-Wallis Test	<.001

Hypothesis Test Summary

	Decision
1	Reject the null hypothesis.

a. The significance level is .050.

b. Asymptotic significance is displayed.

Independent-Samples Kruskal-Wallis Test

OmniDis across Omni

Independent-Samples Kruskal-Wallis Test Summary

Total N	70
Test Statistic	50.844[a]
Degree Of Freedom	6
Asymptotic Sig.(2-sided test)	<.001

a. The test statistic is adjusted for ties.

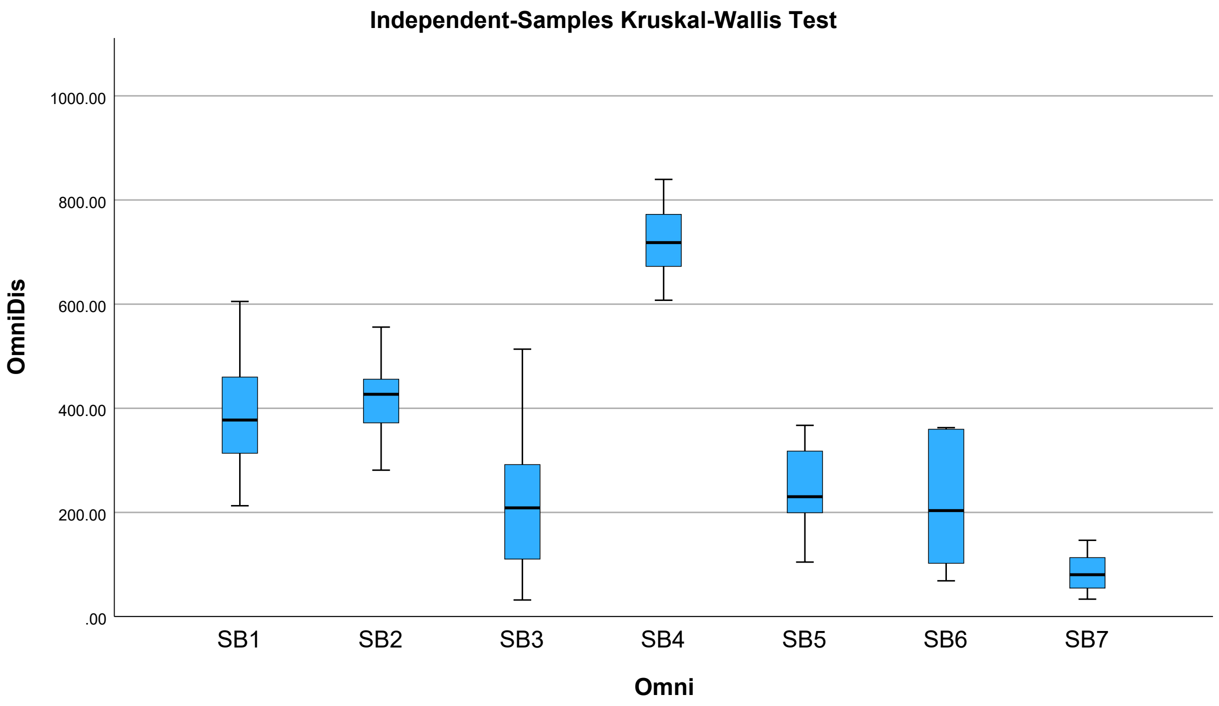

Pairwise Comparisons of Omni

Sample 1-Sample 2	Test Statistic	Std. Error	Std. Test Statistic	Sig.	Adj. Sig.[a]
SB7-SB3	15.300	9.101	1.681	.093	1.000
SB7-SB6	15.700	9.101	1.725	.085	1.000
SB7-SB5	19.400	9.101	2.132	.033	.694
SB7-SB1	35.600	9.101	3.912	<.001	.002
SB7-SB2	39.400	9.101	4.329	<.001	.000
SB7-SB4	55.900	9.101	6.142	<.001	.000
SB3-SB6	-.400	9.101	-.044	.965	1.000
SB3-SB5	-4.100	9.101	-.450	.652	1.000
SB3-SB1	20.300	9.101	2.230	.026	.540
SB3-SB2	24.100	9.101	2.648	.008	.170
SB3-SB4	-40.600	9.101	-4.461	<.001	.000
SB6-SB5	3.700	9.101	.407	.684	1.000
SB6-SB1	19.900	9.101	2.187	.029	.604
SB6-SB2	23.700	9.101	2.604	.009	.193
SB6-SB4	40.200	9.101	4.417	<.001	.000
SB5-SB1	16.200	9.101	1.780	.075	1.000
SB5-SB2	20.000	9.101	2.197	.028	.588
SB5-SB4	36.500	9.101	4.010	<.001	.001
SB1-SB2	-3.800	9.101	-.418	.676	1.000
SB1-SB4	-20.300	9.101	-2.230	.026	.540
SB2-SB4	-16.500	9.101	-1.813	.070	1.000

Each row tests the null hypothesis that the Sample 1 and Sample 2 distributions are the same.
Asymptotic significances (2-sided tests) are displayed. The significance level is .050.

a. Significance values have been adjusted by the Bonferroni correction for multiple tests.

Pairwise Comparisons of Omni

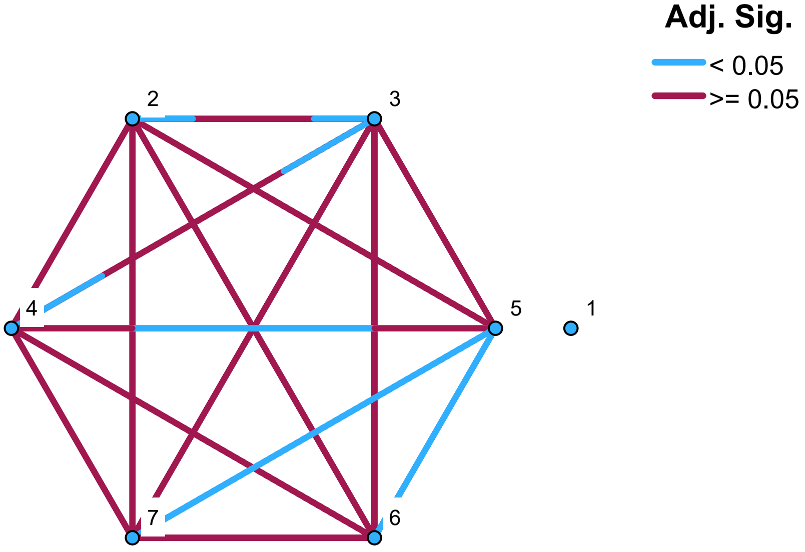

Each node shows the
sample average rank of
Omni.

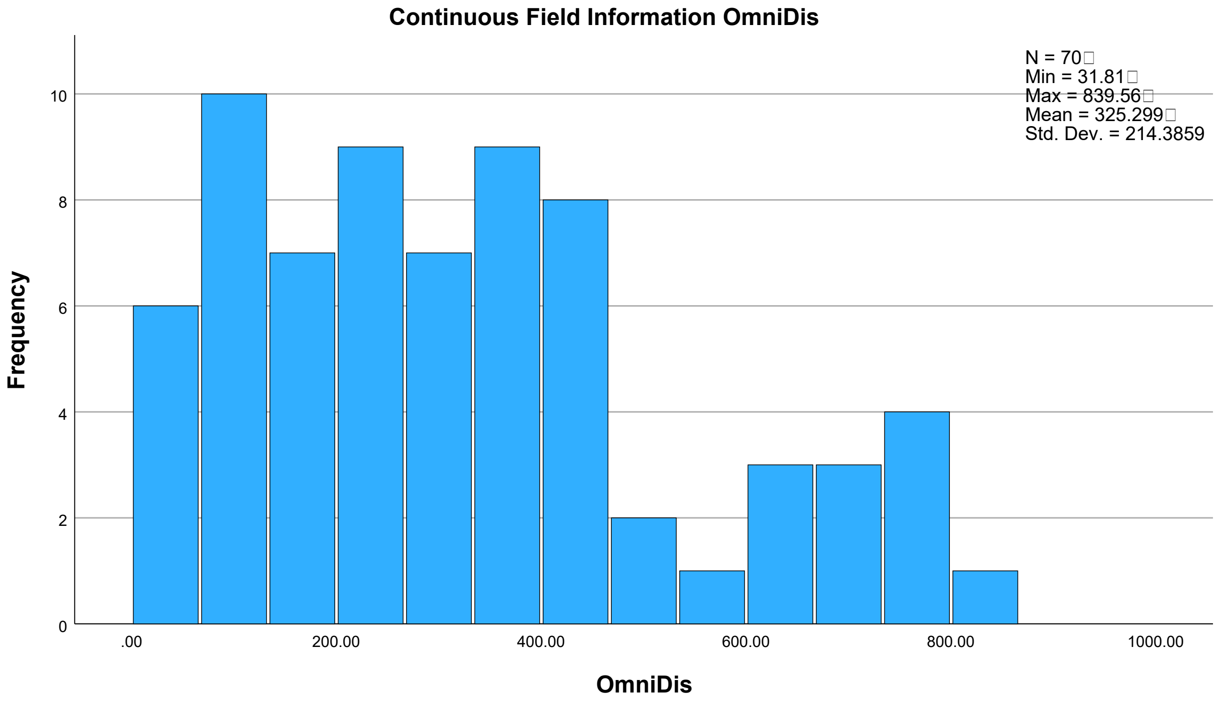
Continuous Field Information OmniDis
N = 70
Min = 31.81
Max = 839.56
Mean = 325.299
Std. Dev. = 214.3859
Frequency
0
2
4
6
8
10
.00
200.00
400.00
600.00
800.00
1000.00
OmniDis

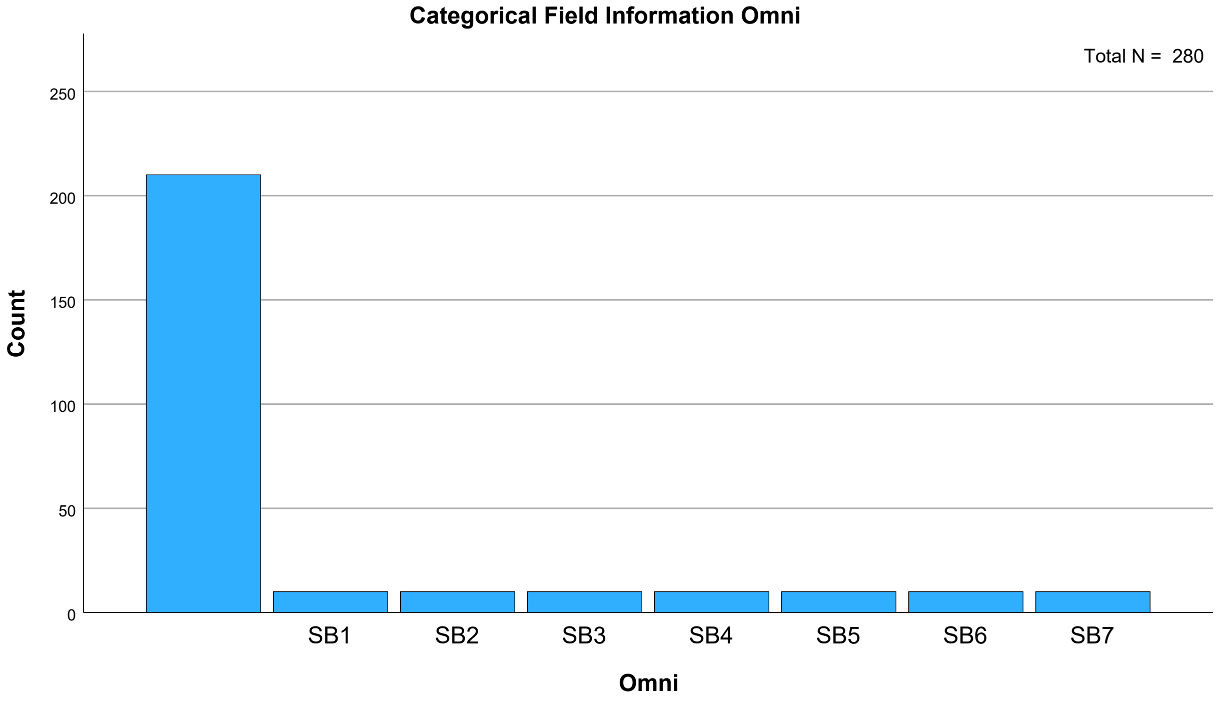
Categorical Field Information Omni
Total N = 280
Count
0
50
100
150
200
250
SB1
SB2
SB3
SB4
SB5
SB6
SB7
Omni

Nonparametric Tests

Notes

Output Created		30-MAY-2023 09:18:59
Comments		
Input	Active Dataset	DataSet2
	Filter	<none>
	Weight	<none>
	Split File	<none>
	N of Rows in Working Data File	280
Syntax		NPTESTS<br>/INDEPENDENT TEST (PrimeDis) GROUP (Prime) KRUSKAL_WALLIS (COMPARE=PAIRWISE)<br>/MISSING SCOPE=ANALYSIS USERMISSING=EXCLUDE<br>/CRITERIA ALPHA=0.05 CILEVEL=95.
Resources	Processor Time	00:00:00.78
	Elapsed Time	00:00:00.72

Hypothesis Test Summary

	Null Hypothesis	Test	Sig.[a,b]
1	The distribution of PrimeDis is the same across categories of Prime.	Independent-Samples Kruskal-Wallis Test	<.001

Hypothesis Test Summary

	Decision
1	Reject the null hypothesis.

a. The significance level is .050.

b. Asymptotic significance is displayed.

Independent-Samples Kruskal-Wallis Test

PrimeDis across Prime

Independent-Samples Kruskal-Wallis Test Summary

Total N	70
Test Statistic	35.489[a]
Degree Of Freedom	6
Asymptotic Sig.(2-sided test)	<.001

a. The test statistic is adjusted for ties.

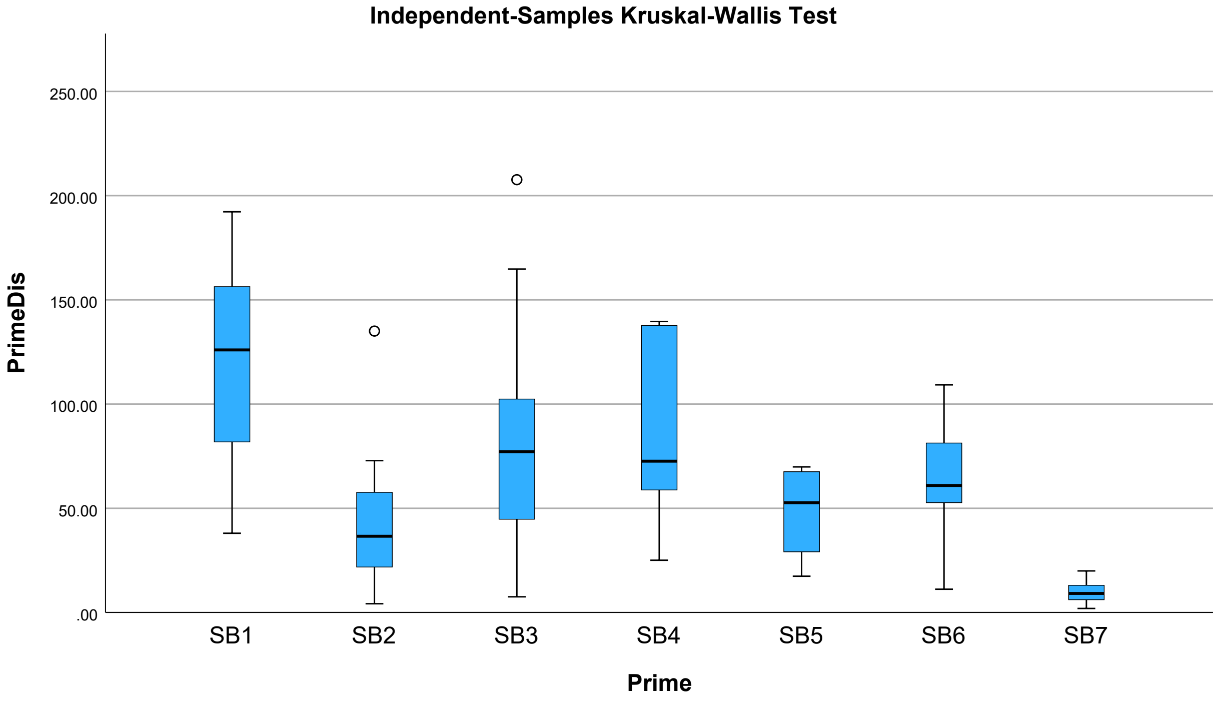

Pairwise Comparisons of Prime

Sample 1-Sample 2	Test Statistic	Std. Error	Std. Test Statistic	Sig.	Adj. Sig.[a]
SB7-SB2	19.600	9.101	2.154	.031	.657
SB7-SB5	23.200	9.101	2.549	.011	.227
SB7-SB6	30.000	9.101	3.296	<.001	.021
SB7-SB3	35.700	9.101	3.923	<.001	.002
SB7-SB4	39.700	9.101	4.362	<.001	.000
SB7-SB1	47.800	9.101	5.252	<.001	.000
SB2-SB5	-3.600	9.101	-.396	.692	1.000
SB2-SB6	-10.400	9.101	-1.143	.253	1.000
SB2-SB3	-16.100	9.101	-1.769	.077	1.000
SB2-SB4	-20.100	9.101	-2.208	.027	.571
SB2-SB1	28.200	9.101	3.098	.002	.041
SB5-SB6	-6.800	9.101	-.747	.455	1.000
SB5-SB3	12.500	9.101	1.373	.170	1.000
SB5-SB4	16.500	9.101	1.813	.070	1.000
SB5-SB1	24.600	9.101	2.703	.007	.144
SB6-SB3	5.700	9.101	.626	.531	1.000
SB6-SB4	9.700	9.101	1.066	.287	1.000
SB6-SB1	17.800	9.101	1.956	.050	1.000
SB3-SB4	-4.000	9.101	-.440	.660	1.000
SB3-SB1	12.100	9.101	1.329	.184	1.000
SB4-SB1	8.100	9.101	.890	.373	1.000

Each row tests the null hypothesis that the Sample 1 and Sample 2 distributions are the same.
Asymptotic significances (2-sided tests) are displayed. The significance level is .050.

a. Significance values have been adjusted by the Bonferroni correction for multiple tests.

Pairwise Comparisons of Prime

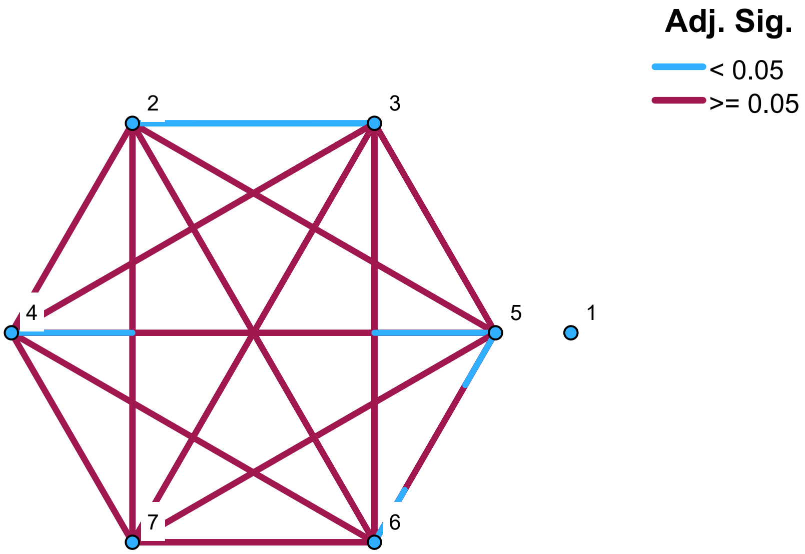

Each node shows the sample average rank of Prime.

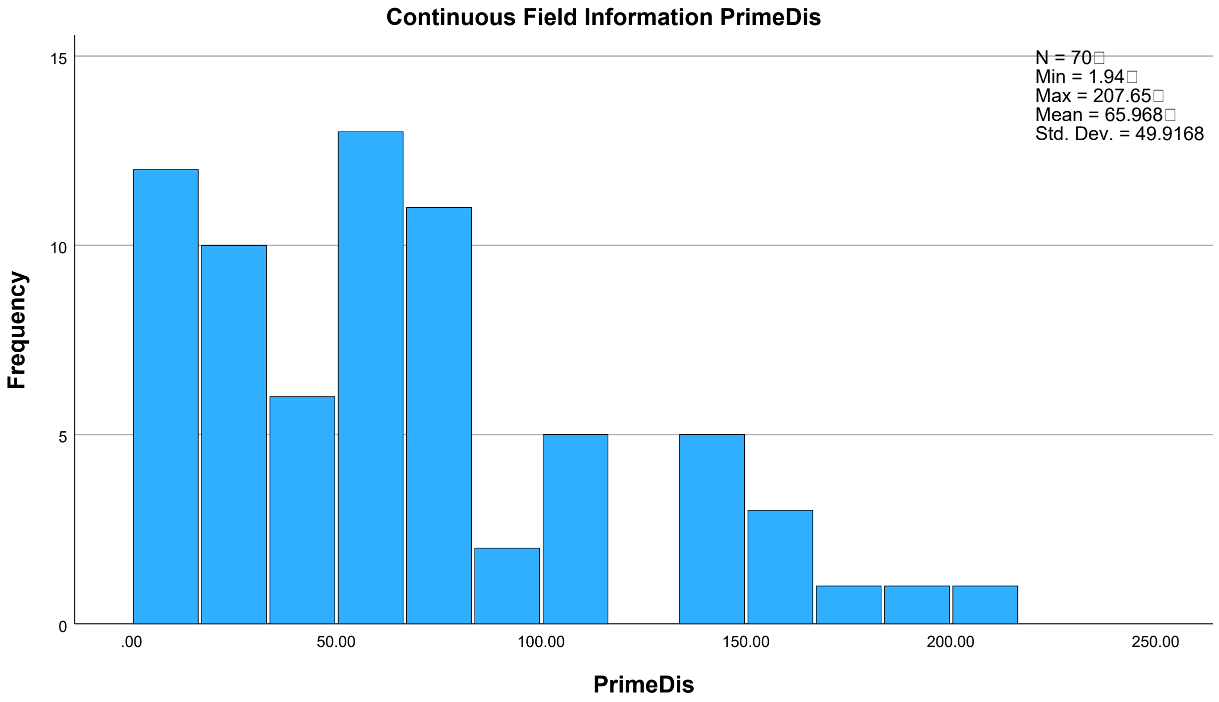
Continuous Field Information PrimeDis
N = 70
Min = 1.94
Max = 207.65
Mean = 65.968
Std. Dev. = 49.9168
Frequency
0
5
10
15
.00
50.00
100.00
150.00
200.00
250.00
PrimeDis

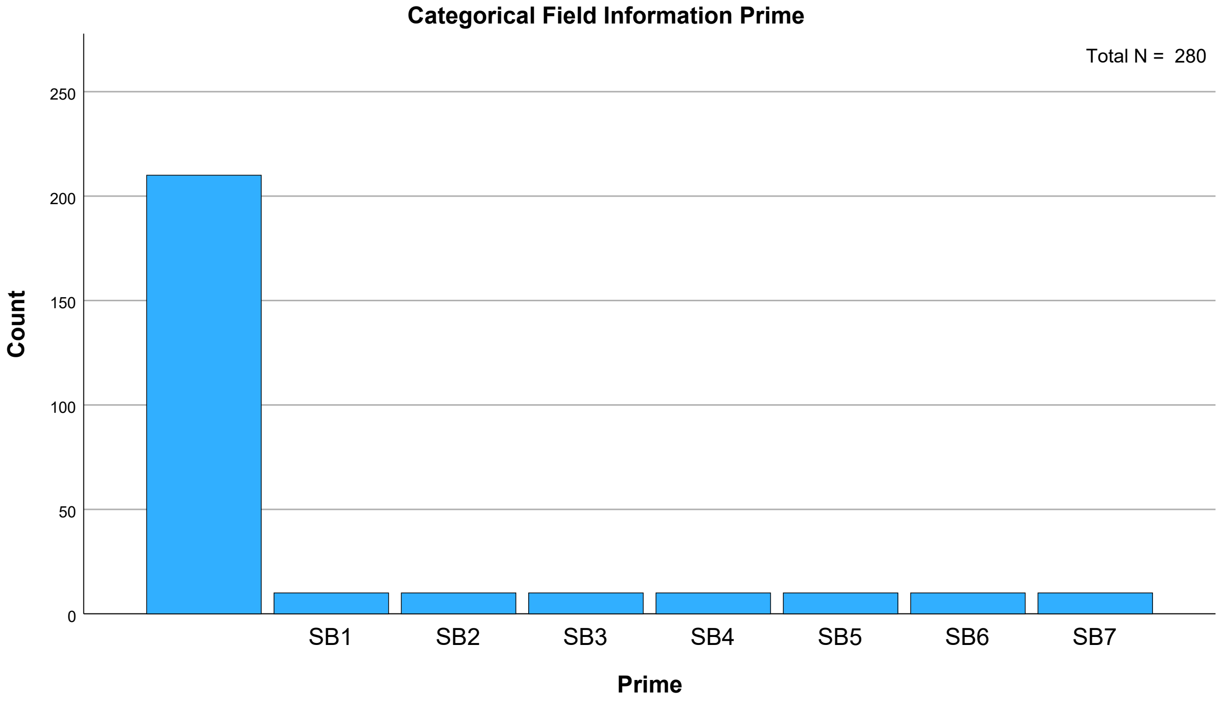
Categorical Field Information Prime
Total N = 280
Count
0
50
100
150
200
250
SB1
SB2
SB3
SB4
SB5
SB6
SB7
Prime

Nonparametric Tests

Notes

Output Created		30-MAY-2023 09:44:36
Comments		
Input	Active Dataset	DataSet2
	Filter	<none>
	Weight	<none>
	Split File	<none>
	N of Rows in Working Data File	280
Syntax		NPTESTS /INDEPENDENT TEST (DisSB1) GROUP (SB1) KRUSKAL_WALLIS (COMPARE=PAIRWISE) /MISSING SCOPE=ANALYSIS USERMISSING=EXCLUDE /CRITERIA ALPHA=0.05 CILEVEL=95.
Resources	Processor Time	00:00:00.78
	Elapsed Time	00:00:00.74

Hypothesis Test Summary

	Null Hypothesis	Test	Sig.[a,b]
1	The distribution of DisSB1 is the same across categories of SB1.	Independent-Samples Kruskal-Wallis Test	<.001

Hypothesis Test Summary

	Decision
1	Reject the null hypothesis.

a. The significance level is .050.

b. Asymptotic significance is displayed.

Independent-Samples Kruskal-Wallis Test

DisSB1 across SB1

Independent-Samples Kruskal-Wallis Test Summary

Total N	40
Test Statistic	26.980[a]
Degree Of Freedom	3
Asymptotic Sig.(2-sided test)	<.001

a. The test statistic is adjusted for ties.

Independent-Samples Kruskal-Wallis Test

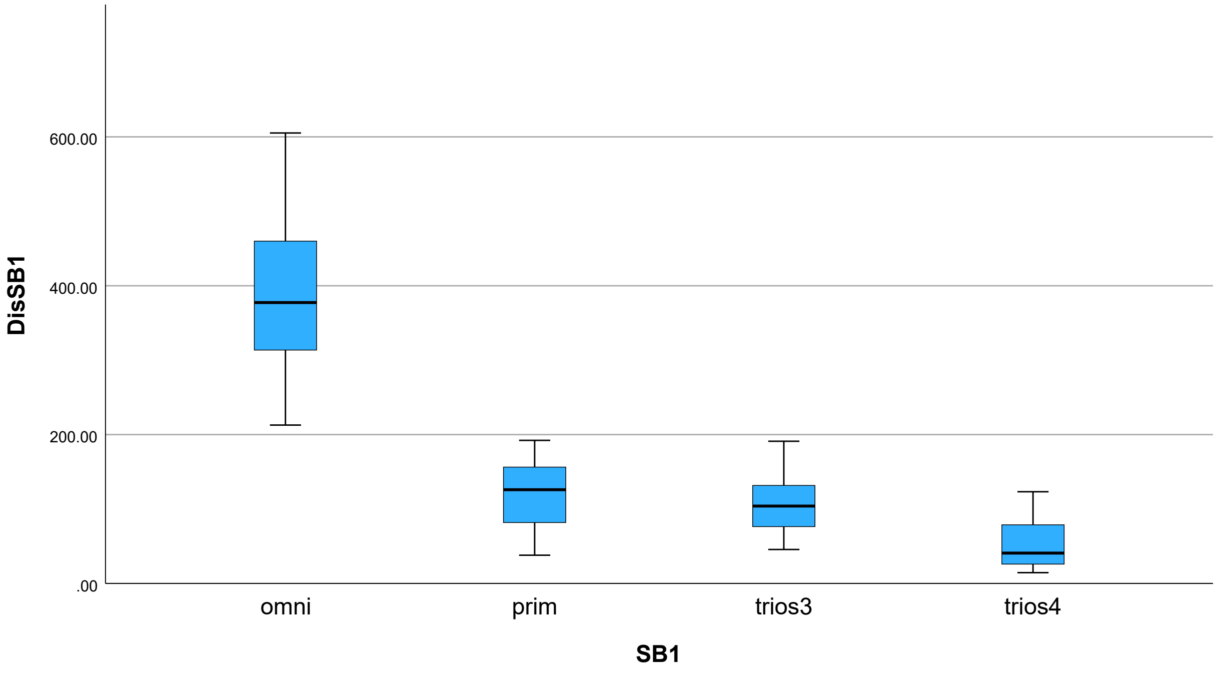

Pairwise Comparisons of SB1

Sample 1-Sample 2	Test Statistic	Std. Error	Std. Test Statistic	Sig.	Adj. Sig.[a]
trios4-trios3	8.600	5.228	1.645	.100	.600
trios4-prim	11.200	5.228	2.142	.032	.193
trios4-omni	26.600	5.228	5.088	<.001	.000
trios3-prim	2.600	5.228	.497	.619	1.000
trios3-omni	18.000	5.228	3.443	<.001	.003
prim-omni	15.400	5.228	2.946	.003	.019

Each row tests the null hypothesis that the Sample 1 and Sample 2 distributions are the same.
Asymptotic significances (2-sided tests) are displayed. The significance level is .050.

a. Significance values have been adjusted by the Bonferroni correction for multiple tests.

Pairwise Comparisons of SB1

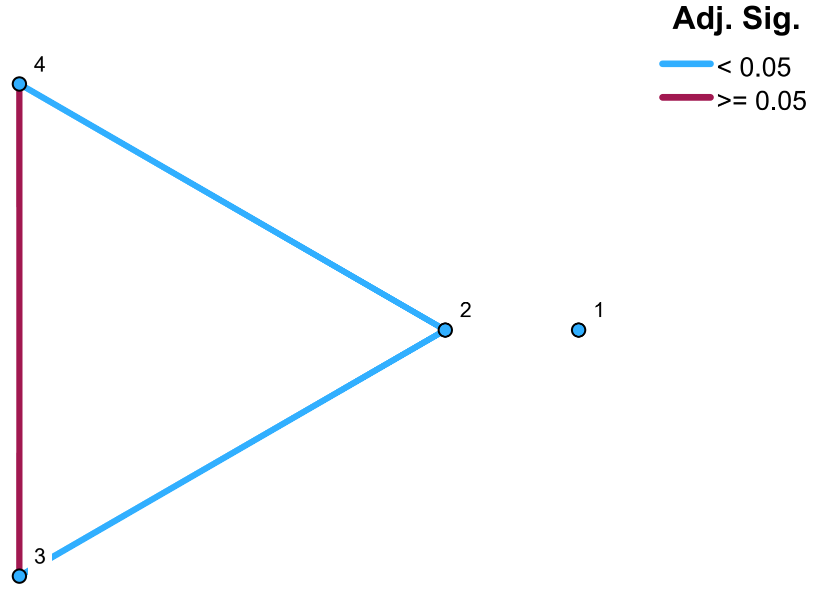

Each node shows the
sample average rank of
SB1.

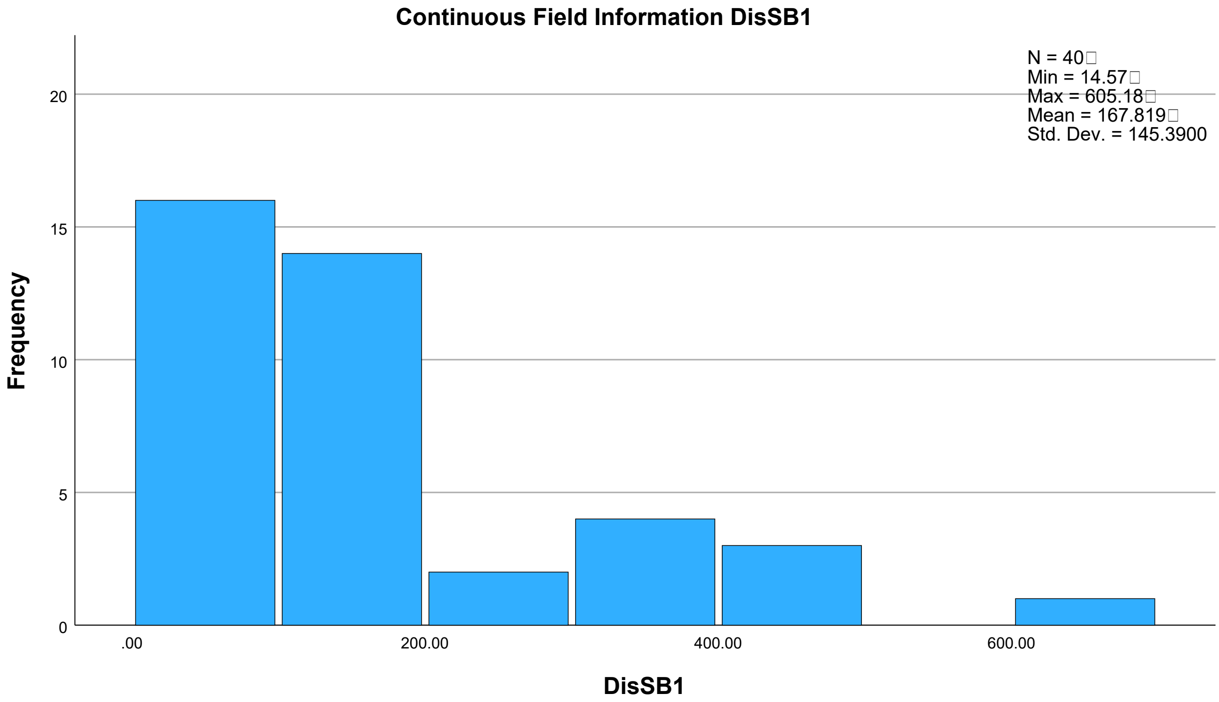
Continuous Field Information DisSB1
N = 40
Min = 14.57
Max = 605.18
Mean = 167.819
Std. Dev. = 145.3900
Frequency
20
15
10
5
0
.00
200.00
400.00
600.00
DisSB1

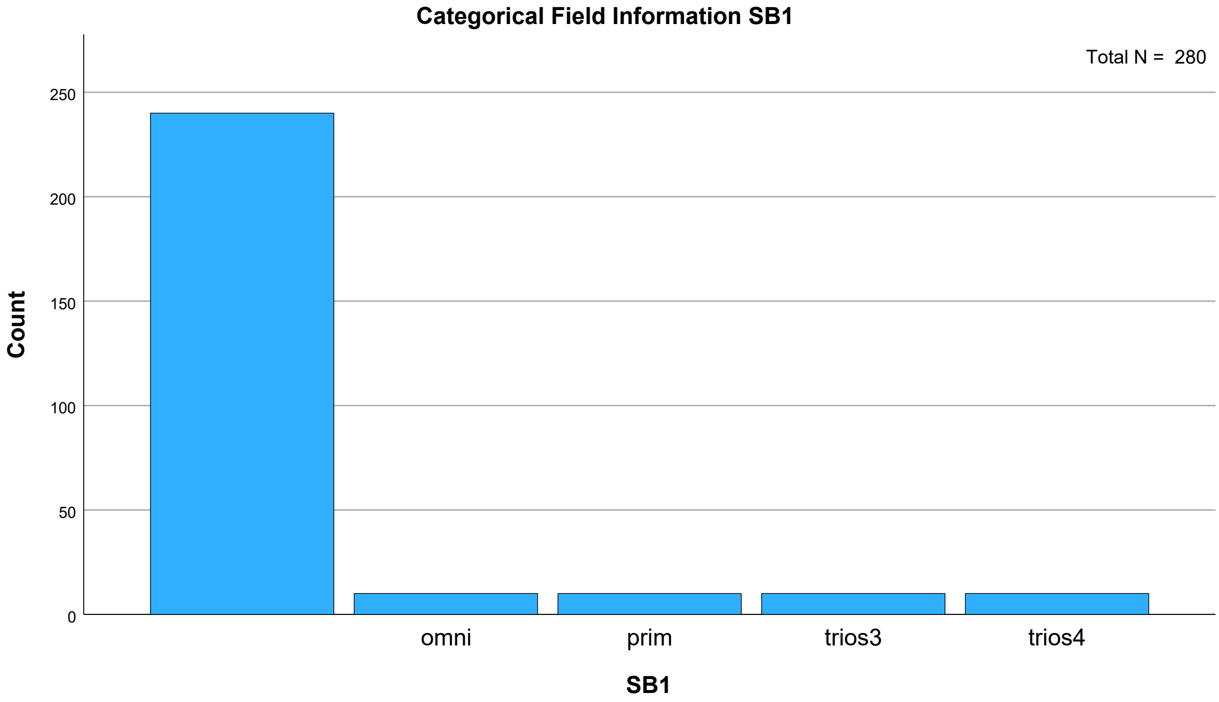
Categorical Field Information SB1
Total N = 280
Count
250
200
150
100
50
0
omni
prim
trios3
trios4
SB1

Nonparametric Tests

Notes

Output Created		30-MAY-2023 09:45:28
Comments		
Input	Active Dataset	DataSet2
	Filter	<none>
	Weight	<none>
	Split File	<none>
	N of Rows in Working Data File	280
Syntax		NPTESTS /INDEPENDENT TEST (DisSB2) GROUP (SB2) KRUSKAL_WALLIS (COMPARE=PAIRWISE) /MISSING SCOPE=ANALYSIS USERMISSING=EXCLUDE /CRITERIA ALPHA=0.05 CILEVEL=95.
Resources	Processor Time	00:00:00.72
	Elapsed Time	00:00:00.72

Hypothesis Test Summary

	Null Hypothesis	Test	Sig.[a,b]
1	The distribution of DisSB2 is the same across categories of SB2.	Independent-Samples Kruskal-Wallis Test	<.001

Hypothesis Test Summary

	Decision
1	Reject the null hypothesis.

a. The significance level is .050.

b. Asymptotic significance is displayed.

Independent-Samples Kruskal-Wallis Test

DisSB2 across SB2

Independent-Samples Kruskal-Wallis Test Summary

Total N	40
Test Statistic	23.887[a]
Degree Of Freedom	3
Asymptotic Sig.(2-sided test)	<.001

a. The test statistic is adjusted for ties.

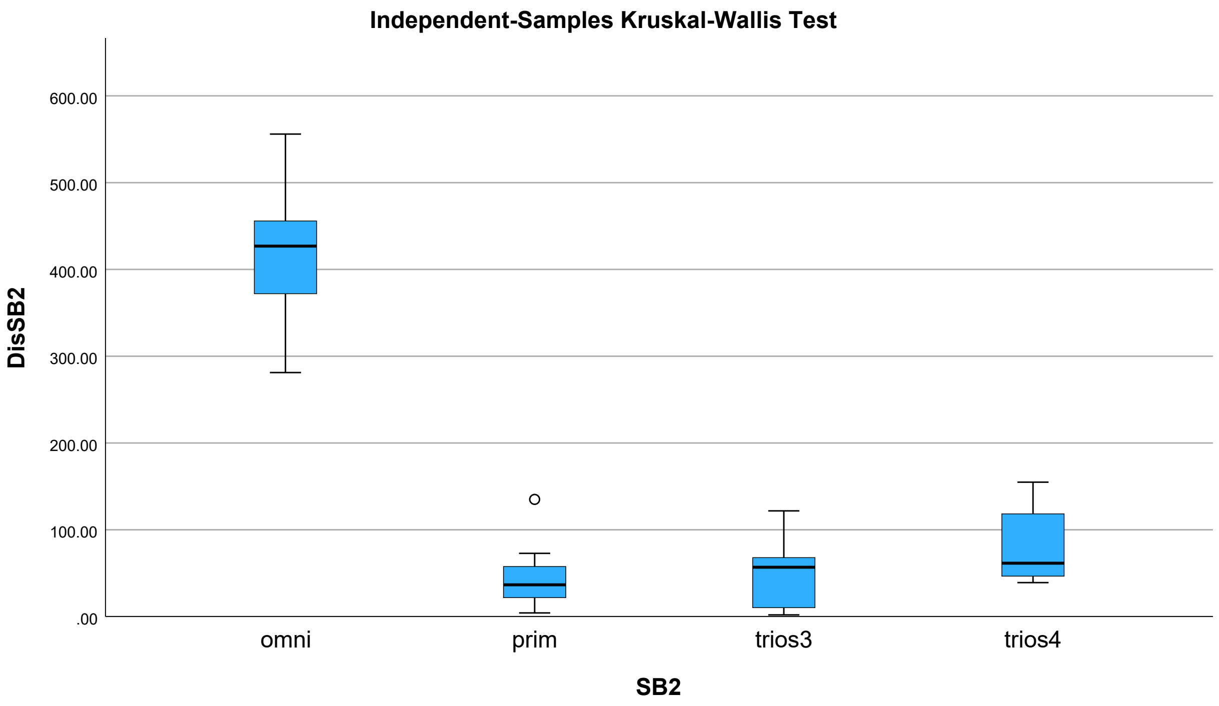

Pairwise Comparisons of SB2

Sample 1-Sample 2	Test Statistic	Std. Error	Std. Test Statistic	Sig.	Adj. Sig.[a]
prim-trios3	-2.700	5.228	-.516	.606	1.000
prim-trios4	-7.200	5.228	-1.377	.168	1.000
prim-omni	23.300	5.228	4.457	<.001	.000
trios3-trios4	-4.500	5.228	-.861	.389	1.000
trios3-omni	20.600	5.228	3.940	<.001	.000
trios4-omni	16.100	5.228	3.079	.002	.012

Each row tests the null hypothesis that the Sample 1 and Sample 2 distributions are the same.
Asymptotic significances (2-sided tests) are displayed. The significance level is .050.

a. Significance values have been adjusted by the Bonferroni correction for multiple tests.

Pairwise Comparisons of SB2

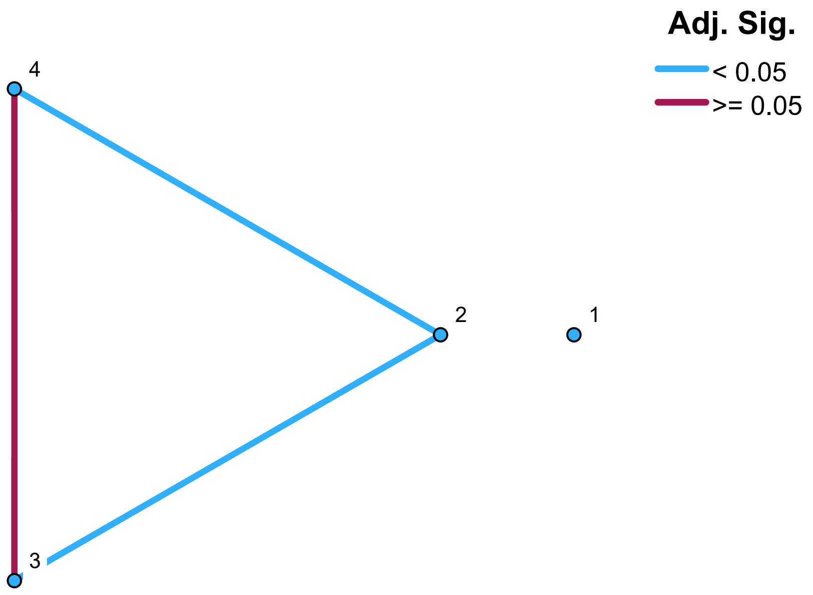

Each node shows the
sample average rank of
SB2.

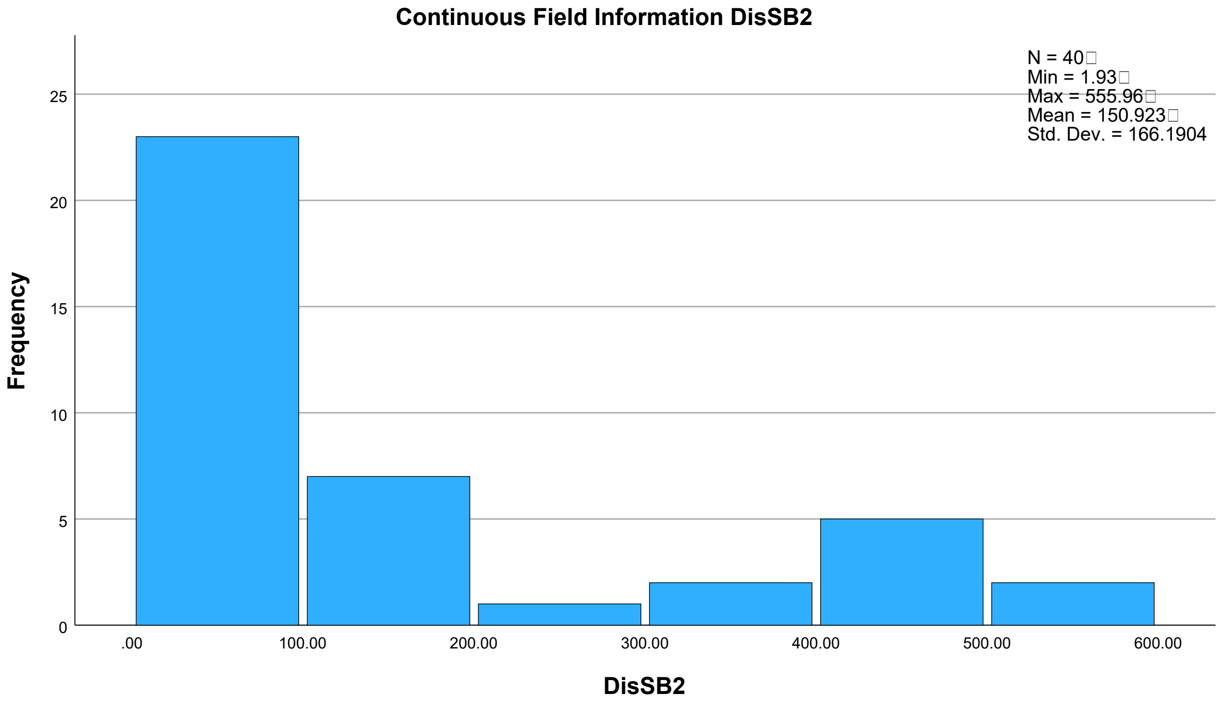
Continuous Field Information DisSB2
N = 40
Min = 1.93
Max = 555.96
Mean = 150.923
Std. Dev. = 166.1904
Frequency
0
5
10
15
20
25
.00
100.00
200.00
300.00
400.00
500.00
600.00
DisSB2

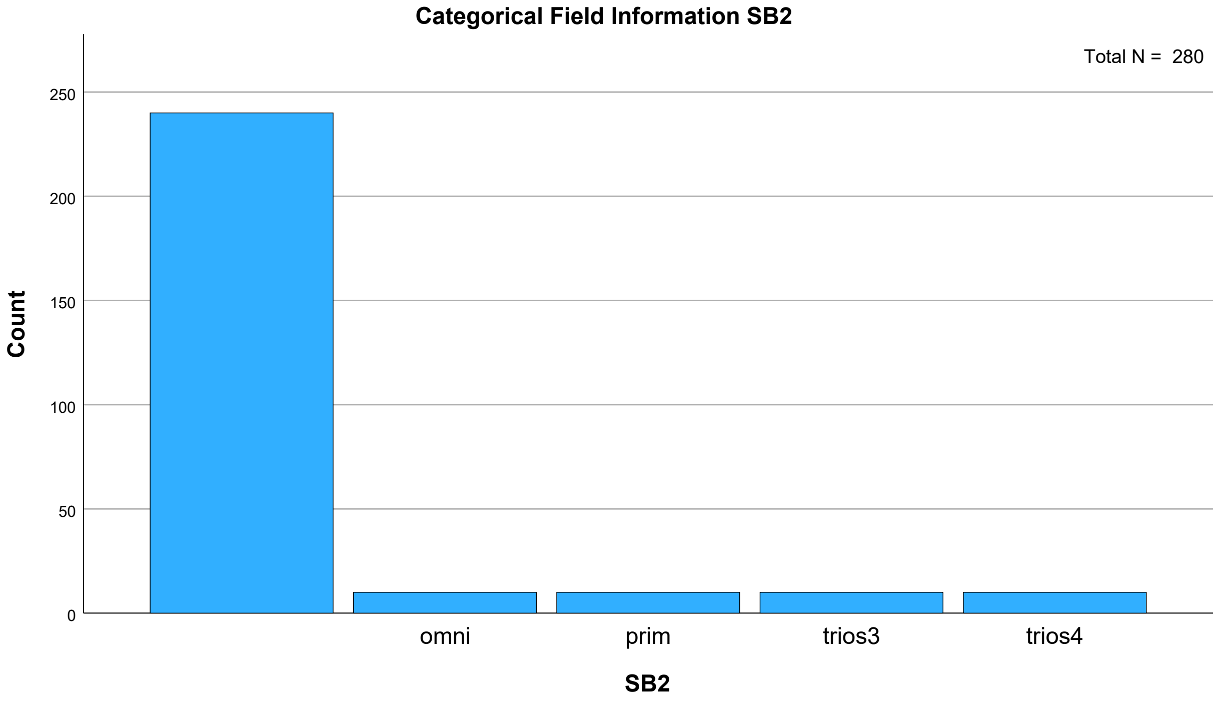
Categorical Field Information SB2
Total N = 280
Count
0
50
100
150
200
250
omni
prim
trios3
trios4
SB2

Nonparametric Tests

Notes

Output Created		30-MAY-2023 09:46:11
Comments		
Input	Active Dataset	DataSet2
	Filter	<none>
	Weight	<none>
	Split File	<none>
	N of Rows in Working Data File	280
Syntax		NPTESTS /INDEPENDENT TEST (DisSB3) GROUP (SB3) KRUSKAL_WALLIS (COMPARE=PAIRWISE) /MISSING SCOPE=ANALYSIS USERMISSING=EXCLUDE /CRITERIA ALPHA=0.05 CILEVEL=95.
Resources	Processor Time	00:00:00.72
	Elapsed Time	00:00:00.69

Hypothesis Test Summary

	Null Hypothesis	Test	Sig.[a,b]
1	The distribution of DisSB3 is the same across categories of SB3.	Independent-Samples Kruskal-Wallis Test	.087

Hypothesis Test Summary

	Decision
1	Retain the null hypothesis.

a. The significance level is .050.

b. Asymptotic significance is displayed.

Independent-Samples Kruskal-Wallis Test

DisSB3 across SB3

Independent-Samples Kruskal-Wallis Test Summary

Total N	40
Test Statistic	6.578[a]
Degree Of Freedom	3
Asymptotic Sig.(2-sided test)	.087

a. The test statistic is adjusted for ties.

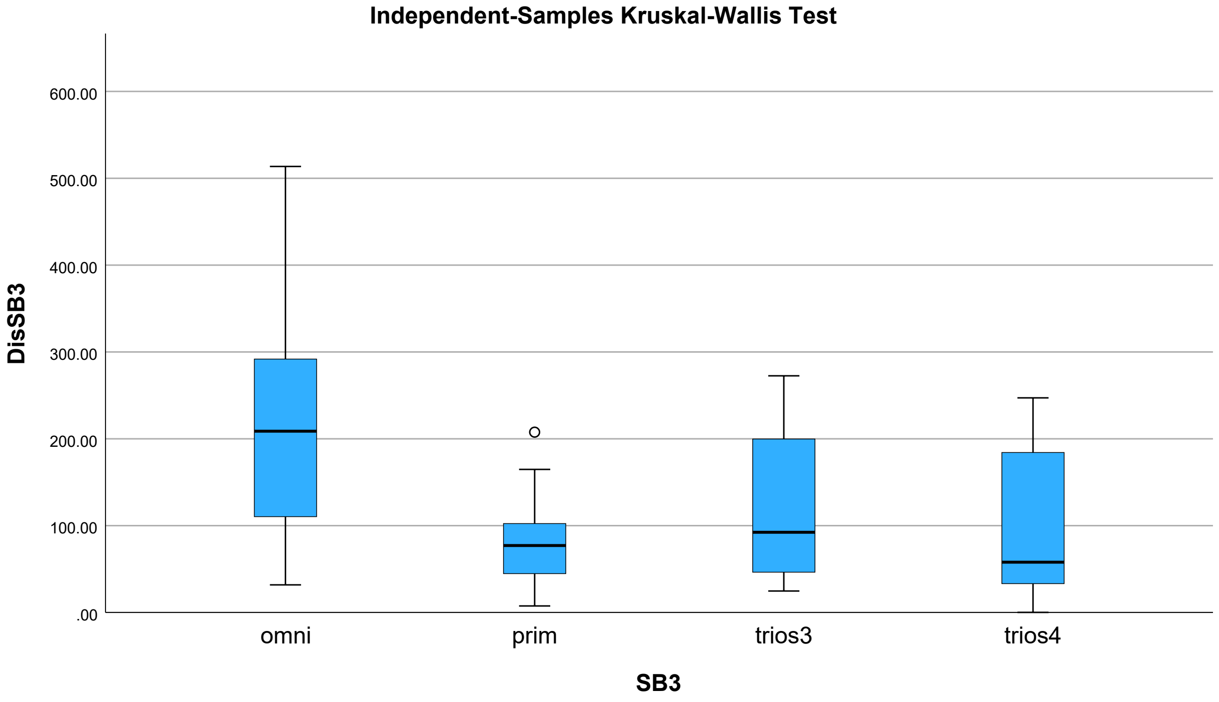

Pairwise Comparisons of SB3

Sample 1-Sample 2	Test Statistic	Std. Error	Std. Test Statistic	Sig.	Adj. Sig.[a]
trios4-prim	.600	5.228	.115	.909	1.000
trios4-trios3	4.300	5.228	.822	.411	1.000
trios4-omni	11.900	5.228	2.276	.023	.137
prim-trios3	-3.700	5.228	-.708	.479	1.000
prim-omni	11.300	5.228	2.161	.031	.184
trios3-omni	7.600	5.228	1.454	.146	.876

Each row tests the null hypothesis that the Sample 1 and Sample 2 distributions are the same.
Asymptotic significances (2-sided tests) are displayed. The significance level is .050.

a. Significance values have been adjusted by the Bonferroni correction for multiple tests.

Pairwise Comparisons of SB3

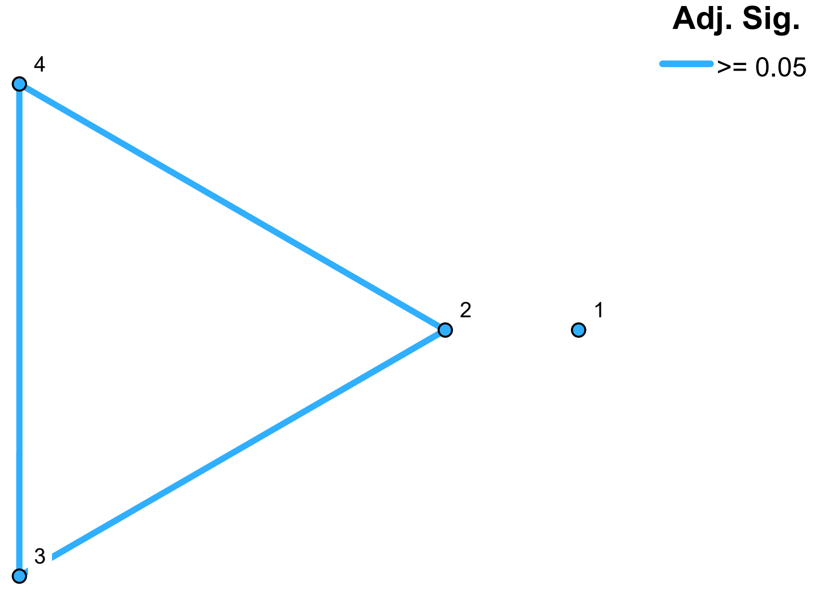

Each node shows the
sample average rank of
SB3.

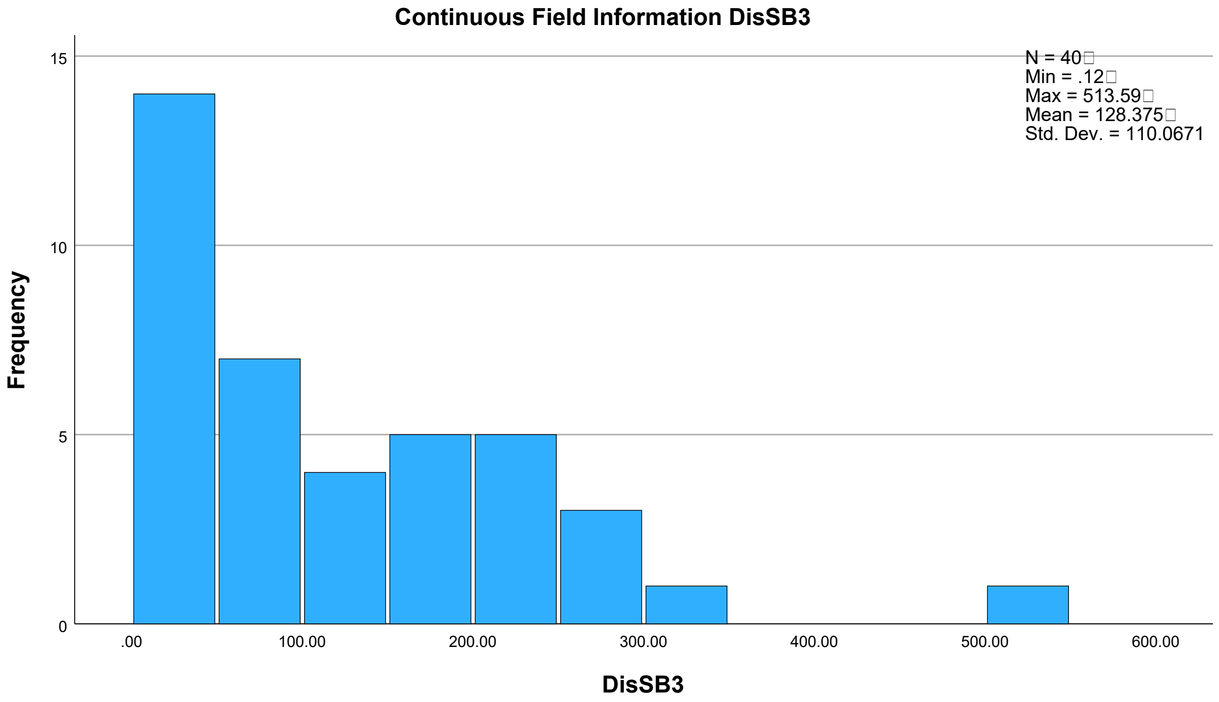
Continuous Field Information DisSB3
N = 40
Min = .12
Max = 513.59
Mean = 128.375
Std. Dev. = 110.0671
Frequency
0
5
10
15
.00
100.00
200.00
300.00
400.00
500.00
600.00
DisSB3

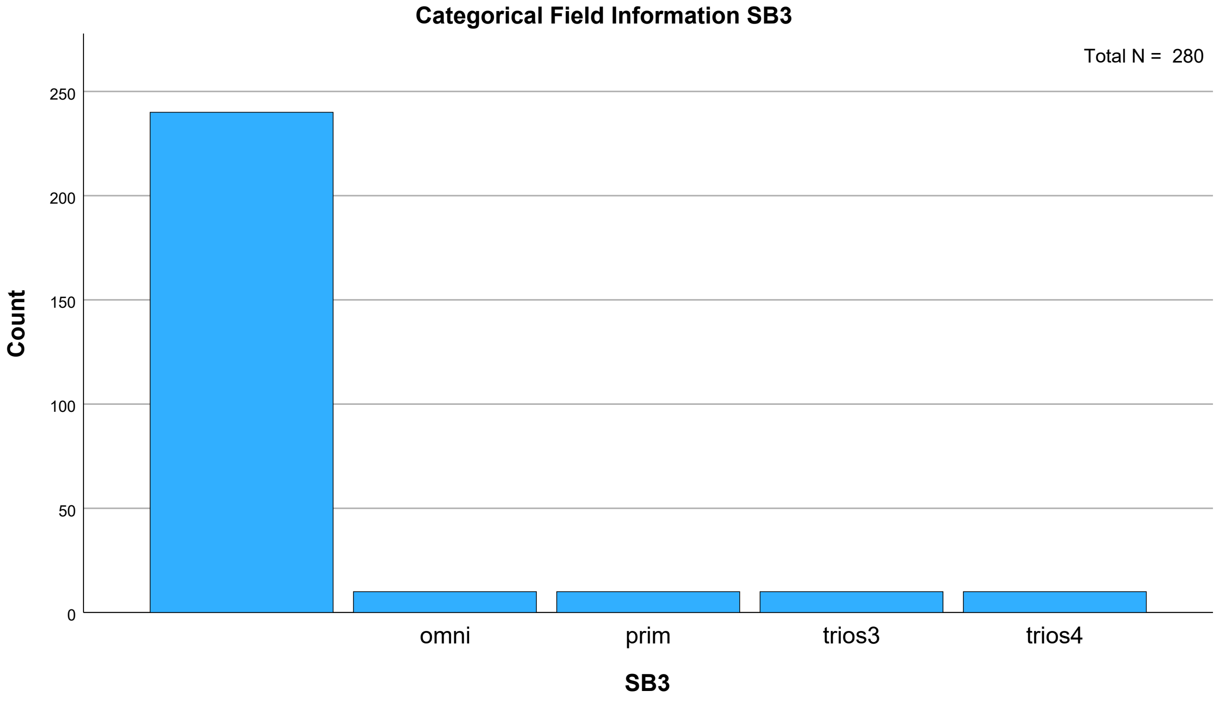
Categorical Field Information SB3
Total N = 280
Count
0
50
100
150
200
250
omni
prim
trios3
trios4
SB3

Nonparametric Tests

Notes

Output Created		30-MAY-2023 09:46:43
Comments		
Input	Active Dataset	DataSet2
	Filter	<none>
	Weight	<none>
	Split File	<none>
	N of Rows in Working Data File	280
Syntax		NPTESTS /INDEPENDENT TEST (DisSB4) GROUP (SB4) KRUSKAL_WALLIS (COMPARE=PAIRWISE) /MISSING SCOPE=ANALYSIS USERMISSING=EXCLUDE /CRITERIA ALPHA=0.05 CILEVEL=95.
Resources	Processor Time	00:00:00.70
	Elapsed Time	00:00:00.72

Hypothesis Test Summary

	Null Hypothesis	Test	Sig.[a,b]
1	The distribution of DisSB4 is the same across categories of SB4.	Independent-Samples Kruskal-Wallis Test	<.001

Hypothesis Test Summary

	Decision
1	Reject the null hypothesis.

a. The significance level is .050.

b. Asymptotic significance is displayed.

Independent-Samples Kruskal-Wallis Test

DisSB4 across SB4

Independent-Samples Kruskal-Wallis Test Summary

Total N	40
Test Statistic	23.650[a]
Degree Of Freedom	3
Asymptotic Sig.(2-sided test)	<.001

a. The test statistic is adjusted for ties.

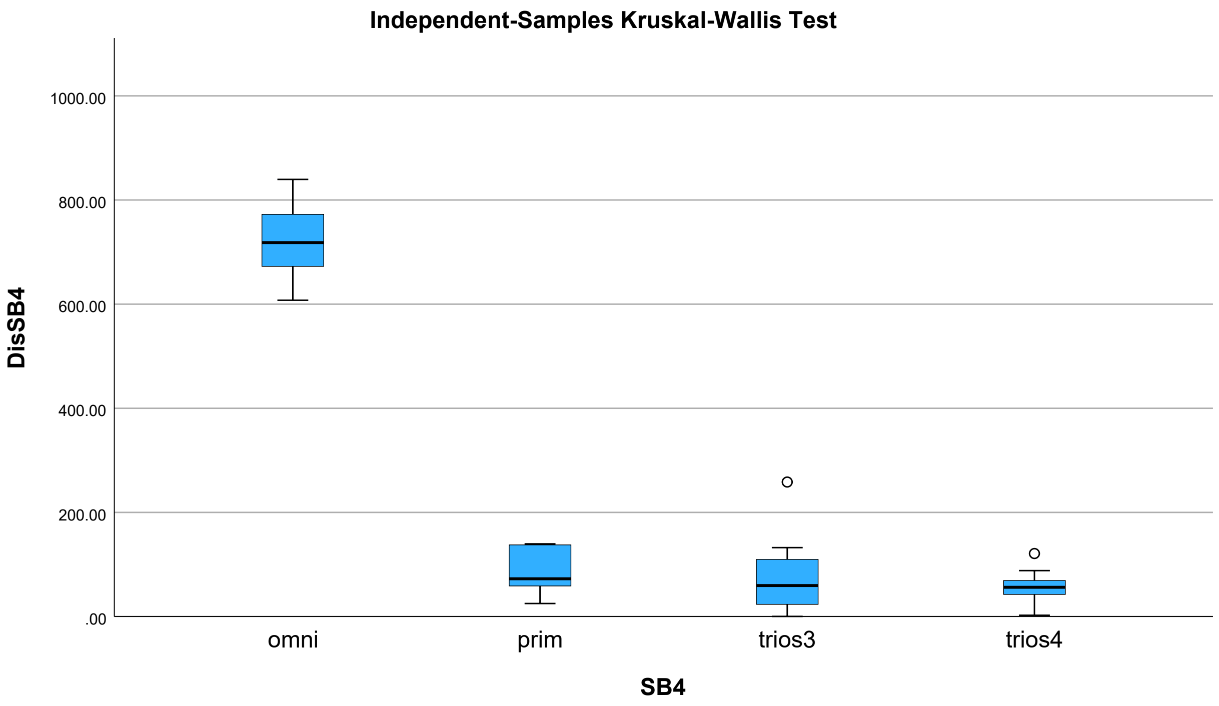

Pairwise Comparisons of SB4

Sample 1-Sample 2	Test Statistic	Std. Error	Std. Test Statistic	Sig.	Adj. Sig.[a]
trios4-trios3	2.300	5.228	.440	.660	1.000
trios4-prim	6.700	5.228	1.282	.200	1.000
trios4-omni	23.000	5.228	4.399	<.001	.000
trios3-prim	4.400	5.228	.842	.400	1.000
trios3-omni	20.700	5.228	3.960	<.001	.000
prim-omni	16.300	5.228	3.118	.002	.011

Each row tests the null hypothesis that the Sample 1 and Sample 2 distributions are the same.
Asymptotic significances (2-sided tests) are displayed. The significance level is .050.

a. Significance values have been adjusted by the Bonferroni correction for multiple tests.

Pairwise Comparisons of SB4

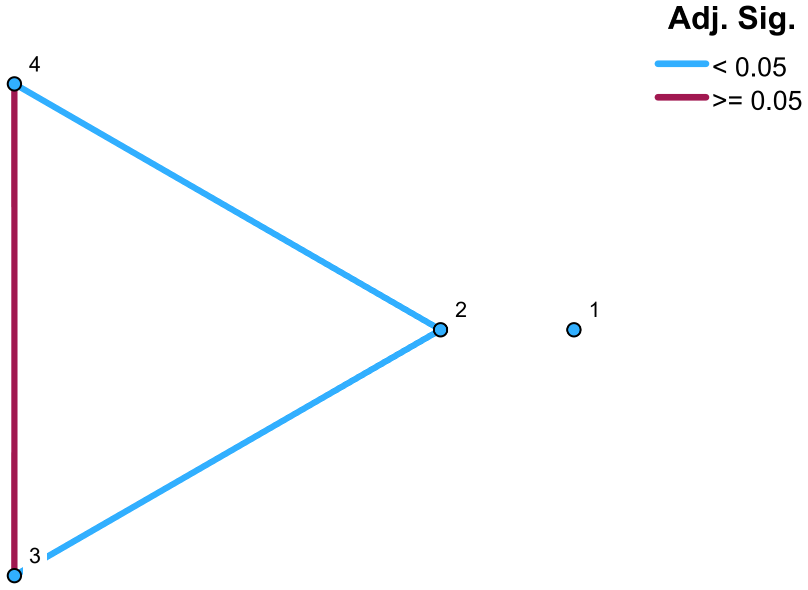

Each node shows the sample average rank of SB4.

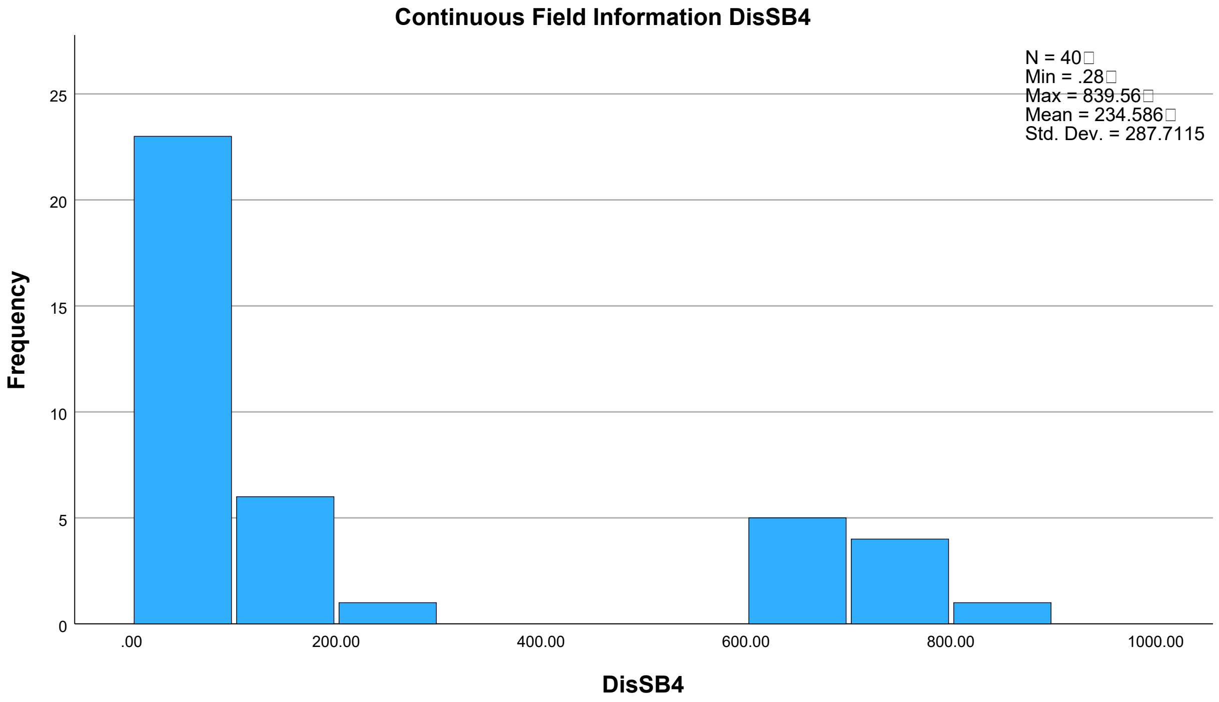
Continuous Field Information DisSB4
N = 40
Min = .28
Max = 839.56
Mean = 234.586
Std. Dev. = 287.7115
Frequency
0
5
10
15
20
25
.00
200.00
400.00
600.00
800.00
1000.00
DisSB4

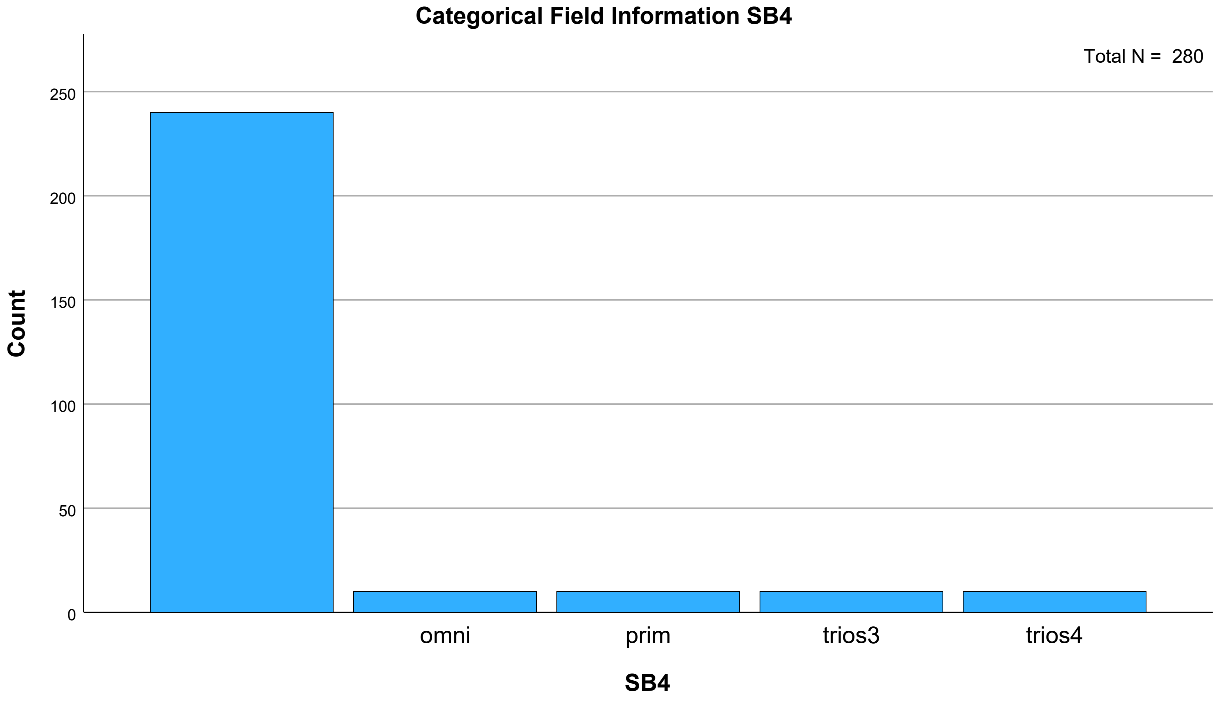
Categorical Field Information SB4
Total N = 280
Count
0
50
100
150
200
250
omni
prim
trios3
trios4
SB4

Nonparametric Tests

Notes

Output Created		30-MAY-2023 09:48:50
Comments		
Input	Active Dataset	DataSet2
	Filter	<none>
	Weight	<none>
	Split File	<none>
	N of Rows in Working Data File	280
Syntax		NPTESTS<br>/INDEPENDENT TEST (DisSB5) GROUP (SB5) KRUSKAL_WALLIS (COMPARE=PAIRWISE)<br>/MISSING SCOPE=ANALYSIS USERMISSING=EXCLUDE<br>/CRITERIA ALPHA=0.05 CILEVEL=95.
Resources	Processor Time	00:00:00.67
	Elapsed Time	00:00:00.69

Hypothesis Test Summary

	Null Hypothesis	Test	Sig.[a,b]
1	The distribution of DisSB5 is the same across categories of SB5.	Independent-Samples Kruskal-Wallis Test	<.001

Hypothesis Test Summary

	Decision
1	Reject the null hypothesis.

a. The significance level is .050.

b. Asymptotic significance is displayed.

Independent-Samples Kruskal-Wallis Test

DisSB5 across SB5

Independent-Samples Kruskal-Wallis Test Summary

Total N	40
Test Statistic	18.887[a]
Degree Of Freedom	3
Asymptotic Sig.(2-sided test)	<.001

a. The test statistic is adjusted for ties.

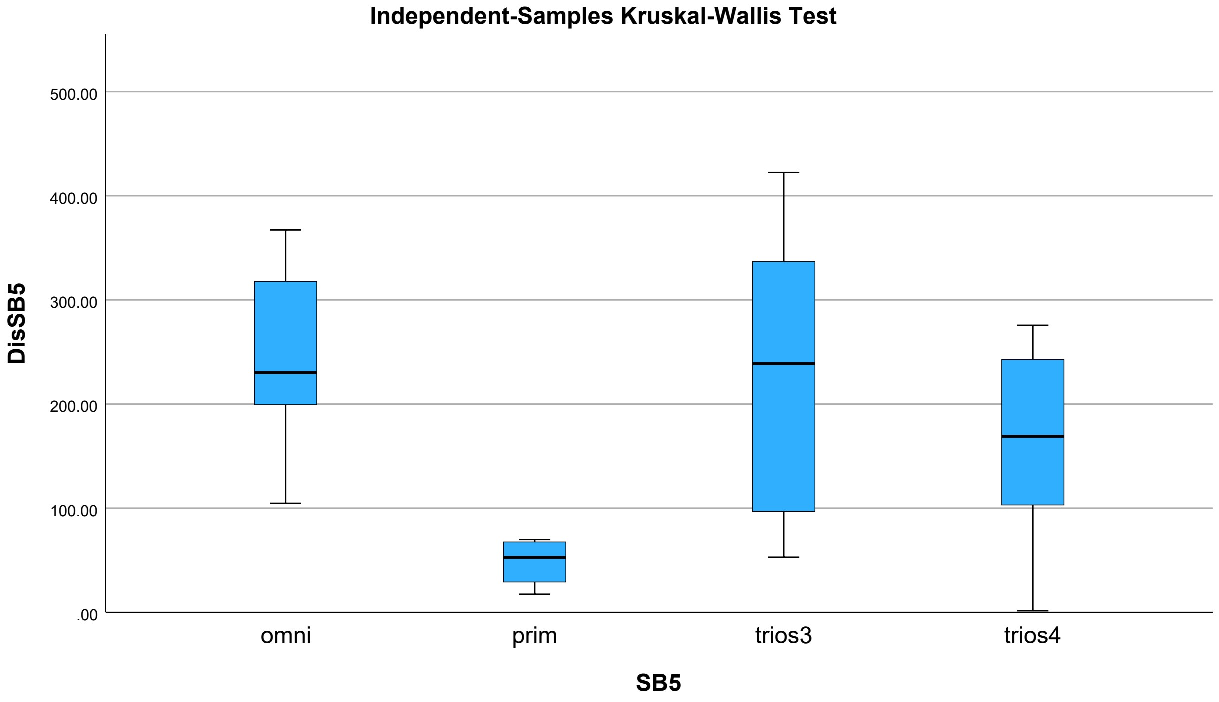

Pairwise Comparisons of SB5

Sample 1-Sample 2	Test Statistic	Std. Error	Std. Test Statistic	Sig.	Adj. Sig.[a]
prim-trios4	-13.400	5.228	-2.563	.010	.062
prim-trios3	-18.000	5.228	-3.443	<.001	.003
prim-omni	21.000	5.228	4.017	<.001	.000
trios4-trios3	4.600	5.228	.880	.379	1.000
trios4-omni	7.600	5.228	1.454	.146	.876
trios3-omni	3.000	5.228	.574	.566	1.000

Each row tests the null hypothesis that the Sample 1 and Sample 2 distributions are the same.
Asymptotic significances (2-sided tests) are displayed. The significance level is .050.

a. Significance values have been adjusted by the Bonferroni correction for multiple tests.

Pairwise Comparisons of SB5

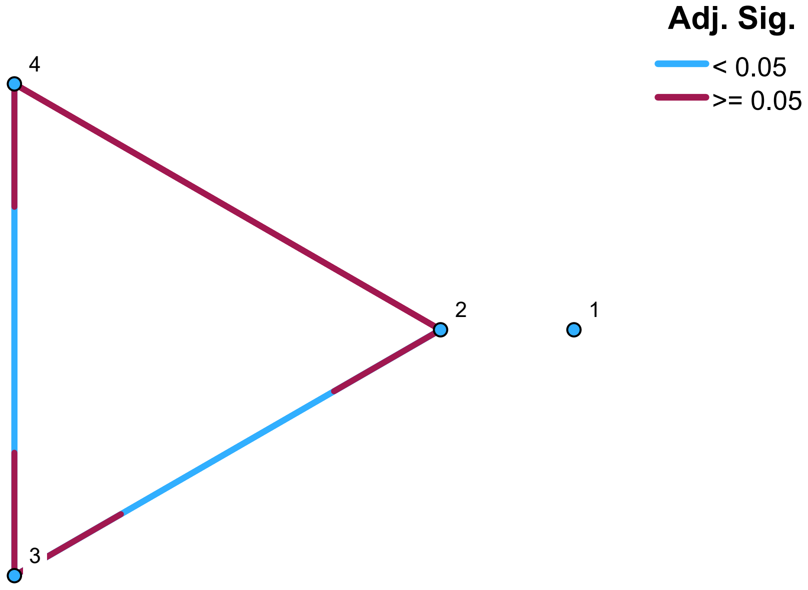

Each node shows the
sample average rank of
SB5.

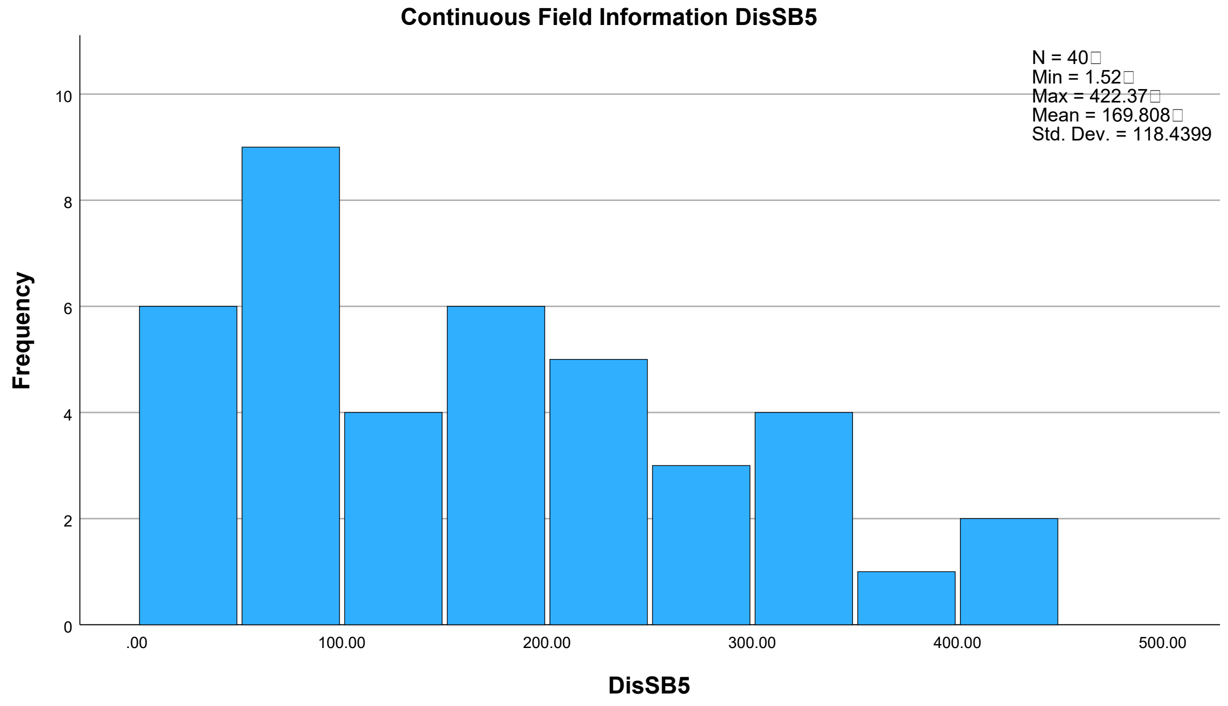
Continuous Field Information DisSB5
N = 40
Min = 1.52
Max = 422.37
Mean = 169.808
Std. Dev. = 118.4399
Frequency
10
8
6
4
2
0
.00
100.00
200.00
300.00
400.00
500.00
DisSB5

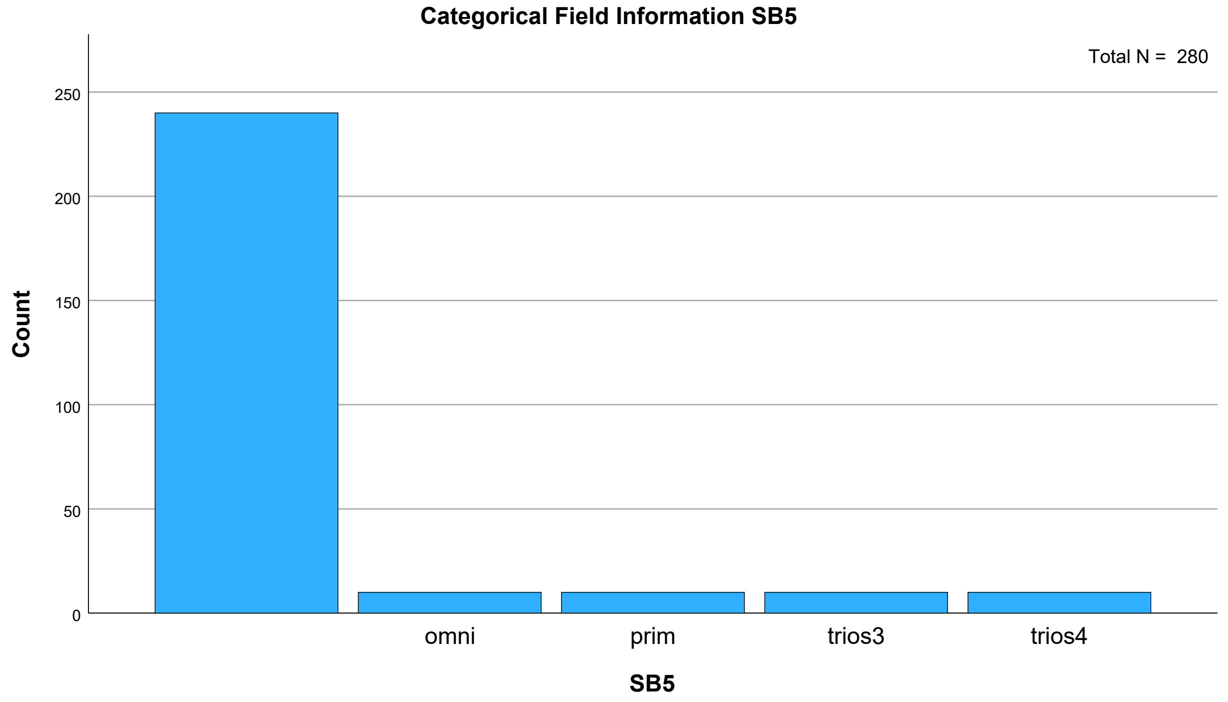
Categorical Field Information SB5
Total N = 280
Count
250
200
150
100
50
0
omni
prim
trios3
trios4
SB5

Nonparametric Tests

Notes

Output Created		30-MAY-2023 09:50:31
Comments		
Input	Active Dataset	DataSet2
	Filter	<none>
	Weight	<none>
	Split File	<none>
	N of Rows in Working Data File	280
Syntax		NPTESTS /INDEPENDENT TEST (DisSB6) GROUP (SB6) KRUSKAL_WALLIS (COMPARE=PAIRWISE) /MISSING SCOPE=ANALYSIS USERMISSING=EXCLUDE /CRITERIA ALPHA=0.05 CILEVEL=95.
Resources	Processor Time	00:00:00.67
	Elapsed Time	00:00:00.70

Hypothesis Test Summary

	Null Hypothesis	Test	Sig.[a,b]
1	The distribution of DisSB6 is the same across categories of SB6.	Independent-Samples Kruskal-Wallis Test	<.001

Hypothesis Test Summary

	Decision
1	Reject the null hypothesis.

a. The significance level is .050.

b. Asymptotic significance is displayed.

Independent-Samples Kruskal-Wallis Test

DisSB6 across SB6

Independent-Samples Kruskal-Wallis Test Summary

Total N	40
Test Statistic	20.786[a]
Degree Of Freedom	3
Asymptotic Sig.(2-sided test)	<.001

a. The test statistic is adjusted for ties.

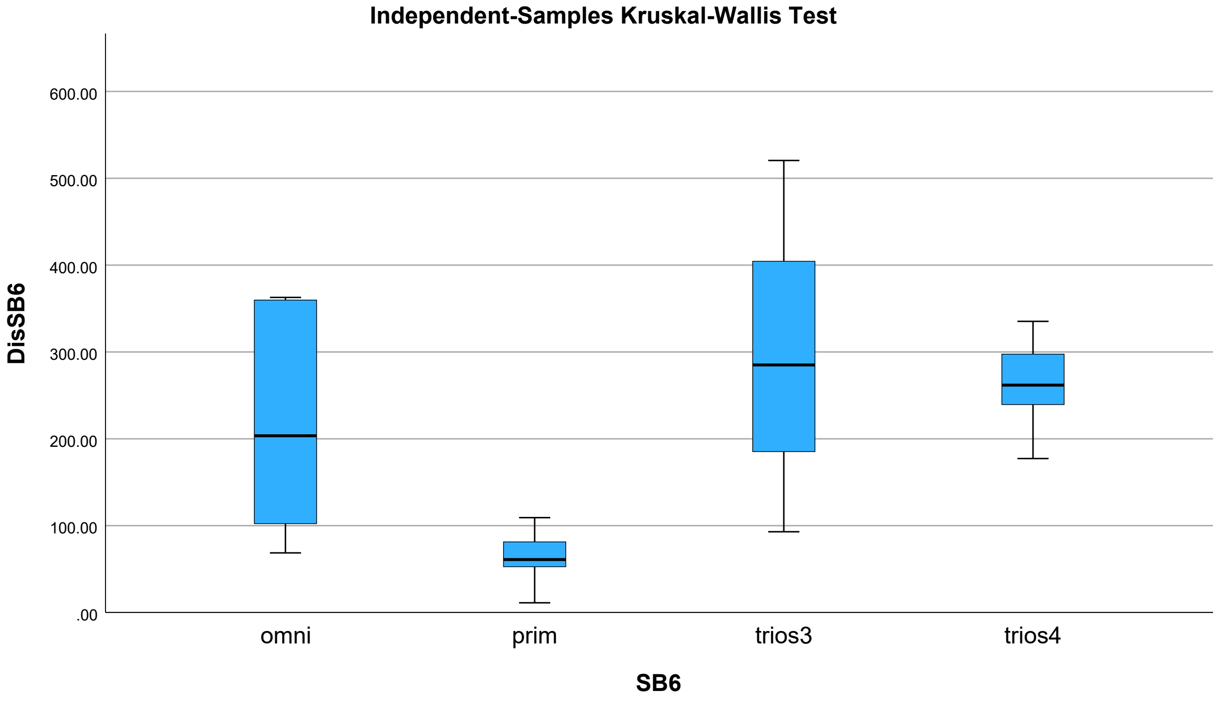

Pairwise Comparisons of SB6

Sample 1-Sample 2	Test Statistic	Std. Error	Std. Test Statistic	Sig.	Adj. Sig.[a]
prim-omni	15.800	5.228	3.022	.003	.015
prim-trios4	-20.000	5.228	-3.825	<.001	.001
prim-trios3	-21.000	5.228	-4.017	<.001	.000
omni-trios4	-4.200	5.228	-.803	.422	1.000
omni-trios3	-5.200	5.228	-.995	.320	1.000
trios4-trios3	1.000	5.228	.191	.848	1.000

Each row tests the null hypothesis that the Sample 1 and Sample 2 distributions are the same.
Asymptotic significances (2-sided tests) are displayed. The significance level is .050.

a. Significance values have been adjusted by the Bonferroni correction for multiple tests.

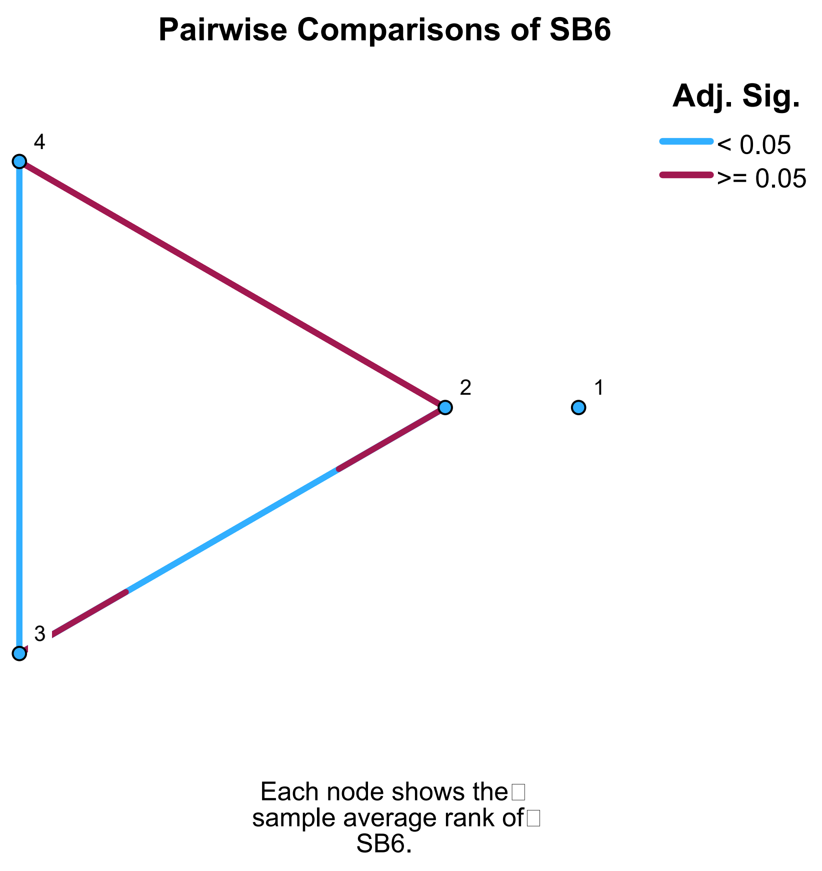

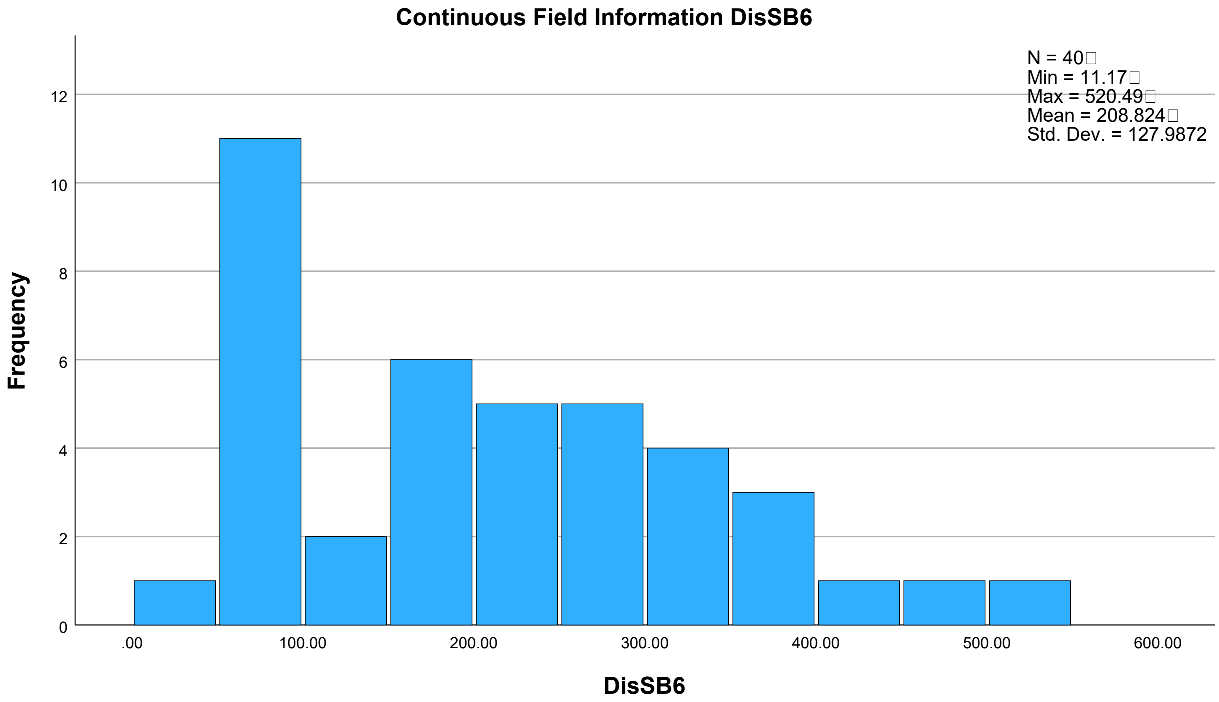
Continuous Field Information DisSB6
N = 40
Min = 11.17
Max = 520.49
Mean = 208.824
Std. Dev. = 127.9872
Frequency
0
2
4
6
8
10
12
.00
100.00
200.00
300.00
400.00
500.00
600.00
DisSB6

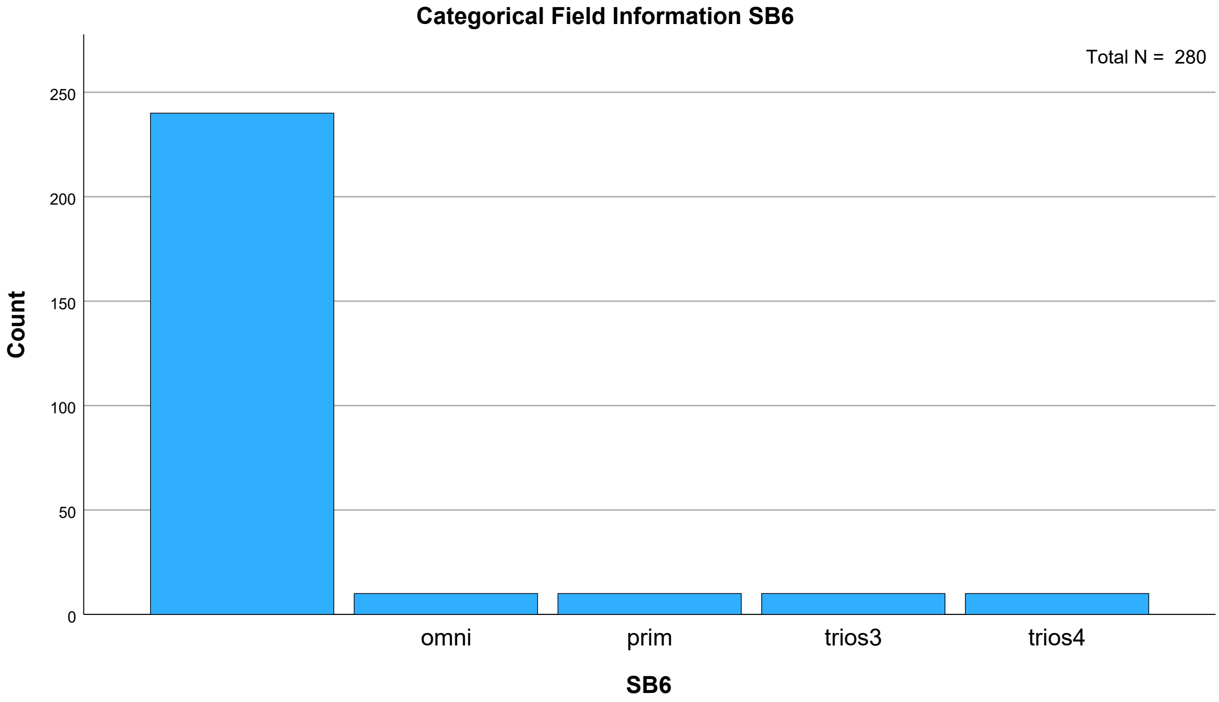
Categorical Field Information SB6
Total N = 280
Count
0
50
100
150
200
250
omni
prim
trios3
trios4
SB6

Nonparametric Tests

Notes

Output Created		30-MAY-2023 09:52:21
Comments		
Input	Active Dataset	DataSet2
	Filter	<none>
	Weight	<none>
	Split File	<none>
	N of Rows in Working Data File	280
Syntax		NPTESTS /INDEPENDENT TEST (DisSB7) GROUP (SB7) KRUSKAL_WALLIS (COMPARE=PAIRWISE) /MISSING SCOPE=ANALYSIS USERMISSING=EXCLUDE /CRITERIA ALPHA=0.05 CILEVEL=95.
Resources	Processor Time	00:00:00.84
	Elapsed Time	00:00:00.74

Hypothesis Test Summary

	Null Hypothesis	Test	Sig.[a,b]
1	The distribution of DisSB7 is the same across categories of SB7.	Independent-Samples Kruskal-Wallis Test	<.001

Hypothesis Test Summary

	Decision
1	Reject the null hypothesis.

a. The significance level is .050.

b. Asymptotic significance is displayed.

Independent-Samples Kruskal-Wallis Test

DisSB7 across SB7

Independent-Samples Kruskal-Wallis Test Summary

Total N	40
Test Statistic	21.165[a]
Degree Of Freedom	3
Asymptotic Sig.(2-sided test)	<.001

a. The test statistic is adjusted for ties.

Independent-Samples Kruskal-Wallis Test

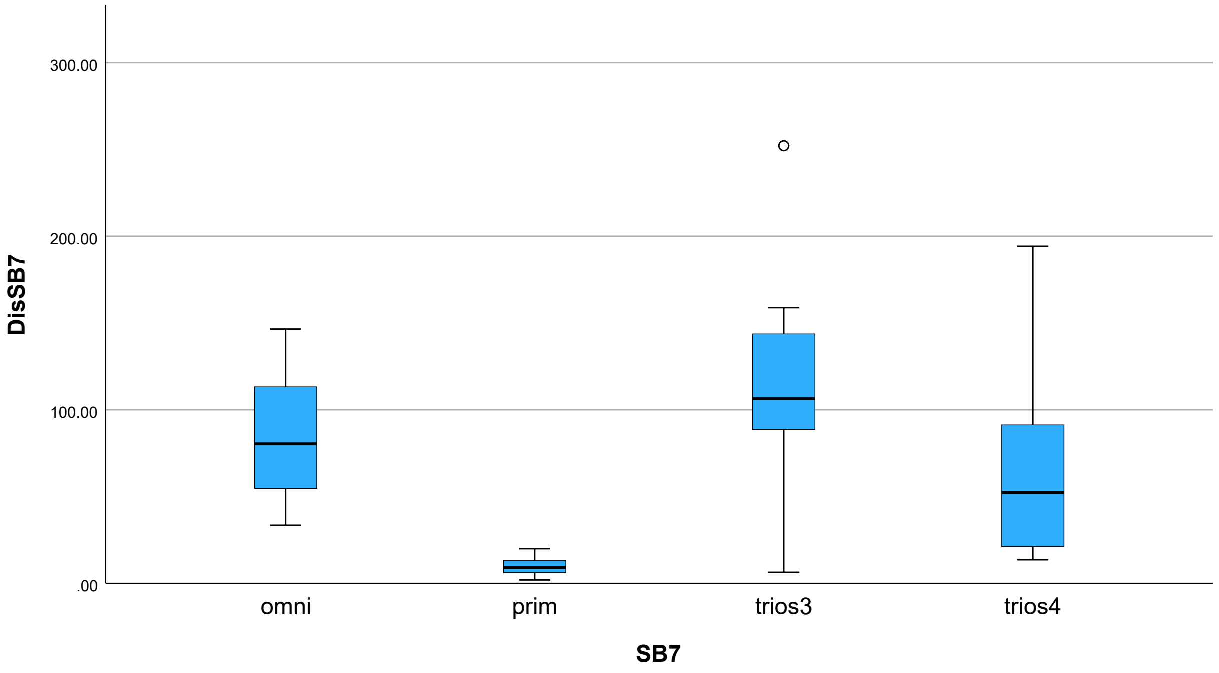

Pairwise Comparisons of SB7

Sample 1-Sample 2	Test Statistic	Std. Error	Std. Test Statistic	Sig.	Adj. Sig.[a]
prim-trios4	-15.100	5.228	-2.888	.004	.023
prim-omni	19.300	5.228	3.692	<.001	.001
prim-trios3	-22.000	5.228	-4.208	<.001	.000
trios4-omni	4.200	5.228	.803	.422	1.000
trios4-trios3	6.900	5.228	1.320	.187	1.000
omni-trios3	-2.700	5.228	-.516	.606	1.000

Each row tests the null hypothesis that the Sample 1 and Sample 2 distributions are the same.
Asymptotic significances (2-sided tests) are displayed. The significance level is .050.

a. Significance values have been adjusted by the Bonferroni correction for multiple tests.

Pairwise Comparisons of SB7

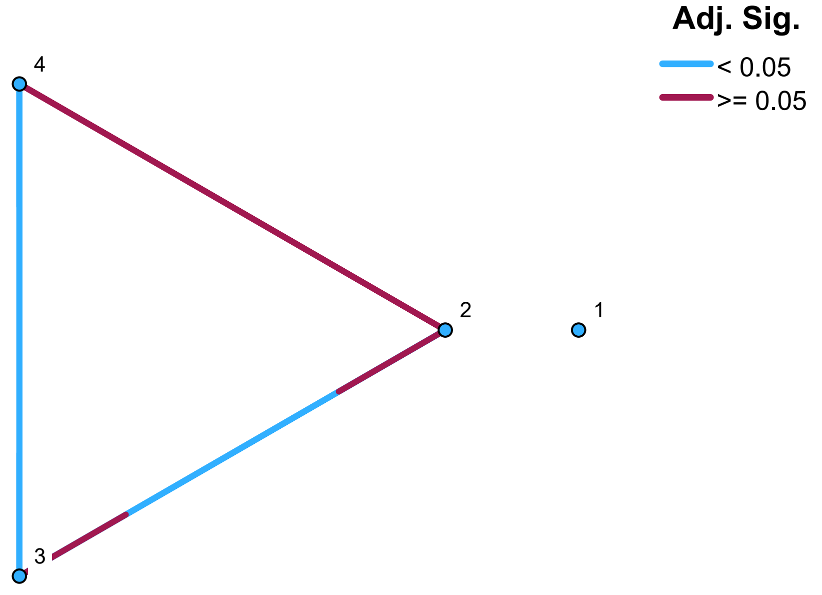

Each node shows the sample average rank of SB7.

Continuous Field Information DisSB7

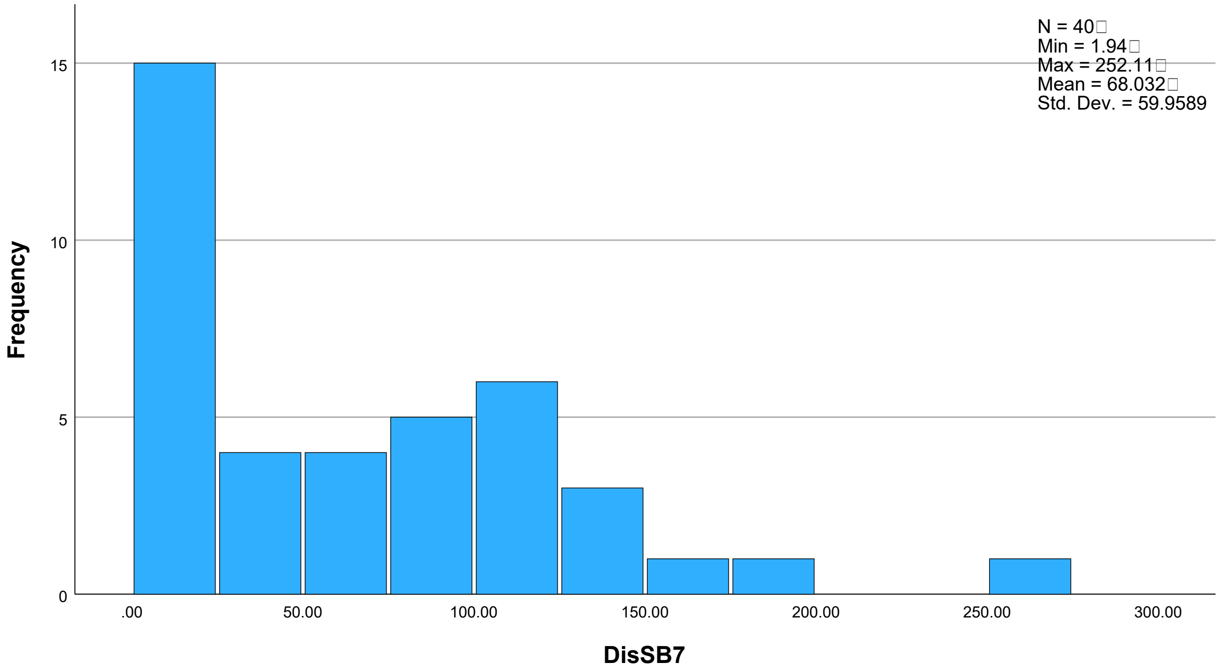

Categorical Field Information SB7

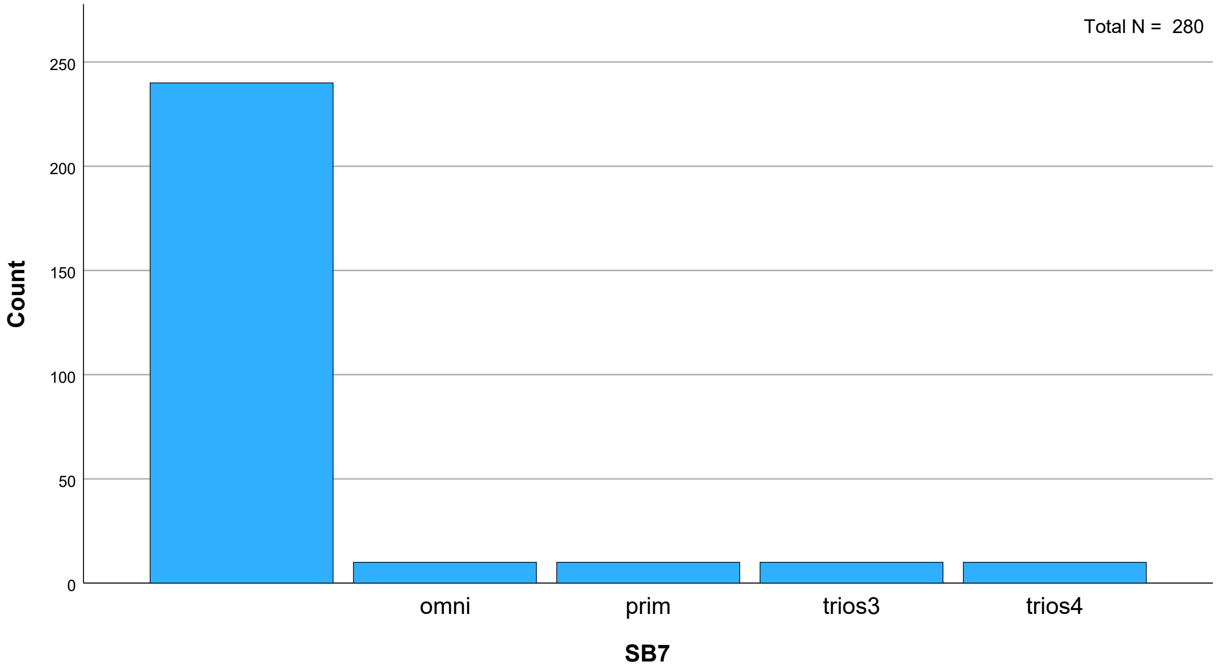

Nonparametric Tests

Notes

Output Created		30-MAY-2023 09:20:03
Comments		
Input	Active Dataset	DataSet2
	Filter	<none>
	Weight	<none>
	Split File	<none>
	N of Rows in Working Data File	280
Syntax		NPTESTS /INDEPENDENT TEST (Trios3Dis) GROUP (Trios3) KRUSKAL_WALLIS (COMPARE=PAIRWISE) /MISSING SCOPE=ANALYSIS USERMISSING=EXCLUDE /CRITERIA ALPHA=0.05 CILEVEL=95.
Resources	Processor Time	00:00:00.81
	Elapsed Time	00:00:00.74

Hypothesis Test Summary

	Null Hypothesis	Test	Sig.[a,b]
1	The distribution of Trios3Dis is the same across categories of Trios3.	Independent-Samples Kruskal-Wallis Test	<.001

Hypothesis Test Summary

	Decision
1	Reject the null hypothesis.

a. The significance level is .050.

b. Asymptotic significance is displayed.

Independent-Samples Kruskal-Wallis Test

Trios3Dis across Trios3

Independent-Samples Kruskal-Wallis Test Summary

Total N	70
Test Statistic	26.230[a]
Degree Of Freedom	6
Asymptotic Sig.(2-sided test)	<.001

a. The test statistic is adjusted for ties.

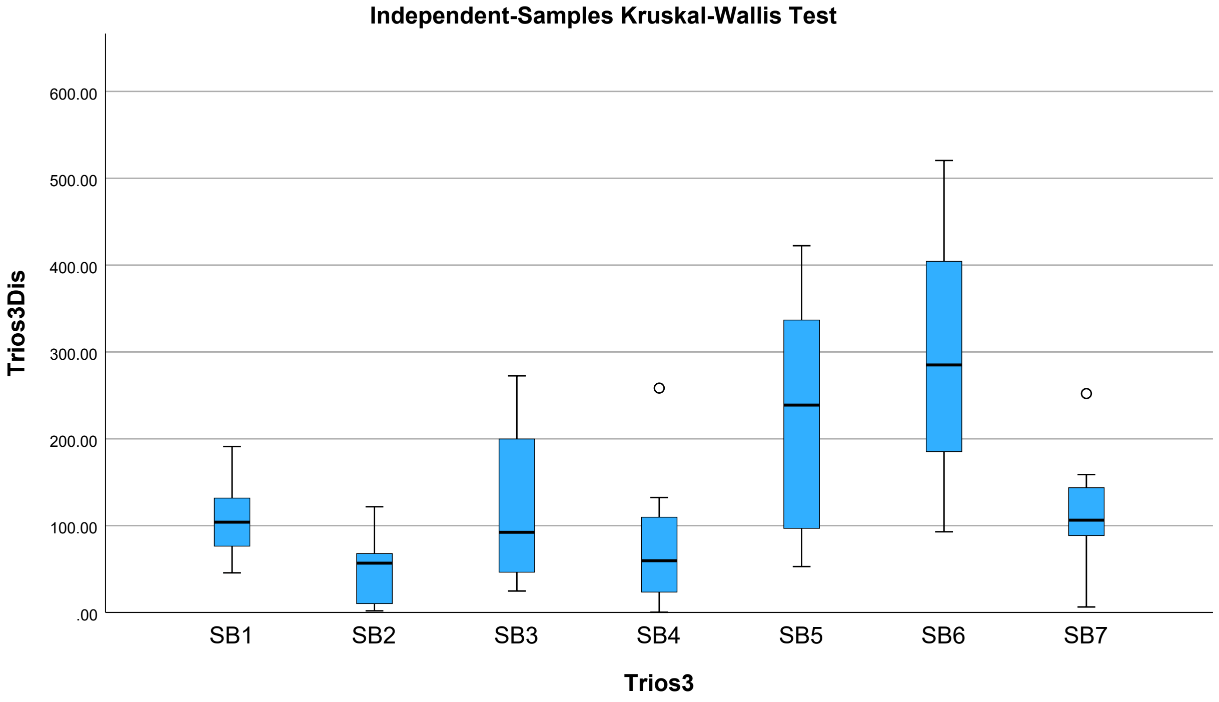

Pairwise Comparisons of Trios3

Sample 1-Sample 2	Test Statistic	Std. Error	Std. Test Statistic	Sig.	Adj. Sig.[a]
SB2-SB4	-4.500	9.101	-.494	.621	1.000
SB2-SB3	-13.400	9.101	-1.472	.141	1.000
SB2-SB7	-14.800	9.101	-1.626	.104	1.000
SB2-SB1	15.500	9.101	1.703	.089	1.000
SB2-SB5	-29.300	9.101	-3.219	.001	.027
SB2-SB6	-38.700	9.101	-4.252	<.001	.000
SB4-SB3	8.900	9.101	.978	.328	1.000
SB4-SB7	-10.300	9.101	-1.132	.258	1.000
SB4-SB1	11.000	9.101	1.209	.227	1.000
SB4-SB5	-24.800	9.101	-2.725	.006	.135
SB4-SB6	-34.200	9.101	-3.758	<.001	.004
SB3-SB7	-1.400	9.101	-.154	.878	1.000
SB3-SB1	2.100	9.101	.231	.818	1.000
SB3-SB5	-15.900	9.101	-1.747	.081	1.000
SB3-SB6	-25.300	9.101	-2.780	.005	.114
SB7-SB1	.700	9.101	.077	.939	1.000
SB7-SB5	14.500	9.101	1.593	.111	1.000
SB7-SB6	23.900	9.101	2.626	.009	.181
SB1-SB5	-13.800	9.101	-1.516	.129	1.000
SB1-SB6	-23.200	9.101	-2.549	.011	.227
SB5-SB6	-9.400	9.101	-1.033	.302	1.000

Each row tests the null hypothesis that the Sample 1 and Sample 2 distributions are the same.
Asymptotic significances (2-sided tests) are displayed. The significance level is .050.

a. Significance values have been adjusted by the Bonferroni correction for multiple tests.

Pairwise Comparisons of Trios3

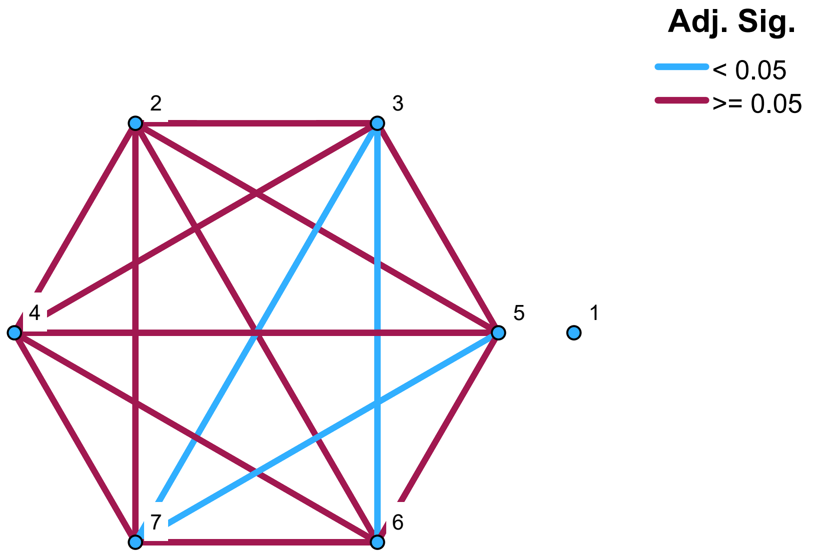

Each node shows the
sample average rank of
Trios3.

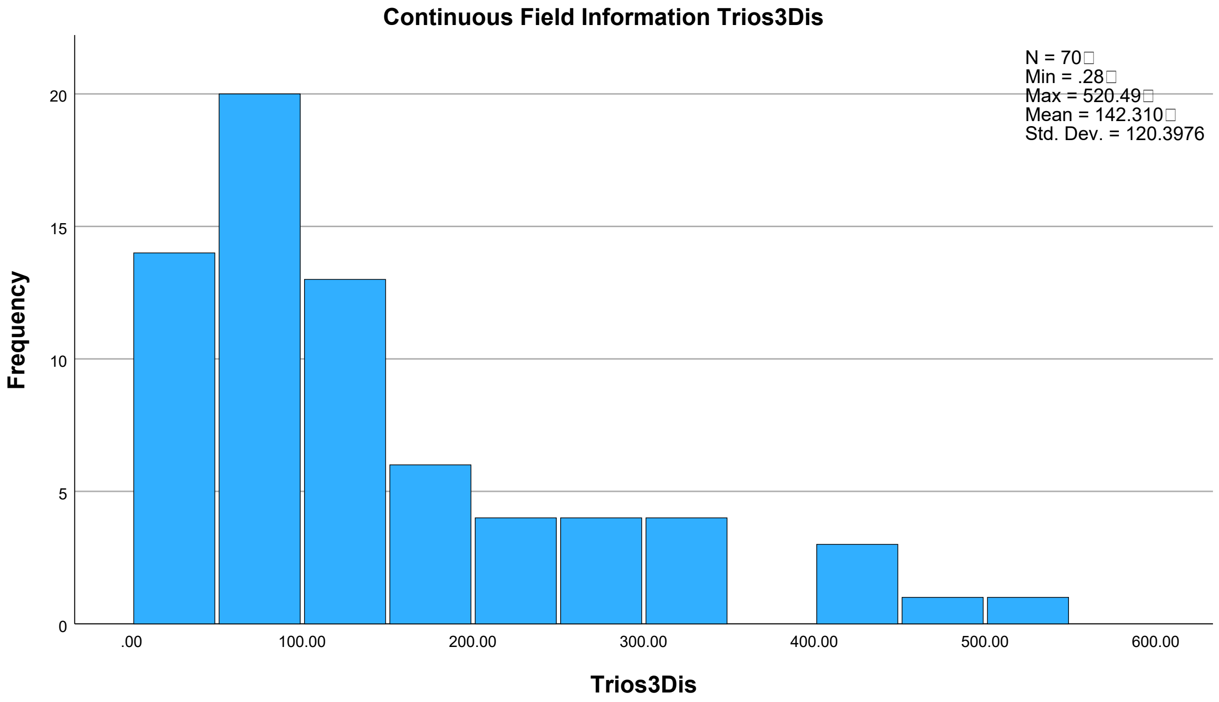
Continuous Field Information Trios3Dis
N = 70
Min = .28
Max = 520.49
Mean = 142.310
Std. Dev. = 120.3976
Frequency
0
5
10
15
20
.00
100.00
200.00
300.00
400.00
500.00
600.00
Trios3Dis

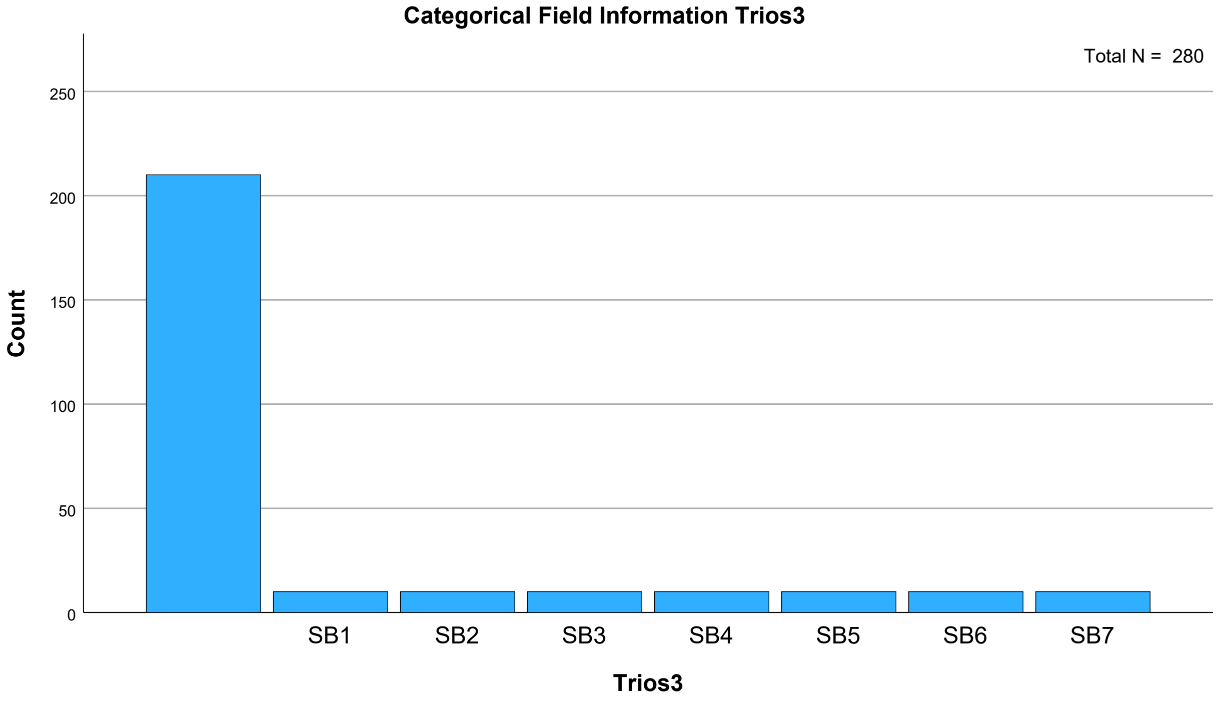
Categorical Field Information Trios3
Total N = 280
Count
0
50
100
150
200
250
SB1
SB2
SB3
SB4
SB5
SB6
SB7
Trios3

Nonparametric Tests

Notes

Output Created		30-MAY-2023 09:21:38
Comments		
Input	Active Dataset	DataSet2
	Filter	<none>
	Weight	<none>
	Split File	<none>
	N of Rows in Working Data File	280
Syntax		NPTESTS /INDEPENDENT TEST (Trios4Dis) GROUP (Trios4) KRUSKAL_WALLIS (COMPARE=PAIRWISE) /MISSING SCOPE=ANALYSIS USERMISSING=EXCLUDE /CRITERIA ALPHA=0.05 CILEVEL=95.
Resources	Processor Time	00:00:00.75
	Elapsed Time	00:00:00.70

Hypothesis Test Summary

	Null Hypothesis	Test	Sig.[a,b]
1	The distribution of Trios4Dis is the same across categories of Trios4.	Independent-Samples Kruskal-Wallis Test	<.001

Hypothesis Test Summary

	Decision
1	Reject the null hypothesis.

a. The significance level is .050.

b. Asymptotic significance is displayed.

Independent-Samples Kruskal-Wallis Test

Trios4Dis across Trios4

Independent-Samples Kruskal-Wallis Test Summary

Total N	70
Test Statistic	30.705[a]
Degree Of Freedom	6
Asymptotic Sig.(2-sided test)	<.001

a. The test statistic is adjusted for ties.

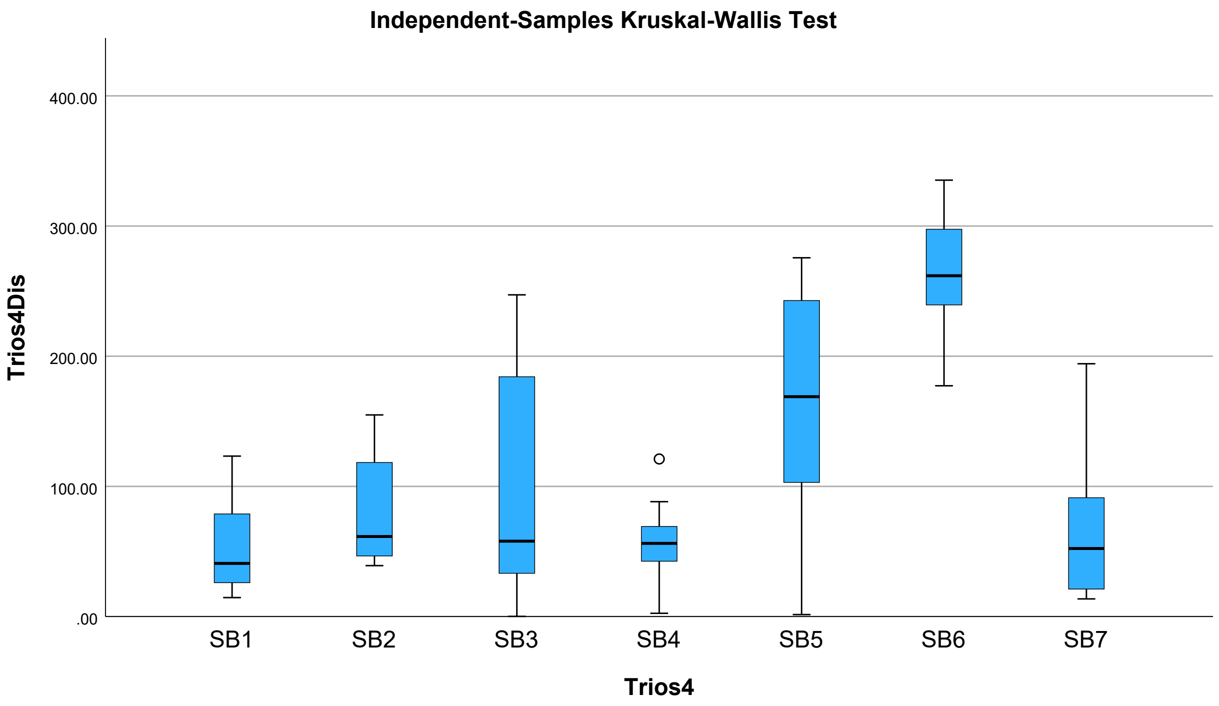

Pairwise Comparisons of Trios4

Sample 1-Sample 2	Test Statistic	Std. Error	Std. Test Statistic	Sig.	Adj. Sig.[a]
SB1-SB4	-.800	9.101	-.088	.930	1.000
SB1-SB7	-2.100	9.101	-.231	.818	1.000
SB1-SB3	-6.600	9.101	-.725	.468	1.000
SB1-SB2	-8.600	9.101	-.945	.345	1.000
SB1-SB5	-23.200	9.101	-2.549	.011	.227
SB1-SB6	-39.200	9.101	-4.307	<.001	.000
SB4-SB7	-1.300	9.101	-.143	.886	1.000
SB4-SB3	5.800	9.101	.637	.524	1.000
SB4-SB2	7.800	9.101	.857	.391	1.000
SB4-SB5	-22.400	9.101	-2.461	.014	.291
SB4-SB6	-38.400	9.101	-4.219	<.001	.001
SB7-SB3	4.500	9.101	.494	.621	1.000
SB7-SB2	6.500	9.101	.714	.475	1.000
SB7-SB5	21.100	9.101	2.318	.020	.429
SB7-SB6	37.100	9.101	4.076	<.001	.001
SB3-SB2	2.000	9.101	.220	.826	1.000
SB3-SB5	-16.600	9.101	-1.824	.068	1.000
SB3-SB6	-32.600	9.101	-3.582	<.001	.007
SB2-SB5	-14.600	9.101	-1.604	.109	1.000
SB2-SB6	-30.600	9.101	-3.362	<.001	.016
SB5-SB6	-16.000	9.101	-1.758	.079	1.000

Each row tests the null hypothesis that the Sample 1 and Sample 2 distributions are the same.
Asymptotic significances (2-sided tests) are displayed. The significance level is .050.

a. Significance values have been adjusted by the Bonferroni correction for multiple tests.

Pairwise Comparisons of Trios4

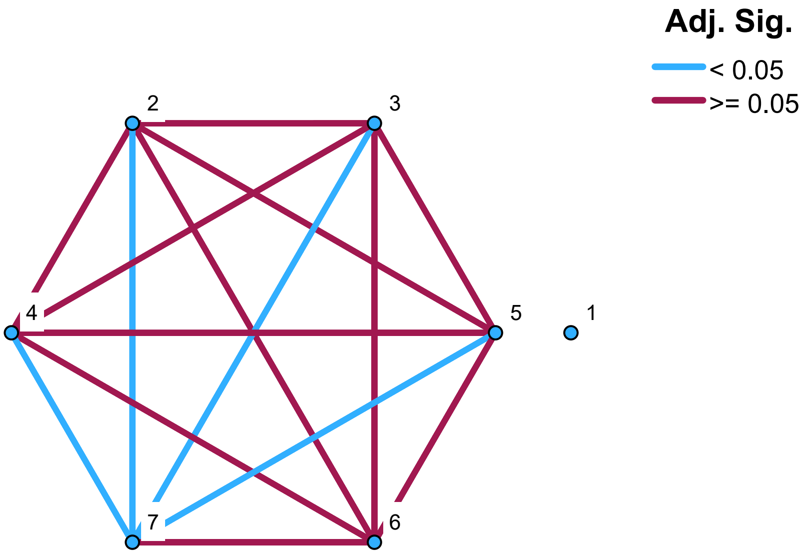

Each node shows the sample average rank of Trios4.

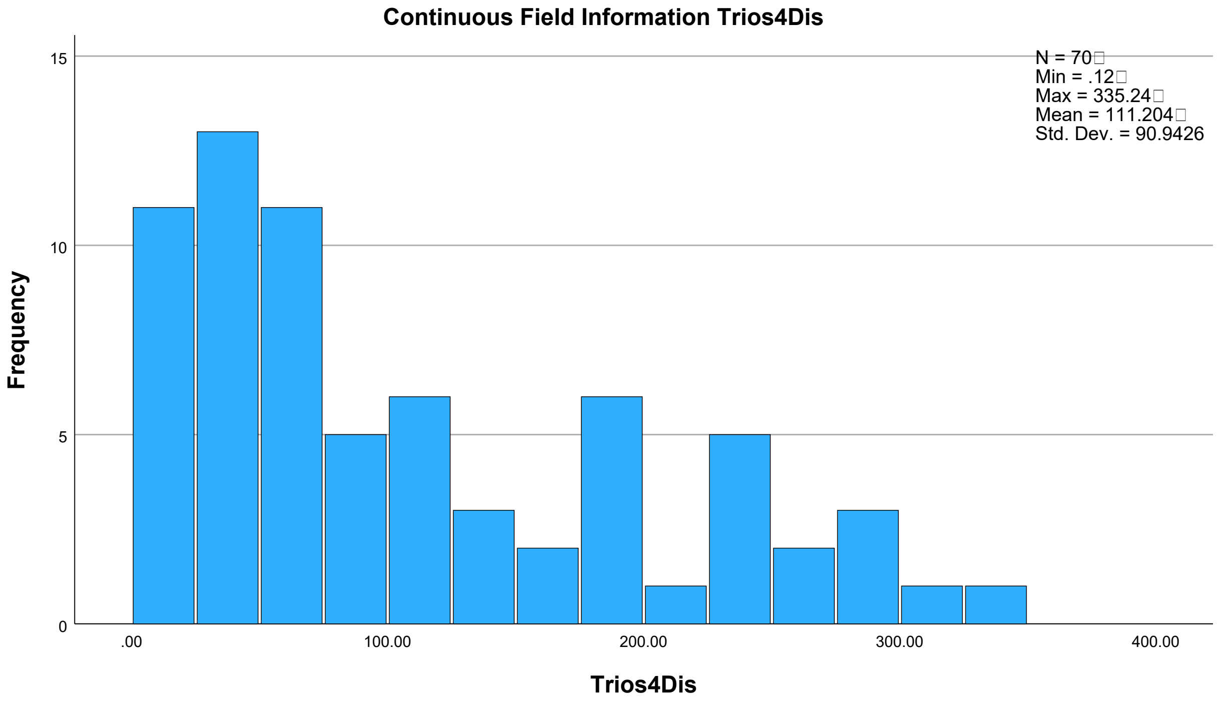
Continuous Field Information Trios4Dis
N = 70
Min = .12
Max = 335.24
Mean = 111.204
Std. Dev. = 90.9426
Frequency
15
10
5
0
.00
100.00
200.00
300.00
400.00
Trios4Dis

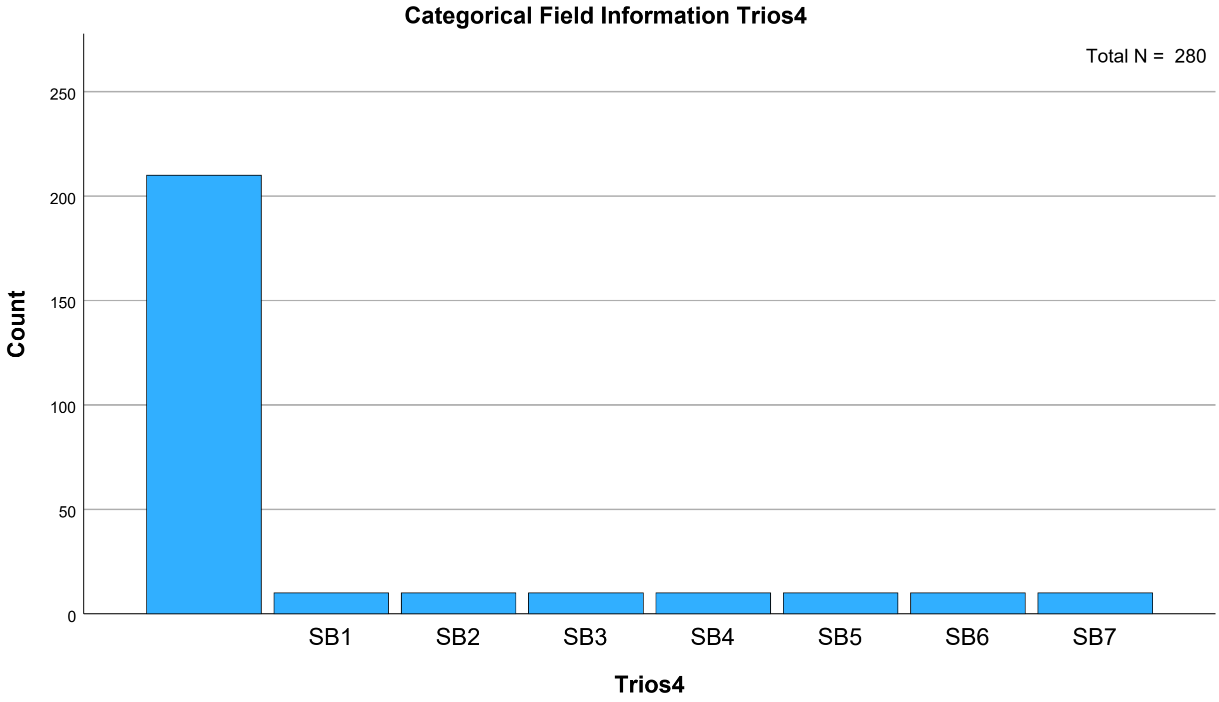
Categorical Field Information Trios4
Total N = 280
Count
250
200
150
100
50
0
SB1
SB2
SB3
SB4
SB5
SB6
SB7
Trios4

Nonparametric Tests

Notes

Output Created		30-MAY-2023 10:52:39
Comments		
Input	Active Dataset	DataSet2
	Filter	<none>
	Weight	<none>
	Split File	<none>
	N of Rows in Working Data File	1680
Syntax		NPTESTS /INDEPENDENT TEST (Trios43D Trios4Ang) GROUP (Trios4) KRUSKAL_WALLIS (COMPARE=PAIRWISE) /MISSING SCOPE=ANALYSIS USERMISSING=EXCLUDE /CRITERIA ALPHA=0.05 CILEVEL=95.
Resources	Processor Time	00:00:01.41
	Elapsed Time	00:00:01.22

Hypothesis Test Summary

	Null Hypothesis	Test	Sig.[a,b]
1	The distribution of Trios43D is the same across categories of Trios4.	Independent-Samples Kruskal-Wallis Test	<.001
2	The distribution of Trios4Ang is the same across categories of Trios4.	Independent-Samples Kruskal-Wallis Test	<.001

Hypothesis Test Summary

	Decision
1	Reject the null hypothesis.
2	Reject the null hypothesis.

a. The significance level is .050.

b. Asymptotic significance is displayed.

Independent-Samples Kruskal-Wallis Test

Trios43D across Trios4

Independent-Samples Kruskal-Wallis Test Summary

Total N	420
Test Statistic	164.482[a]
Degree Of Freedom	6
Asymptotic Sig.(2-sided test)	<.001

a. The test statistic is adjusted for ties.

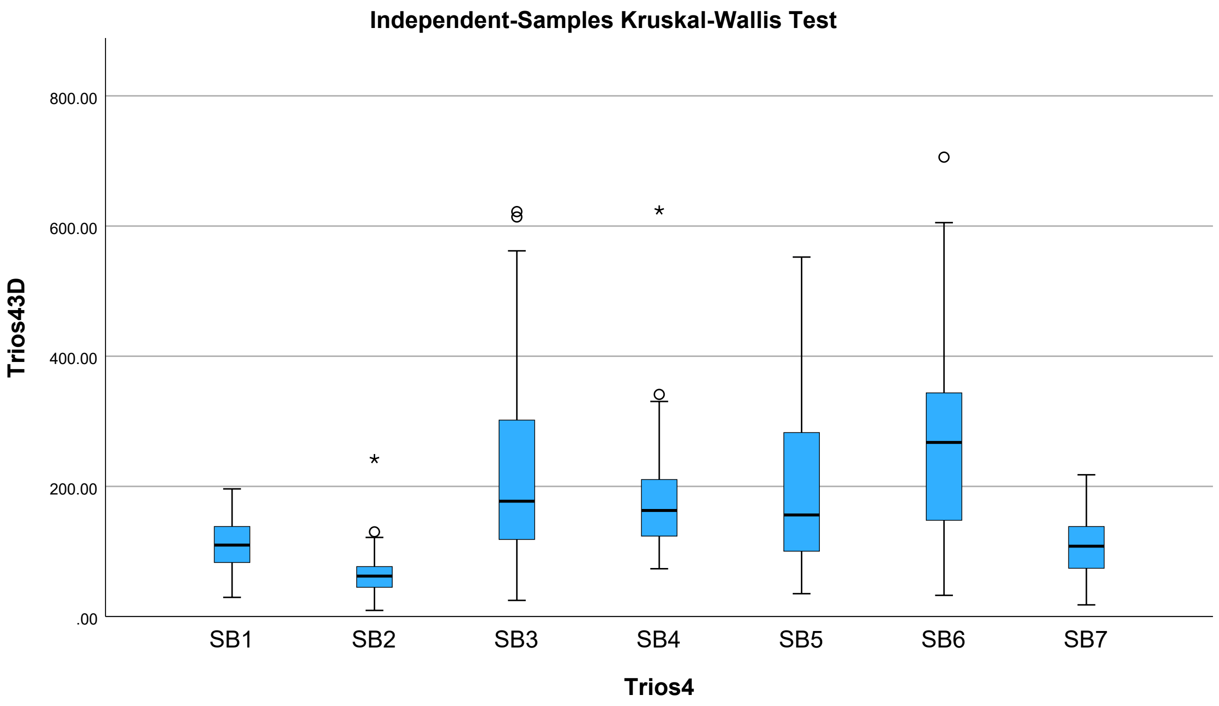

Pairwise Comparisons of Trios4

Sample 1-Sample 2	Test Statistic	Std. Error	Std. Test Statistic	Sig.	Adj. Sig.[a]
SB2-SB7	-89.967	22.162	-4.059	<.001	.001
SB2-SB1	96.817	22.162	4.369	<.001	.000
SB2-SB5	-179.833	22.162	-8.114	<.001	.000
SB2-SB4	-188.217	22.162	-8.493	<.001	.000
SB2-SB3	-198.517	22.162	-8.957	<.001	.000
SB2-SB6	-235.400	22.162	-10.622	<.001	.000
SB7-SB1	6.850	22.162	.309	.757	1.000
SB7-SB5	89.867	22.162	4.055	<.001	.001
SB7-SB4	98.250	22.162	4.433	<.001	.000
SB7-SB3	108.550	22.162	4.898	<.001	.000
SB7-SB6	145.433	22.162	6.562	<.001	.000
SB1-SB5	-83.017	22.162	-3.746	<.001	.004
SB1-SB4	-91.400	22.162	-4.124	<.001	.001
SB1-SB3	-101.700	22.162	-4.589	<.001	.000
SB1-SB6	-138.583	22.162	-6.253	<.001	.000
SB5-SB4	8.383	22.162	.378	.705	1.000
SB5-SB3	18.683	22.162	.843	.399	1.000
SB5-SB6	-55.567	22.162	-2.507	.012	.256
SB4-SB3	10.300	22.162	.465	.642	1.000
SB4-SB6	-47.183	22.162	-2.129	.033	.698
SB3-SB6	-36.883	22.162	-1.664	.096	1.000

Each row tests the null hypothesis that the Sample 1 and Sample 2 distributions are the same.
Asymptotic significances (2-sided tests) are displayed. The significance level is .050.

a. Significance values have been adjusted by the Bonferroni correction for multiple tests.

Pairwise Comparisons of Trios4

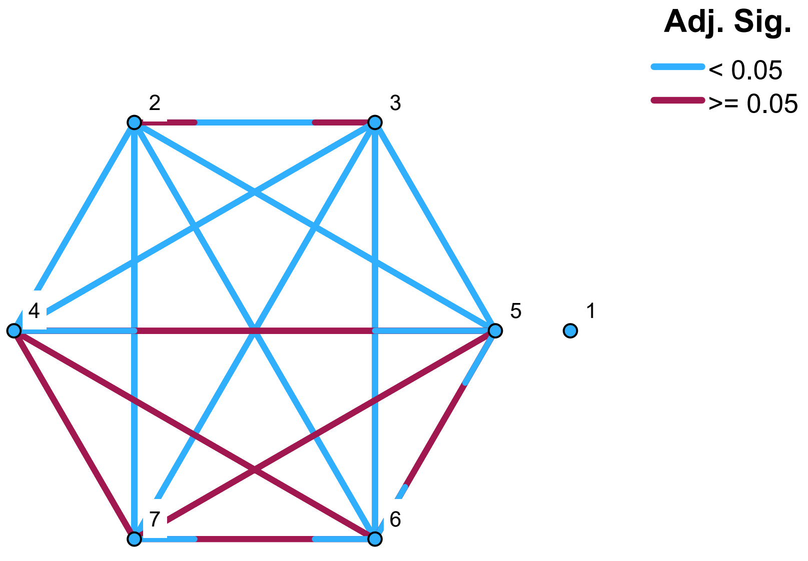

Each node shows the
sample average rank of
Trios4.

Trios4Ang across Trios4

Independent-Samples Kruskal-Wallis Test Summary

Total N	420
Test Statistic	162.141[a]
Degree Of Freedom	6
Asymptotic Sig.(2-sided test)	<.001

a. The test statistic is adjusted for ties.

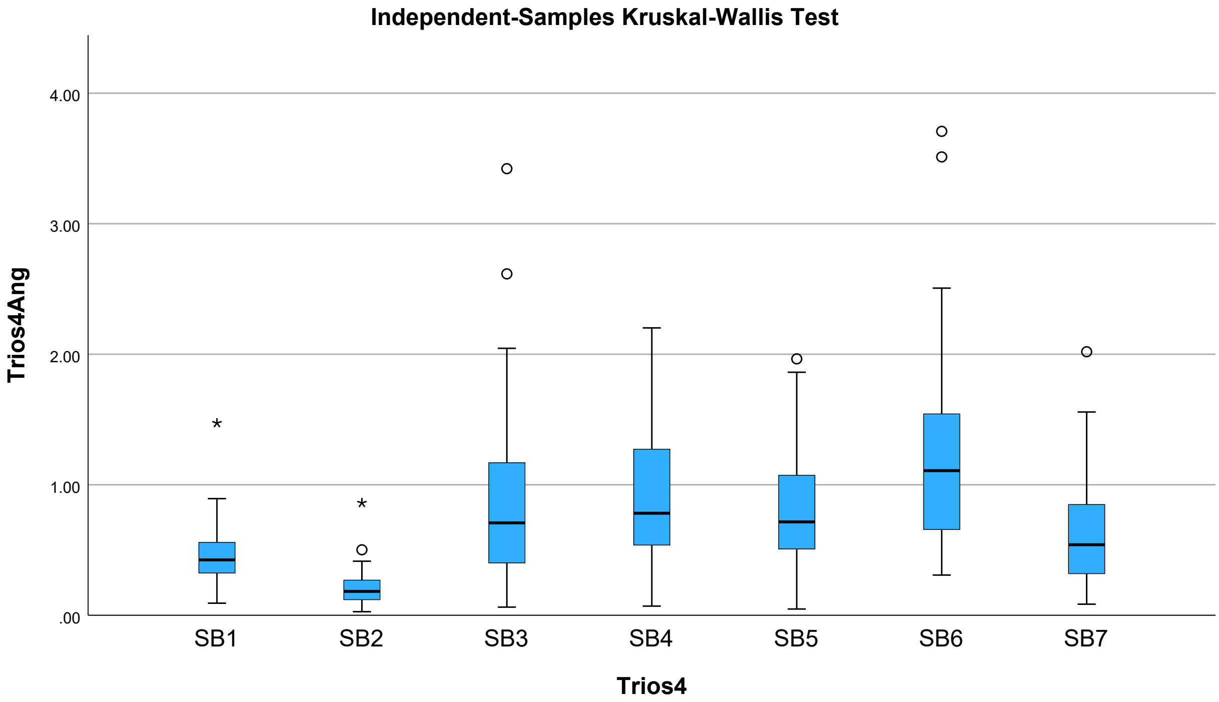

Pairwise Comparisons of Trios4

Sample 1-Sample 2	Test Statistic	Std. Error	Std. Test Statistic	Sig.	Adj. Sig.[a]
SB2-SB1	94.817	22.162	4.278	<.001	.000
SB2-SB7	-134.083	22.162	-6.050	<.001	.000
SB2-SB3	-174.233	22.162	-7.862	<.001	.000
SB2-SB5	-182.367	22.162	-8.229	<.001	.000
SB2-SB4	-200.683	22.162	-9.055	<.001	.000
SB2-SB6	-249.233	22.162	-11.246	<.001	.000
SB1-SB7	-39.267	22.162	-1.772	.076	1.000
SB1-SB3	-79.417	22.162	-3.583	<.001	.007
SB1-SB5	-87.550	22.162	-3.950	<.001	.002
SB1-SB4	-105.867	22.162	-4.777	<.001	.000
SB1-SB6	-154.417	22.162	-6.968	<.001	.000
SB7-SB3	40.150	22.162	1.812	.070	1.000
SB7-SB5	48.283	22.162	2.179	.029	.617
SB7-SB4	66.600	22.162	3.005	.003	.056
SB7-SB6	115.150	22.162	5.196	<.001	.000
SB3-SB5	-8.133	22.162	-.367	.714	1.000
SB3-SB4	-26.450	22.162	-1.193	.233	1.000
SB3-SB6	-75.000	22.162	-3.384	<.001	.015

Pairwise Comparisons of Trios4

Sample 1-Sample 2	Test Statistic	Std. Error	Std. Test Statistic	Sig.	Adj. Sig.[a]
SB5-SB4	18.317	22.162	.826	.409	1.000
SB5-SB6	-66.867	22.162	-3.017	.003	.054
SB4-SB6	-48.550	22.162	-2.191	.028	.598

Each row tests the null hypothesis that the Sample 1 and Sample 2 distributions are the same.
Asymptotic significances (2-sided tests) are displayed. The significance level is .050.

a. Significance values have been adjusted by the Bonferroni correction for multiple tests.

Pairwise Comparisons of Trios4

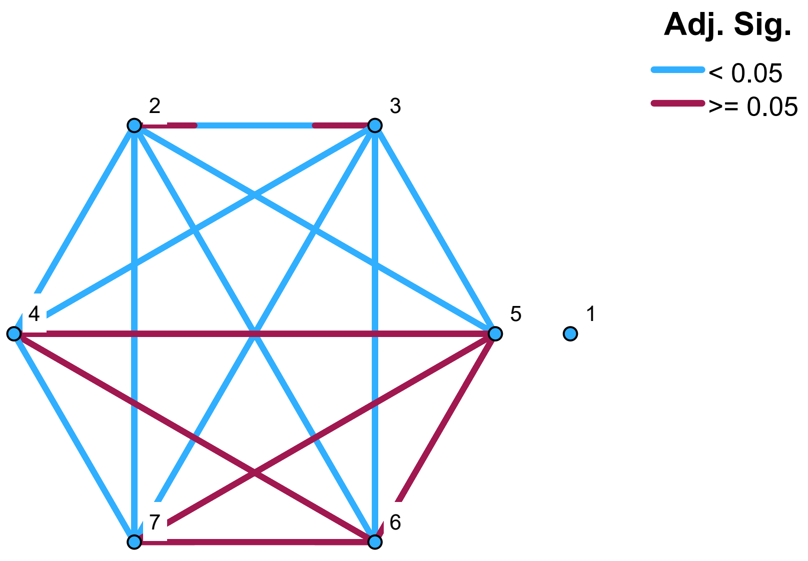

Each node shows the sample average rank of Trios4.

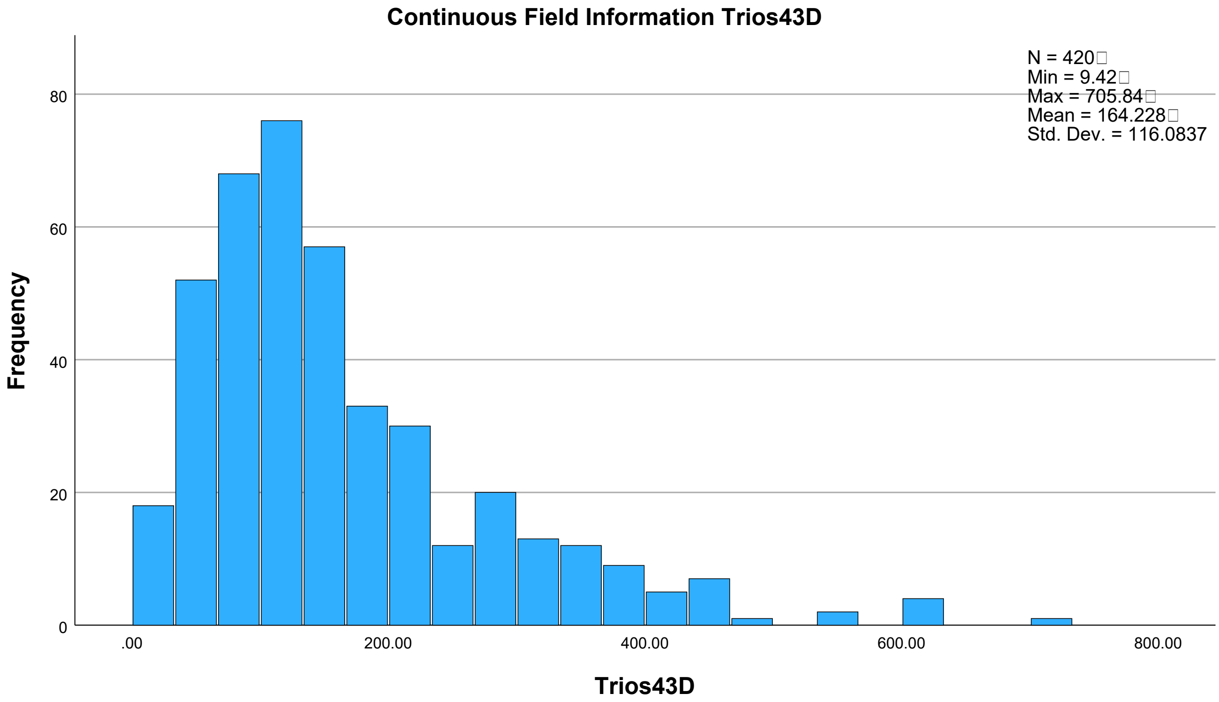
Continuous Field Information Trios43D
N = 420
Min = 9.42
Max = 705.84
Mean = 164.228
Std. Dev. = 116.0837
Frequency
0
20
40
60
80
.00
200.00
400.00
600.00
800.00
Trios43D

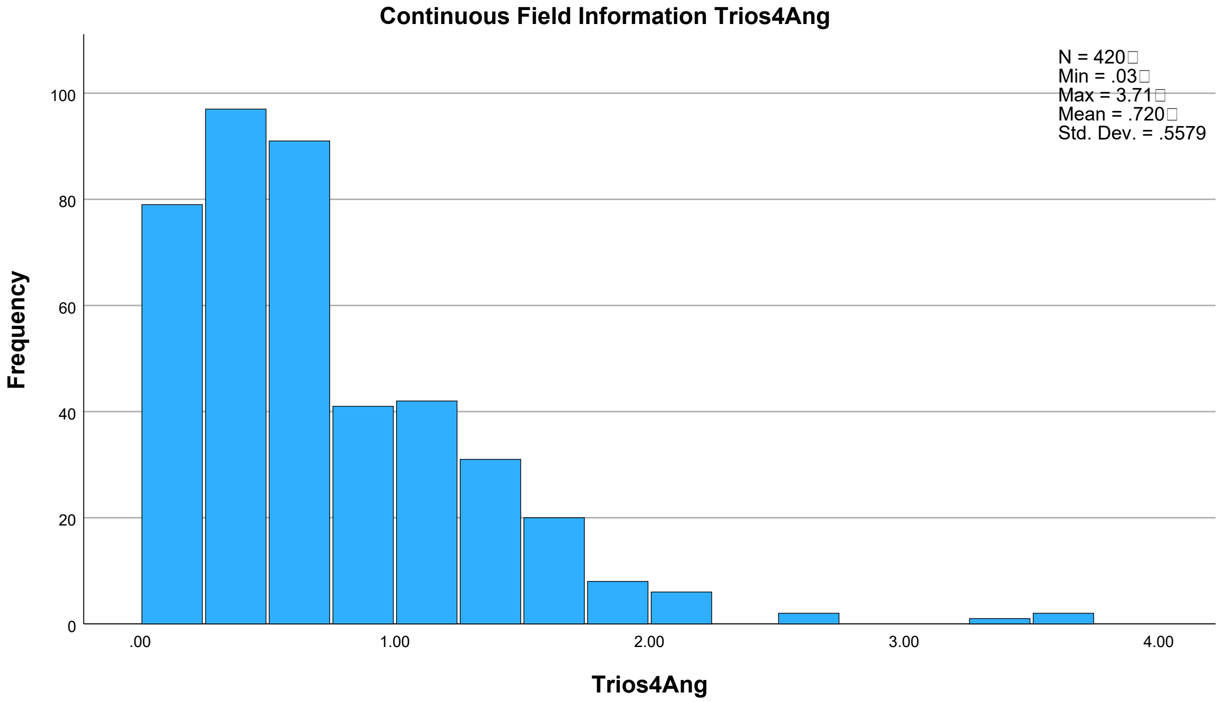
Continuous Field Information Trios4Ang
N = 420
Min = .03
Max = 3.71
Mean = .720
Std. Dev. = .5579
Frequency
0
20
40
60
80
100
.00
1.00
2.00
3.00
4.00
Trios4Ang

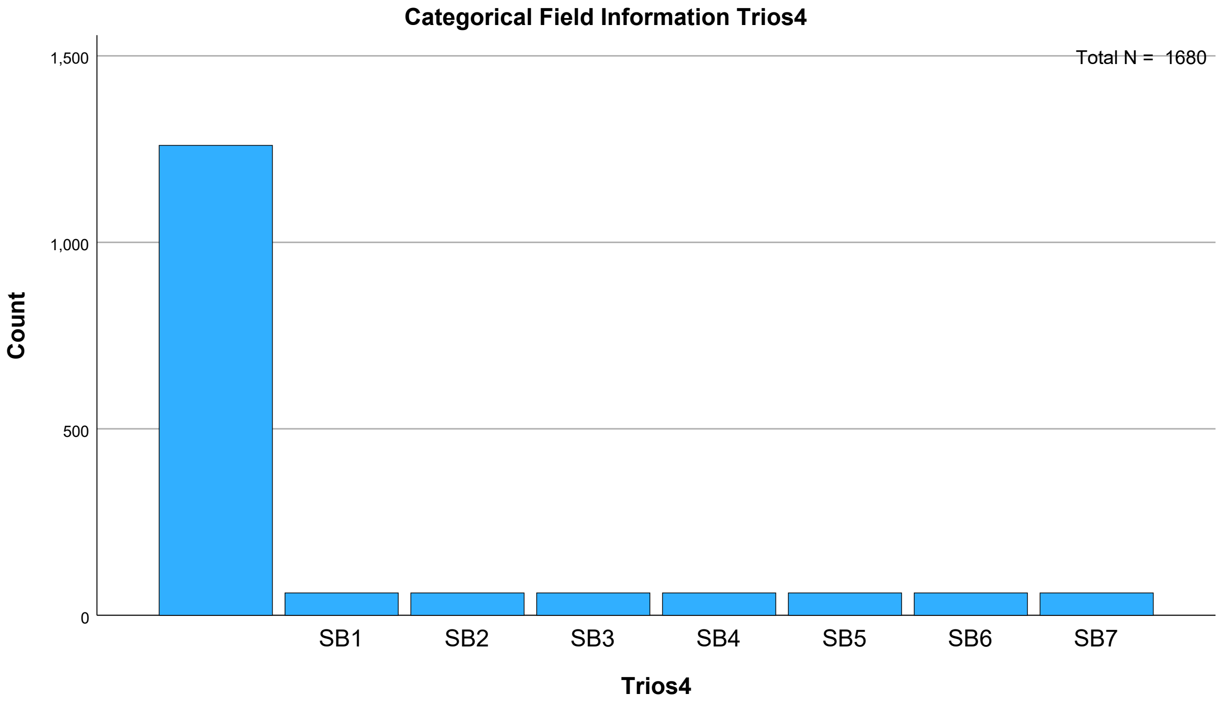
Categorical Field Information Trios4
Total N = 1680
1,500
1,000
500
0
Count
SB1
SB2
SB3
SB4
SB5
SB6
SB7
Trios4

Nonparametric Tests

Notes

Output Created		30-MAY-2023 10:38:07
Comments		
Input	Data	\\Client\C$\SSPS\All3Ddis and ang.sav
	Active Dataset	DataSet2
	Filter	<none>
	Weight	<none>
	Split File	<none>
	N of Rows in Working Data File	1680
Syntax		NPTESTS /INDEPENDENT TEST (Distance_Deviation Angular_Deviation) GROUP (Scanners) KRUSKAL_WALLIS (COMPARE=PAIRWISE) /MISSING SCOPE=ANALYSIS USERMISSING=EXCLUDE /CRITERIA ALPHA=0.05 CILEVEL=95.
Resources	Processor Time	00:00:01.75
	Elapsed Time	00:00:01.56

Hypothesis Test Summary

	Null Hypothesis	Test	Sig.[a,b]
1	The distribution of Distance_Deviation is the same across categories of Scanners.	Independent-Samples Kruskal-Wallis Test	<.001
2	The distribution of Angular_Deviation is the same across categories of Scanners.	Independent-Samples Kruskal-Wallis Test	<.001

Hypothesis Test Summary

	Decision
1	Reject the null hypothesis.
2	Reject the null hypothesis.

a. The significance level is .050.

b. Asymptotic significance is displayed.

Independent-Samples Kruskal-Wallis Test

Distance_Deviation across Scanners

Independent-Samples Kruskal-Wallis Test Summary

Total N	1680
Test Statistic	190.021[a]
Degree Of Freedom	3
Asymptotic Sig.(2-sided test)	<.001

a. The test statistic is adjusted for ties.

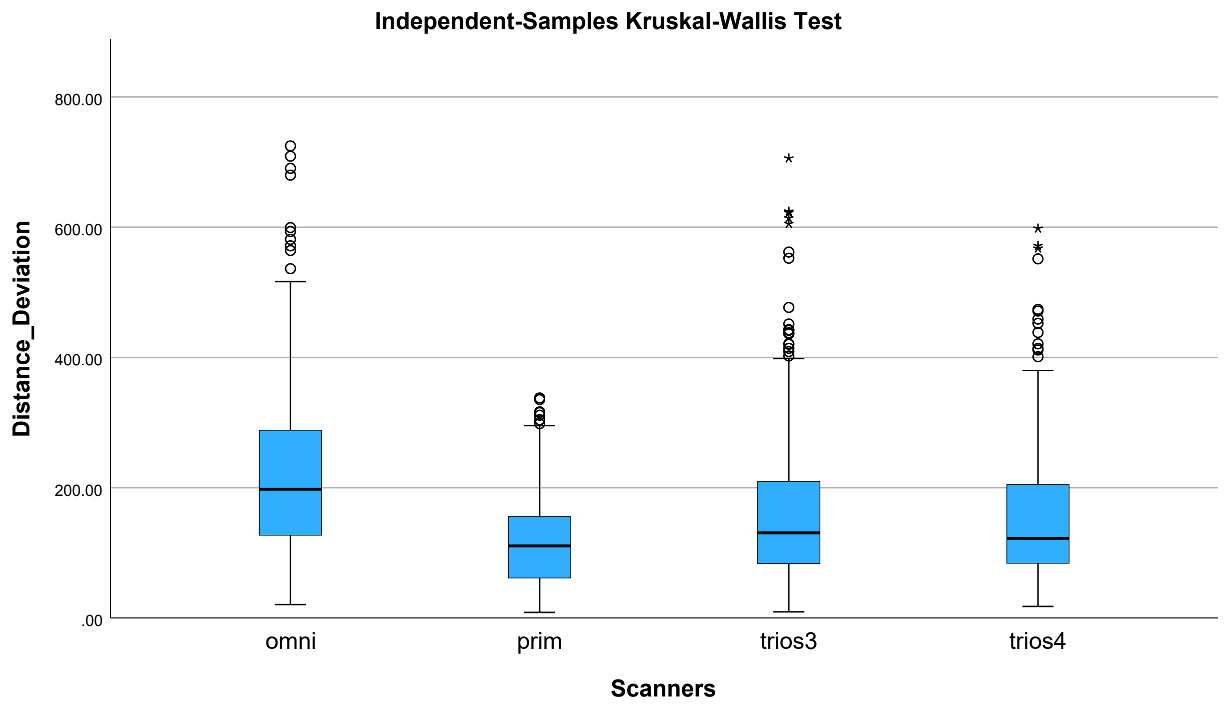

Pairwise Comparisons of Scanners

Sample 1-Sample 2	Test Statistic	Std. Error	Std. Test Statistic	Sig.	Adj. Sig.[a]
prim-trios4	-166.050	33.476	-4.960	<.001	.000
prim-trios3	-193.262	33.476	-5.773	<.001	.000
prim-omni	455.602	33.476	13.610	<.001	.000
trios4-trios3	27.212	33.476	.813	.416	1.000
trios4-omni	289.552	33.476	8.649	<.001	.000
trios3-omni	262.340	33.476	7.837	<.001	.000

Each row tests the null hypothesis that the Sample 1 and Sample 2 distributions are the same.
Asymptotic significances (2-sided tests) are displayed. The significance level is .050.

a. Significance values have been adjusted by the Bonferroni correction for multiple tests.

Pairwise Comparisons of Scanners

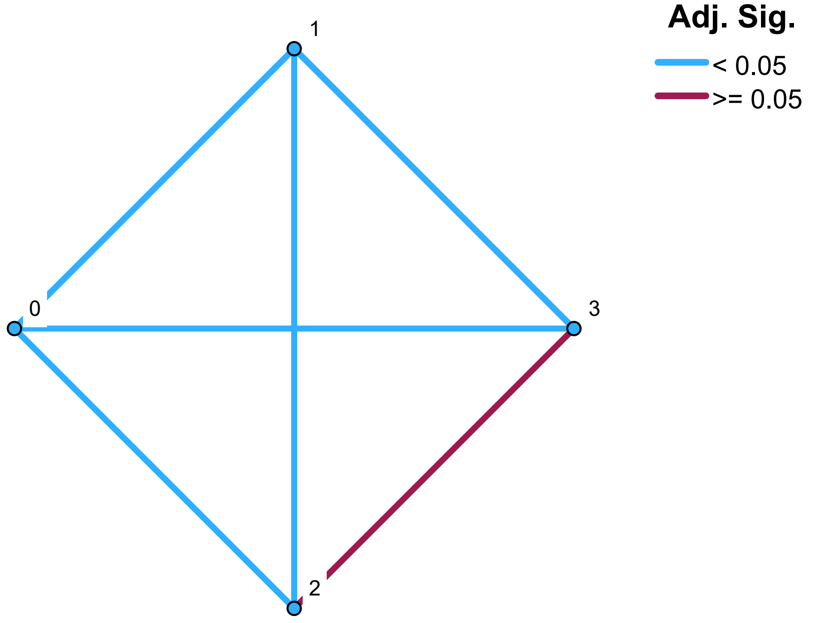

Each node shows the sample average rank of Scanners.

Angular_Deviation across Scanners

Independent-Samples Kruskal-Wallis Test Summary

Total N	1680
Test Statistic	47.568[a]
Degree Of Freedom	3
Asymptotic Sig.(2-sided test)	<.001

a. The test statistic is adjusted for ties.

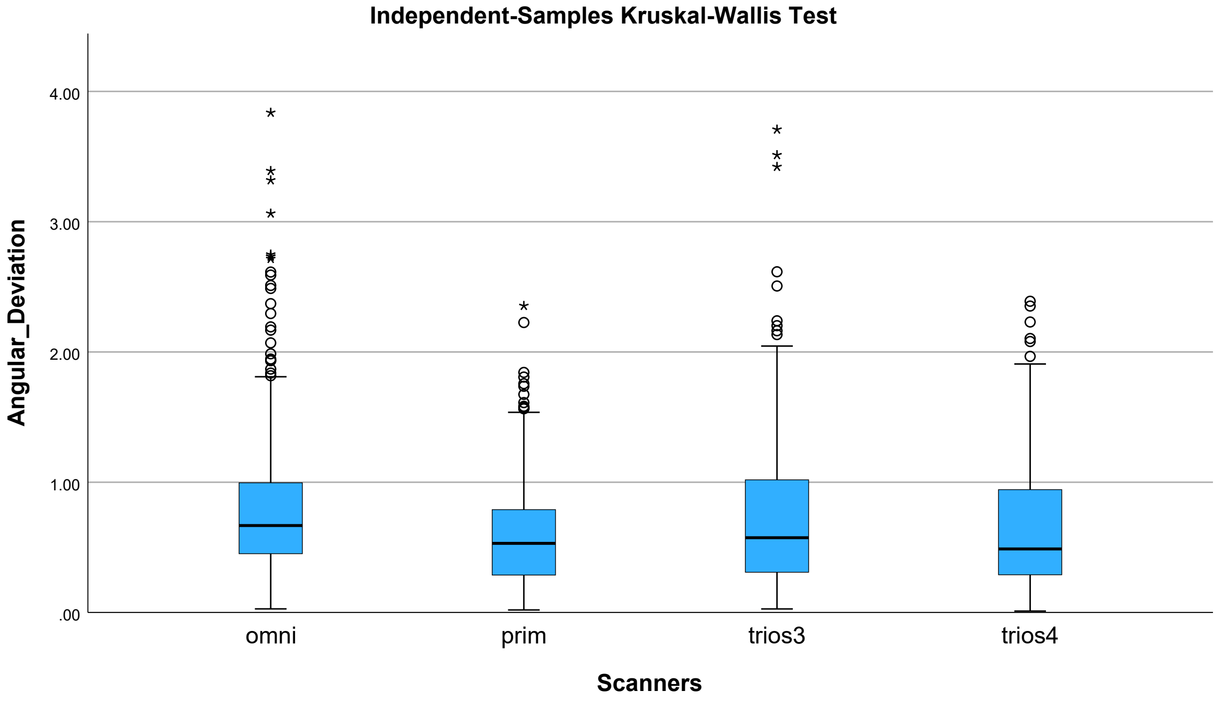

Pairwise Comparisons of Scanners

Sample 1-Sample 2	Test Statistic	Std. Error	Std. Test Statistic	Sig.	Adj. Sig.[a]
prim-trios4	-21.106	33.476	-.630	.528	1.000
prim-trios3	-85.014	33.476	-2.540	.011	.067
prim-omni	209.480	33.476	6.258	<.001	.000
trios4-trios3	63.908	33.476	1.909	.056	.338
trios4-omni	188.374	33.476	5.627	<.001	.000
trios3-omni	124.465	33.476	3.718	<.001	.001

Each row tests the null hypothesis that the Sample 1 and Sample 2 distributions are the same.
Asymptotic significances (2-sided tests) are displayed. The significance level is .050.

a. Significance values have been adjusted by the Bonferroni correction for multiple tests.

Pairwise Comparisons of Scanners

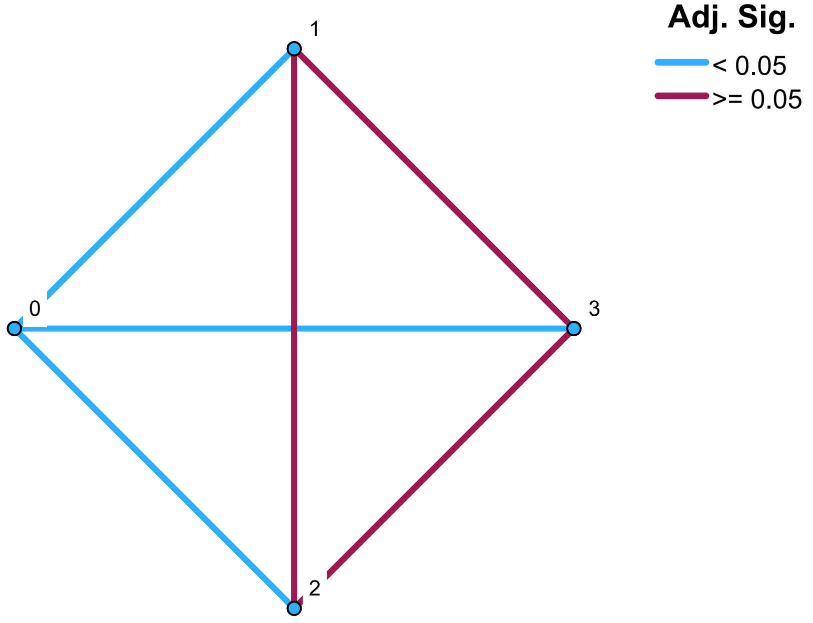

Each node shows the sample average rank of Scanners.

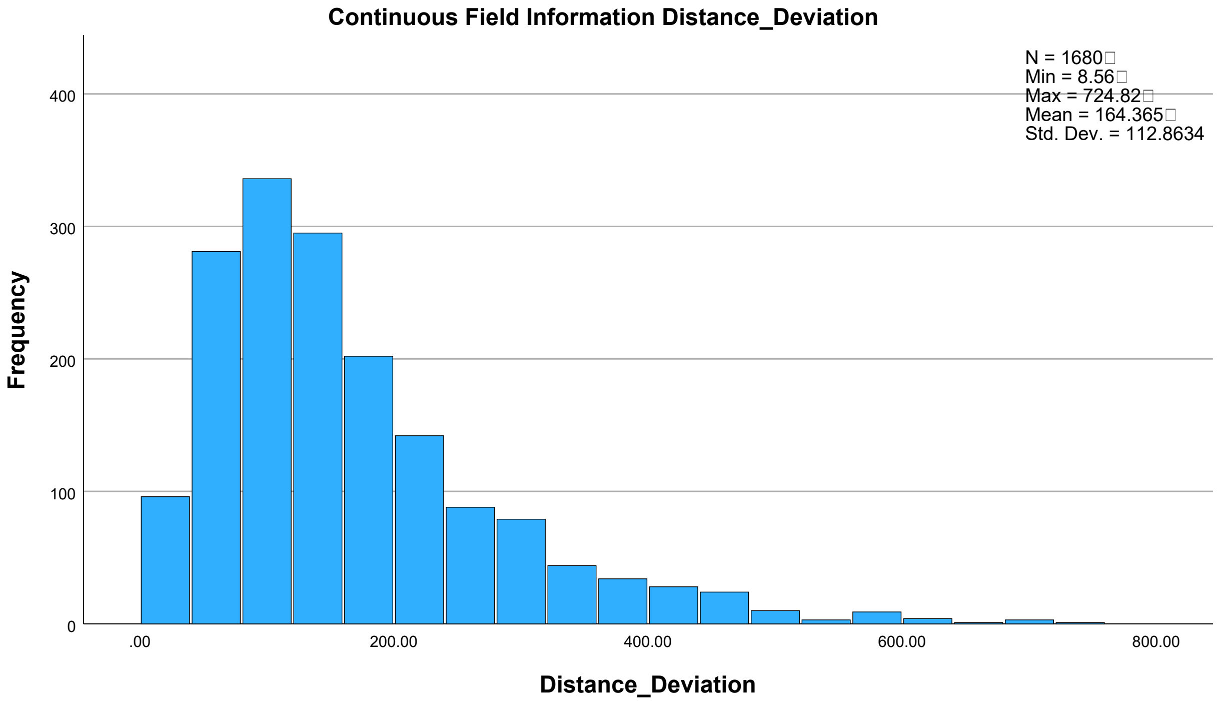
Continuous Field Information Distance_Deviation
N = 1680
Min = 8.56
Max = 724.82
Mean = 164.365
Std. Dev. = 112.8634
Frequency
0
100
200
300
400
.00
200.00
400.00
600.00
800.00
Distance_Deviation

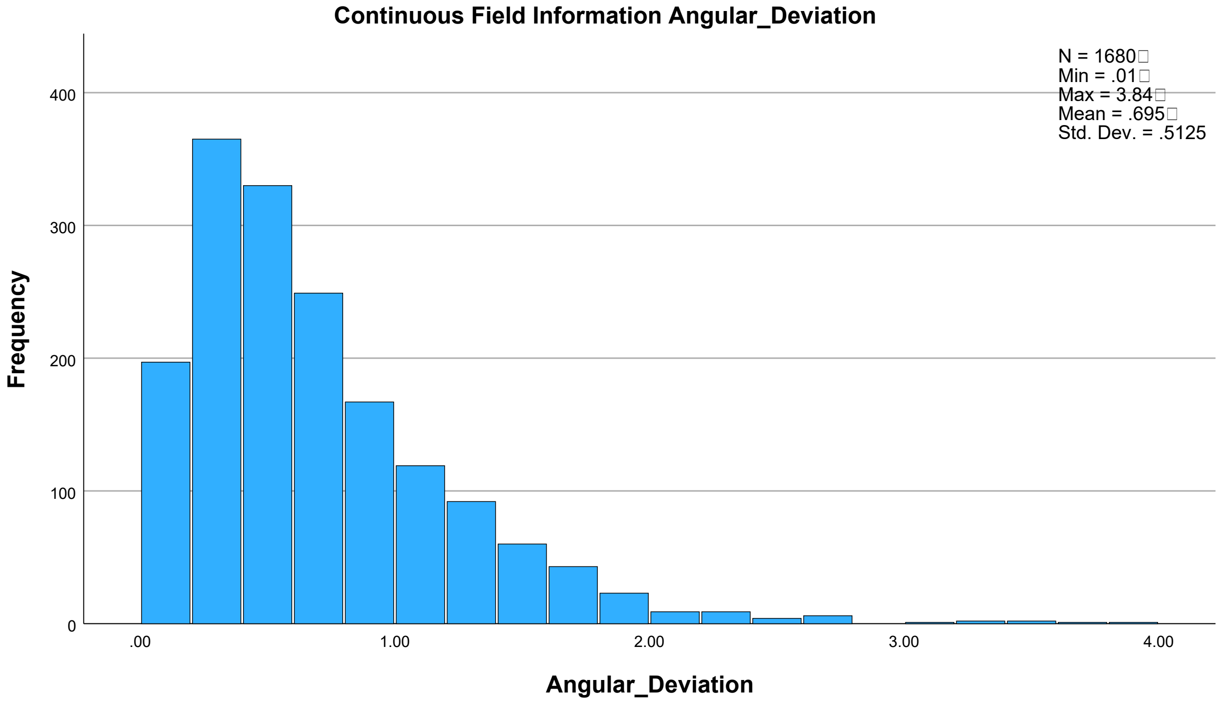
Continuous Field Information Angular_Deviation
N = 1680
Min = .01
Max = 3.84
Mean = .695
Std. Dev. = .5125
Frequency
0
100
200
300
400
.00
1.00
2.00
3.00
4.00
Angular_Deviation

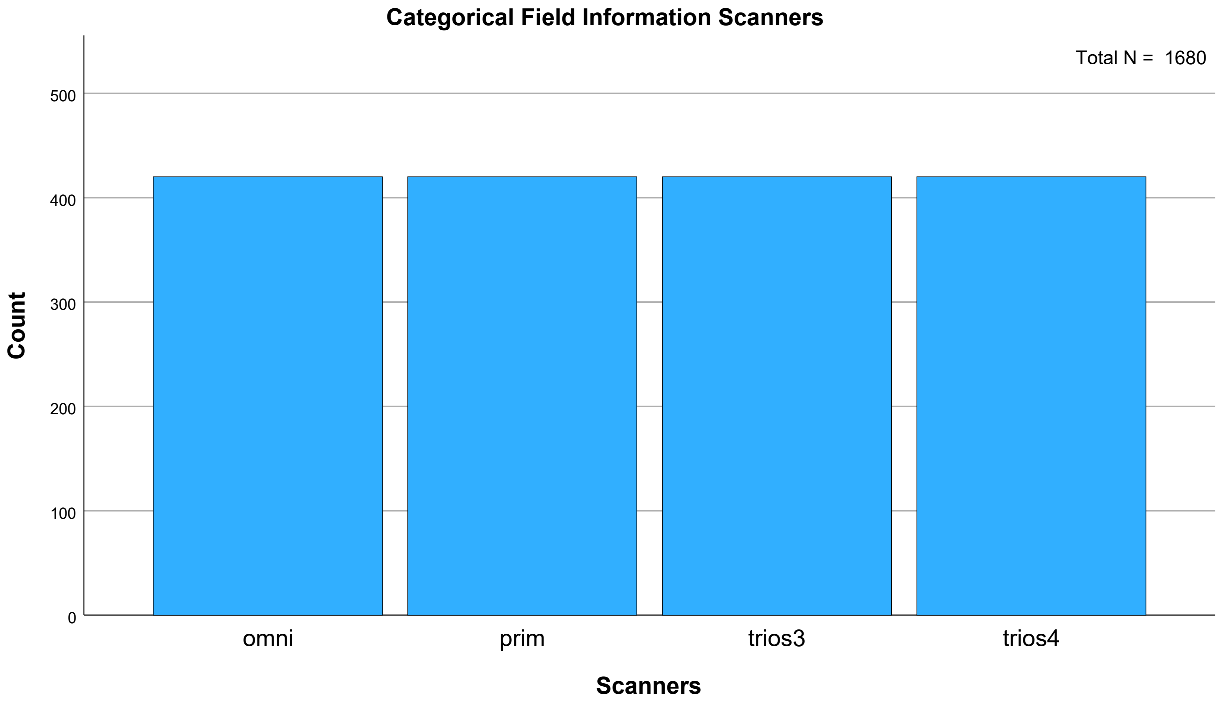
Categorical Field Information Scanners
Total N = 1680
500
400
300
200
100
0
Count
omni
prim
trios3
trios4
Scanners

Nonparametric Tests

Notes

Output Created		30-MAY-2023 10:51:55
Comments		
Input	Active Dataset	DataSet2
	Filter	<none>
	Weight	<none>
	Split File	<none>
	N of Rows in Working Data File	1680
Syntax		NPTESTS /INDEPENDENT TEST (Tios33D Trios3Ang) GROUP (Trios3) KRUSKAL_WALLIS (COMPARE=PAIRWISE) /MISSING SCOPE=ANALYSIS USERMISSING=EXCLUDE /CRITERIA ALPHA=0.05 CILEVEL=95.
Resources	Processor Time	00:00:01.36
	Elapsed Time	00:00:01.22

Hypothesis Test Summary

	Null Hypothesis	Test	Sig.[a,b]
1	The distribution of Tios33D is the same across categories of Trios3.	Independent-Samples Kruskal-Wallis Test	<.001
2	The distribution of Trios3Ang is the same across categories of Trios3.	Independent-Samples Kruskal-Wallis Test	<.001

Hypothesis Test Summary

	Decision
1	Reject the null hypothesis.
2	Reject the null hypothesis.

a. The significance level is .050.

b. Asymptotic significance is displayed.

Independent-Samples Kruskal-Wallis Test

Tios33D across Trios3

Independent-Samples Kruskal-Wallis Test Summary

Total N	420
Test Statistic	164.482[a]
Degree Of Freedom	6
Asymptotic Sig.(2-sided test)	<.001

a. The test statistic is adjusted for ties.

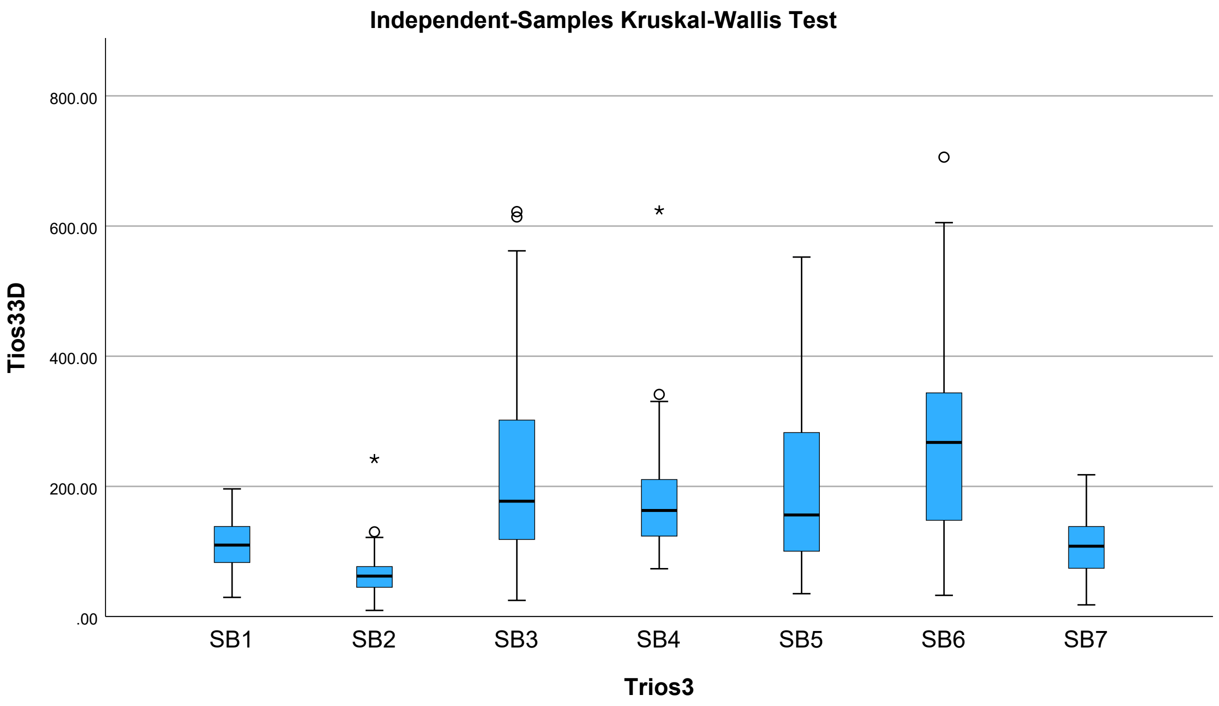

Pairwise Comparisons of Trios3

Sample 1-Sample 2	Test Statistic	Std. Error	Std. Test Statistic	Sig.	Adj. Sig.[a]
SB2-SB7	-89.967	22.162	-4.059	<.001	.001
SB2-SB1	96.817	22.162	4.369	<.001	.000
SB2-SB5	-179.833	22.162	-8.114	<.001	.000
SB2-SB4	-188.217	22.162	-8.493	<.001	.000
SB2-SB3	-198.517	22.162	-8.957	<.001	.000
SB2-SB6	-235.400	22.162	-10.622	<.001	.000
SB7-SB1	6.850	22.162	.309	.757	1.000
SB7-SB5	89.867	22.162	4.055	<.001	.001
SB7-SB4	98.250	22.162	4.433	<.001	.000
SB7-SB3	108.550	22.162	4.898	<.001	.000
SB7-SB6	145.433	22.162	6.562	<.001	.000
SB1-SB5	-83.017	22.162	-3.746	<.001	.004
SB1-SB4	-91.400	22.162	-4.124	<.001	.001
SB1-SB3	-101.700	22.162	-4.589	<.001	.000
SB1-SB6	-138.583	22.162	-6.253	<.001	.000
SB5-SB4	8.383	22.162	.378	.705	1.000
SB5-SB3	18.683	22.162	.843	.399	1.000
SB5-SB6	-55.567	22.162	-2.507	.012	.256
SB4-SB3	10.300	22.162	.465	.642	1.000
SB4-SB6	-47.183	22.162	-2.129	.033	.698
SB3-SB6	-36.883	22.162	-1.664	.096	1.000

Each row tests the null hypothesis that the Sample 1 and Sample 2 distributions are the same.
Asymptotic significances (2-sided tests) are displayed. The significance level is .050.

a. Significance values have been adjusted by the Bonferroni correction for multiple tests.

Pairwise Comparisons of Trios3

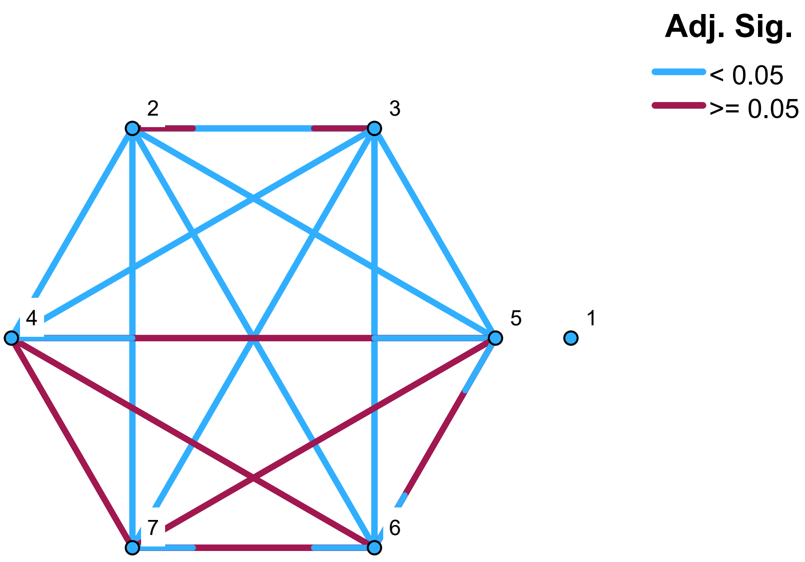

Each node shows the sample average rank of Trios3.

Trios3Ang across Trios3

Independent-Samples Kruskal-Wallis Test Summary

Total N	420
Test Statistic	162.141[a]
Degree Of Freedom	6
Asymptotic Sig.(2-sided test)	<.001

a. The test statistic is adjusted for ties.

Independent-Samples Kruskal-Wallis Test

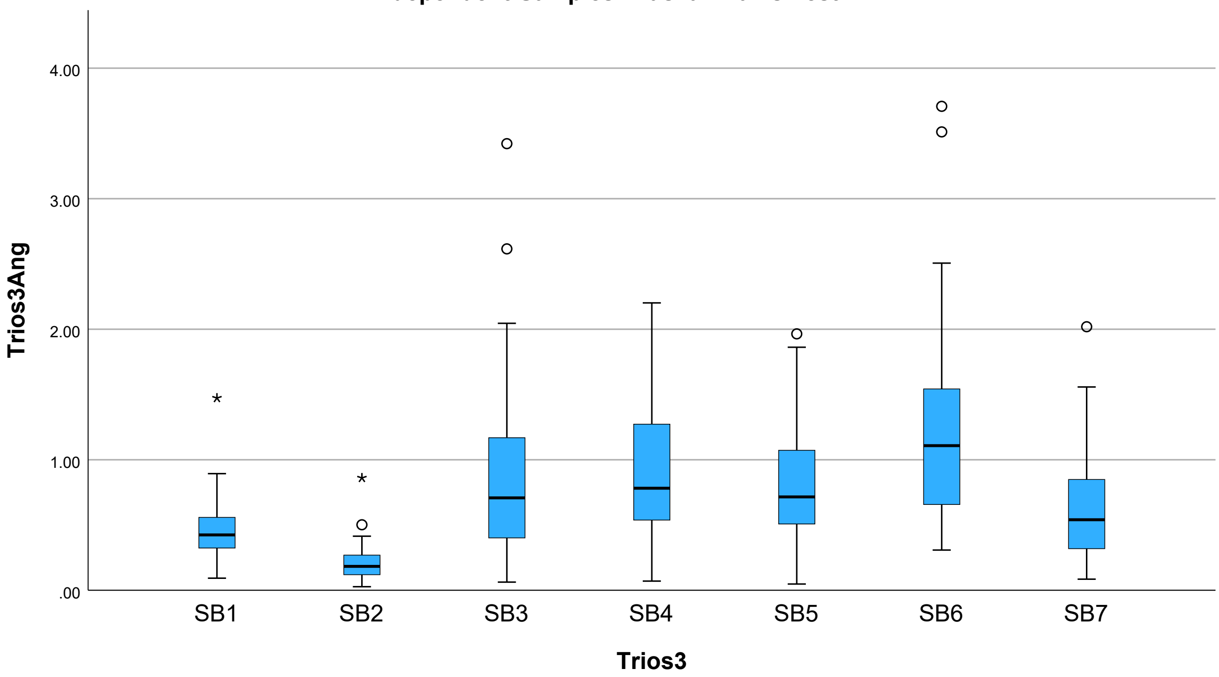

Pairwise Comparisons of Trios3

Sample 1-Sample 2	Test Statistic	Std. Error	Std. Test Statistic	Sig.	Adj. Sig.[a]
SB2-SB1	94.817	22.162	4.278	<.001	.000
SB2-SB7	-134.083	22.162	-6.050	<.001	.000
SB2-SB3	-174.233	22.162	-7.862	<.001	.000
SB2-SB5	-182.367	22.162	-8.229	<.001	.000
SB2-SB4	-200.683	22.162	-9.055	<.001	.000
SB2-SB6	-249.233	22.162	-11.246	<.001	.000
SB1-SB7	-39.267	22.162	-1.772	.076	1.000
SB1-SB3	-79.417	22.162	-3.583	<.001	.007
SB1-SB5	-87.550	22.162	-3.950	<.001	.002
SB1-SB4	-105.867	22.162	-4.777	<.001	.000
SB1-SB6	-154.417	22.162	-6.968	<.001	.000
SB7-SB3	40.150	22.162	1.812	.070	1.000
SB7-SB5	48.283	22.162	2.179	.029	.617
SB7-SB4	66.600	22.162	3.005	.003	.056
SB7-SB6	115.150	22.162	5.196	<.001	.000
SB3-SB5	-8.133	22.162	-.367	.714	1.000
SB3-SB4	-26.450	22.162	-1.193	.233	1.000
SB3-SB6	-75.000	22.162	-3.384	<.001	.015

Pairwise Comparisons of Trios3

Sample 1-Sample 2	Test Statistic	Std. Error	Std. Test Statistic	Sig.	Adj. Sig.[a]
SB5-SB4	18.317	22.162	.826	.409	1.000
SB5-SB6	-66.867	22.162	-3.017	.003	.054
SB4-SB6	-48.550	22.162	-2.191	.028	.598

Each row tests the null hypothesis that the Sample 1 and Sample 2 distributions are the same.
Asymptotic significances (2-sided tests) are displayed. The significance level is .050.

a. Significance values have been adjusted by the Bonferroni correction for multiple tests.

Pairwise Comparisons of Trios3

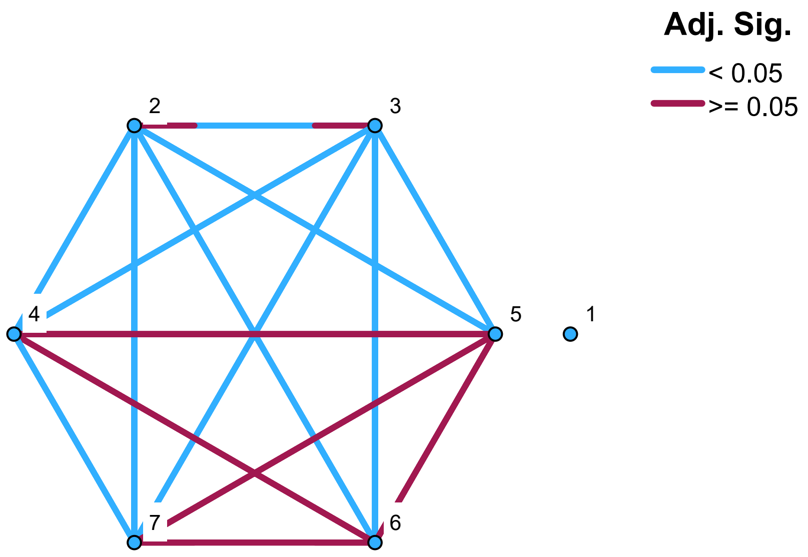

Each node shows the sample average rank of Trios3.

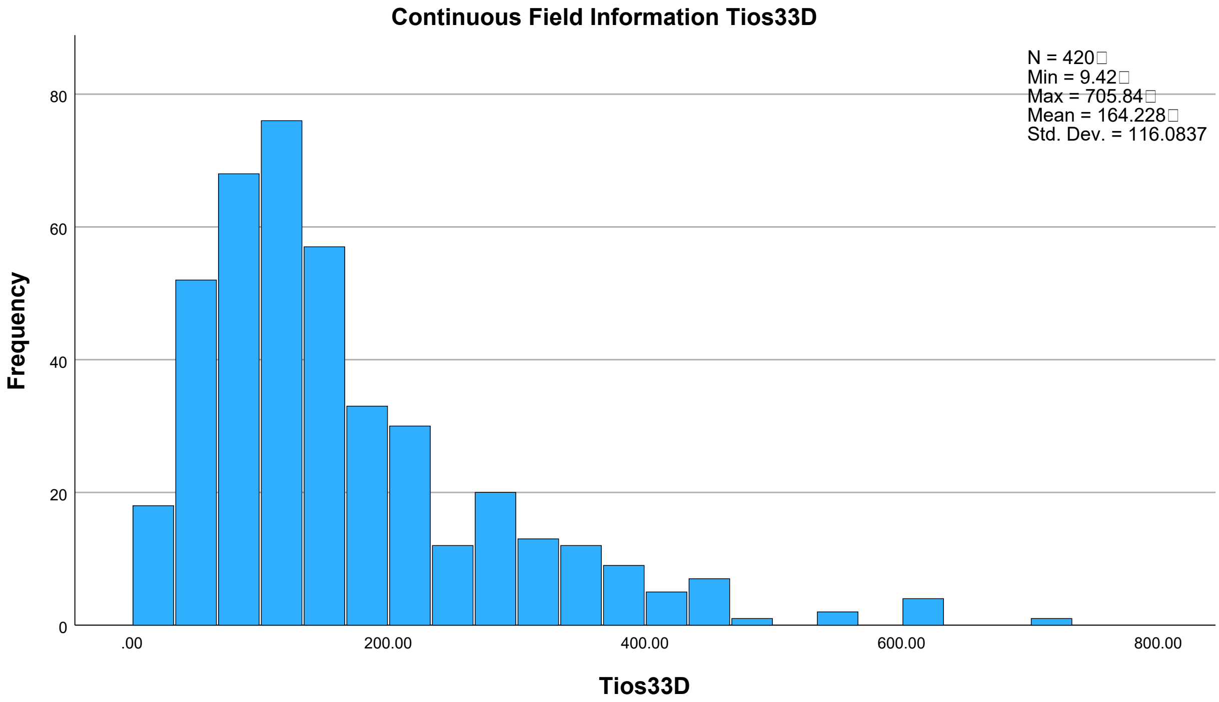
Continuous Field Information Tios33D
N = 420
Min = 9.42
Max = 705.84
Mean = 164.228
Std. Dev. = 116.0837
Frequency
0
20
40
60
80
.00
200.00
400.00
600.00
800.00
Tios33D

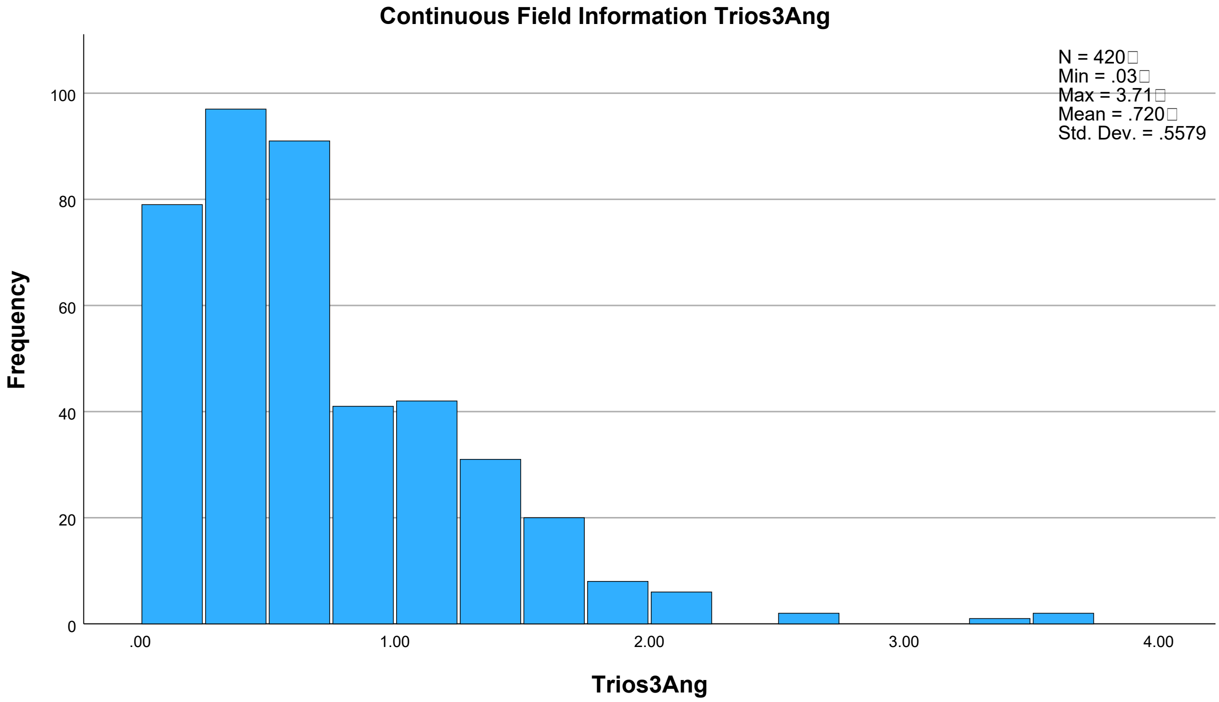
Continuous Field Information Trios3Ang
N = 420
Min = .03
Max = 3.71
Mean = .720
Std. Dev. = .5579
Frequency
0
20
40
60
80
100
.00
1.00
2.00
3.00
4.00
Trios3Ang

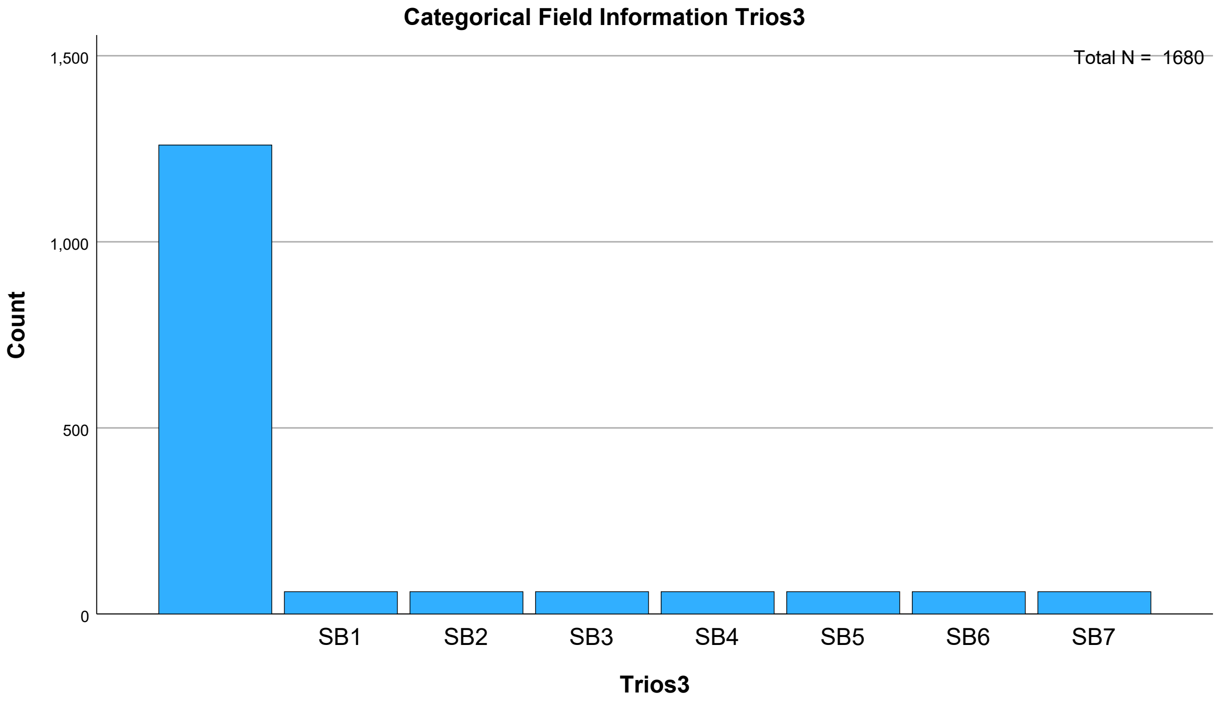
Categorical Field Information Trios3
Total N = 1680
1,500
1,000
500
0
Count
SB1
SB2
SB3
SB4
SB5
SB6
SB7
Trios3

Nonparametric Tests

Notes

Output Created		30-MAY-2023 10:40:12
Comments		
Input	Data	\\Client\C$\SSPS\All3Ddis and ang.sav
	Active Dataset	DataSet2
	Filter	<none>
	Weight	<none>
	Split File	<none>
	N of Rows in Working Data File	1680
Syntax		NPTESTS /INDEPENDENT TEST (Distance_Deviation Angular_Deviation) GROUP (SBs) KRUSKAL_WALLIS (COMPARE=PAIRWISE) /MISSING SCOPE=ANALYSIS USERMISSING=EXCLUDE /CRITERIA ALPHA=0.05 CILEVEL=95.
Resources	Processor Time	00:00:01.31
	Elapsed Time	00:00:01.32

Hypothesis Test Summary

	Null Hypothesis	Test	Sig.[a,b]
1	The distribution of Distance_Deviation is the same across categories of SBs.	Independent-Samples Kruskal-Wallis Test	<.001
2	The distribution of Angular_Deviation is the same across categories of SBs.	Independent-Samples Kruskal-Wallis Test	<.001

Hypothesis Test Summary

	Decision
1	Reject the null hypothesis.
2	Reject the null hypothesis.

a. The significance level is .050.

b. Asymptotic significance is displayed.

Independent-Samples Kruskal-Wallis Test

Distance_Deviation across SBs

Independent-Samples Kruskal-Wallis Test Summary

Total N	1680
Test Statistic	419.920[a]
Degree Of Freedom	6
Asymptotic Sig.(2-sided test)	<.001

a. The test statistic is adjusted for ties.

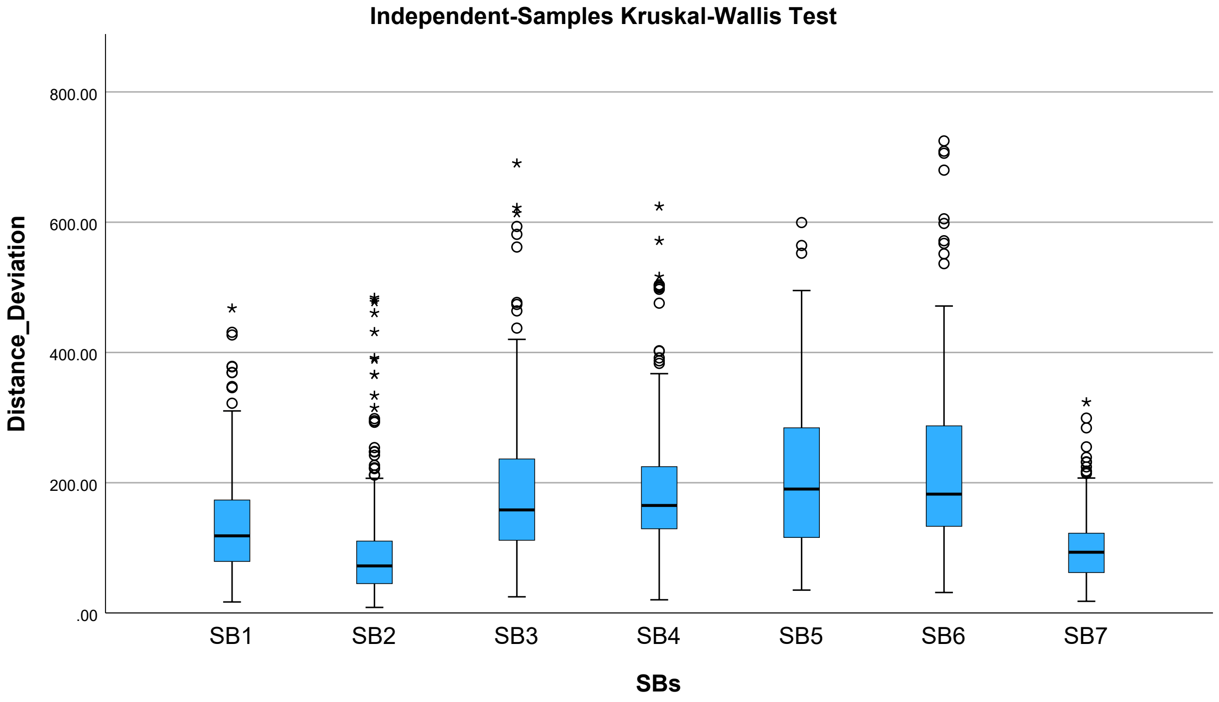

Pairwise Comparisons of SBs

Sample 1-Sample 2	Test Statistic	Std. Error	Std. Test Statistic	Sig.	Adj. Sig.[a]
SB2-SB7	-74.404	44.285	-1.680	.093	1.000
SB2-SB1	268.310	44.285	6.059	<.001	.000
SB2-SB3	-512.140	44.285	-11.565	<.001	.000
SB2-SB4	-568.119	44.285	-12.829	<.001	.000
SB2-SB5	-591.573	44.285	-13.358	<.001	.000
SB2-SB6	-632.810	44.285	-14.289	<.001	.000
SB7-SB1	193.906	44.285	4.379	<.001	.000
SB7-SB3	437.735	44.285	9.884	<.001	.000
SB7-SB4	493.715	44.285	11.149	<.001	.000
SB7-SB5	517.169	44.285	11.678	<.001	.000
SB7-SB6	558.406	44.285	12.609	<.001	.000
SB1-SB3	-243.829	44.285	-5.506	<.001	.000
SB1-SB4	-299.808	44.285	-6.770	<.001	.000
SB1-SB5	-323.262	44.285	-7.300	<.001	.000
SB1-SB6	-364.500	44.285	-8.231	<.001	.000
SB3-SB4	-55.979	44.285	-1.264	.206	1.000
SB3-SB5	-79.433	44.285	-1.794	.073	1.000
SB3-SB6	-120.671	44.285	-2.725	.006	.135
SB4-SB5	-23.454	44.285	-.530	.596	1.000
SB4-SB6	-64.692	44.285	-1.461	.144	1.000
SB5-SB6	-41.237	44.285	-.931	.352	1.000

Each row tests the null hypothesis that the Sample 1 and Sample 2 distributions are the same.
Asymptotic significances (2-sided tests) are displayed. The significance level is .050.

a. Significance values have been adjusted by the Bonferroni correction for multiple tests.

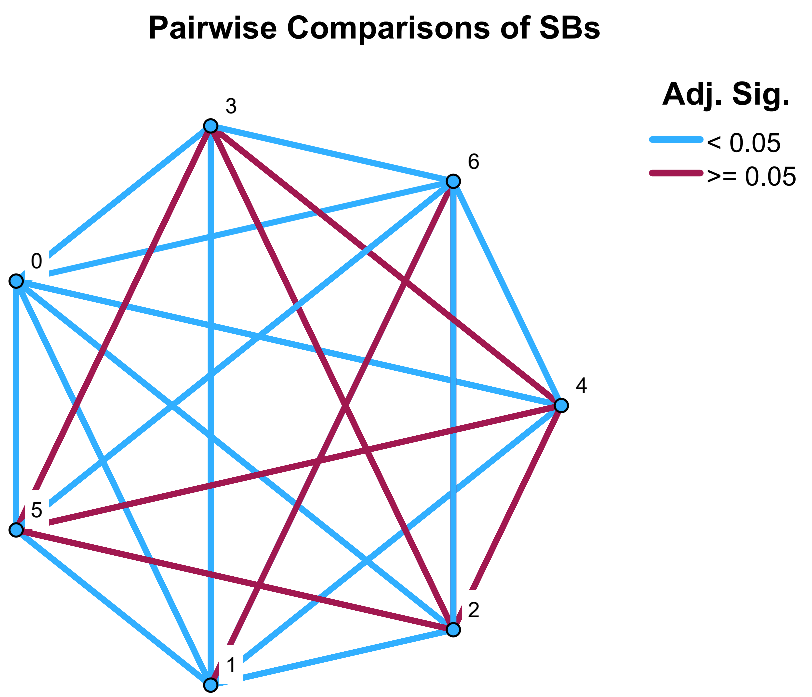

Each node shows the sample average rank of SBs.

Angular_Deviation across SBs

Independent-Samples Kruskal-Wallis Test Summary

Total N	1680
Test Statistic	410.318[a]
Degree Of Freedom	6
Asymptotic Sig.(2-sided test)	<.001

a. The test statistic is adjusted for ties.

Independent-Samples Kruskal-Wallis Test

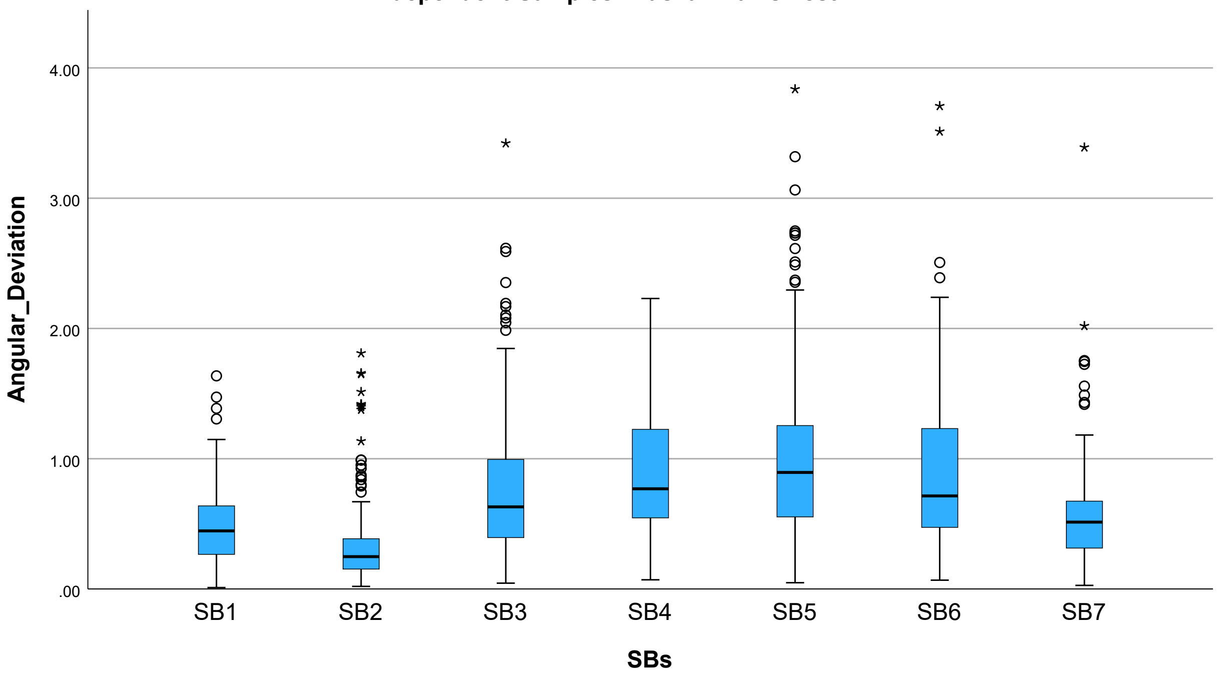

Pairwise Comparisons of SBs

Sample 1-Sample 2	Test Statistic	Std. Error	Std. Test Statistic	Sig.	Adj. Sig.[a]
SB2-SB1	247.238	44.285	5.583	<.001	.000
SB2-SB7	-327.273	44.285	-7.390	<.001	.000
SB2-SB3	-521.735	44.285	-11.781	<.001	.000
SB2-SB6	-630.273	44.285	-14.232	<.001	.000
SB2-SB4	-666.760	44.285	-15.056	<.001	.000
SB2-SB5	-694.144	44.285	-15.674	<.001	.000
SB1-SB7	-80.035	44.285	-1.807	.071	1.000
SB1-SB3	-274.498	44.285	-6.198	<.001	.000
SB1-SB6	-383.035	44.285	-8.649	<.001	.000
SB1-SB4	-419.523	44.285	-9.473	<.001	.000
SB1-SB5	-446.906	44.285	-10.092	<.001	.000
SB7-SB3	194.463	44.285	4.391	<.001	.000
SB7-SB6	303.000	44.285	6.842	<.001	.000
SB7-SB4	339.488	44.285	7.666	<.001	.000
SB7-SB5	366.871	44.285	8.284	<.001	.000
SB3-SB6	-108.537	44.285	-2.451	.014	.299
SB3-SB4	-145.025	44.285	-3.275	.001	.022
SB3-SB5	-172.408	44.285	-3.893	<.001	.002
SB6-SB4	36.488	44.285	.824	.410	1.000

Pairwise Comparisons of SBs

Sample 1-Sample 2	Test Statistic	Std. Error	Std. Test Statistic	Sig.	Adj. Sig.[a]
SB6-SB5	63.871	44.285	1.442	.149	1.000
SB4-SB5	-27.383	44.285	-.618	.536	1.000

Each row tests the null hypothesis that the Sample 1 and Sample 2 distributions are the same.
Asymptotic significances (2-sided tests) are displayed. The significance level is .050.

a. Significance values have been adjusted by the Bonferroni correction for multiple tests.

Pairwise Comparisons of SBs

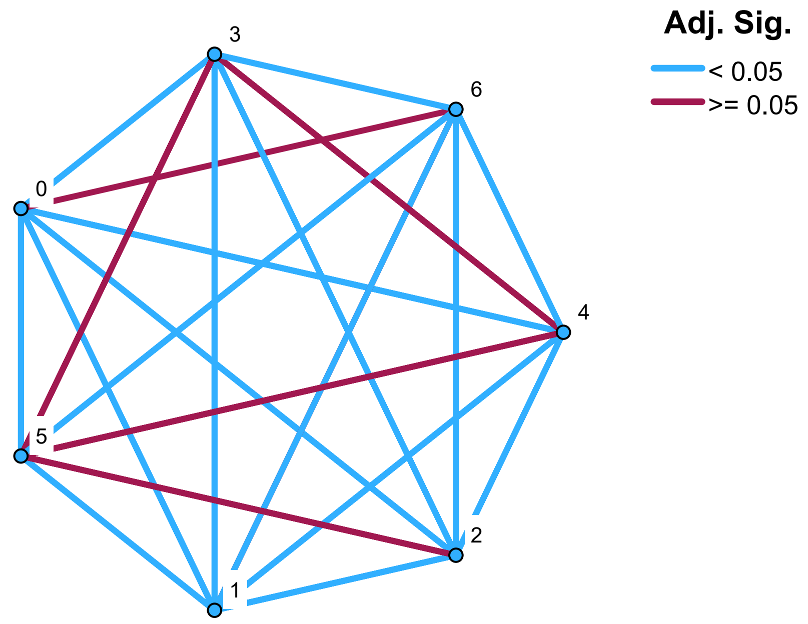

Each node shows the sample average rank of SBs.

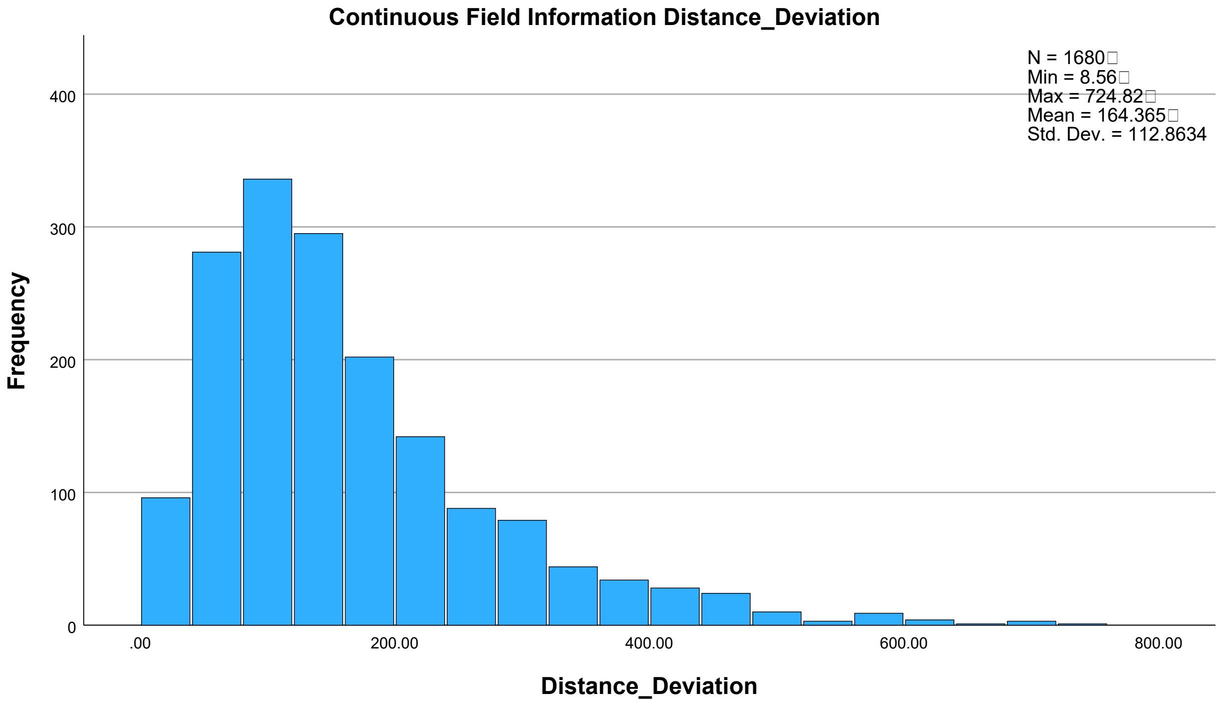
Continuous Field Information Distance_Deviation
N = 1680
Min = 8.56
Max = 724.82
Mean = 164.365
Std. Dev. = 112.8634
Frequency
400
300
200
100
0
.00
200.00
400.00
600.00
800.00
Distance_Deviation

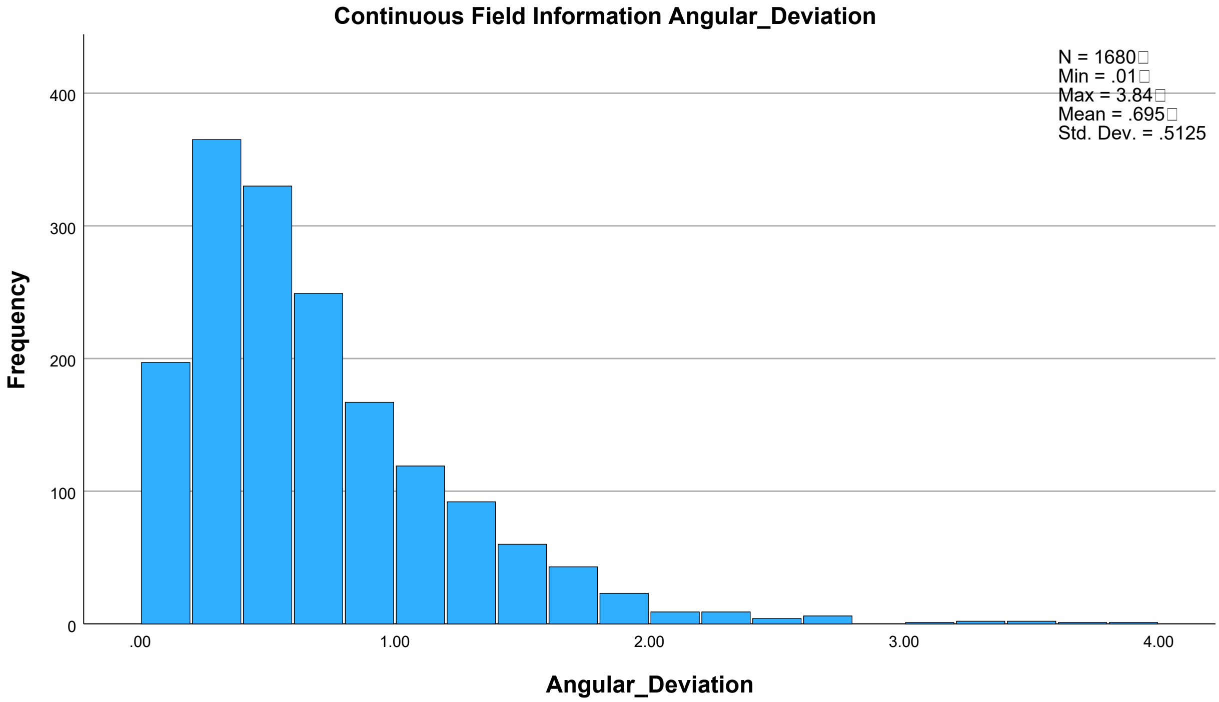
Continuous Field Information Angular_Deviation
N = 1680
Min = .01
Max = 3.84
Mean = .695
Std. Dev. = .5125
Frequency
400
300
200
100
0
.00
1.00
2.00
3.00
4.00
Angular_Deviation

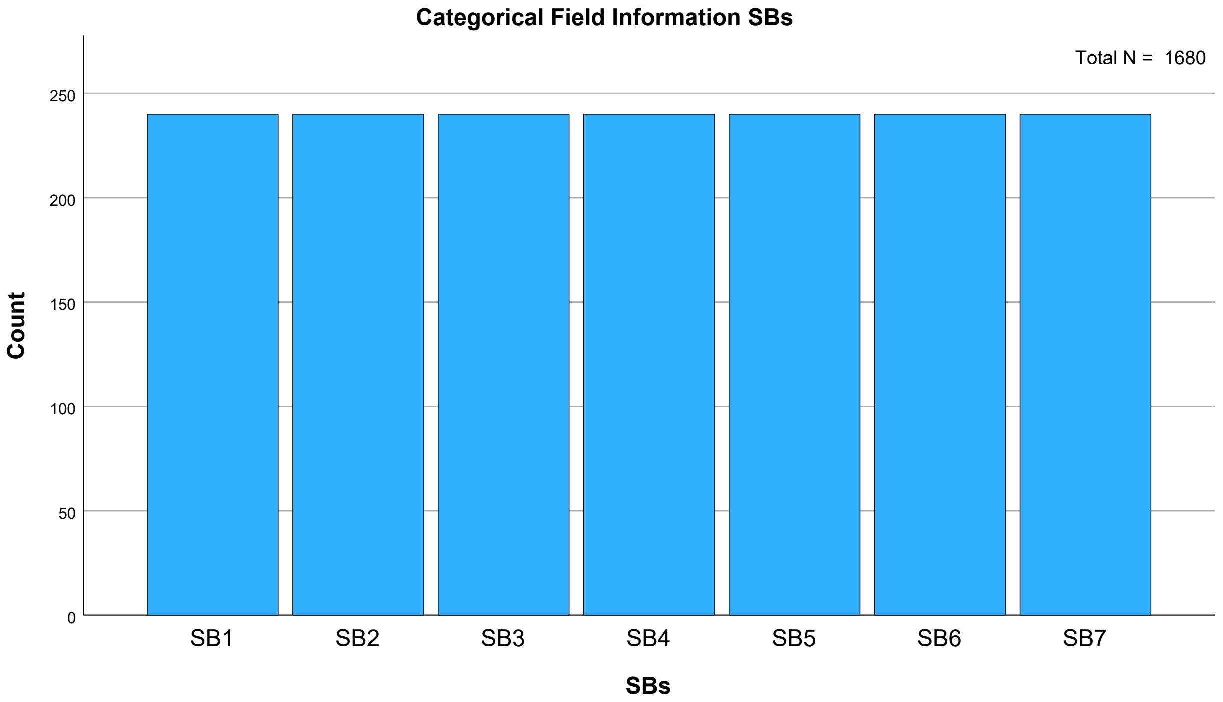
Categorical Field Information SBs
Total N = 1680
Count
250
200
150
100
50
0
SB1
SB2
SB3
SB4
SB5
SB6
SB7
SBs

Nonparametric Tests

Notes

Output Created		30-MAY-2023 10:45:49
Comments		
Input	Data	\\Client\C$\SSPS\All3Ddis and ang.sav
	Active Dataset	DataSet2
	Filter	<none>
	Weight	<none>
	Split File	<none>
	N of Rows in Working Data File	1680
Syntax		NPTESTS /INDEPENDENT TEST (Dis6 Ang6) GROUP (S6) KRUSKAL_WALLIS (COMPARE=PAIRWISE) /MISSING SCOPE=ANALYSIS USERMISSING=EXCLUDE /CRITERIA ALPHA=0.05 CILEVEL=95.
Resources	Processor Time	00:00:01.17
	Elapsed Time	00:00:01.22

Hypothesis Test Summary

	Null Hypothesis	Test	Sig.[a,b]
1	The distribution of Dis6 is the same across categories of S6.	Independent-Samples Kruskal-Wallis Test	.002
2	The distribution of Ang6 is the same across categories of S6.	Independent-Samples Kruskal-Wallis Test	<.001

Hypothesis Test Summary

	Decision
1	Reject the null hypothesis.
2	Reject the null hypothesis.

a. The significance level is .050.

b. Asymptotic significance is displayed.

Independent-Samples Kruskal-Wallis Test

Dis6 across S6

Independent-Samples Kruskal-Wallis Test Summary

Total N	240
Test Statistic	14.767[a]
Degree Of Freedom	3
Asymptotic Sig.(2-sided test)	.002

a. The test statistic is adjusted for ties.

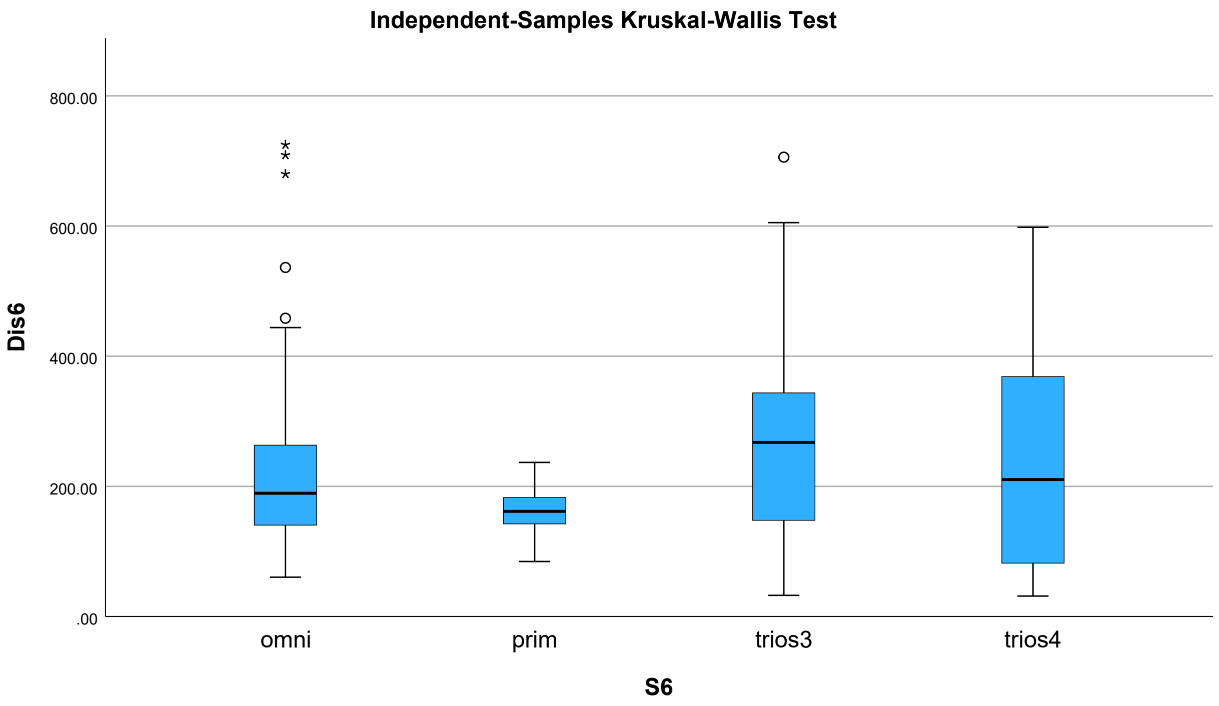

Pairwise Comparisons of S6

Sample 1-Sample 2	Test Statistic	Std. Error	Std. Test Statistic	Sig.	Adj. Sig.[a]
prim-trios4	-21.867	12.675	-1.725	.085	.507
prim-omni	29.067	12.675	2.293	.022	.131
prim-trios3	-48.133	12.675	-3.797	<.001	.001
trios4-omni	7.200	12.675	.568	.570	1.000
trios4-trios3	26.267	12.675	2.072	.038	.229
omni-trios3	-19.067	12.675	-1.504	.133	.795

Each row tests the null hypothesis that the Sample 1 and Sample 2 distributions are the same.
Asymptotic significances (2-sided tests) are displayed. The significance level is .050.

a. Significance values have been adjusted by the Bonferroni correction for multiple tests.

Pairwise Comparisons of S6

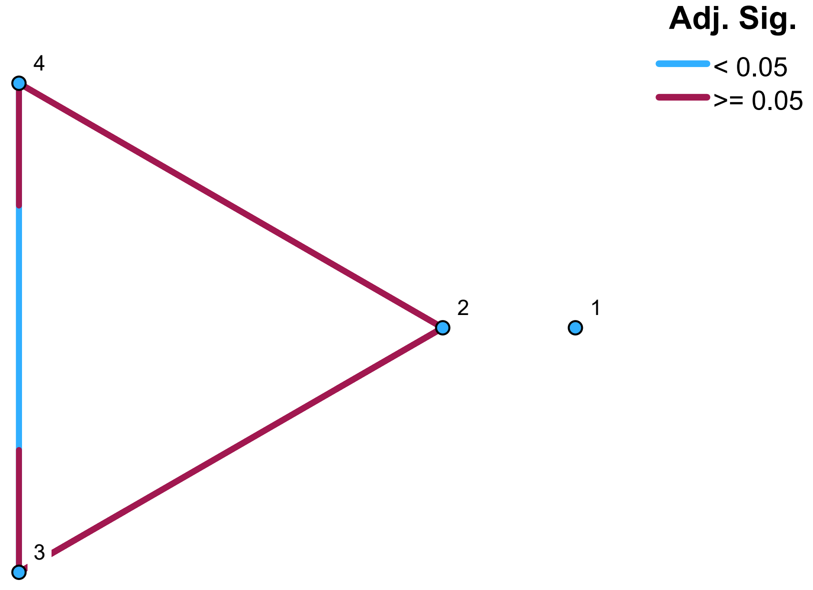

Each node shows the sample average rank of S6.

Ang6 across S6

Independent-Samples Kruskal-Wallis Test Summary

Total N	240
Test Statistic	30.931[a]
Degree Of Freedom	3
Asymptotic Sig.(2-sided test)	<.001

a. The test statistic is adjusted for ties.

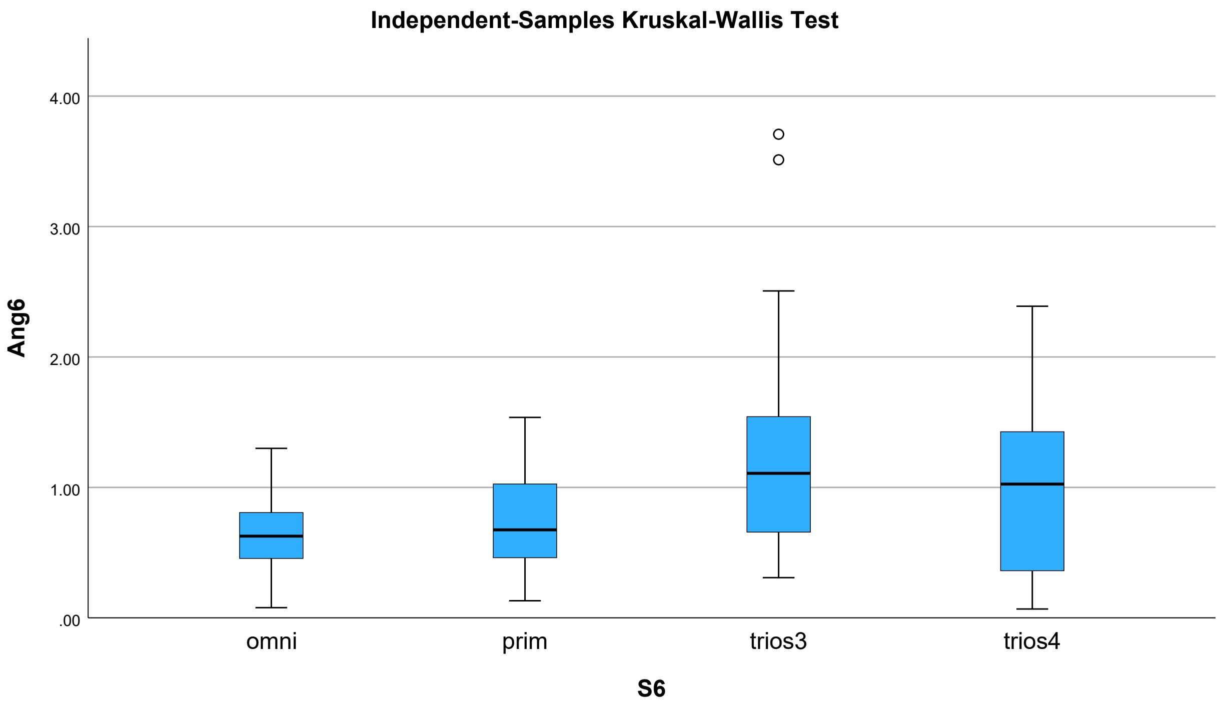

Pairwise Comparisons of S6

Sample 1-Sample 2	Test Statistic	Std. Error	Std. Test Statistic	Sig.	Adj. Sig.[a]
omni-prim	-14.767	12.675	-1.165	.244	1.000
omni-trios4	-32.783	12.675	-2.586	.010	.058
omni-trios3	-66.783	12.675	-5.269	<.001	.000
prim-trios4	-18.017	12.675	-1.421	.155	.931
prim-trios3	-52.017	12.675	-4.104	<.001	.000
trios4-trios3	34.000	12.675	2.682	.007	.044

Each row tests the null hypothesis that the Sample 1 and Sample 2 distributions are the same.
Asymptotic significances (2-sided tests) are displayed. The significance level is .050.

a. Significance values have been adjusted by the Bonferroni correction for multiple tests.

Pairwise Comparisons of S6

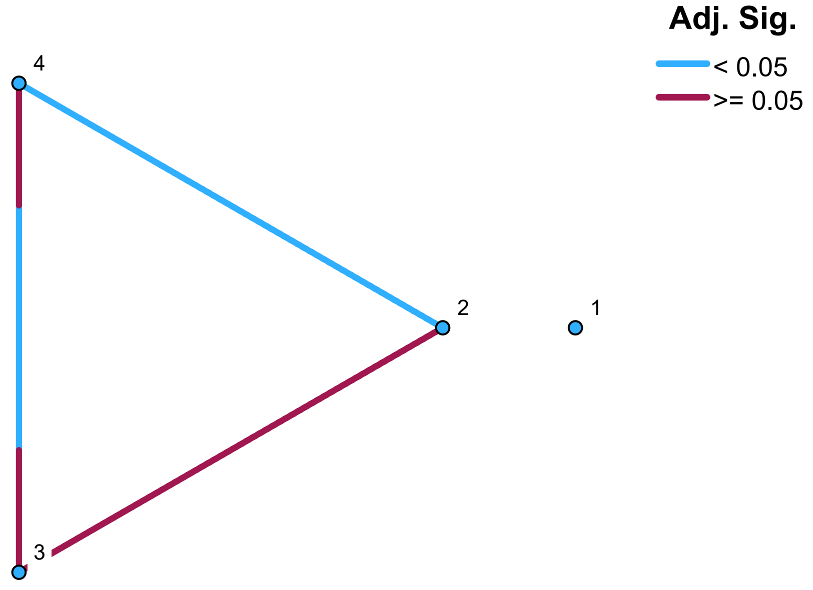

Each node shows the
sample average rank of
S6.

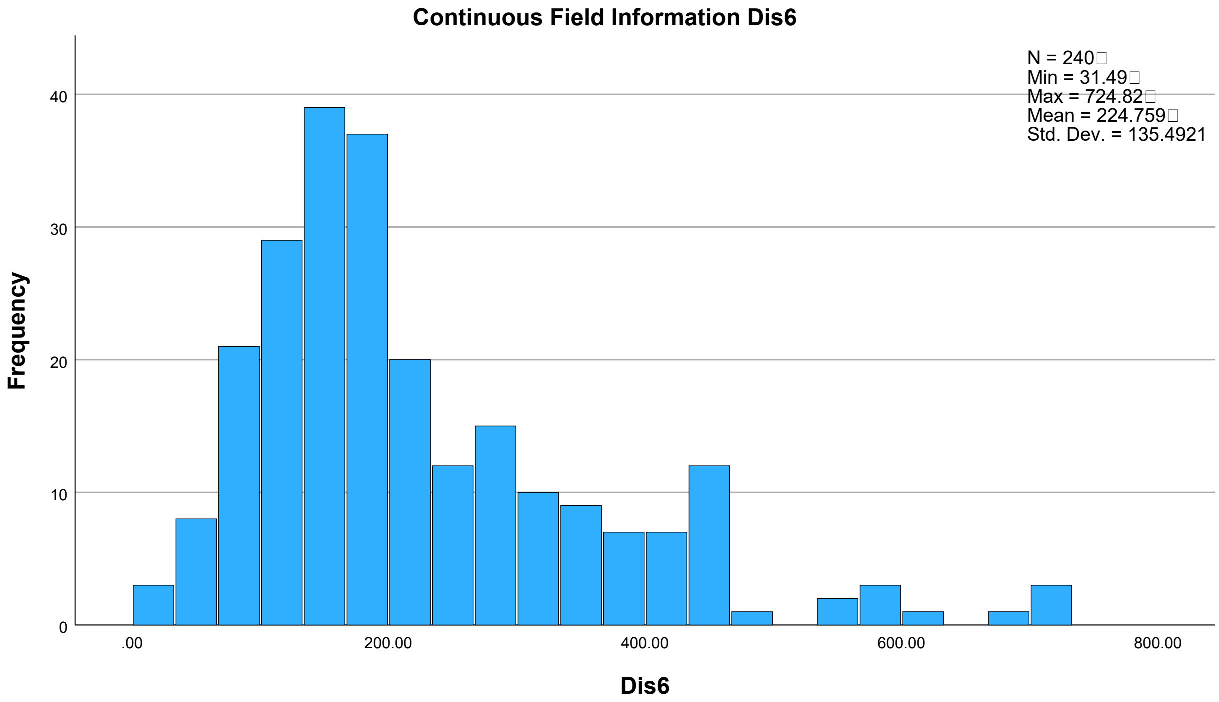
Continuous Field Information Dis6
N = 240
Min = 31.49
Max = 724.82
Mean = 224.759
Std. Dev. = 135.4921
Frequency
0
10
20
30
40
.00
200.00
400.00
600.00
800.00
Dis6

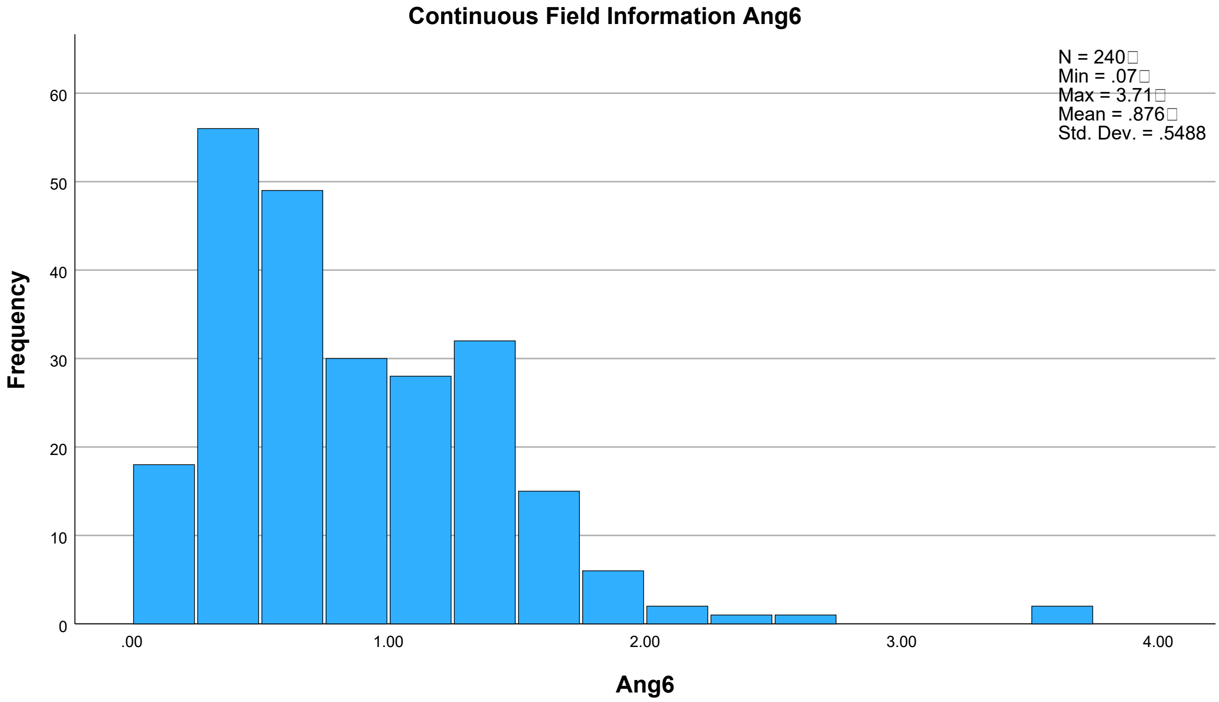
Continuous Field Information Ang6
N = 240
Min = .07
Max = 3.71
Mean = .876
Std. Dev. = .5488
Frequency
0
10
20
30
40
50
60
.00
1.00
2.00
3.00
4.00
Ang6

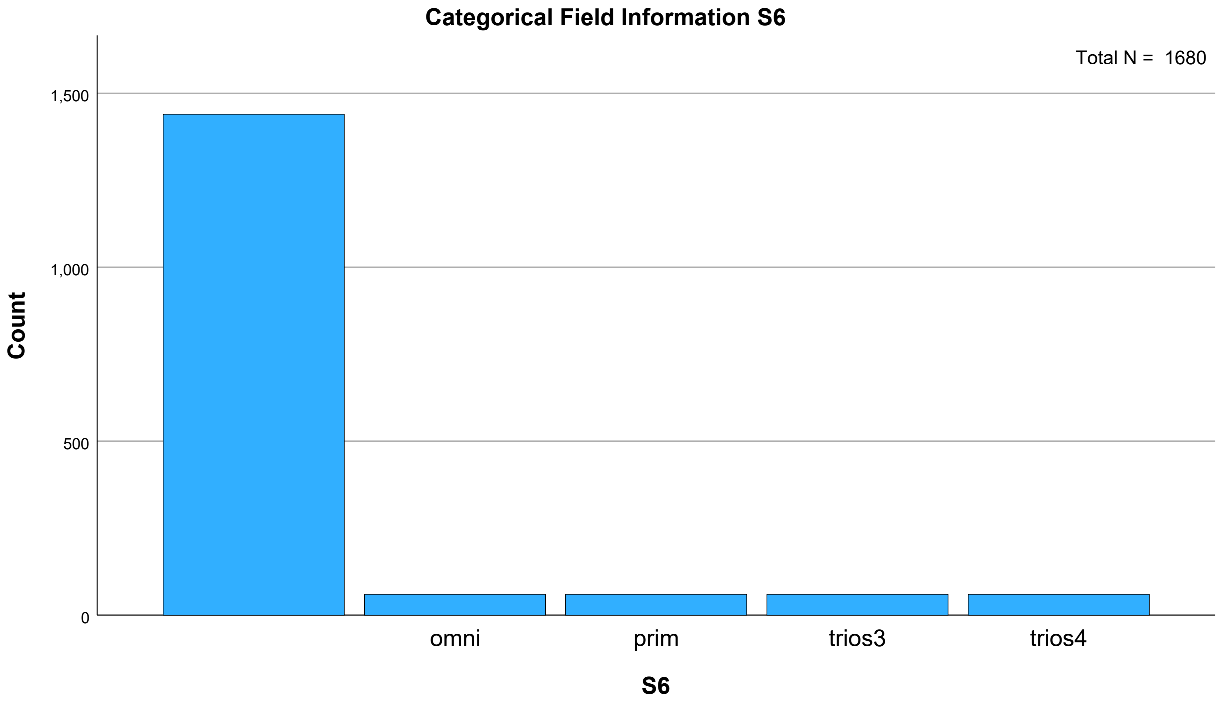
Categorical Field Information S6
Total N = 1680
1,500
1,000
500
0
Count
omni
prim
trios3
trios4
S6

Nonparametric Tests

Notes

Output Created		30-MAY-2023 10:46:34
Comments		
Input	Data	\\Client\C$\SSPS\All3Ddis and ang.sav
	Active Dataset	DataSet2
	Filter	<none>
	Weight	<none>
	Split File	<none>
	N of Rows in Working Data File	1680
Syntax		NPTESTS<br>/INDEPENDENT TEST (Dis7 Ang7) GROUP (S7) KRUSKAL_WALLIS (COMPARE=PAIRWISE)<br>/MISSING SCOPE=ANALYSIS USERMISSING=EXCLUDE<br>/CRITERIA ALPHA=0.05 CILEVEL=95.
Resources	Processor Time	00:00:01.22
	Elapsed Time	00:00:01.25

Hypothesis Test Summary

	Null Hypothesis	Test	Sig.[a,b]
1	The distribution of Dis7 is the same across categories of S7.	Independent-Samples Kruskal-Wallis Test	<.001
2	The distribution of Ang7 is the same across categories of S7.	Independent-Samples Kruskal-Wallis Test	.222

Hypothesis Test Summary

	Decision
1	Reject the null hypothesis.
2	Retain the null hypothesis.

a. The significance level is .050.

b. Asymptotic significance is displayed.

Independent-Samples Kruskal-Wallis Test

Dis7 across S7

Independent-Samples Kruskal-Wallis Test Summary

Total N	240
Test Statistic	22.406[a]
Degree Of Freedom	3
Asymptotic Sig.(2-sided test)	<.001

a. The test statistic is adjusted for ties.

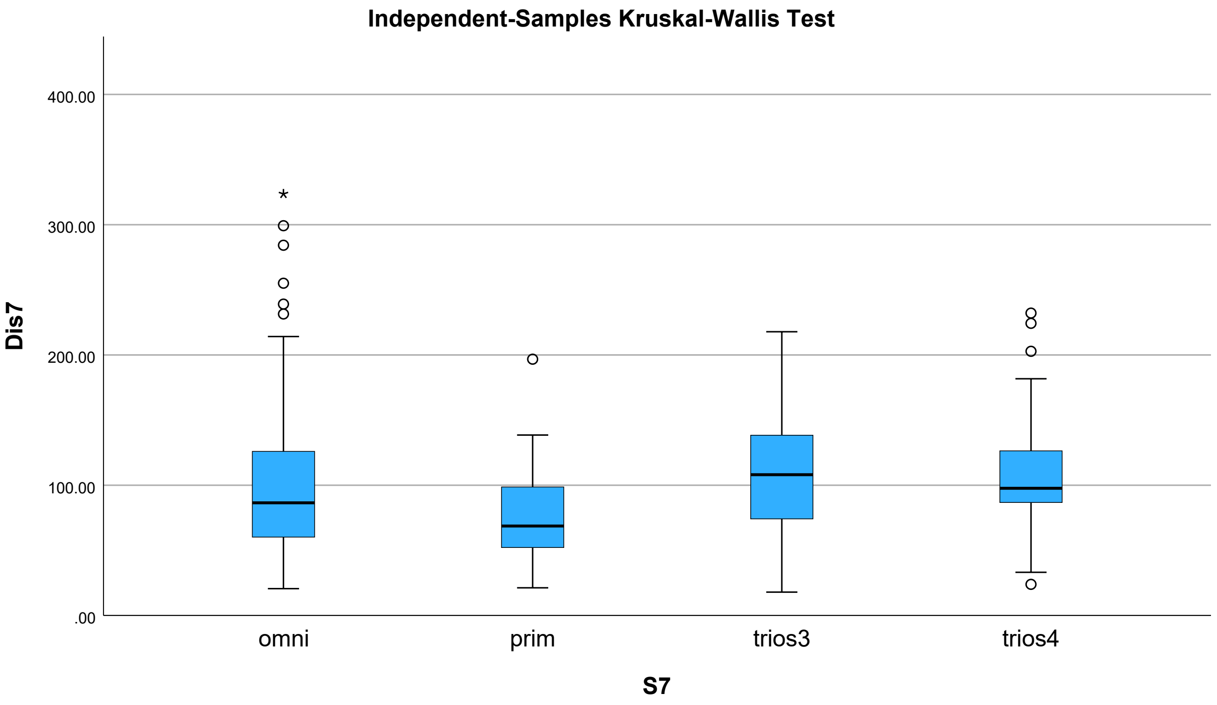

Pairwise Comparisons of S7

Sample 1-Sample 2	Test Statistic	Std. Error	Std. Test Statistic	Sig.	Adj. Sig.[a]
prim-omni	35.017	12.675	2.763	.006	.034
prim-trios4	-51.750	12.675	-4.083	<.001	.000
prim-trios3	-52.167	12.675	-4.116	<.001	.000
omni-trios4	-16.733	12.675	-1.320	.187	1.000
omni-trios3	-17.150	12.675	-1.353	.176	1.000
trios4-trios3	.417	12.675	.033	.974	1.000

Each row tests the null hypothesis that the Sample 1 and Sample 2 distributions are the same.
Asymptotic significances (2-sided tests) are displayed. The significance level is .050.

a. Significance values have been adjusted by the Bonferroni correction for multiple tests.

Pairwise Comparisons of S7

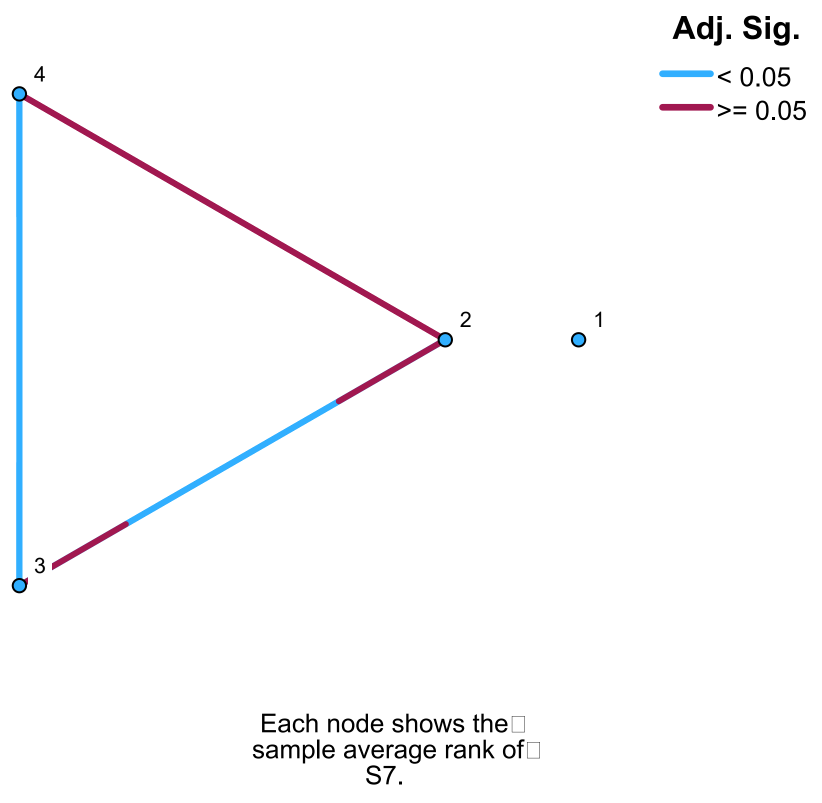

Ang7 across S7

Independent-Samples Kruskal-Wallis Test Summary

Total N	240
Test Statistic	4.391[a]
Degree Of Freedom	3
Asymptotic Sig.(2-sided test)	.222

a. The test statistic is adjusted for ties.

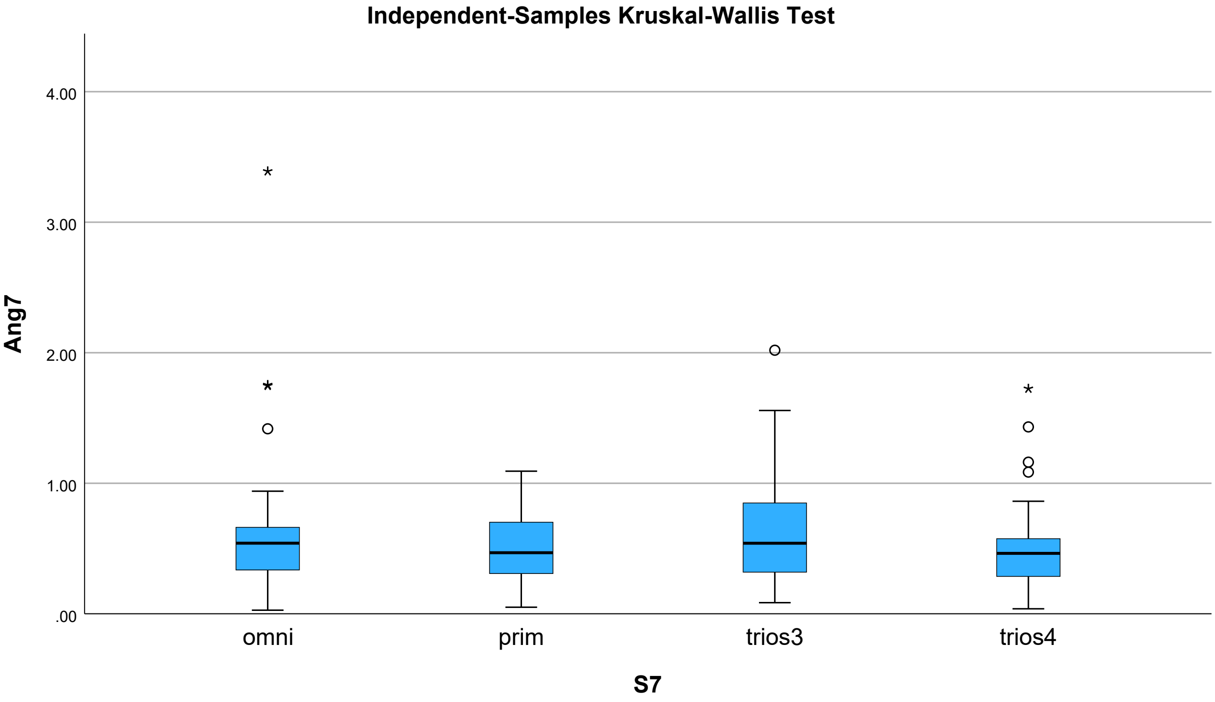

Pairwise Comparisons of S7

Sample 1-Sample 2	Test Statistic	Std. Error	Std. Test Statistic	Sig.	Adj. Sig.[a]
trios4-prim	9.133	12.675	.721	.471	1.000
trios4-omni	20.517	12.675	1.619	.106	.633
trios4-trios3	23.617	12.675	1.863	.062	.375
prim-omni	11.383	12.675	.898	.369	1.000
prim-trios3	-14.483	12.675	-1.143	.253	1.000
omni-trios3	-3.100	12.675	-.245	.807	1.000

Each row tests the null hypothesis that the Sample 1 and Sample 2 distributions are the same.
Asymptotic significances (2-sided tests) are displayed. The significance level is .050.

a. Significance values have been adjusted by the Bonferroni correction for multiple tests.

Pairwise Comparisons of S7

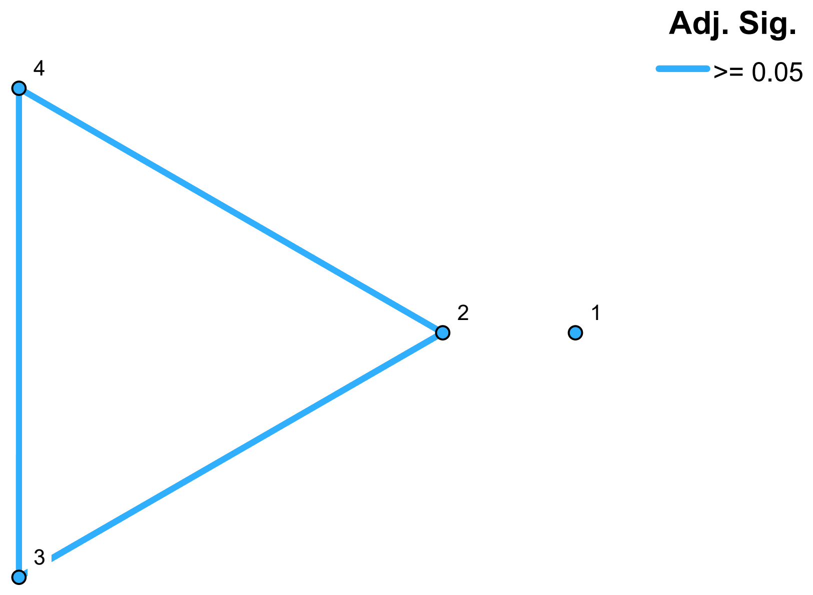

Each node shows the
sample average rank of
S7.

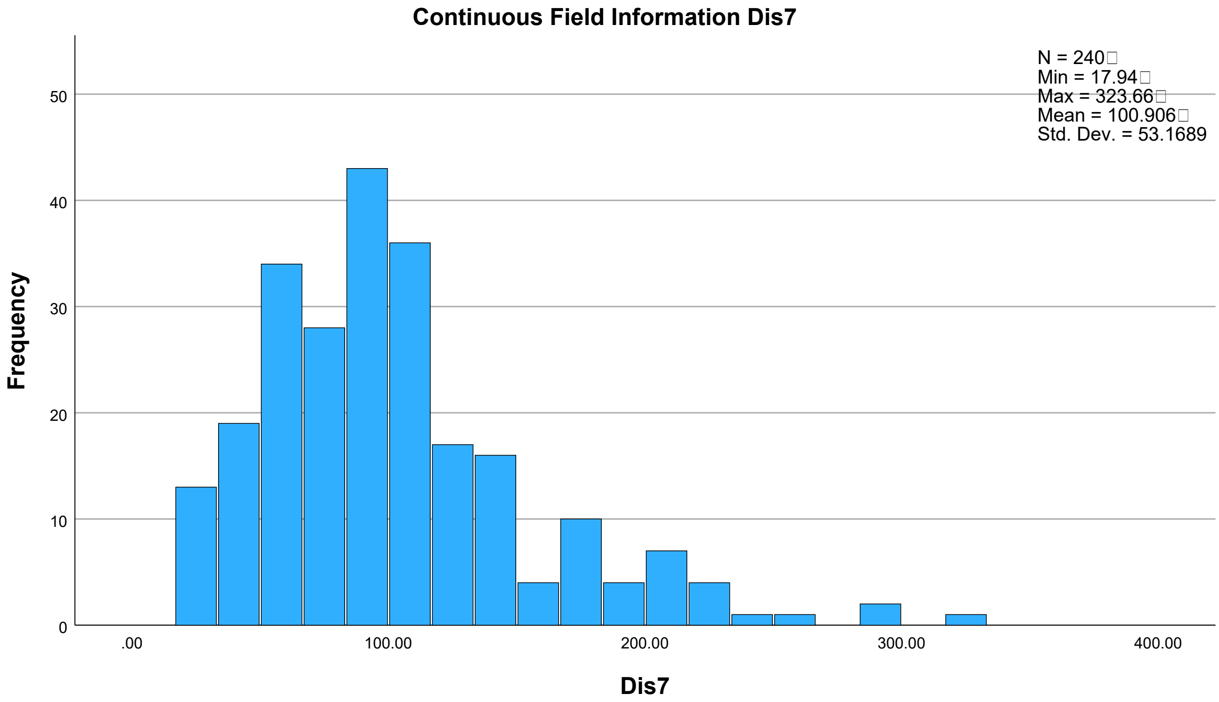
Continuous Field Information Dis7
N = 240
Min = 17.94
Max = 323.66
Mean = 100.906
Std. Dev. = 53.1689
Frequency
0
10
20
30
40
50
.00
100.00
200.00
300.00
400.00
Dis7

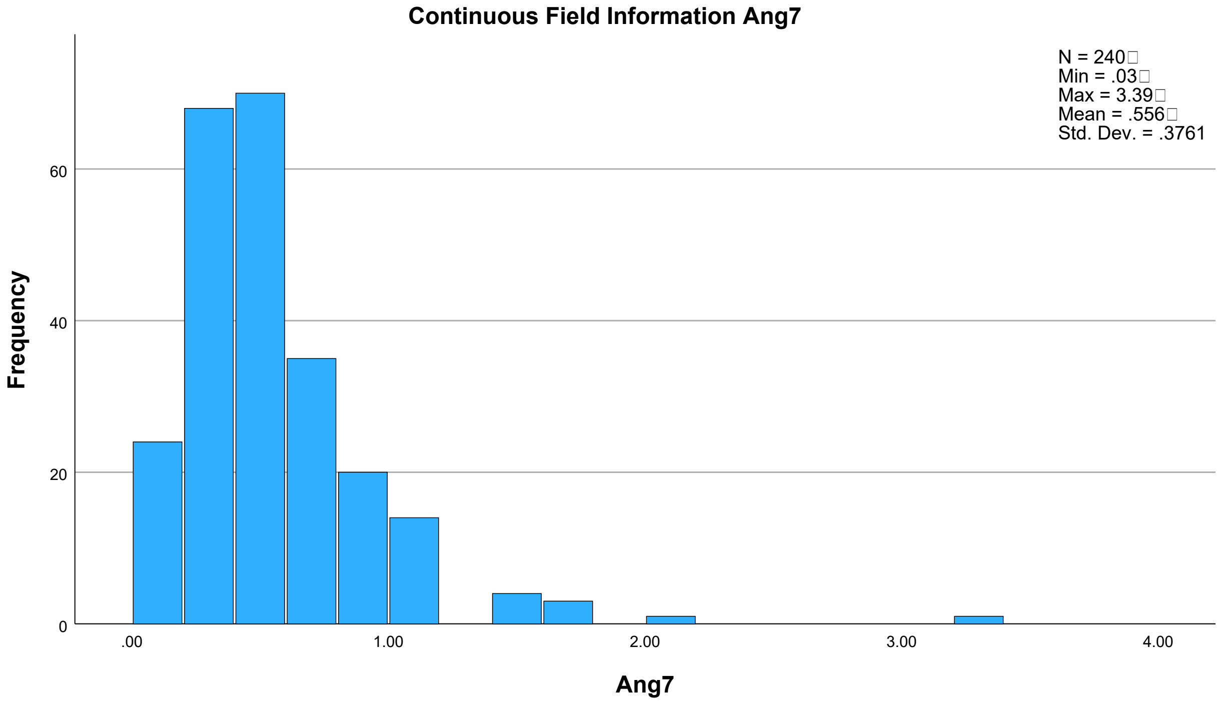
Continuous Field Information Ang7
N = 240
Min = .03
Max = 3.39
Mean = .556
Std. Dev. = .3761
Frequency
0
20
40
60
.00
1.00
2.00
3.00
4.00
Ang7

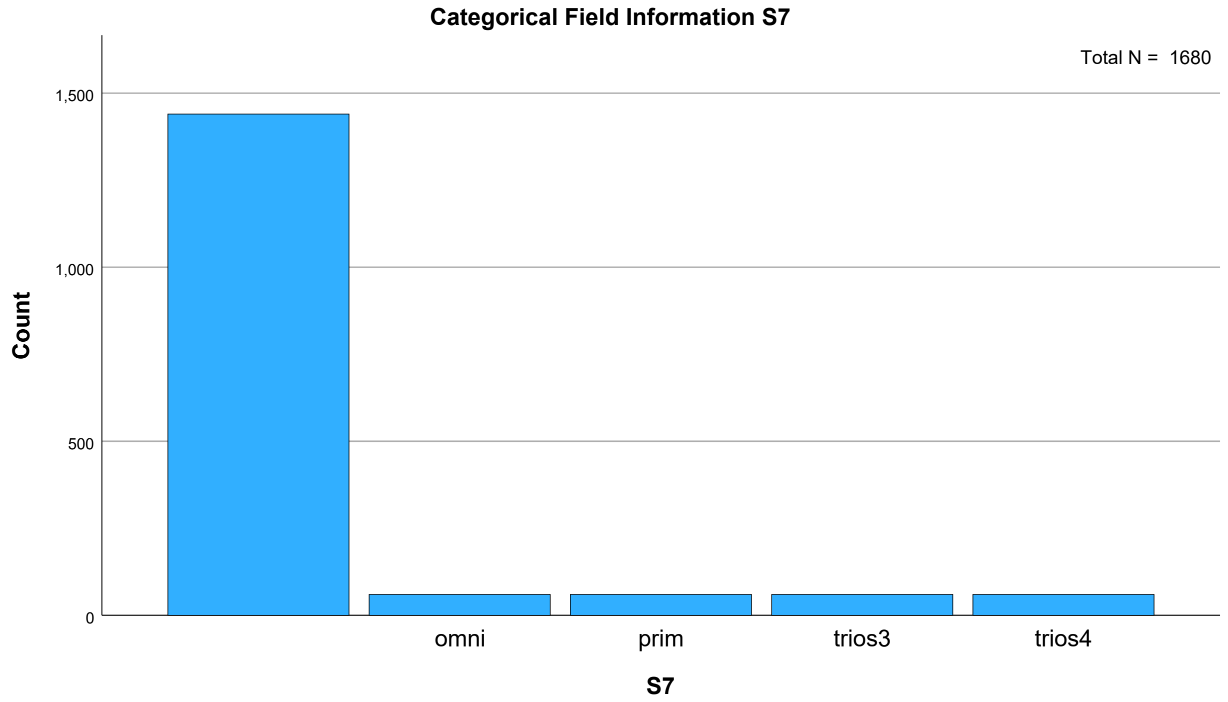
Categorical Field Information S7
Total N = 1680
1,500
1,000
500
0
Count
omni
prim
trios3
trios4
S7

Nonparametric Tests

Notes

Output Created		30-MAY-2023 10:42:41
Comments		
Input	Data	\\Client\C$\SSPS\All3Ddis and ang.sav
	Active Dataset	DataSet2
	Filter	<none>
	Weight	<none>
	Split File	<none>
	N of Rows in Working Data File	1680
Syntax		NPTESTS /INDEPENDENT TEST (Dis3 Ang3) GROUP (S3) KRUSKAL_WALLIS (COMPARE=PAIRWISE) /MISSING SCOPE=ANALYSIS USERMISSING=EXCLUDE /CRITERIA ALPHA=0.05 CILEVEL=95.
Resources	Processor Time	00:00:01.23
	Elapsed Time	00:00:01.25

Hypothesis Test Summary

	Null Hypothesis	Test	Sig.[a,b]
1	The distribution of Dis3 is the same across categories of S3.	Independent-Samples Kruskal-Wallis Test	<.001
2	The distribution of Ang3 is the same across categories of S3.	Independent-Samples Kruskal-Wallis Test	.008

Hypothesis Test Summary

	Decision
1	Reject the null hypothesis.
2	Reject the null hypothesis.

a. The significance level is .050.

b. Asymptotic significance is displayed.

Independent-Samples Kruskal-Wallis Test

Dis3 across S3

Independent-Samples Kruskal-Wallis Test Summary

Total N	240
Test Statistic	36.055[a]
Degree Of Freedom	3
Asymptotic Sig.(2-sided test)	<.001

a. The test statistic is adjusted for ties.

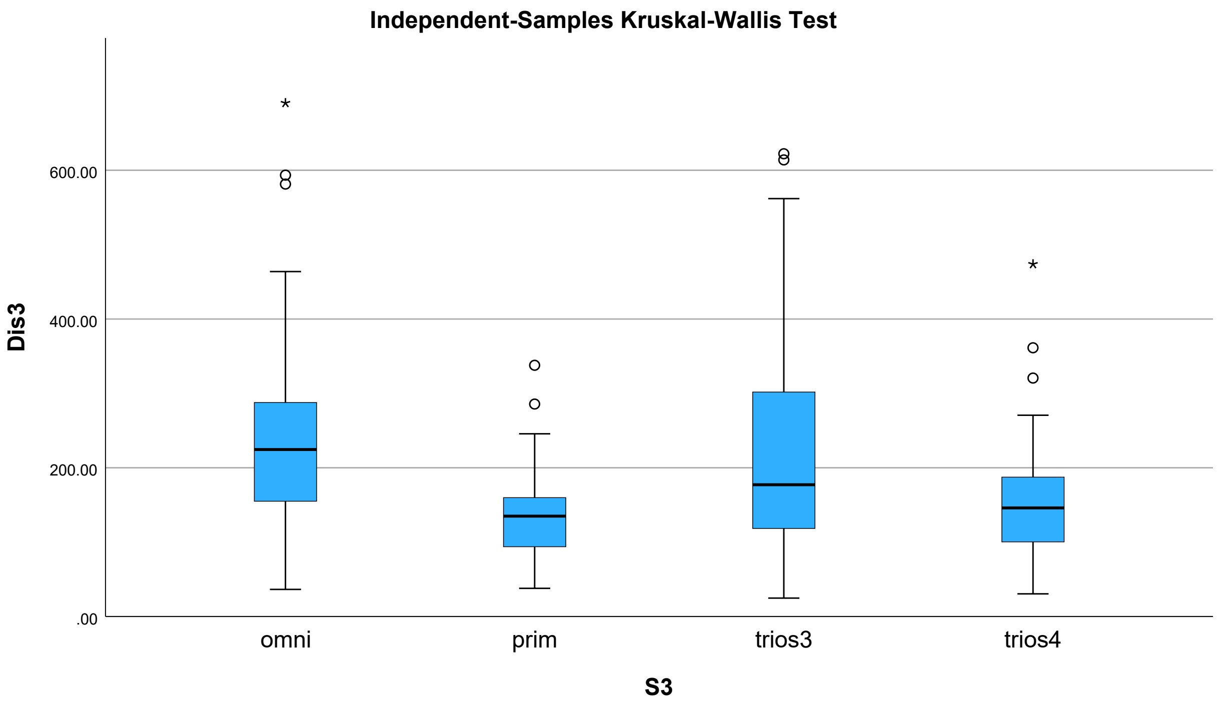

Pairwise Comparisons of S3

Sample 1-Sample 2	Test Statistic	Std. Error	Std. Test Statistic	Sig.	Adj. Sig.[a]
prim-trios4	-17.133	12.675	-1.352	.176	1.000
prim-trios3	-48.733	12.675	-3.845	<.001	.001
prim-omni	69.200	12.675	5.459	<.001	.000
trios4-trios3	31.600	12.675	2.493	.013	.076
trios4-omni	52.067	12.675	4.108	<.001	.000
trios3-omni	20.467	12.675	1.615	.106	.638

Each row tests the null hypothesis that the Sample 1 and Sample 2 distributions are the same.
Asymptotic significances (2-sided tests) are displayed. The significance level is .050.

a. Significance values have been adjusted by the Bonferroni correction for multiple tests.

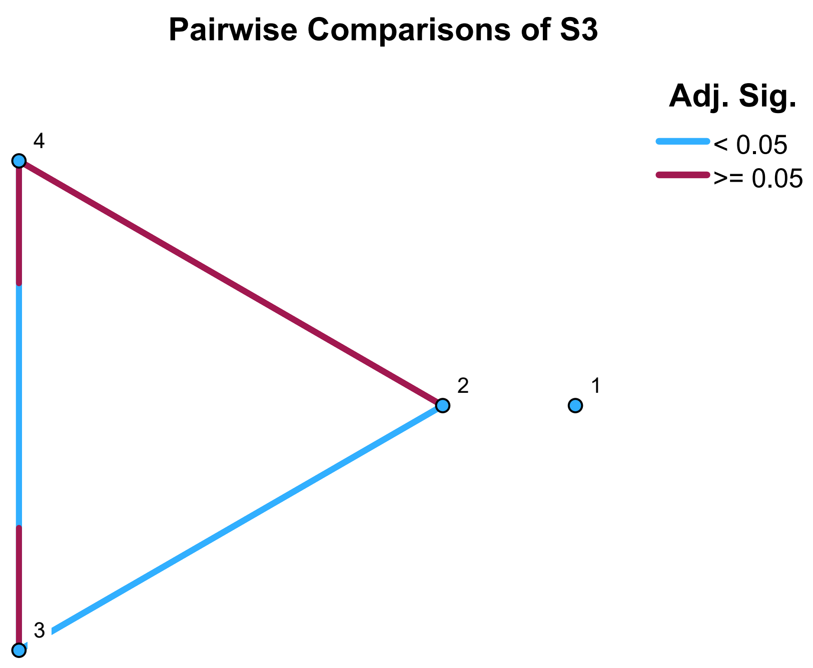

Each node shows the sample average rank of S3.

Ang3 across S3

Independent-Samples Kruskal-Wallis Test Summary

Total N	240
Test Statistic	11.874[a]
Degree Of Freedom	3
Asymptotic Sig.(2-sided test)	.008

a. The test statistic is adjusted for ties.

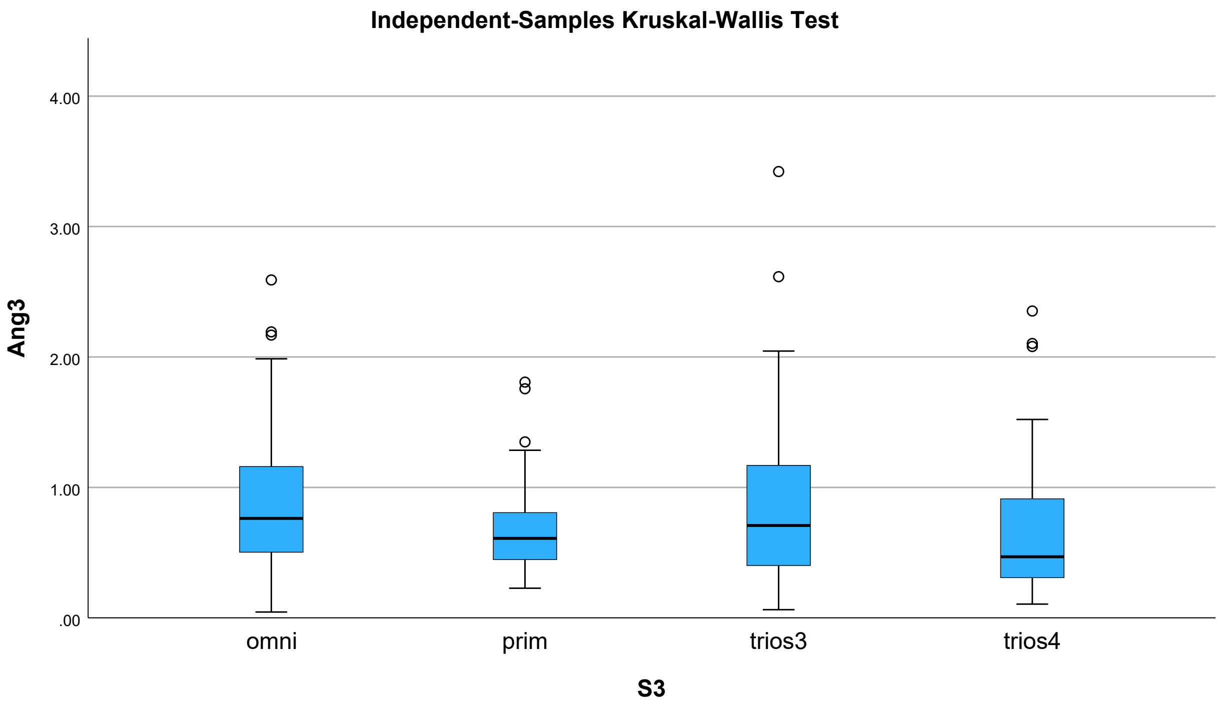

Pairwise Comparisons of S3

Sample 1-Sample 2	Test Statistic	Std. Error	Std. Test Statistic	Sig.	Adj. Sig.[a]
trios4-prim	17.967	12.675	1.417	.156	.938
trios4-trios3	29.067	12.675	2.293	.022	.131
trios4-omni	42.100	12.675	3.321	<.001	.005
prim-trios3	-11.100	12.675	-.876	.381	1.000
prim-omni	24.133	12.675	1.904	.057	.342
trios3-omni	13.033	12.675	1.028	.304	1.000

Each row tests the null hypothesis that the Sample 1 and Sample 2 distributions are the same.
Asymptotic significances (2-sided tests) are displayed. The significance level is .050.

a. Significance values have been adjusted by the Bonferroni correction for multiple tests.

Pairwise Comparisons of S3

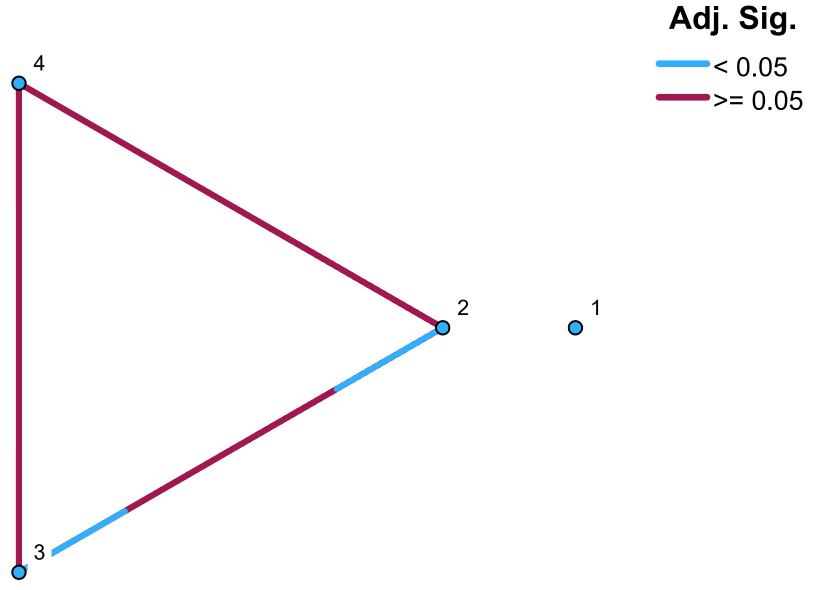

Each node shows the sample average rank of S3.

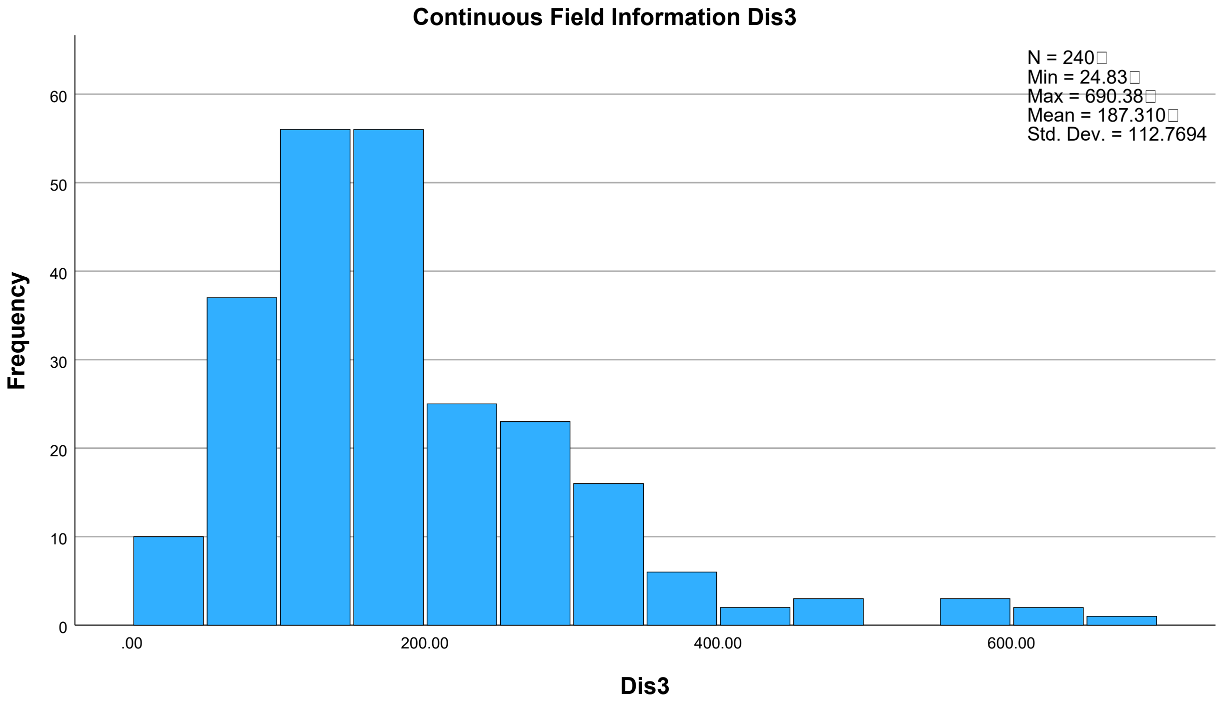
Continuous Field Information Dis3
N = 240
Min = 24.83
Max = 690.38
Mean = 187.310
Std. Dev. = 112.7694
Frequency
0
10
20
30
40
50
60
.00
200.00
400.00
600.00
Dis3

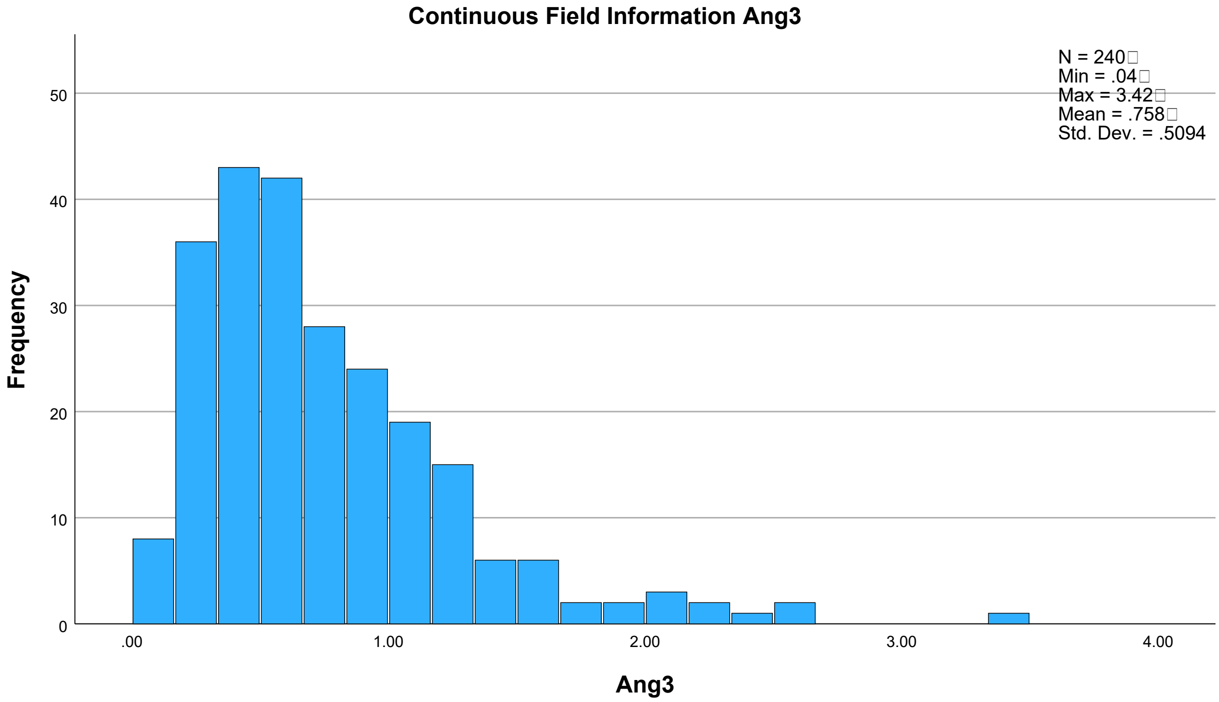
Continuous Field Information Ang3
N = 240
Min = .04
Max = 3.42
Mean = .758
Std. Dev. = .5094
Frequency
0
10
20
30
40
50
.00
1.00
2.00
3.00
4.00
Ang3

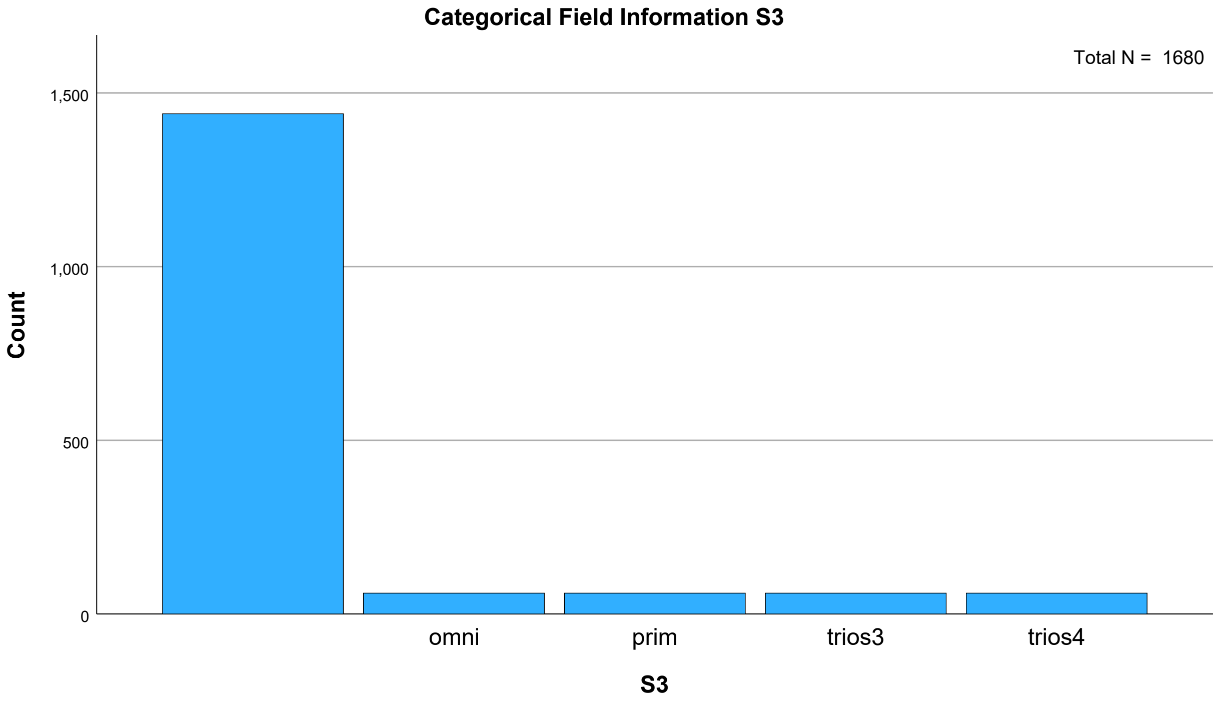
Categorical Field Information S3
Total N = 1680
1,500
1,000
500
0
Count
omni
prim
trios3
trios4
S3

Nonparametric Tests

Notes

Output Created		30-MAY-2023 10:44:01
Comments		
Input	Data	\\Client\C$\SSPS\All3Ddis and ang.sav
	Active Dataset	DataSet2
	Filter	<none>
	Weight	<none>
	Split File	<none>
	N of Rows in Working Data File	1680
Syntax		NPTESTS /INDEPENDENT TEST (Dis4 Ang4) GROUP (S4) KRUSKAL_WALLIS (COMPARE=PAIRWISE) /MISSING SCOPE=ANALYSIS USERMISSING=EXCLUDE /CRITERIA ALPHA=0.05 CILEVEL=95.
Resources	Processor Time	00:00:01.20
	Elapsed Time	00:00:01.27

Hypothesis Test Summary

	Null Hypothesis	Test	Sig.[a,b]
1	The distribution of Dis4 is the same across categories of S4.	Independent-Samples Kruskal-Wallis Test	<.001
2	The distribution of Ang4 is the same across categories of S4.	Independent-Samples Kruskal-Wallis Test	<.001

Hypothesis Test Summary

	Decision
1	Reject the null hypothesis.
2	Reject the null hypothesis.

a. The significance level is .050.

b. Asymptotic significance is displayed.

Independent-Samples Kruskal-Wallis Test

Dis4 across S4

Independent-Samples Kruskal-Wallis Test Summary

Total N	240
Test Statistic	59.419[a]
Degree Of Freedom	3
Asymptotic Sig.(2-sided test)	<.001

a. The test statistic is adjusted for ties.

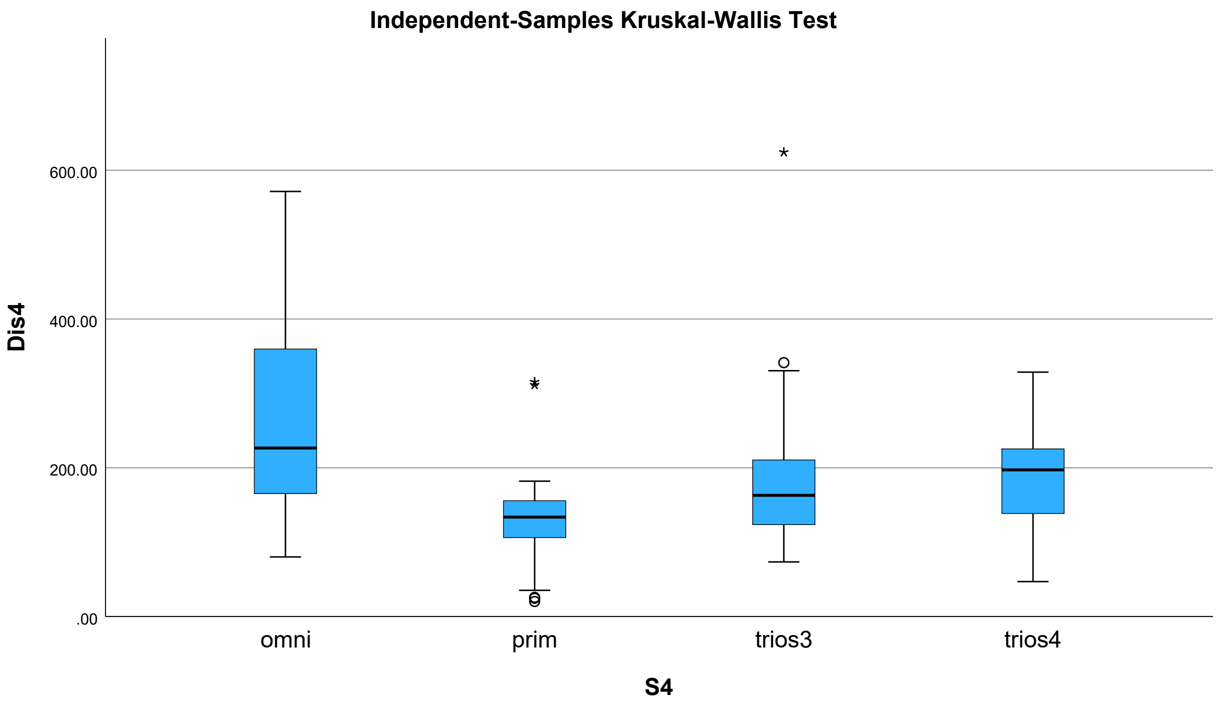

Pairwise Comparisons of S4

Sample 1-Sample 2	Test Statistic	Std. Error	Std. Test Statistic	Sig.	Adj. Sig.[a]
prim-trios3	-46.317	12.675	-3.654	<.001	.002
prim-trios4	-63.483	12.675	-5.008	<.001	.000
prim-omni	95.667	12.675	7.547	<.001	.000
trios3-trios4	-17.167	12.675	-1.354	.176	1.000
trios3-omni	49.350	12.675	3.893	<.001	.001
trios4-omni	32.183	12.675	2.539	.011	.067

Each row tests the null hypothesis that the Sample 1 and Sample 2 distributions are the same.
Asymptotic significances (2-sided tests) are displayed. The significance level is .050.

a. Significance values have been adjusted by the Bonferroni correction for multiple tests.

Pairwise Comparisons of S4

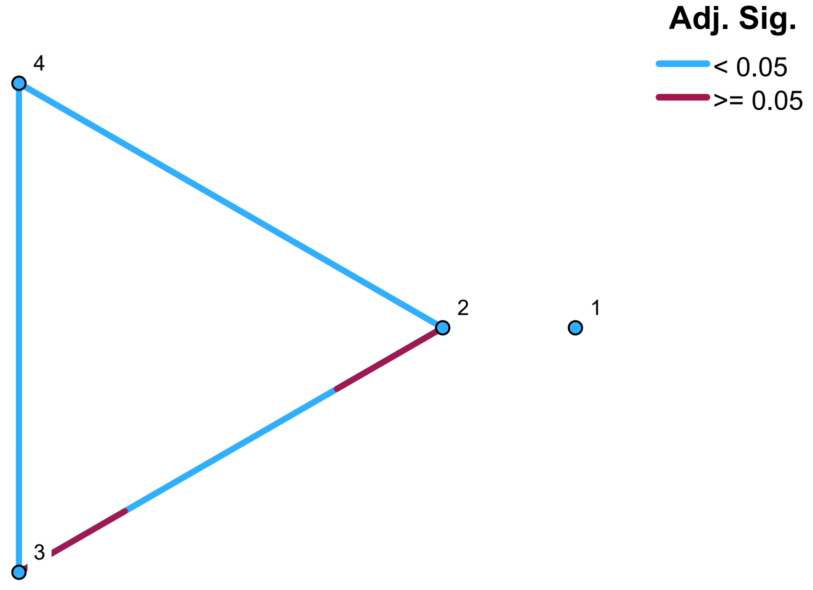

Each node shows the sample average rank of S4.

Ang4 across S4

Independent-Samples Kruskal-Wallis Test Summary

Total N	240
Test Statistic	28.607[a]
Degree Of Freedom	3
Asymptotic Sig.(2-sided test)	<.001

a. The test statistic is adjusted for ties.

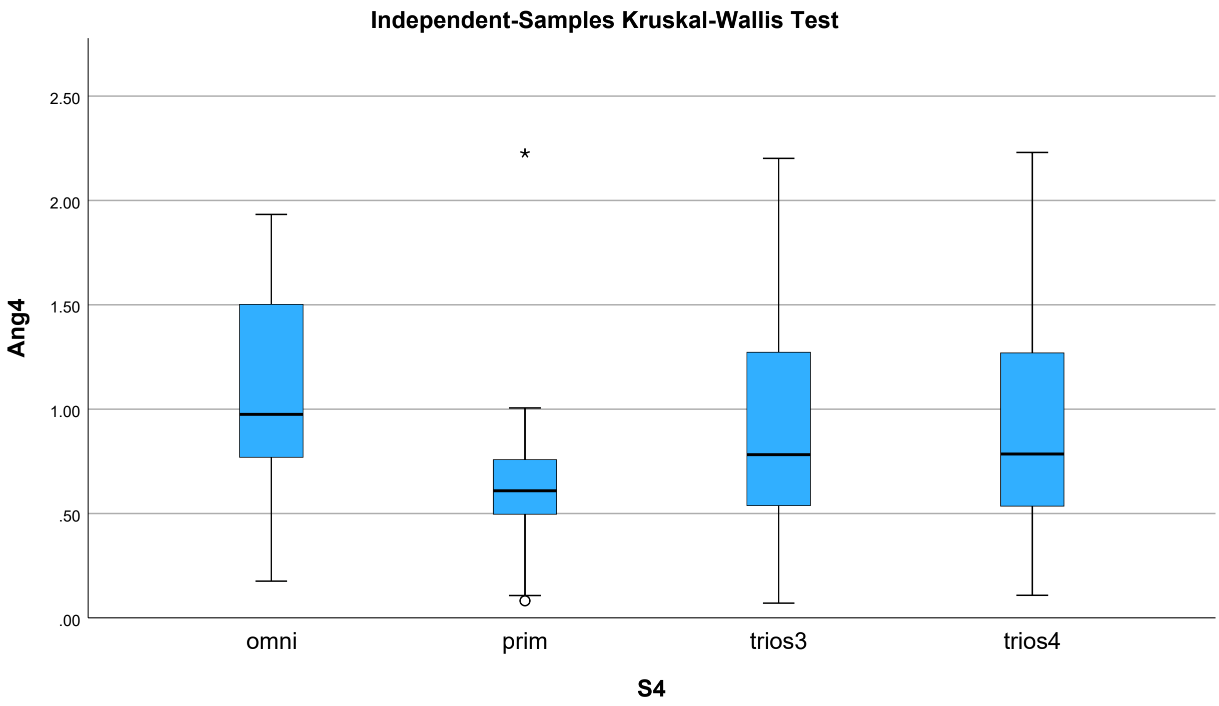

Pairwise Comparisons of S4

Sample 1-Sample 2	Test Statistic	Std. Error	Std. Test Statistic	Sig.	Adj. Sig.[a]
prim-trios4	-36.600	12.675	-2.887	.004	.023
prim-trios3	-39.100	12.675	-3.085	.002	.012
prim-omni	67.500	12.675	5.325	<.001	.000
trios4-trios3	2.500	12.675	.197	.844	1.000
trios4-omni	30.900	12.675	2.438	.015	.089
trios3-omni	28.400	12.675	2.241	.025	.150

Each row tests the null hypothesis that the Sample 1 and Sample 2 distributions are the same.
Asymptotic significances (2-sided tests) are displayed. The significance level is .050.

a. Significance values have been adjusted by the Bonferroni correction for multiple tests.

Pairwise Comparisons of S4

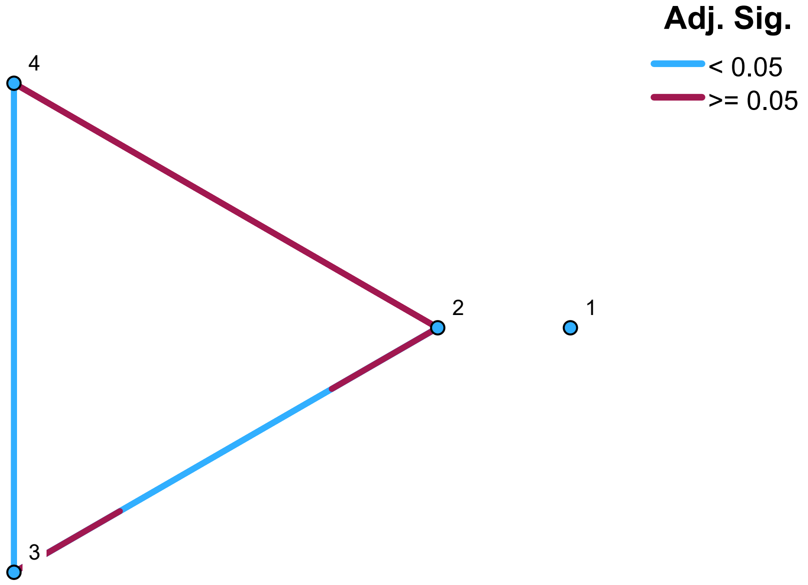

Each node shows the sample average rank of S4.

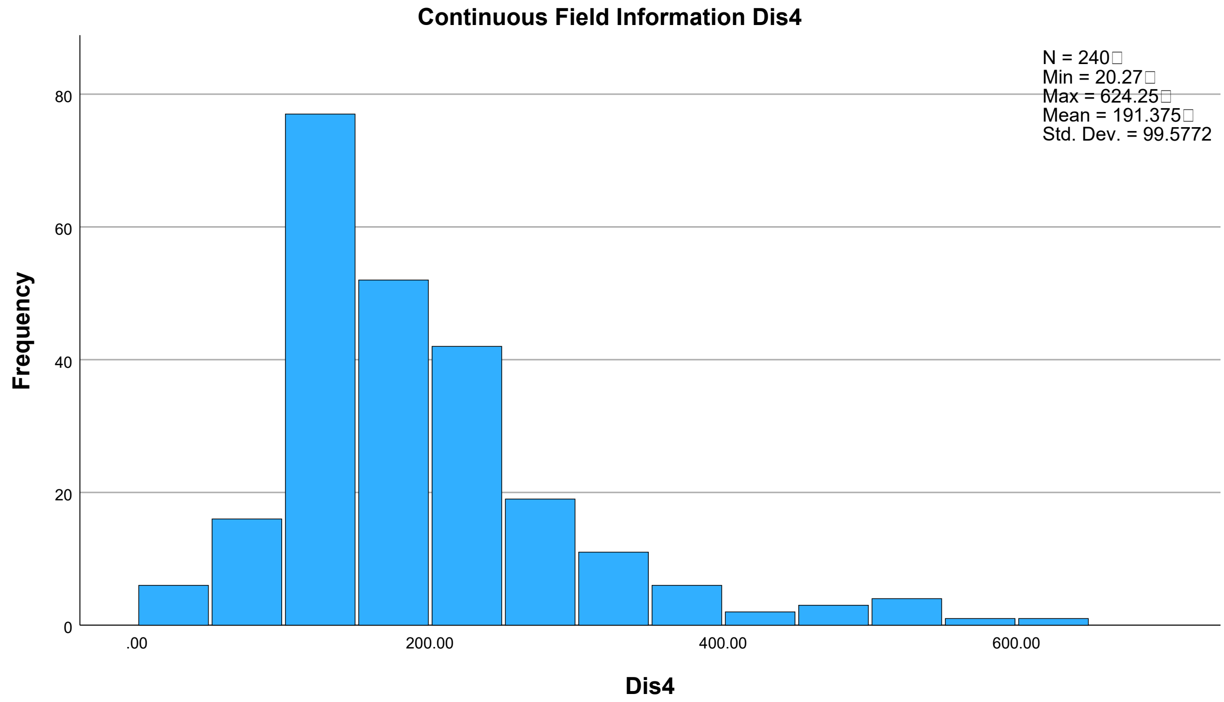
Continuous Field Information Dis4
N = 240
Min = 20.27
Max = 624.25
Mean = 191.375
Std. Dev. = 99.5772
Frequency
0
20
40
60
80
.00
200.00
400.00
600.00
Dis4

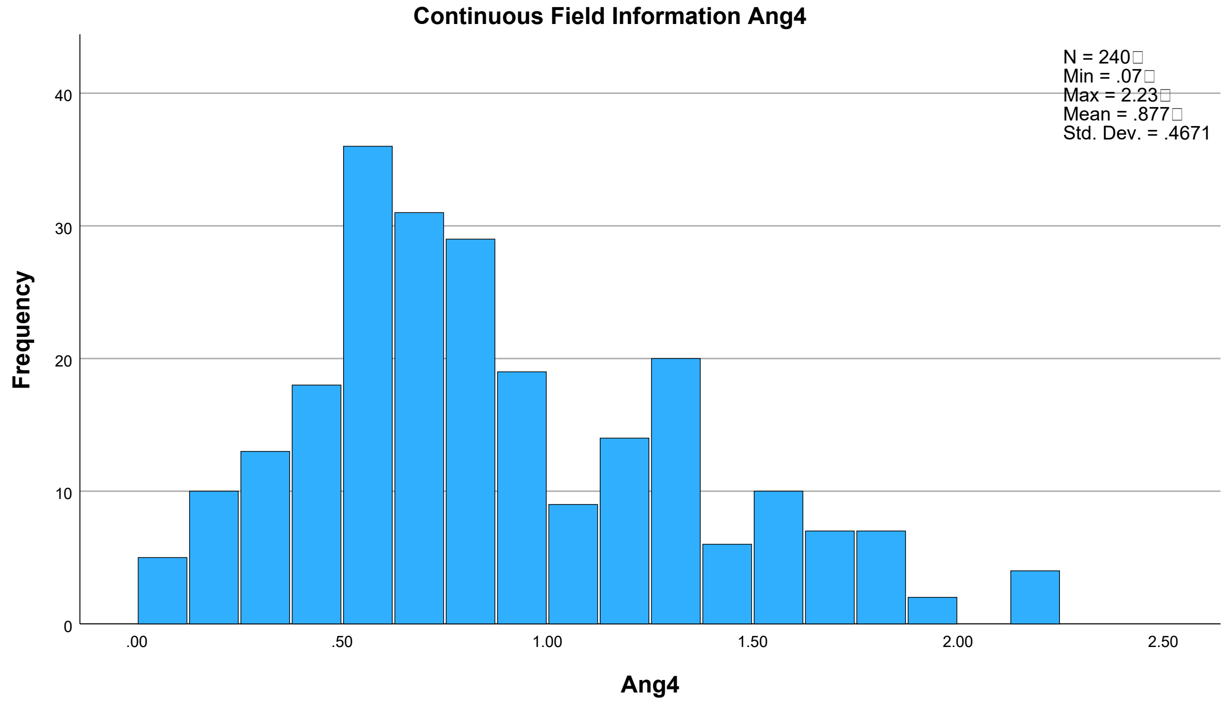
Continuous Field Information Ang4
N = 240
Min = .07
Max = 2.23
Mean = .877
Std. Dev. = .4671
Frequency
0
10
20
30
40
.00
.50
1.00
1.50
2.00
2.50
Ang4

Categorical Field Information S4

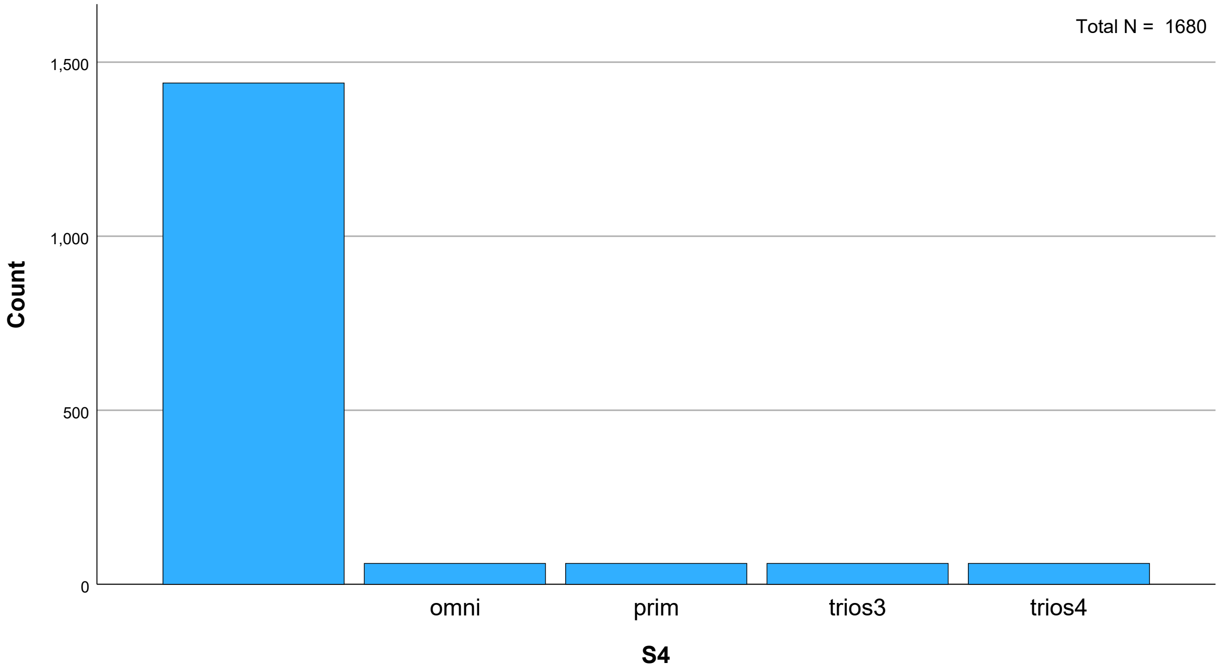

Nonparametric Tests

Notes

Output Created		30-MAY-2023 10:41:09
Comments		
Input	Data	\\Client\C$\SSPS\All3Ddis and ang.sav
	Active Dataset	DataSet2
	Filter	<none>
	Weight	<none>
	Split File	<none>
	N of Rows in Working Data File	1680
Syntax		NPTESTS /INDEPENDENT TEST (Dis1 Ang1) GROUP (S1) KRUSKAL_WALLIS (COMPARE=PAIRWISE) /MISSING SCOPE=ANALYSIS USERMISSING=EXCLUDE /CRITERIA ALPHA=0.05 CILEVEL=95.
Resources	Processor Time	00:00:01.28
	Elapsed Time	00:00:01.28

Hypothesis Test Summary

	Null Hypothesis	Test	Sig.[a,b]
1	The distribution of Dis1 is the same across categories of S1.	Independent-Samples Kruskal-Wallis Test	<.001
2	The distribution of Ang1 is the same across categories of S1.	Independent-Samples Kruskal-Wallis Test	<.001

Hypothesis Test Summary

	Decision
1	Reject the null hypothesis.
2	Reject the null hypothesis.

a. The significance level is .050.

b. Asymptotic significance is displayed.

Independent-Samples Kruskal-Wallis Test

Dis1 across S1

Independent-Samples Kruskal-Wallis Test Summary

Total N	240
Test Statistic	100.161[a]
Degree Of Freedom	3
Asymptotic Sig.(2-sided test)	<.001

a. The test statistic is adjusted for ties.

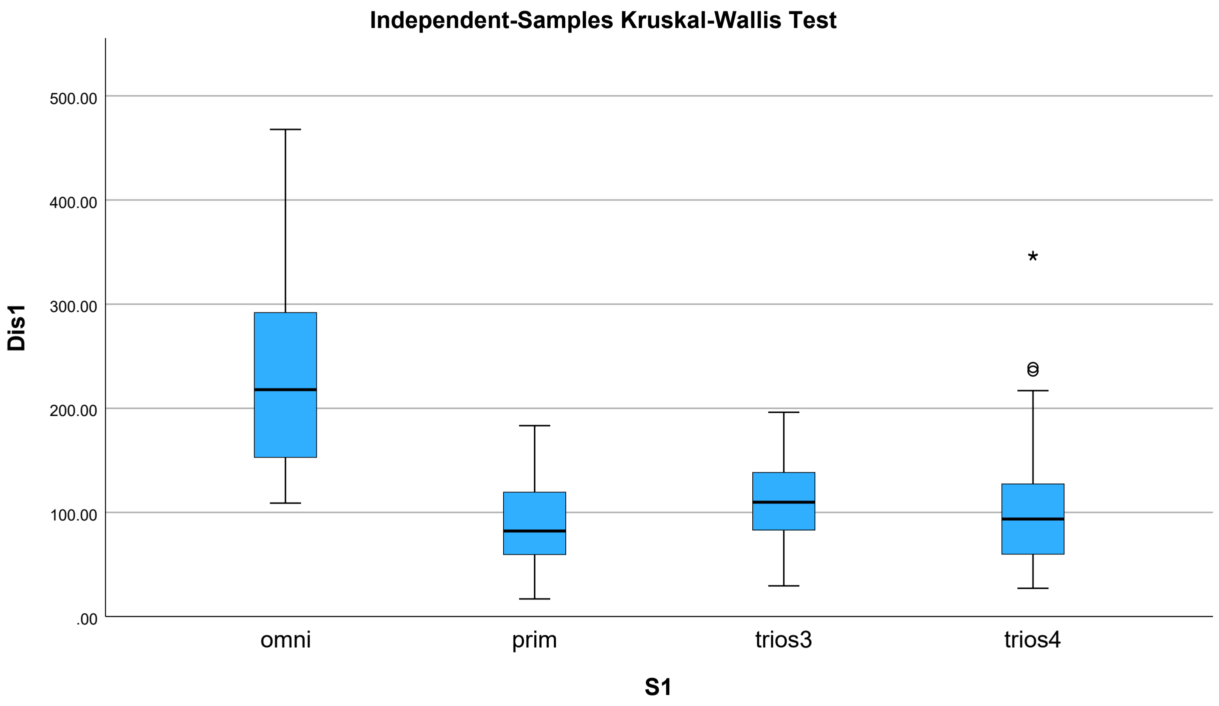

Pairwise Comparisons of S1

Sample 1-Sample 2	Test Statistic	Std. Error	Std. Test Statistic	Sig.	Adj. Sig.[a]
prim-trios4	-13.050	12.675	-1.030	.303	1.000
prim-trios3	-28.450	12.675	-2.244	.025	.149
prim-omni	114.767	12.675	9.054	<.001	.000
trios4-trios3	15.400	12.675	1.215	.224	1.000
trios4-omni	101.717	12.675	8.025	<.001	.000
trios3-omni	86.317	12.675	6.810	<.001	.000

Each row tests the null hypothesis that the Sample 1 and Sample 2 distributions are the same.
Asymptotic significances (2-sided tests) are displayed. The significance level is .050.

a. Significance values have been adjusted by the Bonferroni correction for multiple tests.

Pairwise Comparisons of S1

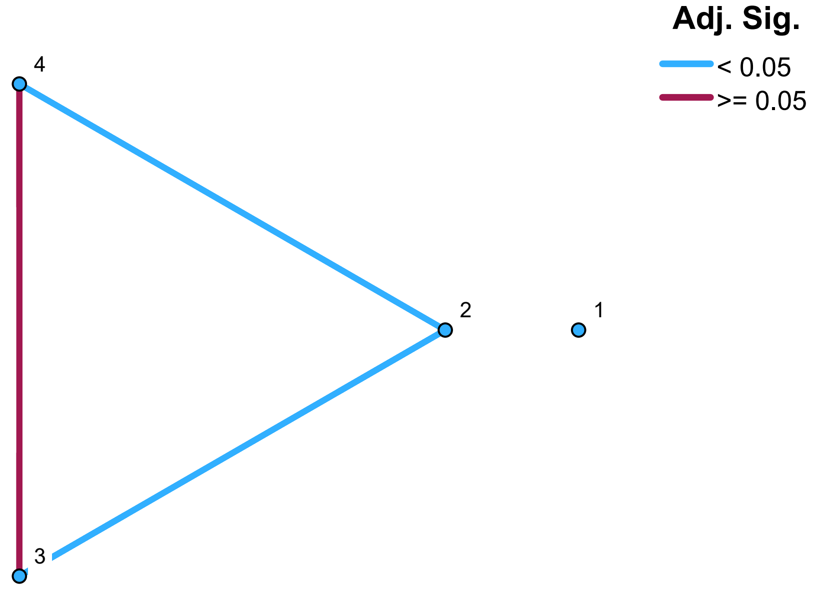

Each node shows the sample average rank of S1.

Ang1 across S1

Independent-Samples Kruskal-Wallis Test Summary

Total N	240
Test Statistic	44.823[a]
Degree Of Freedom	3
Asymptotic Sig.(2-sided test)	<.001

a. The test statistic is adjusted for ties.

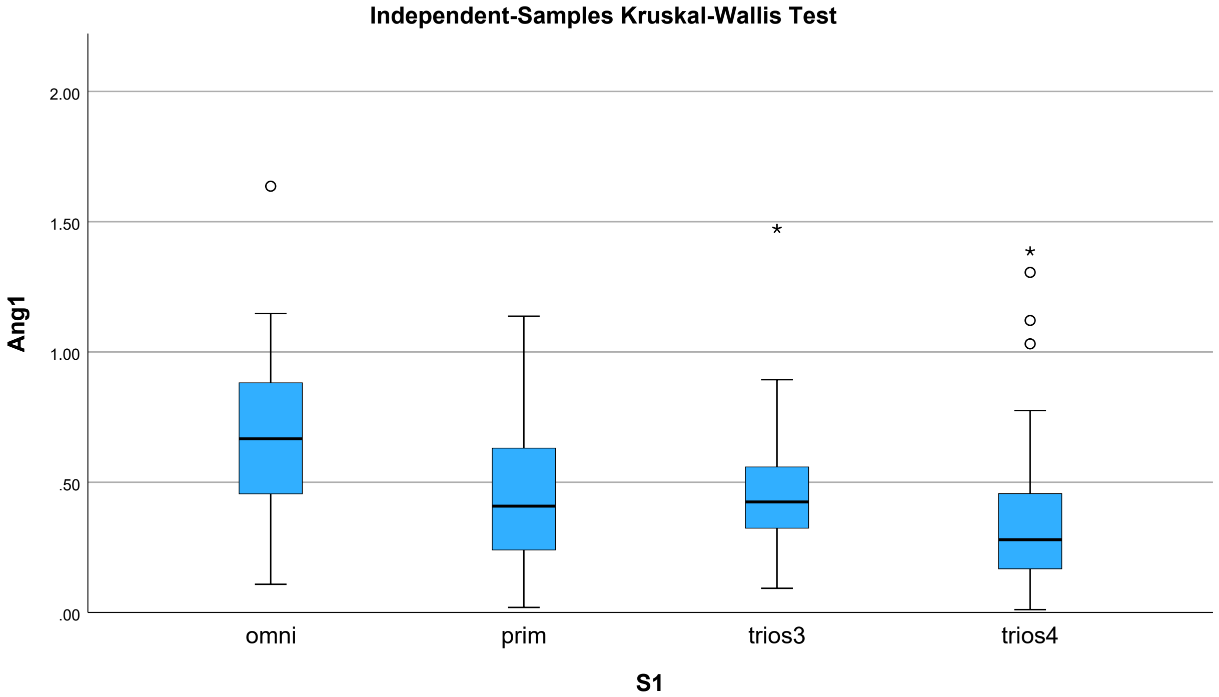

Pairwise Comparisons of S1

Sample 1-Sample 2	Test Statistic	Std. Error	Std. Test Statistic	Sig.	Adj. Sig.[a]
trios4-prim	33.633	12.675	2.653	.008	.048
trios4-trios3	36.667	12.675	2.893	.004	.023
trios4-omni	84.233	12.675	6.645	<.001	.000
prim-trios3	-3.033	12.675	-.239	.811	1.000
prim-omni	50.600	12.675	3.992	<.001	.000
trios3-omni	47.567	12.675	3.753	<.001	.001

Each row tests the null hypothesis that the Sample 1 and Sample 2 distributions are the same.
Asymptotic significances (2-sided tests) are displayed. The significance level is .050.

a. Significance values have been adjusted by the Bonferroni correction for multiple tests.

Pairwise Comparisons of S1

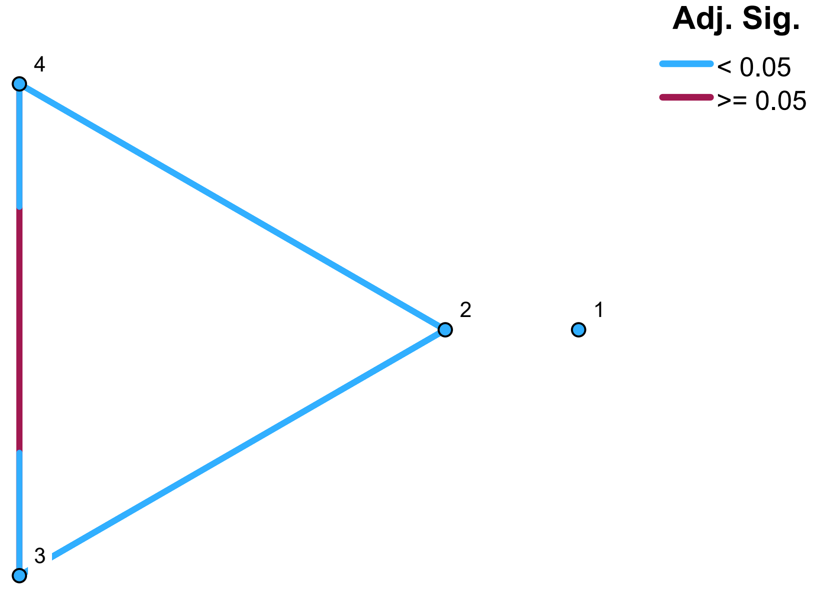

Each node shows the sample average rank of S1.

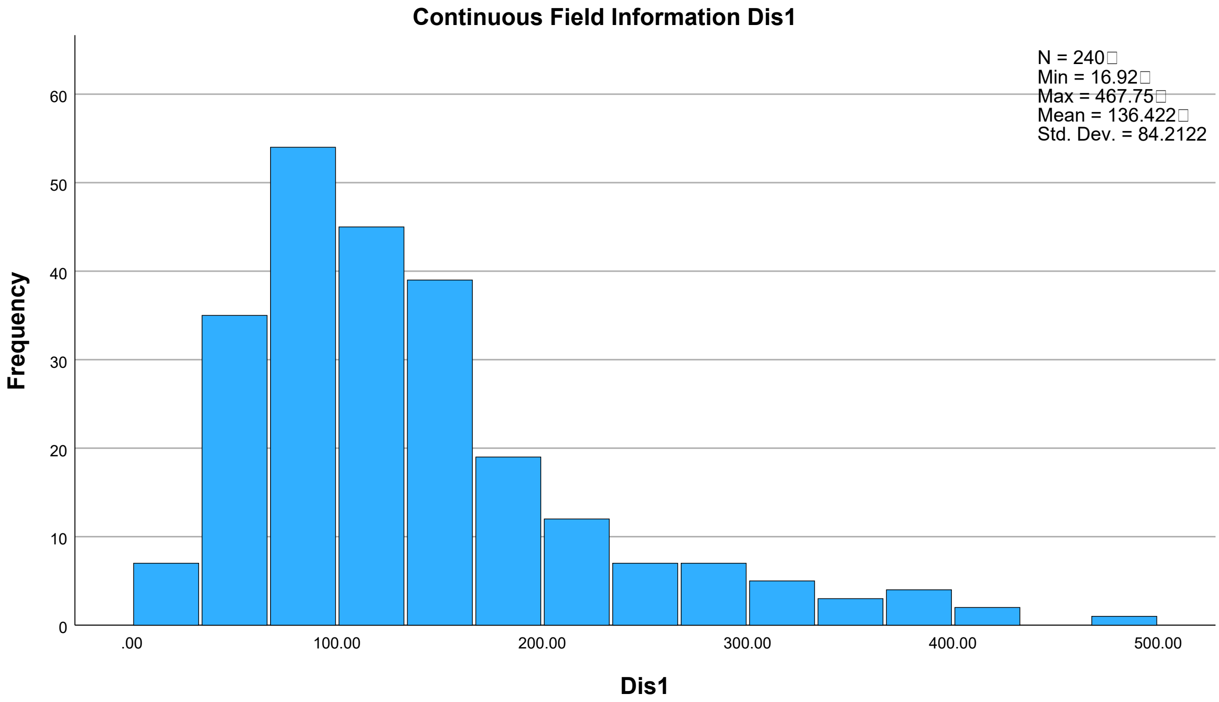
Continuous Field Information Dis1
N = 240
Min = 16.92
Max = 467.75
Mean = 136.422
Std. Dev. = 84.2122
Frequency
60
50
40
30
20
10
0
.00
100.00
200.00
300.00
400.00
500.00
Dis1

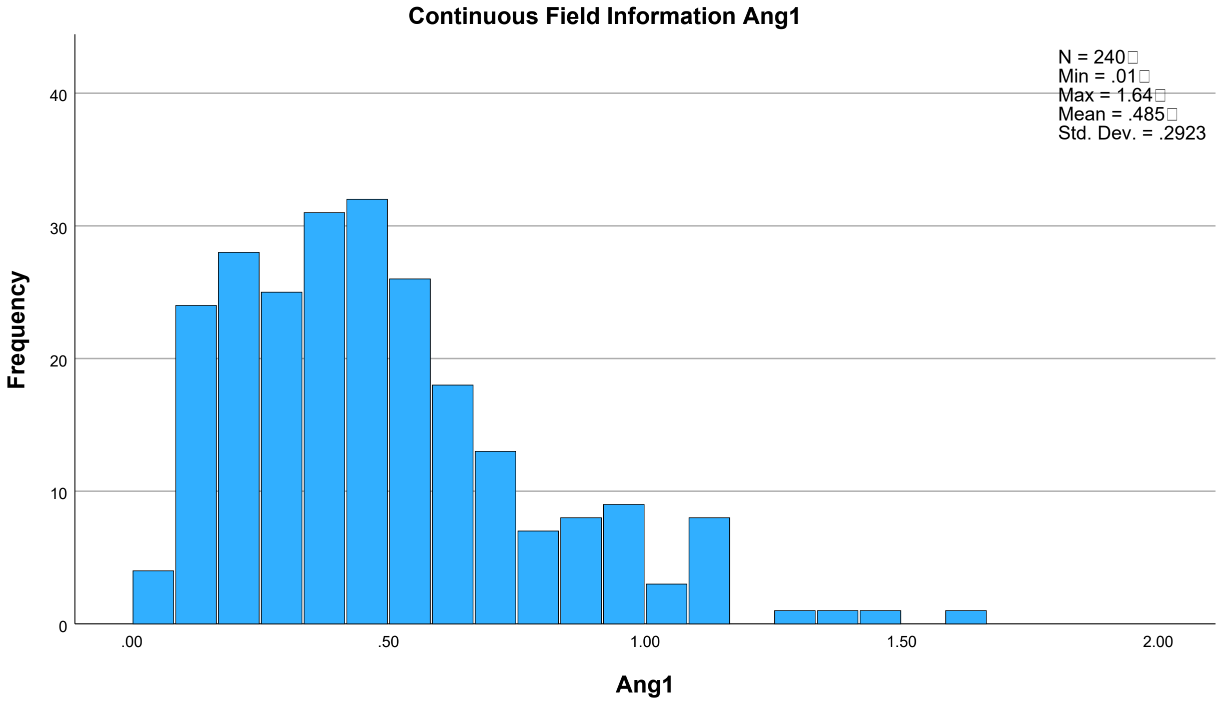
Continuous Field Information Ang1
N = 240
Min = .01
Max = 1.64
Mean = .485
Std. Dev. = .2923
Frequency
40
30
20
10
0
.00
.50
1.00
1.50
2.00
Ang1

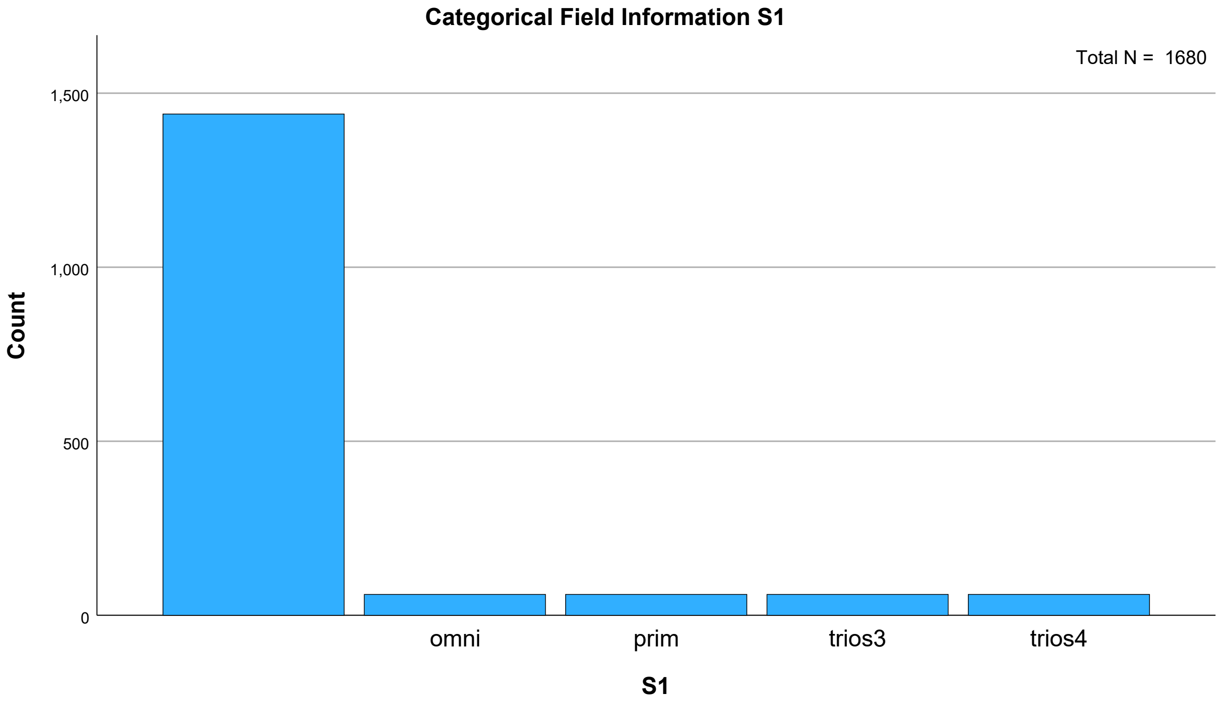

Categorical Field Information S1
Total N = 1680
1,500
1,000
500
0
Count
omni
prim
trios3
trios4
S1

Nonparametric Tests

Notes

Output Created		30-MAY-2023 10:41:55
Comments		
Input	Data	\\Client\C$\SSPS\All3Ddis and ang.sav
	Active Dataset	DataSet2
	Filter	<none>
	Weight	<none>
	Split File	<none>
	N of Rows in Working Data File	1680
Syntax		NPTESTS /INDEPENDENT TEST (Dis2 Ang2) GROUP (S2) KRUSKAL_WALLIS (COMPARE=PAIRWISE) /MISSING SCOPE=ANALYSIS USERMISSING=EXCLUDE /CRITERIA ALPHA=0.05 CILEVEL=95.
Resources	Processor Time	00:00:01.20
	Elapsed Time	00:00:01.28

Hypothesis Test Summary

	Null Hypothesis	Test	Sig.[a,b]
1	The distribution of Dis2 is the same across categories of S2.	Independent-Samples Kruskal-Wallis Test	<.001
2	The distribution of Ang2 is the same across categories of S2.	Independent-Samples Kruskal-Wallis Test	<.001

Hypothesis Test Summary

	Decision
1	Reject the null hypothesis.
2	Reject the null hypothesis.

a. The significance level is .050.

b. Asymptotic significance is displayed.

Independent-Samples Kruskal-Wallis Test

Dis2 across S2

Independent-Samples Kruskal-Wallis Test Summary

Total N	240
Test Statistic	121.050[a]
Degree Of Freedom	3
Asymptotic Sig.(2-sided test)	<.001

a. The test statistic is adjusted for ties.

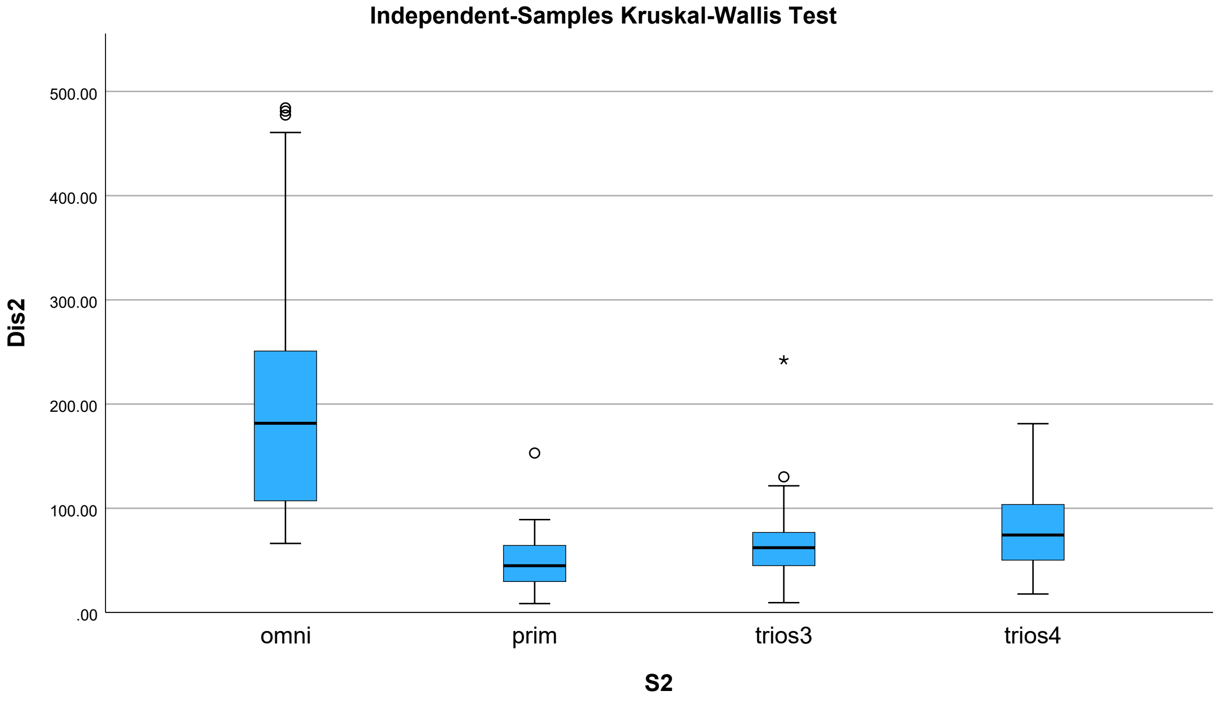

Pairwise Comparisons of S2

Sample 1-Sample 2	Test Statistic	Std. Error	Std. Test Statistic	Sig.	Adj. Sig.[a]
prim-trios3	-30.417	12.675	-2.400	.016	.098
prim-trios4	-55.850	12.675	-4.406	<.001	.000
prim-omni	133.067	12.675	10.498	<.001	.000
trios3-trios4	-25.433	12.675	-2.007	.045	.269
trios3-omni	102.650	12.675	8.098	<.001	.000
trios4-omni	77.217	12.675	6.092	<.001	.000

Each row tests the null hypothesis that the Sample 1 and Sample 2 distributions are the same.
Asymptotic significances (2-sided tests) are displayed. The significance level is .050.

a. Significance values have been adjusted by the Bonferroni correction for multiple tests.

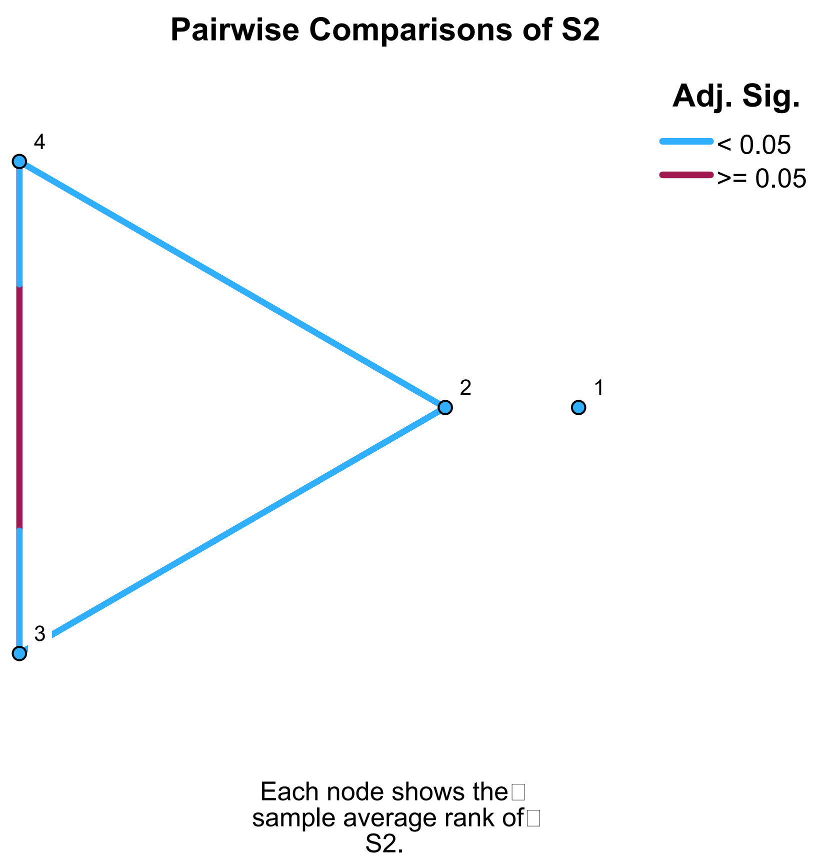

Ang2 across S2

Independent-Samples Kruskal-Wallis Test Summary

Total N	240
Test Statistic	91.405[a]
Degree Of Freedom	3
Asymptotic Sig.(2-sided test)	<.001

a. The test statistic is adjusted for ties.

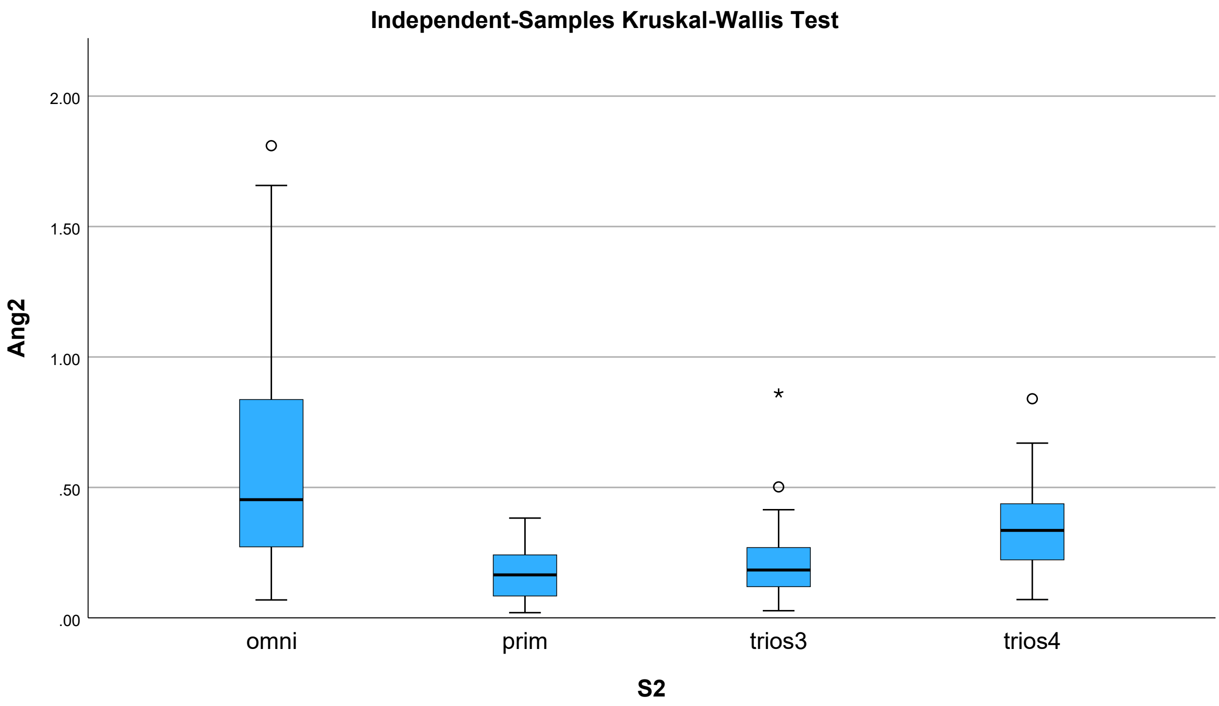

Pairwise Comparisons of S2

Sample 1-Sample 2	Test Statistic	Std. Error	Std. Test Statistic	Sig.	Adj. Sig.[a]
prim-trios3	-14.683	12.675	-1.158	.247	1.000
prim-trios4	-74.883	12.675	-5.908	<.001	.000
prim-omni	104.633	12.675	8.255	<.001	.000
trios3-trios4	-60.200	12.675	-4.749	<.001	.000
trios3-omni	89.950	12.675	7.096	<.001	.000
trios4-omni	29.750	12.675	2.347	.019	.114

Each row tests the null hypothesis that the Sample 1 and Sample 2 distributions are the same.
Asymptotic significances (2-sided tests) are displayed. The significance level is .050.

a. Significance values have been adjusted by the Bonferroni correction for multiple tests.

Pairwise Comparisons of S2

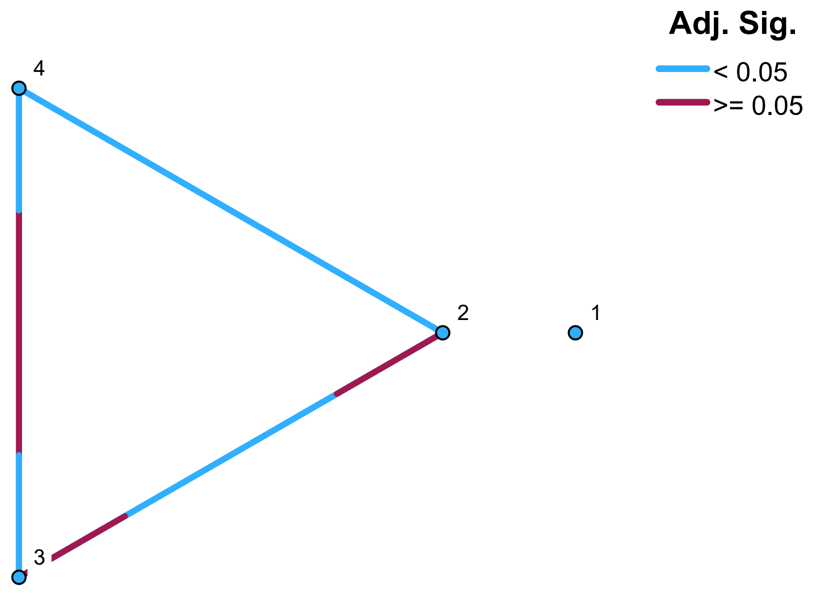

Each node shows the sample average rank of S2.

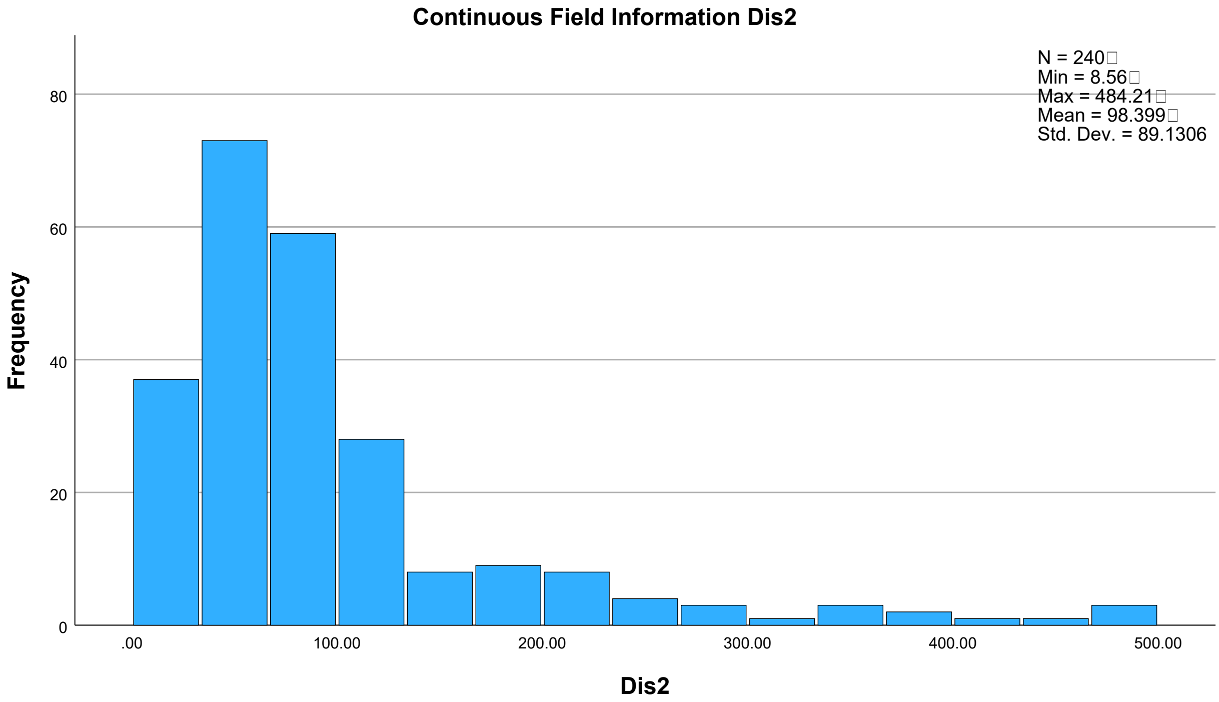
Continuous Field Information Dis2
N = 240
Min = 8.56
Max = 484.21
Mean = 98.399
Std. Dev. = 89.1306
Frequency
0
20
40
60
80
.00
100.00
200.00
300.00
400.00
500.00
Dis2

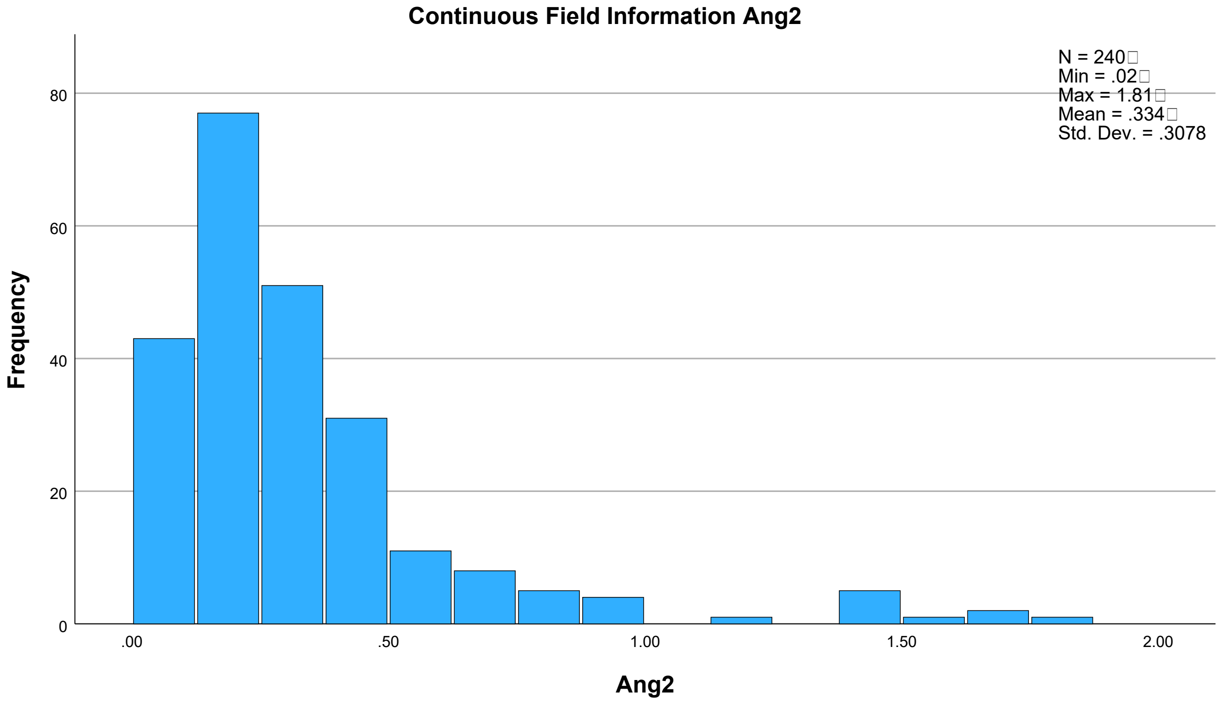
Continuous Field Information Ang2
N = 240
Min = .02
Max = 1.81
Mean = .334
Std. Dev. = .3078
Frequency
0
20
40
60
80
.00
.50
1.00
1.50
2.00
Ang2

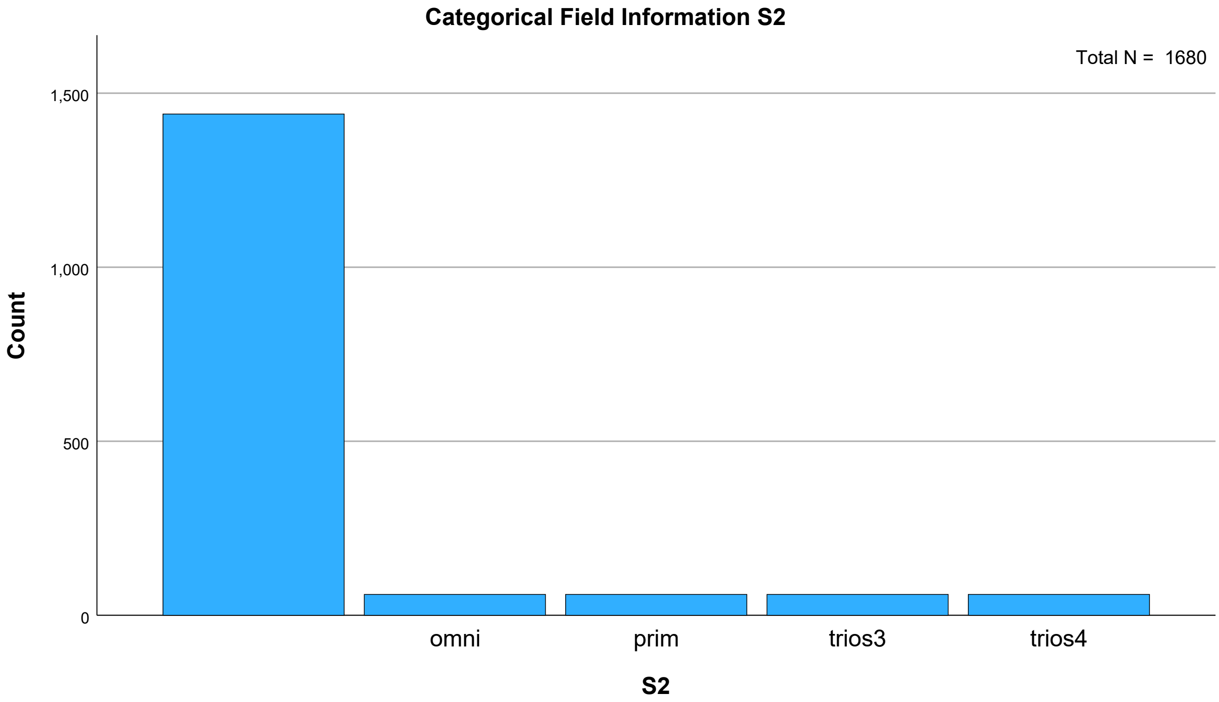

Categorical Field Information S2
Total N = 1680
1,500
1,000
500
0
Count
omni
prim
trios3
trios4
S2

Nonparametric Tests

Notes

Output Created		30-MAY-2023 10:51:16
Comments		
Input	Active Dataset	DataSet2
	Filter	<none>
	Weight	<none>
	Split File	<none>
	N of Rows in Working Data File	1680
Syntax		NPTESTS /INDEPENDENT TEST (Omni3d OminAng) GROUP (Omni) KRUSKAL_WALLIS (COMPARE=PAIRWISE) /MISSING SCOPE=ANALYSIS USERMISSING=EXCLUDE /CRITERIA ALPHA=0.05 CILEVEL=95.
Resources	Processor Time	00:00:01.36
	Elapsed Time	00:00:01.22

Hypothesis Test Summary

	Null Hypothesis	Test	Sig.[a,b]
1	The distribution of Omni3d is the same across categories of Omni.	Independent-Samples Kruskal-Wallis Test	<.001
2	The distribution of OminAng is the same across categories of Omni.	Independent-Samples Kruskal-Wallis Test	<.001

Hypothesis Test Summary

	Decision
1	Reject the null hypothesis.
2	Reject the null hypothesis.

a. The significance level is .050.

b. Asymptotic significance is displayed.

Independent-Samples Kruskal-Wallis Test

Omni3d across Omni

Independent-Samples Kruskal-Wallis Test Summary

Total N	420
Test Statistic	85.674[a]
Degree Of Freedom	6
Asymptotic Sig.(2-sided test)	<.001

a. The test statistic is adjusted for ties.

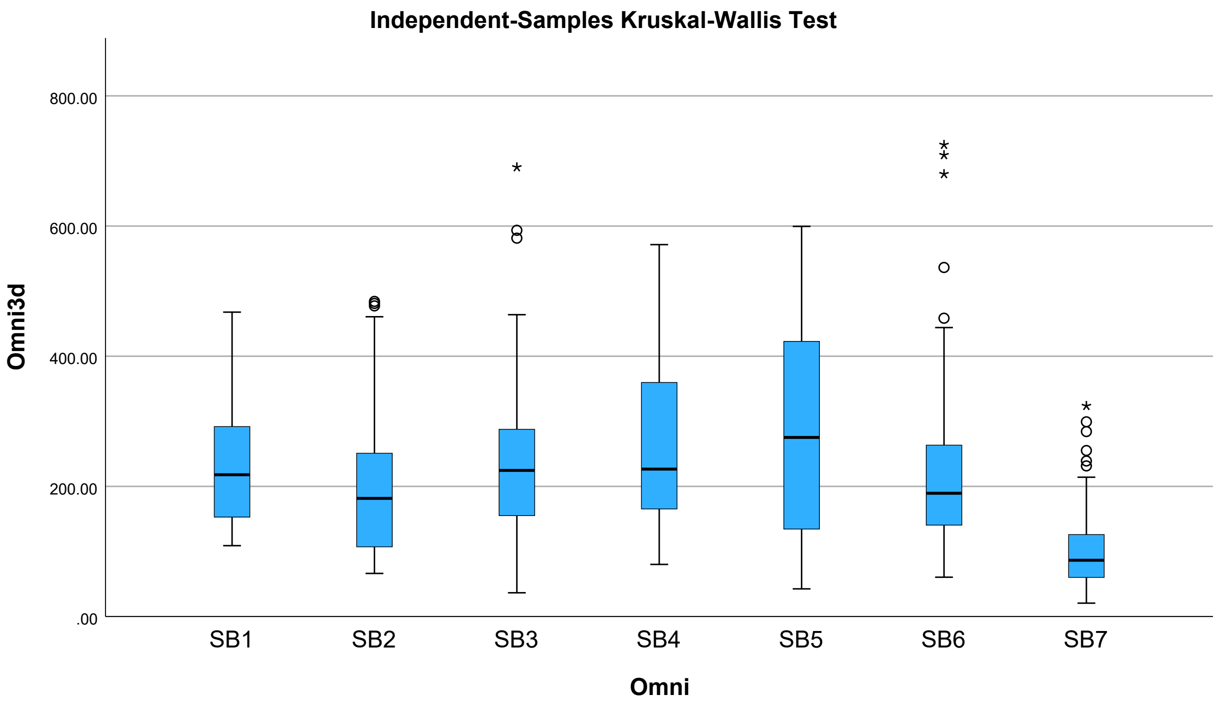

Pairwise Comparisons of Omni

Sample 1-Sample 2	Test Statistic	Std. Error	Std. Test Statistic	Sig.	Adj. Sig.[a]
SB7-SB2	102.525	22.162	4.626	<.001	.000
SB7-SB6	128.700	22.162	5.807	<.001	.000
SB7-SB3	147.200	22.162	6.642	<.001	.000
SB7-SB1	152.250	22.162	6.870	<.001	.000
SB7-SB4	167.817	22.162	7.572	<.001	.000
SB7-SB5	167.875	22.162	7.575	<.001	.000
SB2-SB6	-26.175	22.162	-1.181	.238	1.000
SB2-SB3	-44.675	22.162	-2.016	.044	.920
SB2-SB1	49.725	22.162	2.244	.025	.522
SB2-SB4	-65.292	22.162	-2.946	.003	.068
SB2-SB5	-65.350	22.162	-2.949	.003	.067
SB6-SB3	18.500	22.162	.835	.404	1.000
SB6-SB1	23.550	22.162	1.063	.288	1.000
SB6-SB4	39.117	22.162	1.765	.078	1.000
SB6-SB5	39.175	22.162	1.768	.077	1.000
SB3-SB1	5.050	22.162	.228	.820	1.000
SB3-SB4	-20.617	22.162	-.930	.352	1.000
SB3-SB5	-20.675	22.162	-.933	.351	1.000
SB1-SB4	-15.567	22.162	-.702	.482	1.000
SB1-SB5	-15.625	22.162	-.705	.481	1.000
SB4-SB5	-.058	22.162	-.003	.998	1.000

Each row tests the null hypothesis that the Sample 1 and Sample 2 distributions are the same.
Asymptotic significances (2-sided tests) are displayed. The significance level is .050.

a. Significance values have been adjusted by the Bonferroni correction for multiple tests.

Pairwise Comparisons of Omni

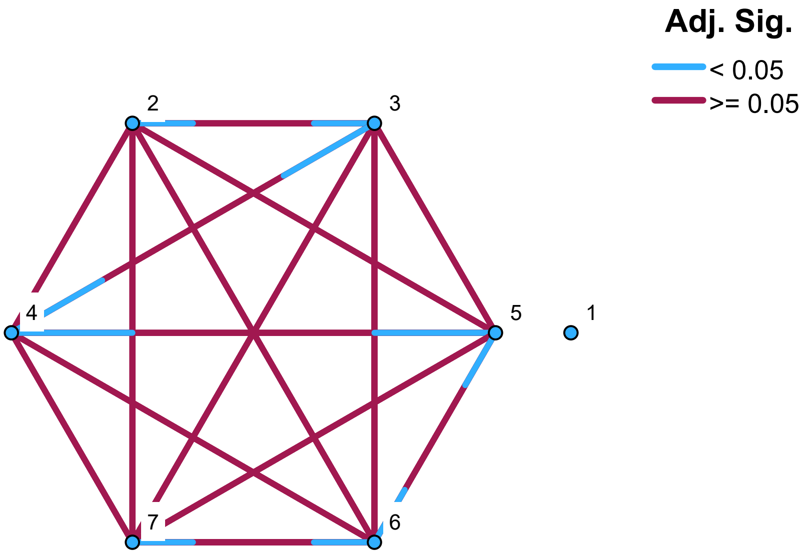

Each node shows the
sample average rank of
Omni.

OminAng across Omni

Independent-Samples Kruskal-Wallis Test Summary

Total N	420
Test Statistic	71.729[a]
Degree Of Freedom	6
Asymptotic Sig.(2-sided test)	<.001

a. The test statistic is adjusted for ties.

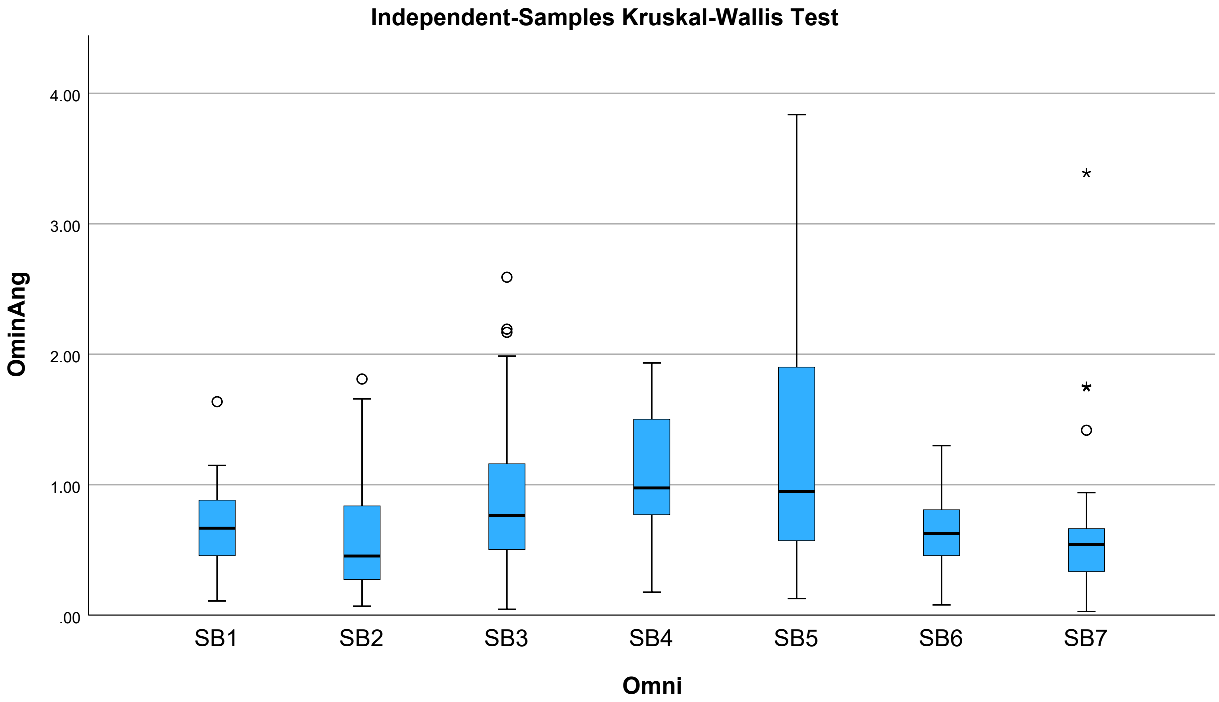

Pairwise Comparisons of Omni

Sample 1-Sample 2	Test Statistic	Std. Error	Std. Test Statistic	Sig.	Adj. Sig.[a]
SB7-SB2	2.833	22.162	.128	.898	1.000
SB7-SB6	28.550	22.162	1.288	.198	1.000
SB7-SB1	38.933	22.162	1.757	.079	1.000
SB7-SB3	78.900	22.162	3.560	<.001	.008
SB7-SB5	121.667	22.162	5.490	<.001	.000
SB7-SB4	132.083	22.162	5.960	<.001	.000
SB2-SB6	-25.717	22.162	-1.160	.246	1.000
SB2-SB1	36.100	22.162	1.629	.103	1.000
SB2-SB3	-76.067	22.162	-3.432	<.001	.013
SB2-SB5	-118.833	22.162	-5.362	<.001	.000
SB2-SB4	-129.250	22.162	-5.832	<.001	.000
SB6-SB1	10.383	22.162	.469	.639	1.000
SB6-SB3	50.350	22.162	2.272	.023	.485
SB6-SB5	93.117	22.162	4.202	<.001	.001
SB6-SB4	103.533	22.162	4.672	<.001	.000
SB1-SB3	-39.967	22.162	-1.803	.071	1.000
SB1-SB5	-82.733	22.162	-3.733	<.001	.004
SB1-SB4	-93.150	22.162	-4.203	<.001	.001

Pairwise Comparisons of Omni

Sample 1-Sample 2	Test Statistic	Std. Error	Std. Test Statistic	Sig.	Adj. Sig.[a]
SB3-SB5	-42.767	22.162	-1.930	.054	1.000
SB3-SB4	-53.183	22.162	-2.400	.016	.345
SB5-SB4	10.417	22.162	.470	.638	1.000

Each row tests the null hypothesis that the Sample 1 and Sample 2 distributions are the same.
Asymptotic significances (2-sided tests) are displayed. The significance level is .050.

a. Significance values have been adjusted by the Bonferroni correction for multiple tests.

Pairwise Comparisons of Omni

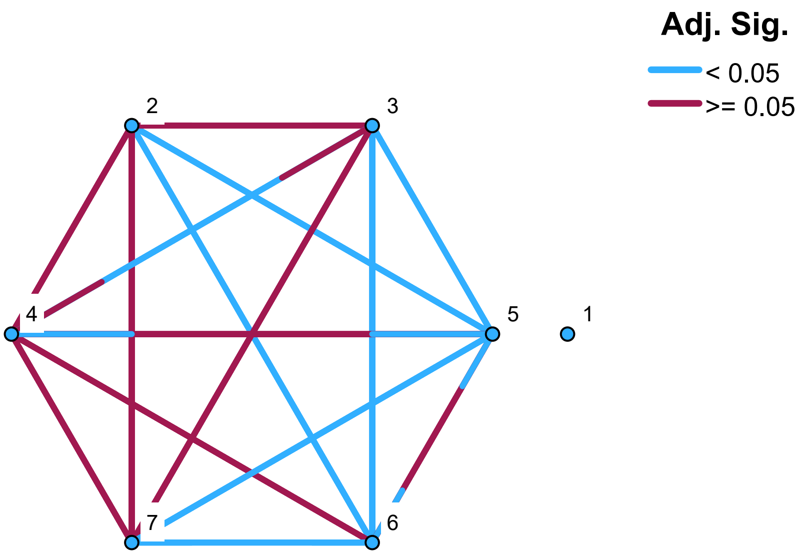

Each node shows the sample average rank of Omni.

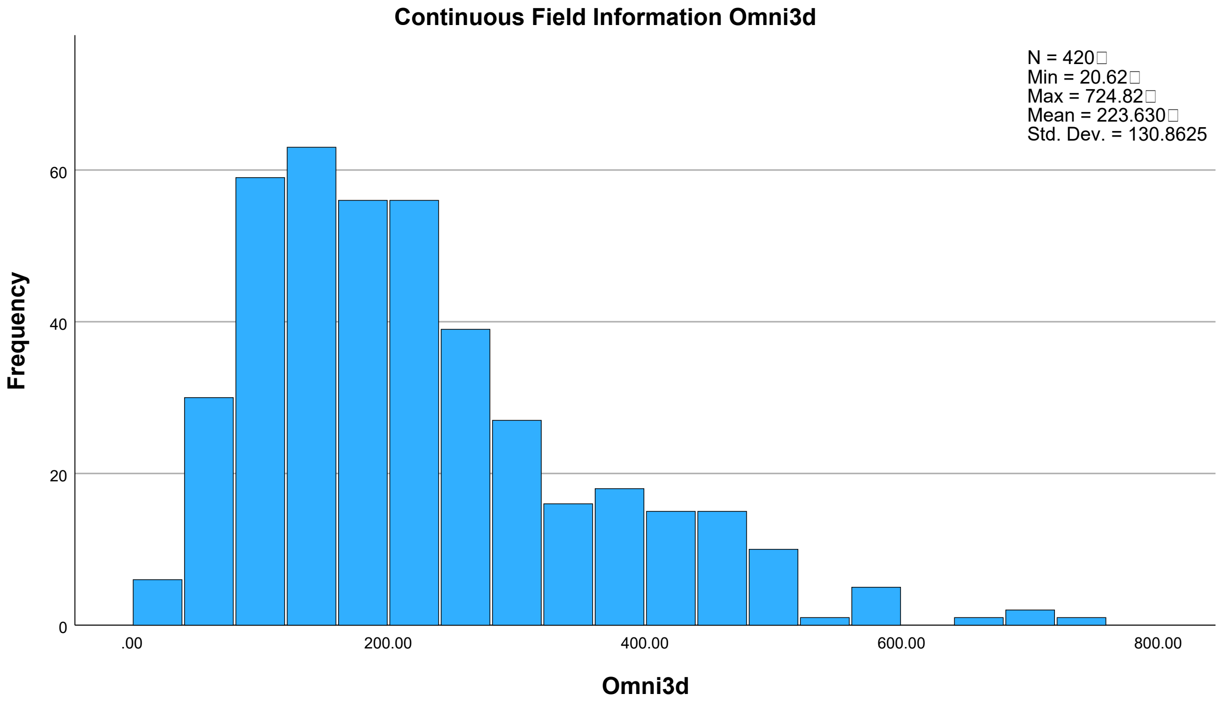
Continuous Field Information Omni3d
N = 420
Min = 20.62
Max = 724.82
Mean = 223.630
Std. Dev. = 130.8625
Frequency
0
20
40
60
.00
200.00
400.00
600.00
800.00
Omni3d

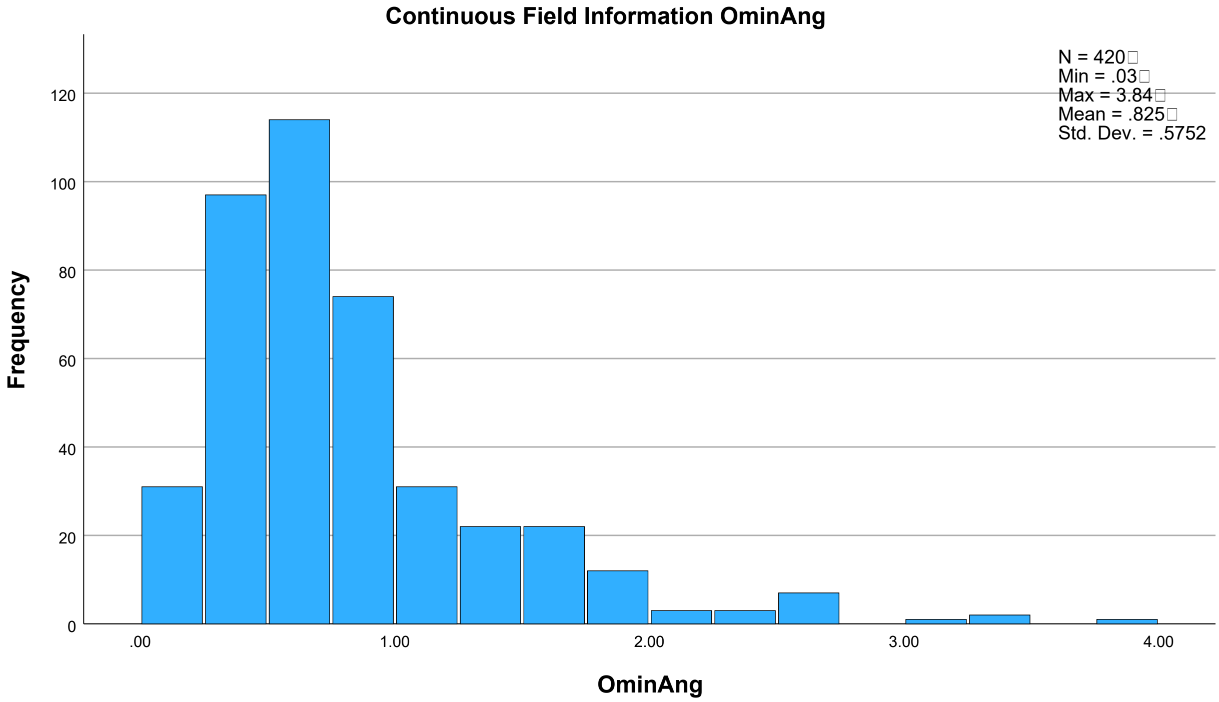
Continuous Field Information OminAng
N = 420
Min = .03
Max = 3.84
Mean = .825
Std. Dev. = .5752
Frequency
0
20
40
60
80
100
120
.00
1.00
2.00
3.00
4.00
OminAng

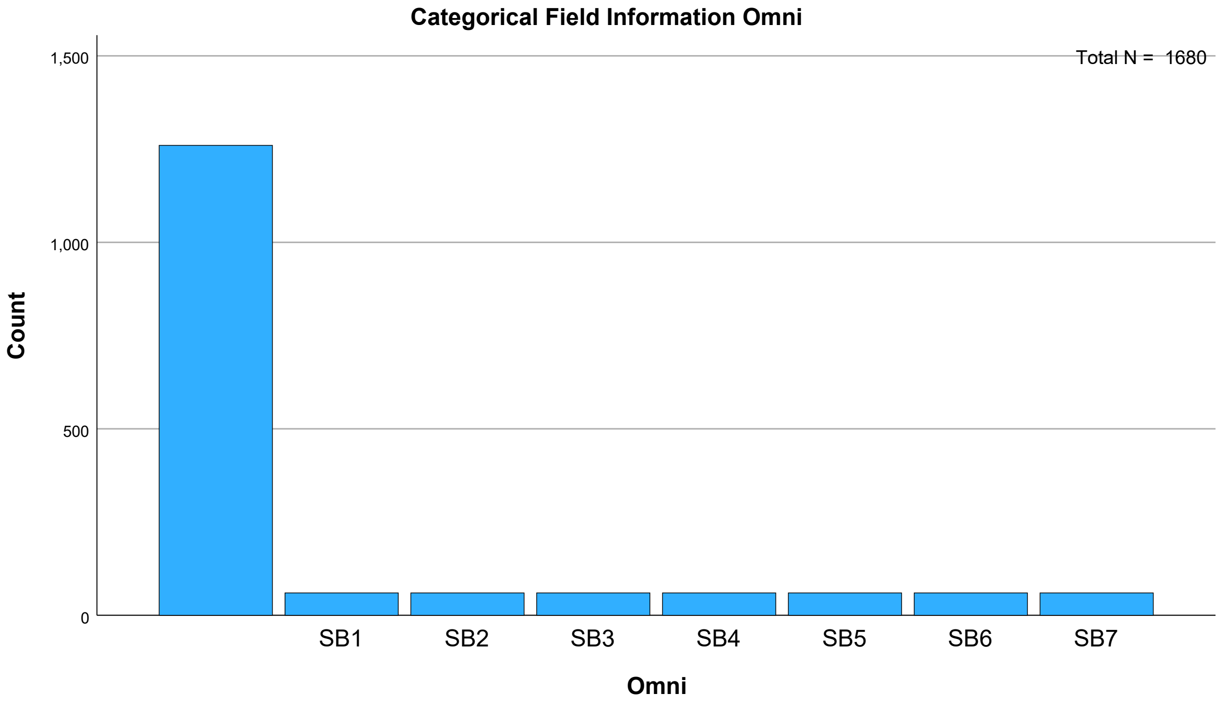
Categorical Field Information Omni
Total N = 1680
1,500
1,000
500
0
Count
SB1
SB2
SB3
SB4
SB5
SB6
SB7
Omni

Nonparametric Tests

Notes

Output Created		30-MAY-2023 10:50:27
Comments		
Input	Active Dataset	DataSet2
	Filter	<none>
	Weight	<none>
	Split File	<none>
	N of Rows in Working Data File	1680
Syntax		NPTESTS /INDEPENDENT TEST (Prime3d primeang) GROUP (Prime) KRUSKAL_WALLIS (COMPARE=PAIRWISE) /MISSING SCOPE=ANALYSIS USERMISSING=EXCLUDE /CRITERIA ALPHA=0.05 CILEVEL=95.
Resources	Processor Time	00:00:01.69
	Elapsed Time	00:00:01.25

Hypothesis Test Summary

	Null Hypothesis	Test	Sig.[a,b]
1	The distribution of Prime3d is the same across categories of Prime.	Independent-Samples Kruskal-Wallis Test	<.001
2	The distribution of primeang is the same across categories of Prime.	Independent-Samples Kruskal-Wallis Test	<.001

Hypothesis Test Summary

	Decision
1	Reject the null hypothesis.
2	Reject the null hypothesis.

a. The significance level is .050.

b. Asymptotic significance is displayed.

Independent-Samples Kruskal-Wallis Test

Prime3d across Prime

Independent-Samples Kruskal-Wallis Test Summary

Total N	420
Test Statistic	193.719[a]
Degree Of Freedom	6
Asymptotic Sig.(2-sided test)	<.001

a. The test statistic is adjusted for ties.

Independent-Samples Kruskal-Wallis Test

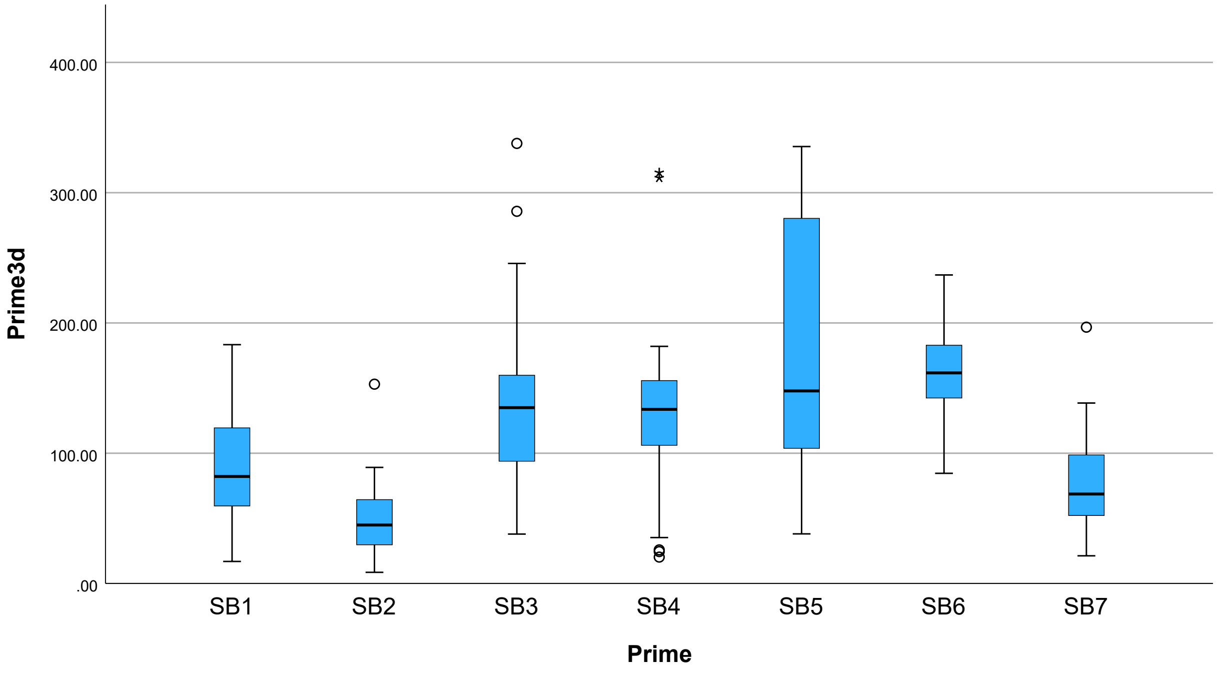

Pairwise Comparisons of Prime

Sample 1-Sample 2	Test Statistic	Std. Error	Std. Test Statistic	Sig.	Adj. Sig.[a]
SB2-SB7	-62.733	22.162	-2.831	.005	.098
SB2-SB1	99.867	22.162	4.506	<.001	.000
SB2-SB4	-172.433	22.162	-7.780	<.001	.000
SB2-SB3	-181.933	22.162	-8.209	<.001	.000
SB2-SB5	-214.517	22.162	-9.679	<.001	.000
SB2-SB6	-249.683	22.162	-11.266	<.001	.000
SB7-SB1	37.133	22.162	1.676	.094	1.000
SB7-SB4	109.700	22.162	4.950	<.001	.000
SB7-SB3	119.200	22.162	5.379	<.001	.000
SB7-SB5	151.783	22.162	6.849	<.001	.000
SB7-SB6	186.950	22.162	8.436	<.001	.000
SB1-SB4	-72.567	22.162	-3.274	.001	.022
SB1-SB3	-82.067	22.162	-3.703	<.001	.004
SB1-SB5	-114.650	22.162	-5.173	<.001	.000
SB1-SB6	-149.817	22.162	-6.760	<.001	.000
SB4-SB3	9.500	22.162	.429	.668	1.000
SB4-SB5	-42.083	22.162	-1.899	.058	1.000
SB4-SB6	-77.250	22.162	-3.486	<.001	.010
SB3-SB5	-32.583	22.162	-1.470	.142	1.000
SB3-SB6	-67.750	22.162	-3.057	.002	.047
SB5-SB6	-35.167	22.162	-1.587	.113	1.000

Each row tests the null hypothesis that the Sample 1 and Sample 2 distributions are the same.
Asymptotic significances (2-sided tests) are displayed. The significance level is .050.

a. Significance values have been adjusted by the Bonferroni correction for multiple tests.

Pairwise Comparisons of Prime

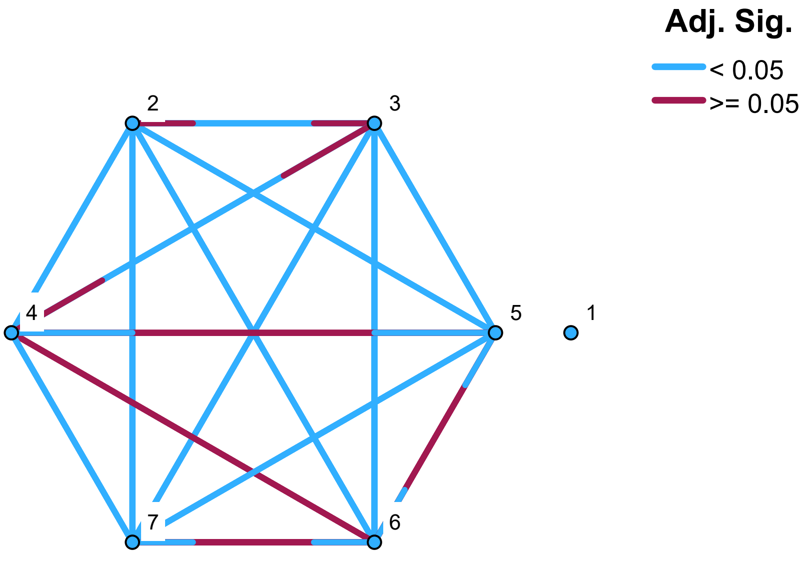

Each node shows the
sample average rank of
Prime.

primeang across Prime

Independent-Samples Kruskal-Wallis Test Summary

Total N	420
Test Statistic	162.880[a]
Degree Of Freedom	6
Asymptotic Sig.(2-sided test)	<.001

a. The test statistic is adjusted for ties.

Independent-Samples Kruskal-Wallis Test

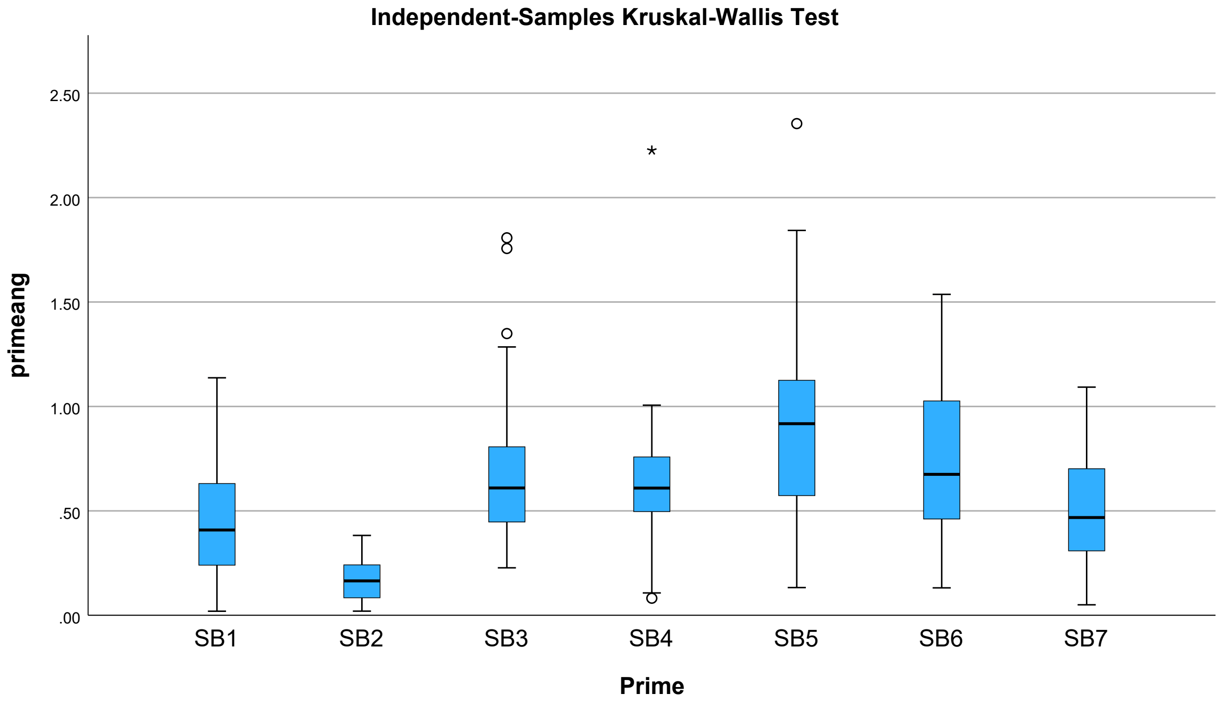

Pairwise Comparisons of Prime

Sample 1-Sample 2	Test Statistic	Std. Error	Std. Test Statistic	Sig.	Adj. Sig.[a]
SB2-SB1	122.367	22.162	5.521	<.001	.000
SB2-SB7	-142.283	22.162	-6.420	<.001	.000
SB2-SB4	-192.117	22.162	-8.669	<.001	.000
SB2-SB3	-196.667	22.162	-8.874	<.001	.000
SB2-SB6	-212.417	22.162	-9.585	<.001	.000
SB2-SB5	-246.917	22.162	-11.141	<.001	.000
SB1-SB7	-19.917	22.162	-.899	.369	1.000
SB1-SB4	-69.750	22.162	-3.147	.002	.035
SB1-SB3	-74.300	22.162	-3.353	<.001	.017
SB1-SB6	-90.050	22.162	-4.063	<.001	.001
SB1-SB5	-124.550	22.162	-5.620	<.001	.000
SB7-SB4	49.833	22.162	2.249	.025	.515
SB7-SB3	54.383	22.162	2.454	.014	.297
SB7-SB6	70.133	22.162	3.165	.002	.033
SB7-SB5	104.633	22.162	4.721	<.001	.000
SB4-SB3	4.550	22.162	.205	.837	1.000
SB4-SB6	-20.300	22.162	-.916	.360	1.000
SB4-SB5	-54.800	22.162	-2.473	.013	.282

Pairwise Comparisons of Prime

Sample 1-Sample 2	Test Statistic	Std. Error	Std. Test Statistic	Sig.	Adj. Sig.[a]
SB3-SB6	-15.750	22.162	-.711	.477	1.000
SB3-SB5	-50.250	22.162	-2.267	.023	.491
SB6-SB5	34.500	22.162	1.557	.120	1.000

Each row tests the null hypothesis that the Sample 1 and Sample 2 distributions are the same.
Asymptotic significances (2-sided tests) are displayed. The significance level is .050.

a. Significance values have been adjusted by the Bonferroni correction for multiple tests.

Pairwise Comparisons of Prime

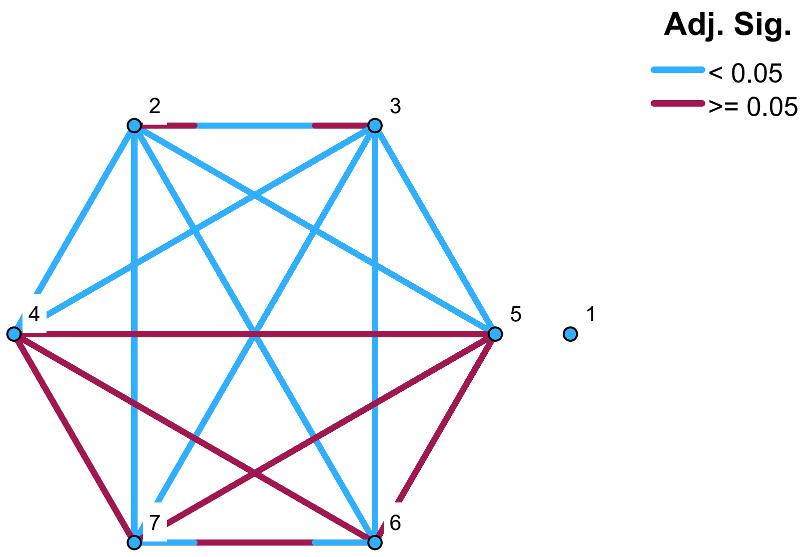

Each node shows the sample average rank of Prime.

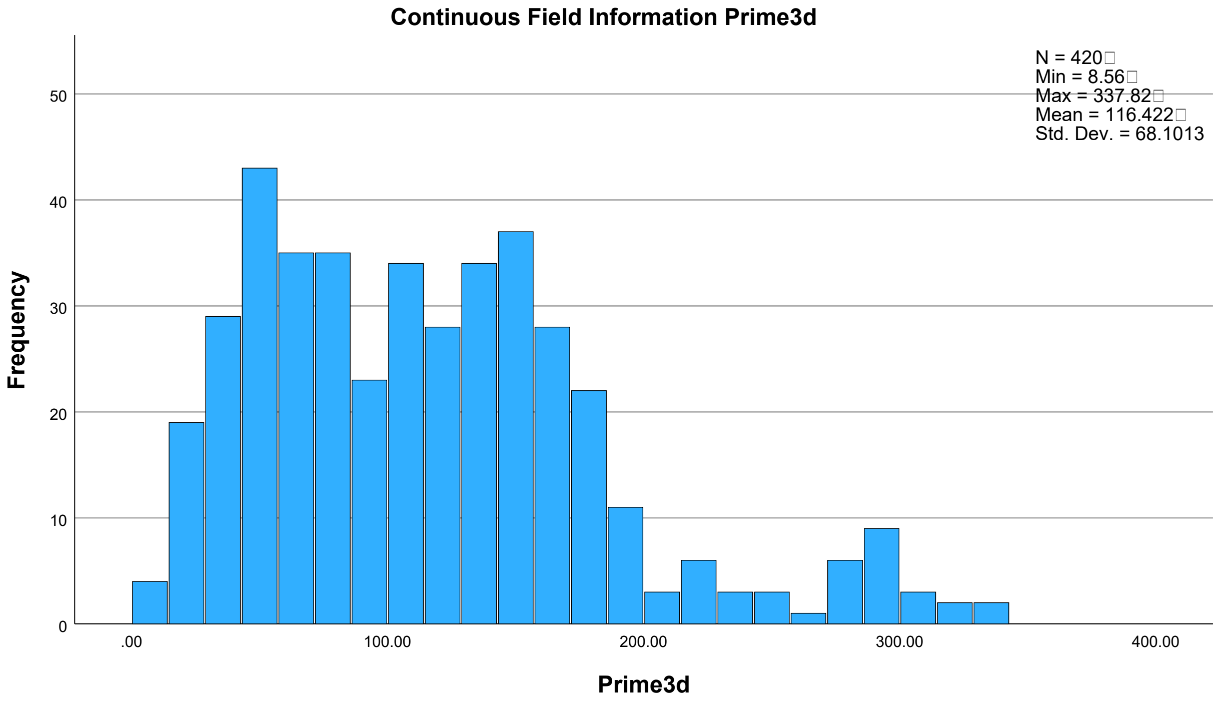
Continuous Field Information Prime3d
N = 420
Min = 8.56
Max = 337.82
Mean = 116.422
Std. Dev. = 68.1013
Frequency
0
10
20
30
40
50
.00
100.00
200.00
300.00
400.00
Prime3d

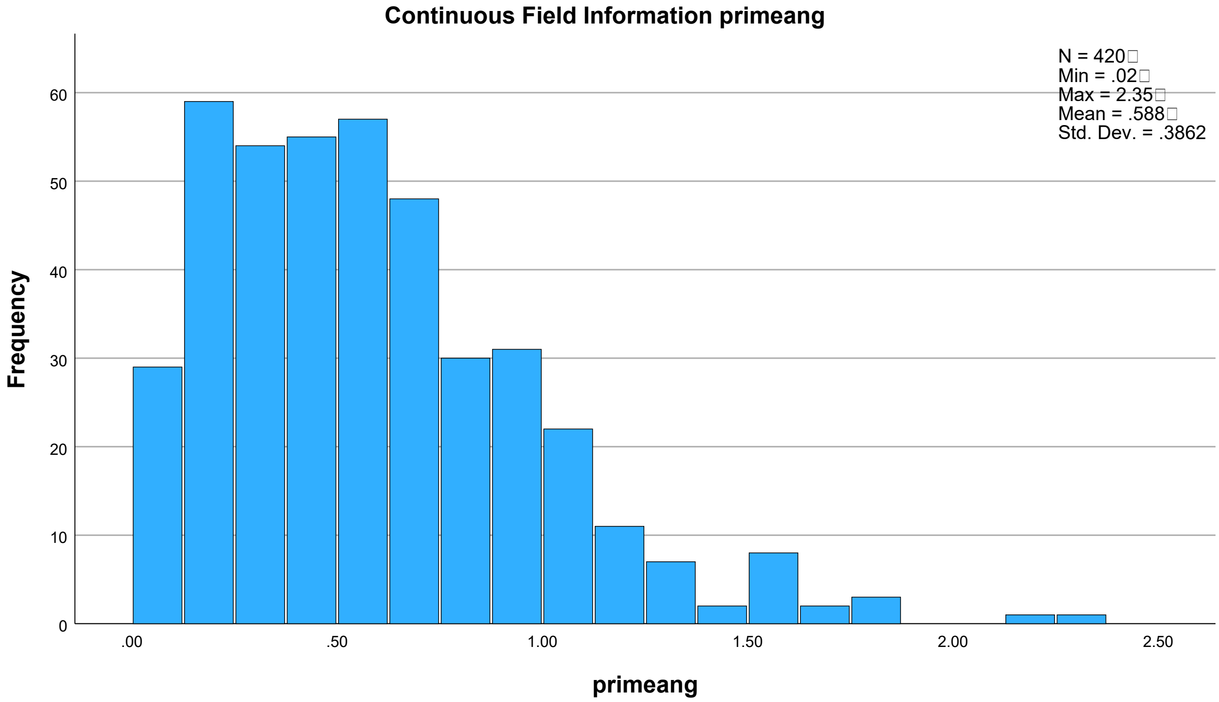
Continuous Field Information primeang
N = 420
Min = .02
Max = 2.35
Mean = .588
Std. Dev. = .3862
Frequency
0
10
20
30
40
50
60
.00
.50
1.00
1.50
2.00
2.50
primeang

Categorical Field Information Prime

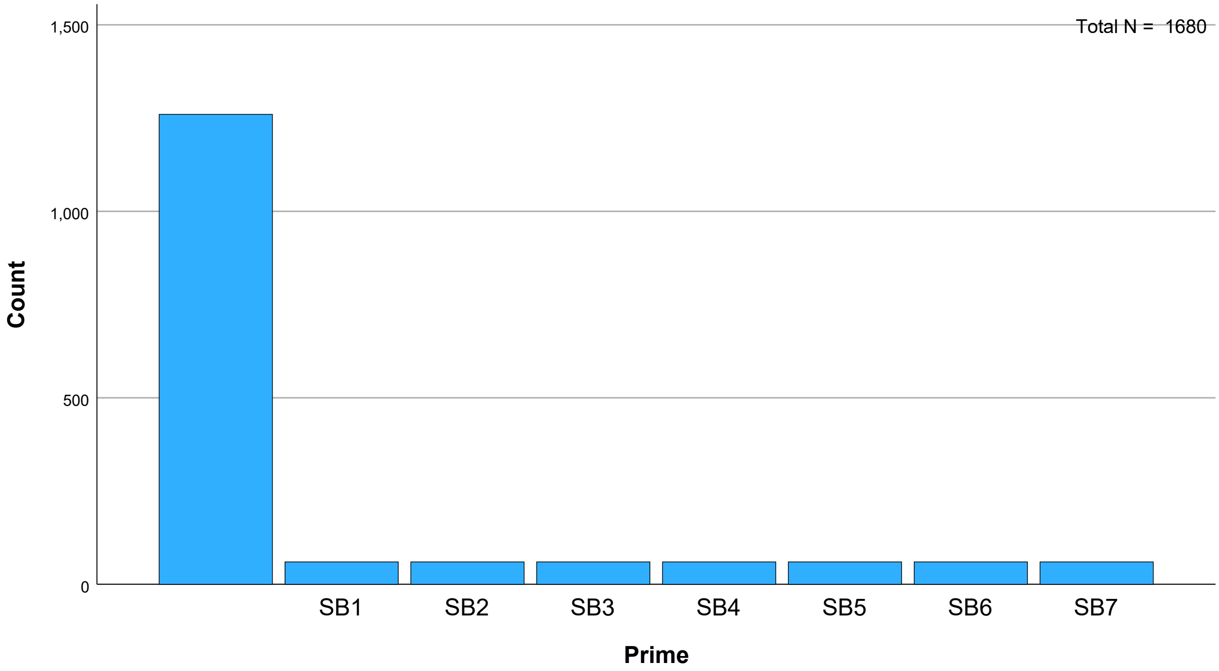

Nonparametric Tests

Notes

Output Created		30-MAY-2023 10:45:07
Comments		
Input	Data	\\Client\C$\SSPS\All3Ddis and ang.sav
	Active Dataset	DataSet2
	Filter	<none>
	Weight	<none>
	Split File	<none>
	N of Rows in Working Data File	1680
Syntax		NPTESTS /INDEPENDENT TEST (Dis5 Ang5) GROUP (S5) KRUSKAL_WALLIS (COMPARE=PAIRWISE) /MISSING SCOPE=ANALYSIS USERMISSING=EXCLUDE /CRITERIA ALPHA=0.05 CILEVEL=95.
Resources	Processor Time	00:00:01.20
	Elapsed Time	00:00:01.26

Hypothesis Test Summary

	Null Hypothesis	Test	Sig.[a,b]
1	The distribution of Dis5 is the same across categories of S5.	Independent-Samples Kruskal-Wallis Test	<.001
2	The distribution of Ang5 is the same across categories of S5.	Independent-Samples Kruskal-Wallis Test	.050

Hypothesis Test Summary

	Decision
1	Reject the null hypothesis.
2	Retain the null hypothesis.

a. The significance level is .050.

b. Asymptotic significance is displayed.

Independent-Samples Kruskal-Wallis Test

Dis5 across S5

Independent-Samples Kruskal-Wallis Test Summary

Total N	240
Test Statistic	16.978[a]
Degree Of Freedom	3
Asymptotic Sig.(2-sided test)	<.001

a. The test statistic is adjusted for ties.

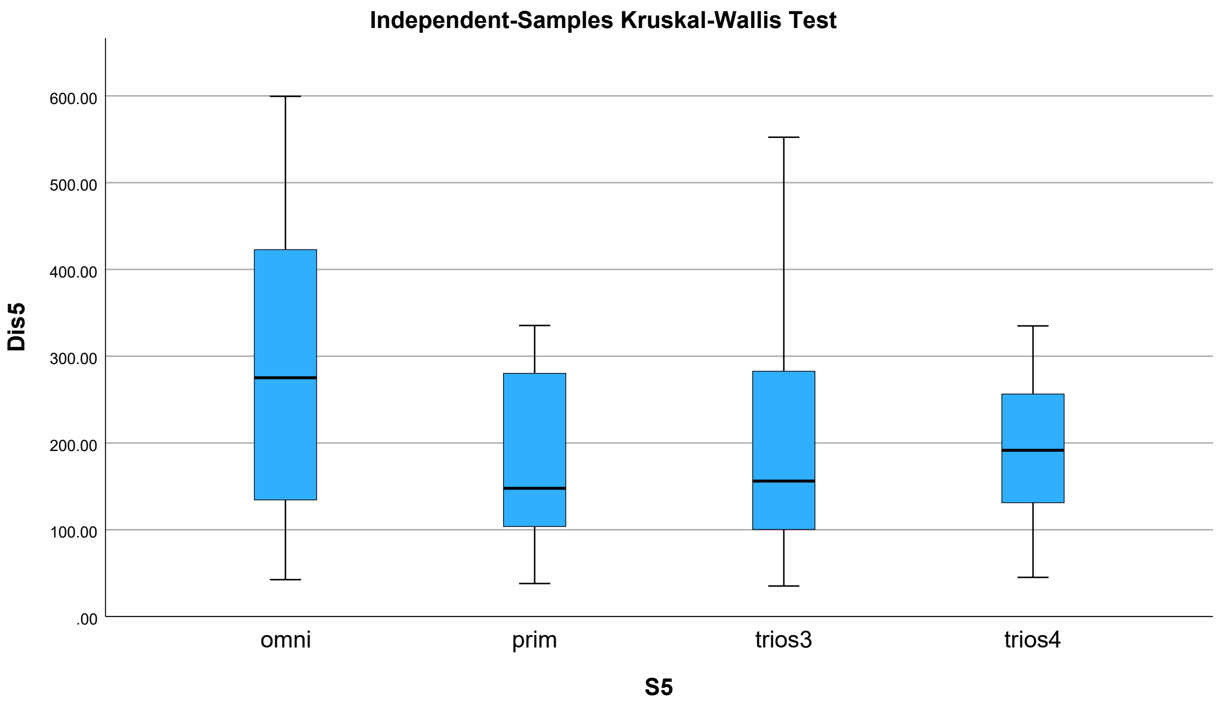

Pairwise Comparisons of S5

Sample 1-Sample 2	Test Statistic	Std. Error	Std. Test Statistic	Sig.	Adj. Sig.[a]
prim-trios3	-7.600	12.675	-.600	.549	1.000
prim-trios4	-12.050	12.675	-.951	.342	1.000
prim-omni	48.017	12.675	3.788	<.001	.001
trios3-trios4	-4.450	12.675	-.351	.726	1.000
trios3-omni	40.417	12.675	3.189	.001	.009
trios4-omni	35.967	12.675	2.838	.005	.027

Each row tests the null hypothesis that the Sample 1 and Sample 2 distributions are the same.
Asymptotic significances (2-sided tests) are displayed. The significance level is .050.

a. Significance values have been adjusted by the Bonferroni correction for multiple tests.

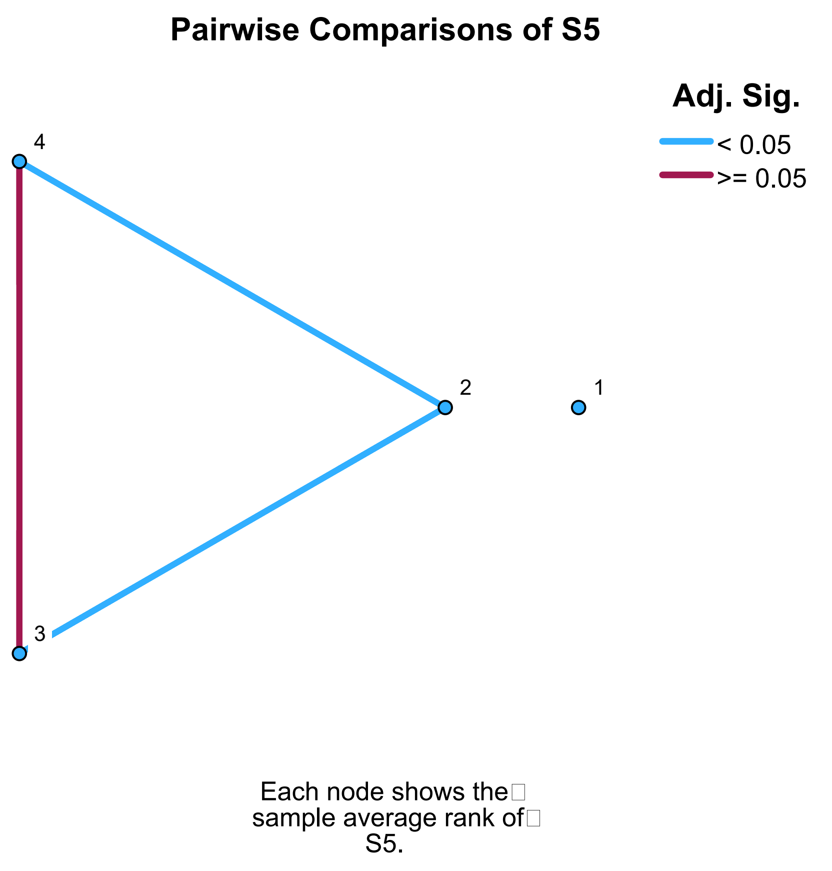

Ang5 across S5

Independent-Samples Kruskal-Wallis Test Summary

Total N	240
Test Statistic	7.810[a]
Degree Of Freedom	3
Asymptotic Sig.(2-sided test)	.050

a. The test statistic is adjusted for ties.

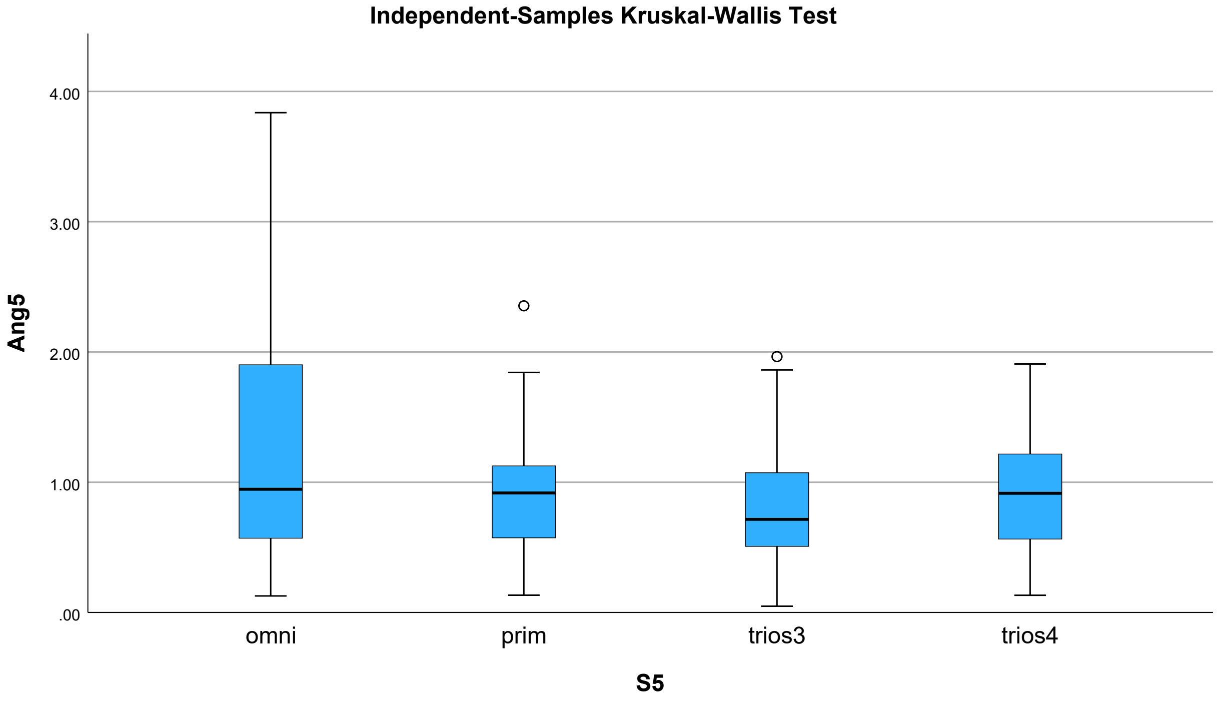

Pairwise Comparisons of S5

Sample 1-Sample 2	Test Statistic	Std. Error	Std. Test Statistic	Sig.	Adj. Sig.[a]
trios3-prim	14.433	12.675	1.139	.255	1.000
trios3-trios4	-14.733	12.675	-1.162	.245	1.000
trios3-omni	35.167	12.675	2.774	.006	.033
prim-trios4	-.300	12.675	-.024	.981	1.000
prim-omni	20.733	12.675	1.636	.102	.611
trios4-omni	20.433	12.675	1.612	.107	.642

Each row tests the null hypothesis that the Sample 1 and Sample 2 distributions are the same.
Asymptotic significances (2-sided tests) are displayed. The significance level is .050.

a. Significance values have been adjusted by the Bonferroni correction for multiple tests.

Pairwise Comparisons of S5

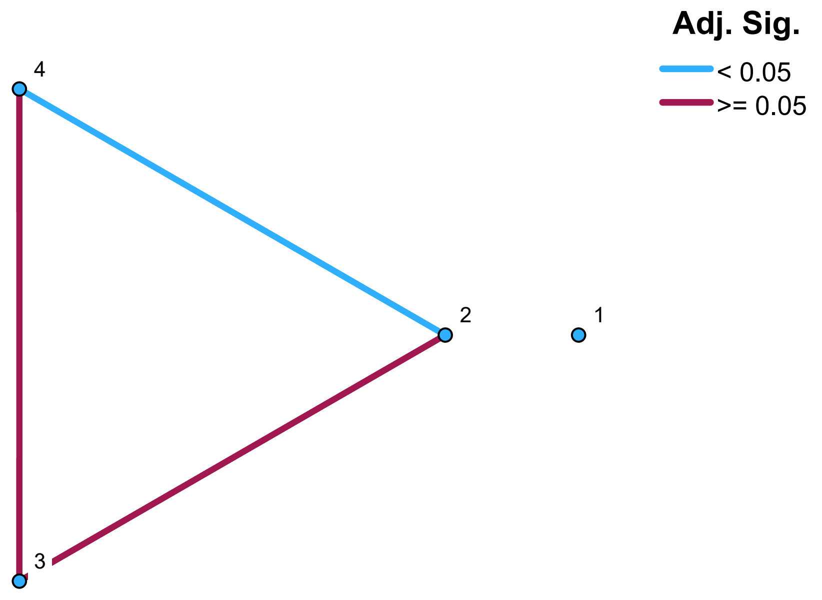

Each node shows the sample average rank of S5.

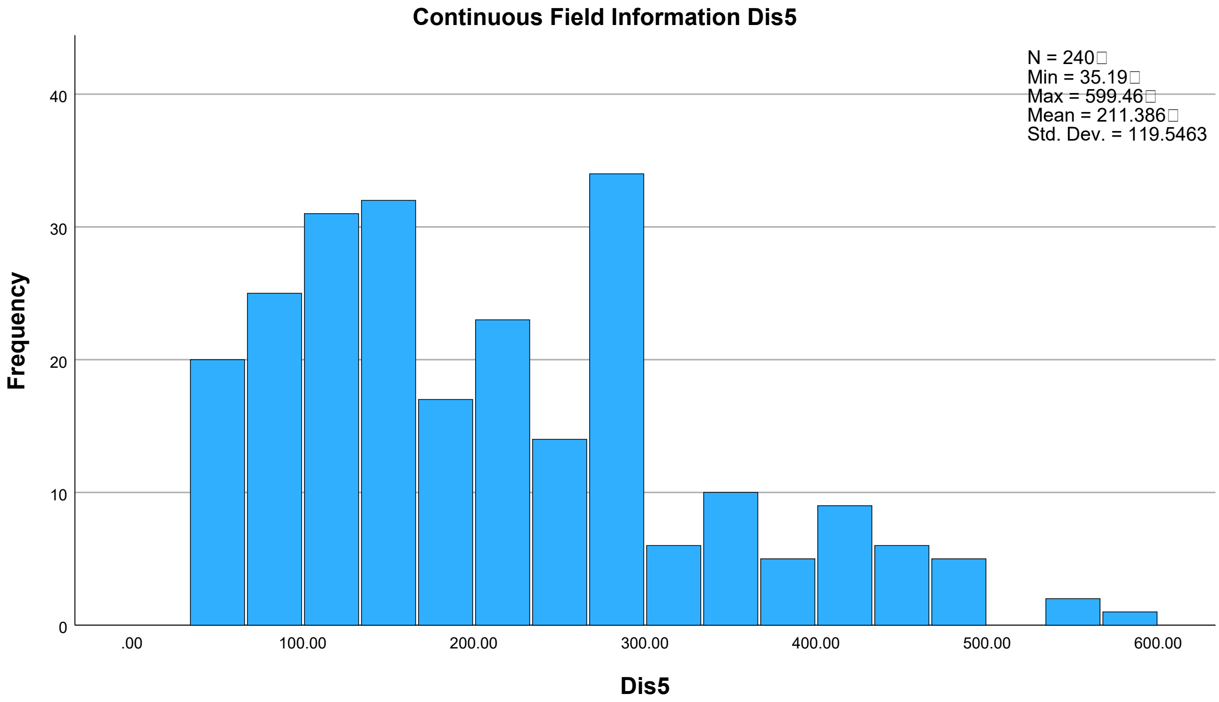
Continuous Field Information Dis5
N = 240
Min = 35.19
Max = 599.46
Mean = 211.386
Std. Dev. = 119.5463
Frequency
0
10
20
30
40
.00
100.00
200.00
300.00
400.00
500.00
600.00
Dis5

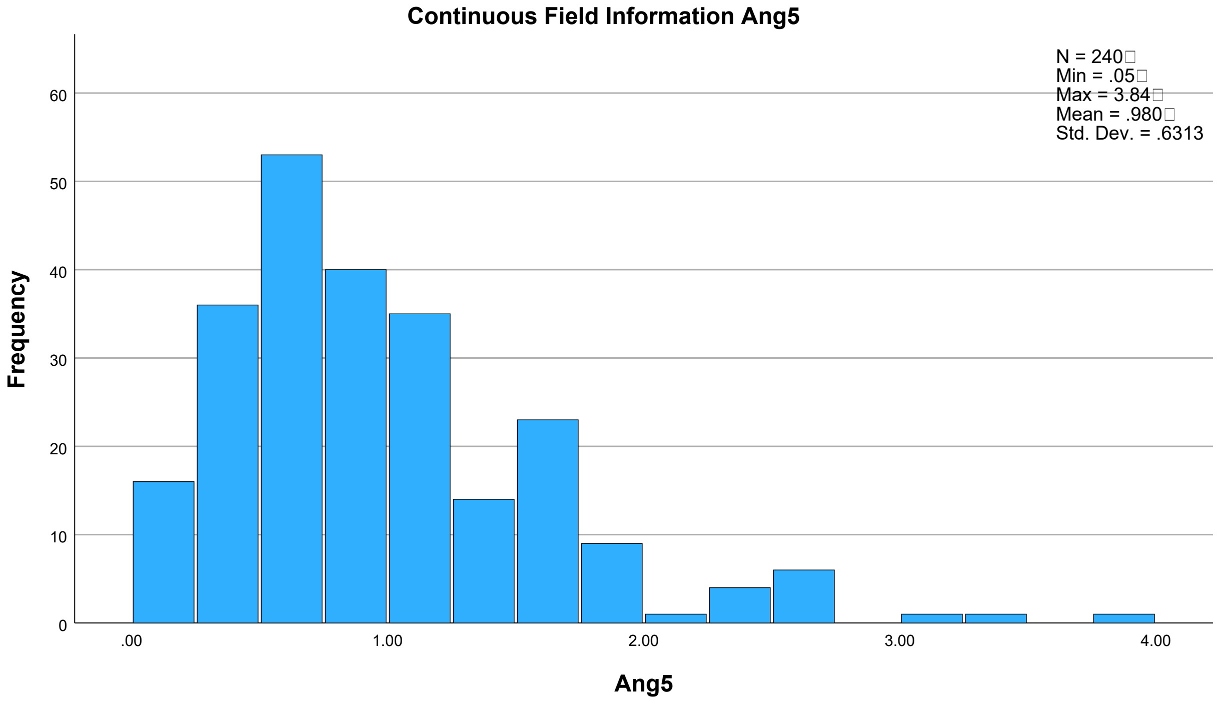
Continuous Field Information Ang5
N = 240
Min = .05
Max = 3.84
Mean = .980
Std. Dev. = .6313
Frequency
0
10
20
30
40
50
60
.00
1.00
2.00
3.00
4.00
Ang5

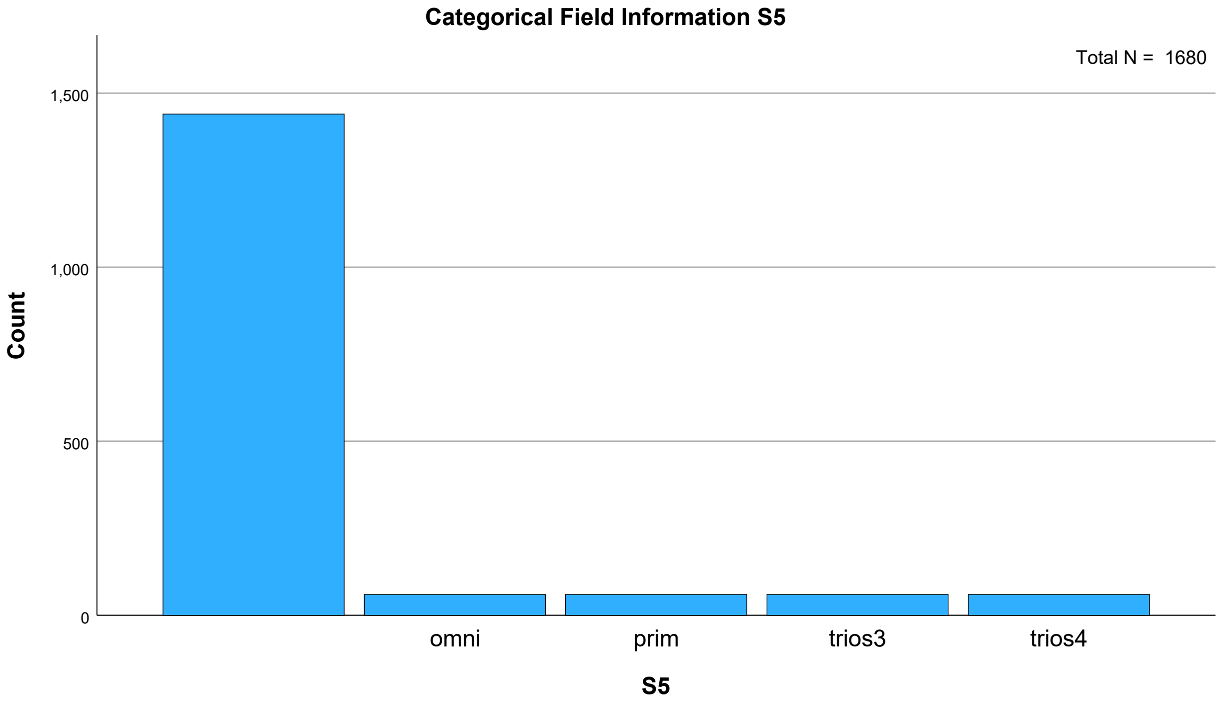
Categorical Field Information S5
Total N = 1680
1,500
1,000
500
0
Count
omni
prim
trios3
trios4
S5
